# Diagnostic accuracy of teledermatology for skin diseases: a systematic review and meta-analysis

**DOI:** 10.3389/fmed.2026.1739592

**Published:** 2026-03-02

**Authors:** Katalin Martyin, Fanni Adél Meznerics, Laura Anna Bokor, Bence Szabó, Péter Hegyi, Norbert Kiss, András Bánvölgyi

**Affiliations:** 1 Faculty of Medicine, Department of Dermatology, Venereology and Dermatooncology, Semmelweis University, Budapest, Hungary; 2 Centre for Translational Medicine, Semmelweis University, Budapest, Hungary; 3 Institute of Pancreatic Diseases, Semmelweis University, Budapest, Hungary; 4 Institute for Translational Medicine, Medical School, University of Pécs, Pécs, Hungary

**Keywords:** meta-analysis, skin cancer, teledermatology, telehealth, telemedicine

## Abstract

**Background:**

Skin diseases affect nearly one-third of the global population, yet limited access to dermatological care remains an unmet challenge. Teledermatology offers a promising solution, however, concerns about technological and workforce demands have limited its broader adoption. Furthermore, its diagnostic reliability across communication platforms and types, and different skin conditions remains unclear.

**Objectives:**

We aimed to evaluate the diagnostic accuracy of teledermatology compared to in-person consultations.

**Methods:**

We searched PubMed, Embase, and CENTRAL on November 19, 2023, for observational and experimental studies, without date or language restrictions. Primary outcomes included diagnostic concordance, Cohen’s kappa, sensitivity, specificity, and predictive values; secondary outcomes were diagnostic time, teledermatology provider and patient satisfaction and interrater agreement. A random intercept logistic regression model was used to pool outcomes. Subgroup analyses were conducted by disease category, communication platform (store-and-forward, real-time, hybrid), type (indirect, direct), dermoscopy use, photography device, and training.

**Results:**

Out of 30,412 records, 155 studies were included, with 139 analyzed quantitatively. Diagnostic concordance was 76% in all skin conditions (95%-CI: 73–79%), 73% in skin cancers (95%-CI: 67–79%) and 76% in pigmented lesions (95%-CI: 67–83%). Use of dermoscopy significantly improved diagnostic concordance from 67% (95%-CI: 58–74%) to 80% (95%-CI, 73–85%) in skin cancers. No significant differences were found by communication type, platform, or photography device. The mean diagnostic time was 1.05 min per case (95%-CI, 0.98–1.12). Patient satisfaction was high (82%, 95%-CI: 76–87%).

**Conclusion:**

Teledermatology demonstrates high diagnostic accuracy, supporting its use as a reliable alternative to in-person care for diagnosing general skin conditions and screening for skin cancer. Given its broad applicability, teledermatology stands out as a potential tool to improve access to dermatological care.

**Systematic review registration:**

https://www.crd.york.ac.uk/PROSPERO/view/CRD42023484476, identifier CRD42023484476.

## 1 Introduction

Skin diseases are a major public health concern, affecting nearly one-third of the world population, and ranking among the leading causes of nonfatal burden globally (1, 2). Moreover, the incidence of skin diseases is rising, with a total demand projected to rise by 12.45% from 2021 to 2036 (3). However, the growing demand for dermatological care remains largely unmet worldwide, with some countries having less than one dermatologist per one million people (4). Although this issue weighs the heaviest on low-income countries, middle- and high-income countries also experience a maldistribution of dermatologists (5). Recognizing this gap, the World Health Organization (WHO) urged to integrate telemedicine platforms into clinical practice, especially in remote areas (6).

Teledermatology provides dermatological care between two distant locations through telecommunication technologies (7). It may offer a solution by reducing waiting times and making patient care more accessible in rural and low-resource areas (8). Although it has been shown to be effective during the COVID-19 pandemic in reducing the need for in-person visits, there is a lack of evidence for its applicability in daily practice (9).

Teledermatology can be categorized by communication platform and type. According to communication platform, there are three main forms: store-and-forward, real-time, and hybrid methods (10). In the case of the store-and-forward method, the connection between the patient and healthcare provider is separated in both time and space. Clinical images may also be complemented by historical and/or clinical information (10). The real-time method uses video conferencing technology for live interactions, while the hybrid method combines the features of both approaches (10). The two types of communication are direct and indirect forms. In the direct form, the patient contacts the dermatologist directly, while in indirect teledermatology, the interaction is mediated by the referring healthcare provider, usually a general practitioner (11). The indirect form can be enhanced by the use of dermoscopy (12).

In light of these factors, teledermatology stands out as a promising tool for facilitating timely and affordable dermatological care worldwide. However, its wider implementation has been constrained by technological and human resource limitations. In addition, its diagnostic reliability across communication platforms and types, as well as its efficacy for evaluating different skin conditions remains uncertain (13). We aimed to comprehensively evaluate the diagnostic accuracy of teledermatology compared to in-person dermatological examinations.

## 2 Materials and methods

### 2.1 Search strategy and selection criteria

We report our systematic review and meta-analysis following the recommendations of the PRISMA 2020 guidelines and the Cochrane Handbook (Version 6.4) (14, 15). The review protocol was registered in PROSPERO [registration number: CRD42023484476, available at https://www.crd.york.ac.uk/PROSPERO/view/CRD42023484476].

A systematic search was conducted in three databases: MEDLINE (via PubMed), Embase, and Cochrane Central Register of Controlled Trials (CENTRAL) on November 19, 2023, without any filters or language restrictions. The detailed search strategy is available in the Supplementary material.

Peer-reviewed observational (cohort, cross-sectional, and case–control) studies and experimental studies (randomized clinical trials, and non-randomized controlled trials) were included if they met the following Population-Intervention-Comparator-Outcome (PICO) framework: (P) patients with dermatological diseases, (I) diagnosis made via teledermatology, (C) diagnosis made during face-to-face visit or histopathology, (O) primary: diagnostic concordance, Cohen’s kappa, sensitivity, specificity, and predictive values, secondary: diagnostic time, teledermatology provider and patient satisfaction, interrater agreement between teledermatology providers, and between face-to-face dermatologists.

Articles were included if both teledermatology and face-to-face evaluation were performed by a dermatology specialist or resident.

After a reference library was created, Endnote 20 (Clarivate Analytics, Philadelphia, PA, USA) was used for automatic and manual removal of duplicates. Title and abstract selection, as well as full-text selection were performed using the Rayyan Systems (Qatar Computing Research Institute, Cambridge, MA, USA/ Doha, Qatar) software. The selection was independently performed by two reviewers (KM, LAB), and a third reviewer (FAM) resolved disagreements.

Two authors (KM, LAB) independently extracted data from eligible articles into a standardized data collection table. The following data were collected: title, first author, year of publication, country, study type, study period, study setting, patient characteristics, intervention details, comparator, primary and secondary outcomes. If available, both summary estimates and individual patient-level data were extracted. Disagreements were resolved by a third reviewer (FAM).

### 2.2 Data analysis

A random-effects model was used to pool effect sizes, as considerable between-study heterogeneity was assumed in all cases.

Proportions were considered as an effect size measure with 95% confidence intervals (CI), for the following outcomes: diagnostic concordance, Cohen’s kappa, sensitivity, specificity, predictive values, interrater agreement, and satisfaction. The number of all patients and the number of matching diagnoses were extracted to calculate study proportions and pooled proportions. If raw numbers were not reported, the point and interval estimates (95% CI, standard error) of the above-mentioned two ratios were extracted. A random intercept logistic regression model was used for pooling outcomes (16, 17). The maximum likelihood method was used to estimate the heterogeneity variance measure (τ^2^). The Clopper-Pearson method was used for the calculation of CIs of proportions of the individual studies (18). For the continuous outcome (diagnostic time), the difference between the mean (MD) was used for the effect size measure with a 95% CI.

Articles were grouped by disease category. Multilevel modeling with study ID as a random effect was used to account for repeated entries from the same article within analyses. Studies were classified and analyzed separately according to whether undiagnosed cases were treated as diagnostic disagreements or excluded from the analysis. Studies reporting both approaches were included in both analyses.

Subgroup analyses were performed in each disease group based on communication platform (store-and-forward, real-time, hybrid), communication type (direct, indirect), the use of dermoscopy, photography device (smartphone or tablet, photo or video camera), training for image acquisition, and comparator (face-to-face examination, histopathology).

The results of all analyses were visualized in forest plots. To perform all calculations, we used the R version 4.2.0 (R Foundation for Statistical Computing, Vienna, Austria) software, supplemented with the meta and metafor packages.

I^2^ and χ^2^ tests were used to assess the statistical heterogeneity, with a *p*-value <0.1 as a threshold for statistically significant difference. Egger’s test and funnel plots were applied to report and visualize publication bias if there were at least ten studies involved in the analysis (19).

The risk of bias was independently assessed by two reviewers (KM, LAB), using the QUADAS-2 tool (University of Bristol, Bristol, UK). Disagreements were resolved by a third reviewer (FAM).

## 3 Results

### 3.1 Study selection

**Figure 1:**
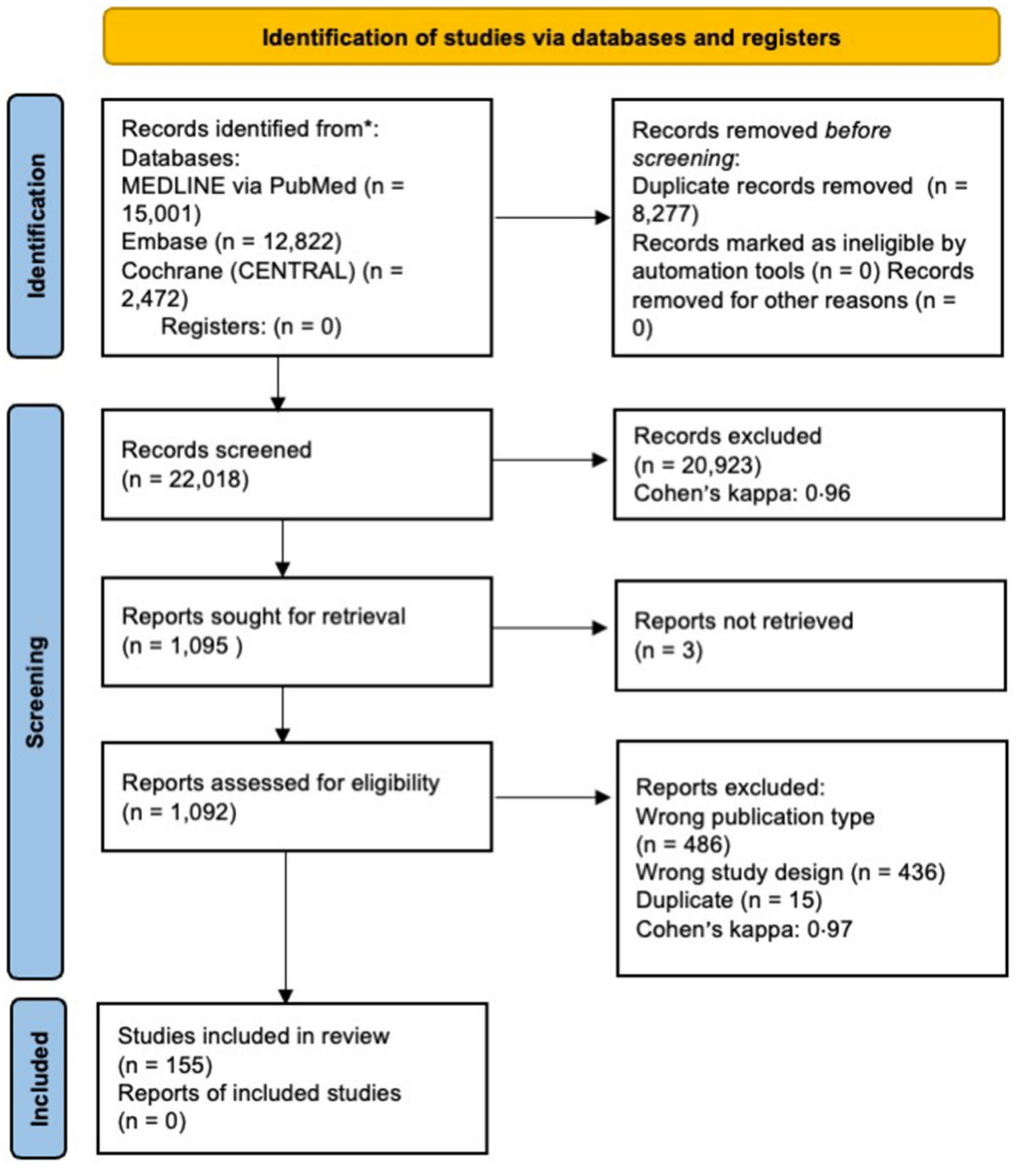
PRISMA 2020 flow diagram of the selection and screening process.

A total of 30,412 records were identified through the systematic search. After duplicate removal and selection, 155 articles were included (20–175), most of which were observational studies. Quantitative analysis was performed on 139 studies (20–159), while 16 articles were only qualitatively assessed (160–175). The flowchart of the selection and screening process is summarized in Figure 1.

### 3.2 Characteristics of studies

The main characteristics of the included articles are presented in Table 1. Additional study and patient characteristics are shown in Supplementary Table S2.

**Table 1:** Characteristics of the included studies.

First author and year of publication	Study design	Study period	Country	Population	Communication method	Communication type	Photography device	Use of dermoscopy or other imaging method	Comparator	Reported outcome
Articles included in the meta-analysis										
Altieri, 2017 (20)	Observational (prospective)	February 2007–June 2007	USA	All skin conditions	SAF	ID	DC	no	FTF	DgC, CK
Baba, 2005 (21)	Observational (prospective)	May 2003–July 2003	Turkey	All skin conditions	SAF / HY	ID	DC / VC	no	FTF ± HP	DgC, CK, IA-TD, P-SAT
Barbieri, 2014 (22)	Observational (prospective)	September 1, 2012 - April 31, 2013	USA	Hospitalized patients, all skin conditions	SAF	ID	SP	no	FTF	DgC, CK, IA-TD
Barcaui, 2018 (23)	Observational (prospective)	April 2017–June 2017	USA	Pigmented lesions	SAF	ID	SP	DS	FTF ± HP	DgC, CK
Barnard, 2000 (24)	Observational (prospective)	NA	USA	All skin conditions	SAF	ID	DC	no	FTF ± HP, HP	DgC, CK
Batalla, 2016/1 (25)	Observational (retrospective)	May 2011–January 2015	Spain	All skin conditions	SAF	ID	NA	no	FTF	DgC
Batalla, 2016/2 (26)	Observational (cross-sectional)	May 2011–April 2014	Spain	All skin conditions	SAF	ID	NA	no	FTF	DgC
Borve, 2012 (27)	Observational (prospective)	NA	Sweden	All skin conditions	SAF	ID	SP	no	FTF	DgC, CK, IA-TD
Borve, 2013 (28)	Observational (prospective)	NA	Sweden	Suspected skin cancer	SAF	ID	SP	DS	HP	DgC, CK, IA-TD
Bowns, 2006 (29)	RCT	NA	UK	All skin conditions	SAF	ID	DC	no	FTF ± HP	DgC, CK, P-SAT
	Observational (prospective)	NA	UK	Suspected skin cancer	SAF	ID	DC	DS	FTF	CK^a^
Braun, 2000 (30)	Observational (prospective)	NA	Switzerland	Pigmented lesions suspicious of skin cancer	SAF	ID	DC	DS	HP	DgC, CK
Carter, 2017 (31)	Observational (prospective cohort, retrospective review)	May 2013–December 2014	USA	All skin conditions	SAF	ID	DC	no	FTF	DgC, CK, IA-TD
Castillo, 2022 (32)	Observational (retrospective)	March 2020–December 2020	USA	All skin conditions	SAF / RT	D	NA	no	HP	DgC, CK
Cazzaniga, 2016 (33)	Observational (retrospective)	July 1, 2015 -December 31, 2015	Italy	Suspected skin cancer	SAF	D	NA	no	HP	DgC
Chan, 2000 (34)	Observational (prospective)	NA	Hong Kong, China	All skin conditions	RT	ID	VC	no	FTF	DgC, CK
Chao, 2003 (35)	Observational (prospective)	April 2002–August 2002	Brazil	All skin conditions	SAF	ID	NA	no	FTF	DgC, CK, Time
Chen, 2010 (36)	Observational (retrospective)	January 1, 2002 - May 1, 2006	USA	All skin conditions	SAF	ID	NA	no	FTF	CK^a^
Cheung, 2018 (37)	Observational (retrospective)	September 2014–March 2016	UK	Solitary skin lesions	SAF	ID	DC	DS	FTF, HP	CK^a^
Chung, 2007 (38)	Observational (prospective)	NA	USA	Hospitalized patients, all skin conditions	SAF	ID	DC	no	FTF	DgC
Clarke, 2023 (39)	Observational (prospective)	October 2019–February 2020	USA	All skin conditions	SAF	ID	DC	no	FTF ± HP, HP	DgC, CK, IA-TD
Congalton, 2015 (40)	Observational (prospective)	April 1, 2012 - March 31. 2014	New Zealand	Suspected melanoma	SAF	ID	DC	DS	HP	DgC, CK, Sens, Spec, PPV, NPV
Coras, 2003 (41)	Observational (prospective)	NA	Germany	Pigmented skin lesions	SAF	ID	DC	DS	FTF ± HP	CK^a^
D’elia, 2007 (42)	Observational (prospective)	NA	Brazil	All skin conditions	SAF	ID	DC	no	FTF	DgC, IA-TD
de Giorgi, 2016 (43)	Observational (prospective)	NA	Italy	Pigmented lesions	SAF	ID	DC	DS	HP	DgC, CK
Dobry, 2021 (44)	Observational (retrospective)	March 2014–December 2017	USA	All skin conditions	SAF	ID	NA	no	FTF	DgC, CK
Du Moulin, 2003 (45)	Observational (prospective)	June 2000–May 2001	The Netherlands	All skin conditions	SAF	ID	DC	no	FTF ± HP	DgC, CK
Edison, 2008 (46)	Observational (prospective)	NA	USA	All skin conditions	SAF, RT	ID	NA	no	FTF	DgC, CK
Eminovic, 2003 (47)	Observational (prospective)	NA	The Netherlands	All skin conditions	SAF	ID	NA	no	FTF	CK^a^, Time^a^
Fabbrocini, 2008 (48)	Observational (prospective)	NA	Italy	Melanocytic and non-melanocytic “pink” lesions (poor/absent pigmentation)	SAF	ID	DC	DS	HP	CK, IA-TD
Faucon, 2022 (49)	Observational (prospective)	November 2016–January 2020	France	All skin conditions	SAF	ID	NA	no	FTF ± HP	CK^a^
Ferrandiz, 2017 (50)	RCT	January 1, 2015 - December 31, 2015	Spain	Suspected skin cancer	SAF	ID	DC	DS	FTF	DgC, CK, Sens, Spec, PPV, NPV, Time
Gabel, 2021 (51)	Observational (retrospective)	July 2023–August 2013	USA	All skin conditions	SAF	ID	DC / tablet	no	FTF	DgC, CK
Gao, 2023 (52)	Observational (retrospective)	July 1, 2017 - December 31, 2017	New Zealand	Suspected skin cancer	SAF	ID	NA	DS	HP	DgC, CK, Time
Gatica, 2015 (53)	Observational (prospective)	NA	Chile	All skin conditions	SAF	ID	DC	no	FTF	DgC
Gemelas, 2019 (54)	Observational (retrospective)	February 1, 2015 - January 31, 2016	USA	Suspected or confirmed melanoma	SAF	ID	DC	DS	FTF ± HP	DgC, CK, Sens
Gerhardt, 2021 (55)	Observational (retrospective)	January 2017–December 2017	USA	All skin conditions	SAF	D	NA	no	FTF	DgC, CK
Giavina-Bianchi, 2020/1 (56)	Observational (retrospective)	July 2017–July 2018	Brazil	Skin neoplasms	SAF	ID	SP	no	FTF, HP	CK^a^
Gilmour, 1998 (57)	Observational (prospective, multicenter)	September 1995–September 1996	UK	All skin conditions	RT	ID	VC	no	FTF	DgC
Gyllencreutz, 2017 (58)	Observational (retrospective)	NA	Sweden	Suspected skin cancer	SAF	ID	SP / NA	DS / no	FTF ± HP	DgC, CK, IA-TD
Gyllencreutz, 2018 (59)	Observational (retrospective)	NA	Sweden	All skin conditions	SAF	ID	SP	DS	FTF ± HP	DgC, CK
Harrison, 1998 (60)	Observational (prospective)	NA	UK	Pigmented lesions	SAF	DC	DC	no	HP	DgC, Sens, P-SAT
Heffner, 2009 (61)	Observational (prospective)	July 2006–August 2007	USA	Rashes	SAF	ID	DC	no	FTF	DgC, CK, IA-TD
Herrmann, 2005 (62)	Observational (prospective)	February 2003–April 2003	Germany	All skin conditions	SAF	ID	DC	no	FTF	DgC
High, 2000 (63)	Observational (prospective)	September 4, 1997 - October 15, 1997	USA	All skin conditions	SAF	ID	DC	no	FTF	DgC, CK
Hines, 2021 (64)	Observational (retrospective)	January 1, 2015 - December 31, 2019	USA	Emergency department patients, all skin conditions	SAF	ID	SP	no	FTF	DgC, CK
Hue, 2015 (65)	Observational (prospective)	January 2015–December 2015	France	Suspected melanoma	SAF	ID	SP	DS	FTF ± HP	DgC, CK
Ilie, 2022 (66)	Observational (prospective)	March 2020–April 2020	UK	All skin conditions	SAF	D	SP	no	FTF	DgC, Time
Ishioka, 2009 (67)	Observational (retrospective)	2005–2007	Brazil	Pigmented skin lesions	SAF	ID	DC	DS	HP	DgC, CK, Sens, Spec, PPV, NPV
Fazil Jaber, 2023 (68)	Observational (retrospective cross-sectional)	January 1, 2020 - June 30, 2020	Sweden	Atypical, pigmented lesions	SAF	ID	DC	DS	HP	CK^a^
Janda, 2020 (69)	RCT (open-label)	March 6, 2017 - June 7, 2018	Australia	Suspected skin cancer	SAF	D	SP	DS	FTF	DgC, IA-TD, IA-FTF
Jang, 2002 (70)	Observational (prospective)	NA	South Korea	All skin conditions	SAF	ID	DC	no	FTF	DgC
Jobbagy, 2022 (71)	Observational (retrospective)	March 2020–July 2020	Hungary	All skin conditions	SAF	D	NA	no	FTF ± HP	DgC, CK, Sens, Spec, PPV, NPV
Jolliffe, 2001 (72)	Observational (prospective)	NA	UK	Pigmented skin lesions	SAF	ID	VC	no	HP	DgC
Jones, 2021 (73)	Observational (retrospective)	July 1, 2016 - December 31, 2020	New Zealand	Suspected skin cancer	SAF	ID	SP	DS	HP	DgC
Josendal, 1991 (74)	Observational (prospective)	NA	Norway	All skin conditions	RT	ID	DC	no	FTF	DgC, CK
Kaliyadan, 2013 (75)	Observational (prospective)	NA	Saudi Arabia	All skin conditions	SAF	ID	SP	no	FTF	DgC, CK, P-SAT, TD-SAT
Keller, 2020 (76)	Observational (prospective)	November 2017–August 2018	USA	All skin conditions	SAF	ID	SP / tablet	no	FTF	DgC, CK
Koop, 2023 (77)	Observational (retrospective)	October 16, 2017 - August 30, 2019	Estonia	Suspected melanoma	SAF	ID	NA	DS	HP	DgC, CK, Sens, Spec, PPV, NPV
Kravets, 2018 (78)	Observational (prospective)	2013–2016	Ukraine	Skin neoplasms	SAF	ID	DC	DS	HP, FTF	DgC, CK
Kroemer, 2011 (79)	Observational (prospective)	NA	Austria	Skin neoplasms	SAF	ID	SP	DS	FTF ± HP	CK^a^
Krupinski, 1999 (80)	Observational (prospective)	NA	USA	All skin conditions	SAF	ID	DC	no	FTF, HP	DgC, CK, Time
Kvedar, 1997 (81)	Observational (prospective)	March 1995–April 1995	USA	All skin conditions	SAF	ID	DC	no	FTF	DgC, CK
Lamel, 2012 (82)	Observational (prospective)	NA	USA	Skin cancer screening	SAF	ID	SP	no	FTF	DgC, CK
Lasierra, 2012 (83)	Observational (prospective)	April 2008–July 2010	Spain	All skin conditions	SAF	ID	DC	no	FTF	DgC, Time
Lepe, 2004 (84)	Observational (prospective)	NA	Mexico	All skin conditions	SAF	ID	VC	no	FTF	DgC, CK
Lesher, 1998 (85)	Observational (prospective)	NA	USA	All skin conditions	RT	ID	VC	no	FTF	DgC, CK, IA-FTF
Lim, 2001 (86)	Observational (prospective)	NA	Australia	All skin conditions	SAF	ID	DC	no	FTF	DgC, CK
Loane, 1997 (87)	Observational (prospective)	NA	UK	All skin conditions	RT	ID	VC	no	FTF	DgC
Loane, 1998 (88)	Observational (prospective)	NA	UK	All skin conditions	RT	ID	VC	no	FTF	DgC
Lowitt, 1998 (89)	Observational (prospective)	NA	USA	All skin conditions	RT	ID	VC	no	FTF	DgC, CK, Sens, Spec TD-SAT
Lyon, 1997 (90)	Observational (prospective)	NA	UK	All skin conditions	SAF	ID	DC	no	FTF, HP	DgC, CK
Maclellan, 2021 (91)	Observational (prospective)	NA	Canada	Pigmented lesions	SAF	ID	DC	DS	HP	DgC, CK, Sens, Spec
Mahendran, 2005 (92)	Observational (prospective)	NA	UK	Suspected skin cancer	SAF	ID	DC	no	FTF	DgC, CK
Mallett, 2003 (93)	Observational (retrospective)	October 1998–January 2003	UK	All skin conditions	SAF	ID	DC	no	FTF	DgC, CK
Manahan, 2015 (94)	Observational (prospective)	May 2013–November 2013	Australia	Pigmented lesions	SAF	D	SP	DS	FTF	CK^a^
Marchell, 2017 (95)	Quasi-randomized controlled trial	NA	USA	All skin conditions	SAF	ID	DC	no	FTF	DgC, CK
Markun, 2017 (96)	Observational (prospective)	May 2013–June 2016	Switzerland	Suspected skin cancer	SAF	ID	SP	DS	FTF ± HP	CK^a^
Massone, 2007 (97)	Observational (prospective)	NA	Austria	Pigmented lesions	SAF	ID	DC	DS	FTF	CK, IA-TD
Massone, 2013 (98)	Observational (prospective)	February 2008–February 2010	Austria	Suspected skin cancer	SAF	ID	DC	DS	FTF ± HP	DgC, CK, Sens, Spec
Montejano, 2022 (99)	Observational (retrospective)	September 2018–March 2019	USA	All skin conditions	SAF	ID	NA	DS	HP	DgC, CK
Moreno-Ramirez, 2005 (100)	Observational (prospective)	January 2004–April 2004	Spain	Pigmented lesions	SAF	ID	DC	no	FTF	DgC, CK, P-SAT
Moreno-Ramirez, 2006 (101)	Observational (prospective pilot)	September 2004–January 2005	Spain	Pigmented lesions	SAF	ID	DC	DS	HP	CK, Sens, Spec
Moreno-Ramirez, 2007 (102)	Observational (longitudinal)	March 2004–July 2005	Spain	Suspected skin cancer	SAF	ID	DC	no	FTF ± HP	DgC, CK, Sens, Spec, PPV, NPV
Muir, 2011 (103)	Observational (prospective pilot)	August 2008–August 2009	Australia	Emergency department patients, acute/subacute skin conditions	SAF	ID	DC	no	FTF	CK, Time
Naka, 2018 (104)	Observational (retrospective)	June 2014–November 2015	USA	All skin conditions	SAF	ID	DC	DS	FTF ± HP	CK, P-SAT
Nami, 2015 (105)	Observational (prospective)	October 2011–October 2012	Italy, Austria	All skin conditions, except pigmented lesions	SAF	ID	SP	no	FTF	CK, Time
Ng, 2011 (106)	Observational (retrospective)	October 2007–April 2008	UK	Suspected skin cancer	SAF	ID	NA	no	FTF ± HP	DgC, CK, PPV, NPV
Nordal, 2001 (107)	Observational (prospective)	1994–1995	Norway	All skin conditions	RT	ID	DC	no	FTF	CK
Norton, 1997 (108)	Observational (prospective)	NA	USA	All skin conditions	RT	ID	NA	no	HP	DgC, CK
O’Connor, 2017 (109)	RCT, observational (prospective)	March 1, 2016 - September 30, 2016	USA	All skin conditions	SAF	D	SP	no	FTF	CK
Oakley, 1997 (110)	Observational (prospective)	NA	New Zealand	All skin conditions	RT	ID	DC	no	FTF	DgC, P-SAT
Oakley, 1998 (111)	Observational (prospective)	NA	New Zealand	All skin conditions	RT	ID	DC	no	FTF, HP	DgC, CK
Oakley, 2006 (112)	Observational (prospective)	NA	New Zealand	All skin conditions	SAF	ID	DC	no	HP	CK
Okita, 2016 (113)	Observational (prospective)	January 2015–April 2015	Brazil	Hospitalized patients, all skin conditions	SAF	ID	SP	no	FTF	CK
Oztas, 2004 (114)	Observational (prospective)	NA	Turkey	All skin conditions	SAF	ID	DC	no	FTF	CK
Pak, 2003 (115)	Observational (prospective)	October 1, 1999 - January 30, 2000	USA	All skin conditions	SAF	ID	DC	no	FTF	CK
Paradela-De-La-Morena, 2015 (116)	Observational (retrospective)	2011–2013	Spain	All skin conditions	SAF	ID	DC	no	FTF	CK
Phillips, 1997 (117)	Observational (prospective)	NA	USA	All skin conditions	RT	ID	DC	DS	FTF	CK
Phillips, 1998 (118)	Observational (retrospective)	NA	USA	Skin cancer screening	RT	ID	DC	DS	FTF	CK
Preclaro, 2022 (119)	Observational (cross sectional)	August 1, 2018 - September 30, 2018	Philippines	All skin conditions	SAF	ID	DC	no	FTF, HP	CK, IA-TD
Rajagopal, 2009 (120)	Observational (retrospective)	March 6, 2009 - March 8, 2009	India	All skin conditions	SAF, RT	ID	VC	n.a.	FTF	CK
Rashid, 2003 (121)	Observational (prospective)	NA	Pakistan	All skin conditions	SAF	ID	DC	no	FTF	DgC, CK
Ribas, 2010 (122)	Observational (cross sectional)	NA	Brazil	All skin conditions	SAF	ID	DC	no	FTF	DgC, CK, IA-TD, IA-FTF
Rios, 2012 (123)	Quasi-experimental, randomized, open-label	April 1, 2009 - April 30, 2009	Spain	All skin conditions	SAF	ID	DC	no	FTF ± HP, FTF, HP	DgC
Romero, 2006 (124)	RCT	June 2004–December 2005	Spain	All skin conditions	SAF	ID	DC	no	FTF	DgC
Romero, 2010 (125)	Observational (prospective pilot)	August 2003–February 2004	Spain	All skin conditions	SAF /HY	ID	DC	no	FTF	DgC
Romero Aguilera, 2014 (126)	RCT	June 2004–December 2005	Spain	All skin conditions	SAF / HY	ID	DC	no	FTF	DgC
Rubegni, 2011 (127)	Observational (prospective)	January 2009–December 2009	Italy	All skin conditions	SAF	ID	DC	DS	FTF	DgC, CK
Ruiz, 2009 (128)	Observational (prospective)	NA	Colombia	All skin conditions	SAF	ID	DC	no	FTF	DgC, IA-TD, IA-FTF
Saleh, 2017 (129)	Observational (prospective)	February 2015–June 2015	Egypt	All skin conditions	SAF	ID	DC	no	FTF	DgC, CK, IA-TD, P-SAT
Santosa, 2023 (130)	Observational (retrospective)	June 2015–December 2015	Singapore	Emergency department patients, all skin conditions	SAF	ID	DC	no	FTF	DgC
Schiener, 2001 (131)	Observational (prospective pilot)	NA	Germany	All skin conditions	SAF	ID	DC	no	FTF	DgC
Senel, 2014 (132)	Observational (retrospective)	NA	Turkey	benign and malignant skin lesions	SAF	ID	DC	DS	FTF ± HP, HP	DgC, CK
Shin, 2014 (133)	Observational (prospective)	NA	South Korea	All skin conditions (military setting)	SAF	ID	SP	no	FTF	DgC, CK
Silva, 2009 (134)	Observational (prospective)	January 2007–April 2007	Brazil	All skin conditions	SAF	ID	DC	no	FTF	DgC
Silveira, 2019 (135)	Observational (prospective)	NA	Brazil	Suspected skin cancer	SAF	ID	DP	no	FTF	CK^a^
Sola-Ortigosa, 2020 (136)	Observational (prospective)	February 2016–March 2017	Spain	Suspected actinic keratosis	SAF	ID	DC	DS	FTF ± HP	DgC, PPV, NPV, IA-TD
Taberner Ferrer, 2009 (137)	Observational (prospective)	December 15, 2005 - July 4, 2008	Spain	All skin conditions	SAF	ID	DC	DS	FTF ± HP	DgC
Tait, 1999 (138)	Observational (prospective)	NA	Australia	All skin conditions	SAF	ID	DC	no	FTF	DgC
Tan, 2010 (139)	Observational (prospective)	March 2008–September 2008	New Zealand	All skin conditions	SAF	ID	DC	DS	FTF ± HP, FTF, HP	DgC, CK, Sens, Spec
Taslidere, 2022 (140)	Observational (prospective)	September 1, 2020 - December 1, 2020	Turkey	All skin conditions	SAF	ID	SP	no	FTF	DgC
Taslidere, 2023 (141)	Observational (prospective)	April 15, 2022 - November 1, 2022	Turkey	All skin conditions	SAF	ID	SP	no	FTF	DgC, CK
Taylor, 2001 (142)	Observational (prospective)	February 4, 1997 - May 2, 1997	UK	All skin conditions	SAF	ID	VC	no	FTF	DgC
Teague, 2022 (143)	Observational (retrospective)	January 1, 2012 - December 31, 2016	New Zealand	Suspected melanoma	SAF	ID	DC	DS	HP	DgC, CK, PPV
Teoh, 2022 (144)	Observational (retrospective)	January 1, 2010 - May 31, 2019	New Zealand	Suspected skin cancer	SAF	ID	DC	DS	HP	DgC, Sens
Tian, 2017 (145)	Observational (prospective)	NA	Singapore	Esthetic conditions	SAF	D	SP	no	FTF	DgC, P-SAT
Tucker, 2005 (146)	Observational (retrospective)	NA	UK	All skin conditions	SAF	ID	DC	no	FTF	DgC, CK
Vano-Galvan, 2011 (147)	Observational (cross sectional, repeated measures study)	January 2009–April 2009	Spain	All skin conditions	SAF	ID	DC	no	FTF	DgC, CK, Time
Villa, 2020 (148)	Observational (prospective)	March 2017–July 2017	Germany	Emergency department patients, all skin conditions	SAF	ID	tablet	no	FTF	DgC, CK
Wang, 2017 (149)	Observational (retrospective)	July 1, 2009 - December 31, 2011	USA	Suspected melanoma	SAF	ID	NA	DS	HP	DgC, CK
Warshaw, 2009/1 (150)	Observational (cross sectional, repeated measures study)	November 2002–August 2005	USA	Pigmented neoplasms	SAF	ID	DC	DS	HP	DgC, CK
Warshaw, 2015 (151)	Observational (cross sectional, repeated measures study)	NA	USA	Skin neoplasms	SAF	ID	DC	DS	FTF ± HP, FTF, HP	DgC, CK
Weingast, 2013 (152)	Observational (prospective)	NA	Austria	All skin conditions	SAF	D	SP	no	FTF	DgC, CK
Whited, 1998 (153)	Observational (prospective)	NA	USA	Suspected skin cancer	SAF	ID	DC	no	FTF	DgC, CK, IA-TD, IA-FTF
Whited, 1999 (154)	Observational (prospective)	NA	USA	All skin conditions	SAF	ID	DC	no	FTF	DgC, CK, IA-TD, IA-FTF
Yamazaki, 2003 (155)	Observational (prospective)	September 1999–August 2002	Japan	All skin conditions	RT	ID	SP	DS	FTF	DgC, CK
Zanini, 2013 (156)	Observational (prospective)	NA	Portugal	All skin conditions	SAF	ID	DC	no	FTF	DgC
Zelickson, 1997 (157)	Observational (prospective)	NA	USA	All skin conditions (nursing home patients)	SAF	ID	VC	no	FTF	DgC
Zink, 2017/1 (158)	Observational (prospective)	NA	Germany	All skin conditions	SAF	ID	SP	no	FTF	DgC
Zink, 2017/2 (159)	Observational (prospective)	NA	Germany	All skin conditions (in need of dermoscopic evaluation)	SAF	ID	SP	DS	FTF, HP	DgC
Articles only included in the systematic review										
Alfageme, 2021 (160)	Observational (prospective, multicenter)	June 2018–January 2019	Spain	Palpable nodular skin lesions	SAF	ID	US	US	FTF	DgC^a^, Sens^a^, Spec^a^, PPV^a^, NPV^a^
Creadore, 2023 (161)	Observational (cross sectional)	NA	USA	Cellulitis/pseudocellulitis	SAF	ID	NA	Thermal imaging	FTF	DgC
Giavina-Bianchi, 2020/2 (162)	Observational (retrospective)	July 2017–July 2018	Brazil	Atopic dermatitis	SAF	ID	SP	no	FTF	CK^a^
Giavina-Bianchi, 2020/3 (163)	Observational (retrospective)	July 2017–July 2018	Brazil	Inflammatory dermatoses	SAF	ID	SP	no	FTF	CK^a^
Lozzi, 2007 (164)	Observational (prospective)	September 2004–February 2005	Italy, Austria	Neoplastic and inflammatory dermatoses	SAF	ID	DC	no	HP	DgC
Ludzik, 2016/1 (165)	Observational (retrospective)	January 2015–May 2015	Poland, Italy	Suspected melanoma	SAF	ID	NA	DS, RCM	HP	DgC, Sens, Spec
Ludzik, 2016/2 (166)	Observational (retrospective)	January 2009–January 2012	Italy	Pink cutaneous lesions	SAF	ID	NA	DS, RCM	HP	DgC, Sens, Spec
Rao, 2013 (167)	Observational (retrospective)	June 2010–September 2011	USA	Lesions that had been selected for removal for either cosmetic or medical reasons	SAF	ID	NA	DS, RCM	HP	DgC, Sens, Spec
Senel, 2013 (168)	Observational (prospective)	April 2009–September 2009	Turkey	Non-melanocytic skin tumors	SAF	ID	DC	DS	FTF ± HP	DgC, CK
Shah, 2023 (169)	Observational (retrospective)	April 2020–October 2021	USA	Dermatitis	SAF	D	NA	no	FTF	DgC, CK
Tognetti, 2021 (170)	Observational (retrospective)	January 2018–March 2019	Italy	Atypical melanocytic lesions	SAF	ID	NA	DS	HP	AUROC
Trindade, 2008 (171)	Observational (prospective)	August 2005–April 2006	Brazil	Suspicious for leprosy	SAF	ID	DC	no	FTF	DgC, Sens, Spec
Tugrul, 2022 (172)	Observational (prospective)	April 2008–March 2019	Turkey	Non-melanocytic skin lesions	SAF	ID	SP	DS	FTF	DgC
Van der Heijden, 2013 (173)	Observational (prospective)	February 2010–May 2011	The Netherlands	Pigmented lesions	SAF	ID	DC	DS	FTF ± HP	CK, IA-TD
Warshaw, 2009/2 (174)	Observational (cross sectional)	NA	USA	Non-pigmented neoplasms	SAF	ID	DC	DS	HP	DgC
Witkowski, 2017 (175)	Observational (retrospective)	January 2010–August 2011	Italy, Poland, Spain	Suspected melanoma	SAF	ID	NA	DS, RCM	HP	Sens, Spec

NA – not applicable, RCT – randomized controlled trial, SAF – store-and-forward, RT – real-time, HY – hybrid, D – direct, ID – indirect, DC – digital camera, VC – video camera, SP – smartphone, DS – dermoscopy, RCM – reflectance confocal microscopy, US – ultrasound, FTF – face-to-face, HP – histopathology, DgC – diagnostic concordance, CK – Cohen’s kappa, Sens – sensitivity, Spec – specificity, PPV – positive predictive value, NPV – negative predictive value, IA-TD – interrater agreement between teledermatologists, IA-FTF – interrater agreement between in-person dermatologists, P-SAT – patient satisfaction, TD-SAT – teledermatologist satisfaction *Results reported only for analyses excluding undiagnosed cases.

### 3.3 Quantitative analysis

The included articles were assigned to three main groups based on disease categories. The articles on all types of skin conditions, without any major exclusions, were pooled together in the group “all skin conditions”. Articles on all types of skin malignancies, including actinic keratosis, squamous cell carcinoma, basal cell carcinoma, and melanoma, were pooled together in the “skin cancer” group. Articles on benign and malignant melanocytic lesions, including nevus, lentigo maligna, and melanoma, formed the group “pigmented lesions”. Within these disease categories, subgroup analyses were conducted based on communication platform, use of dermoscopic images, consultation type, tool used to acquire images, training received for image acquisition, and comparator.

#### 3.3.1 Primary outcomes

All results presented refer to overall results, i.e., undiagnosed cases were included in the analysis. For results calculated excluding undiagnosed cases, see Supplementary Figures S1–S42.

##### 3.3.1.1 Diagnostic concordance, Cohen’s kappa

**Figure 2:**
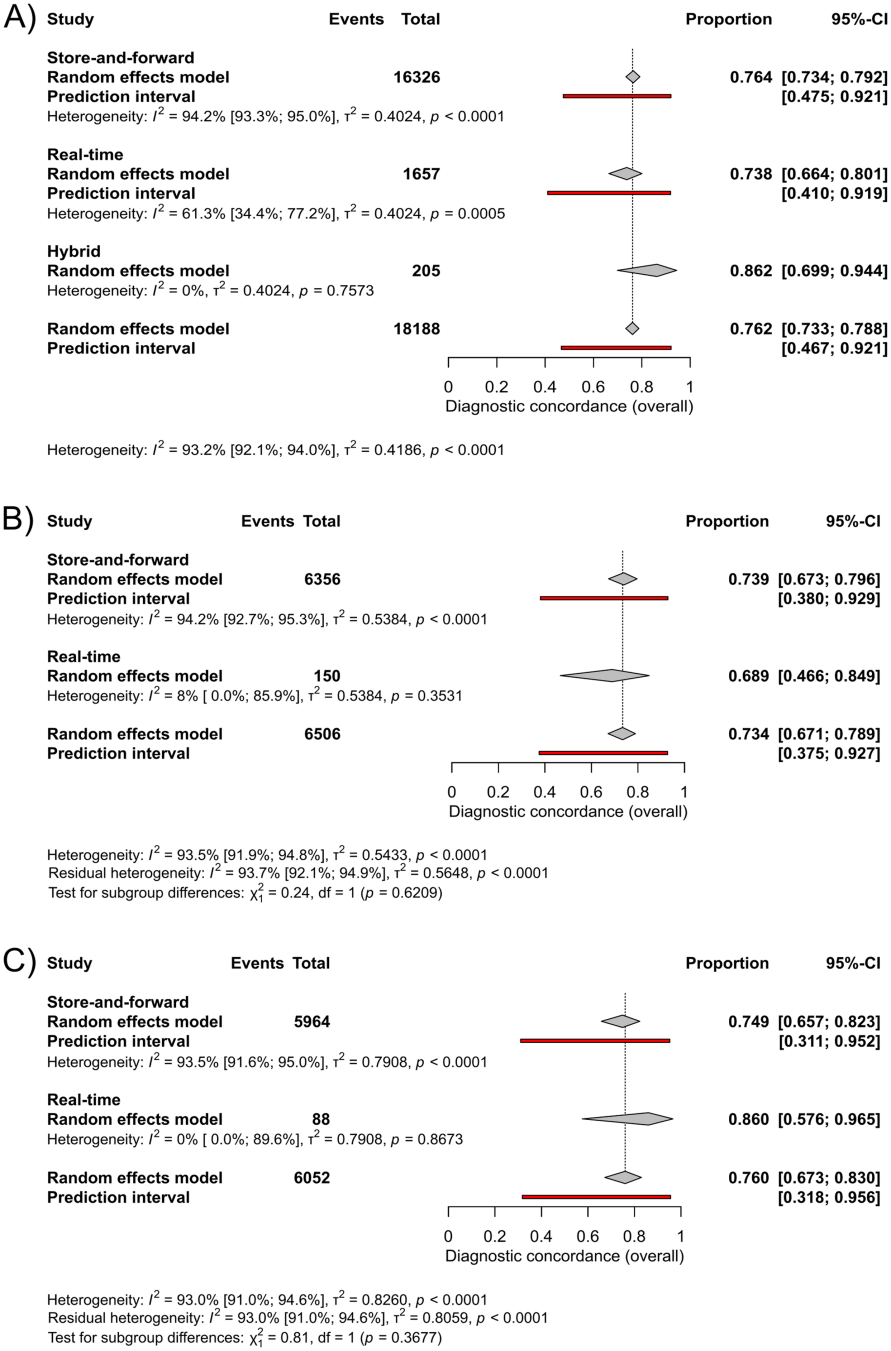
Forest plots for subgrouping based on communication platform: **(A)** Forest plot comparing diagnostic concordance between teledermatological and in-person dermatological diagnosis in the group “all skin conditions”, **(B)** Forest plot comparing diagnostic concordance between teledermatological and in-person dermatological diagnosis in the group “skin cancer”, **(C)** Forest plot comparing diagnostic concordance between teledermatological and in-person dermatological diagnosis in the group “pigmented lesions”.

In the group “all skin conditions”, subgroup analysis by communication platform showed a pooled diagnostic concordance of 76% (95%-CI: 73–79%; I^2^ = 94%) for store-and-forward (*n* = 16,326), and 73% (95%-CI: 66–80%; I^2^ = 61%) for the real-time method (*n* = 1,657) (Figure 2A). In the group “skin cancer”, pooled diagnostic concordance was found to be 74% (95%-CI: 67–79.6%; I2 = 94%) for store-and-forward (*n* = 6,356), and 69% (95%-CI: 47–85%; I^2^ = 8%) for real-time method (*n* = 150) (Figure 2B). For “pigmented lesions” the store-and-forward method (*n* = 5,964) showed a pooled diagnostic concordance of 75% (95%-CI, 66–82%; I^2^ = 94%), whereas for the real-time method (*n* = 88), the pooled diagnostic concordance was 86% (95%-CI, 58–97%; I^2^ = 0%) (Figure 2C). There was no statistically significant difference between communication platforms in any disease category. Data on the hybrid method were not sufficient for pooling. Forest plots displaying individual studies can be seen in Supplementary Figures S43–S45.

**Figure 3:**
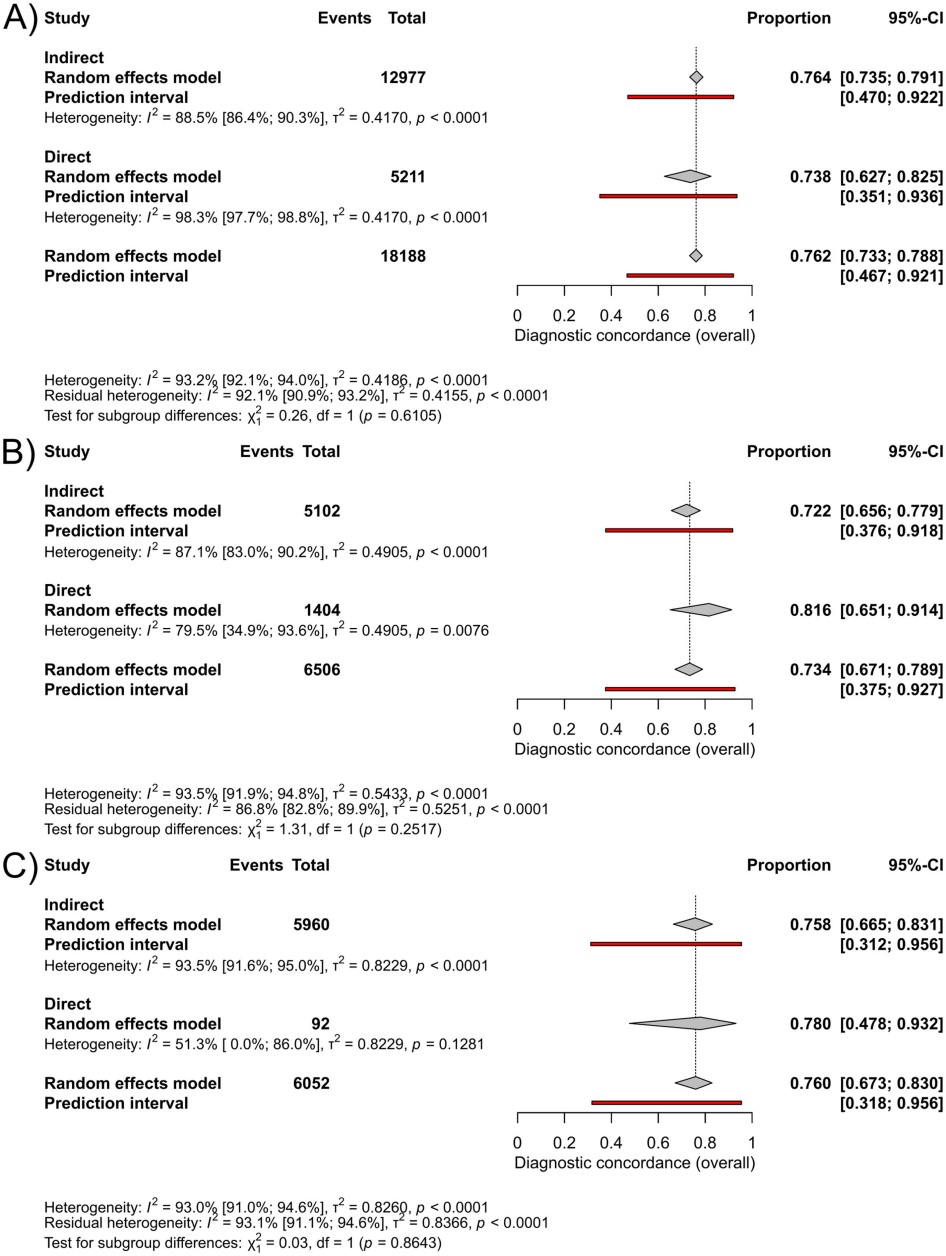
Forest plots for subgrouping based on communication type [direct/indirect]: **(A)** Forest plot comparing diagnostic concordance between teledermatological and in-person dermatological diagnosis in the group “all skin conditions”, **(B)** Forest plot comparing diagnostic concordance between teledermatological and in-person dermatological diagnosis in the group “skin cancer” group, **(C)** Forest plot comparing the diagnostic concordance between teledermatological and in-person dermatological diagnosis in the group “pigmented lesions”.

Subgroup analyses based on communication type showed a pooled diagnostic concordance of 76% (95%-CI: 63–83%; I^2^ = 98%), for direct (*n* = 5,211), and 76% (95%-CI: 74–79%; I^2^ = 89%), for indirect (*n* = 12,977) types in the group “all skin conditions” (Figure 3A). In the group “skin cancer,” pooled diagnostic concordance was found to be 82% (95%-CI: 65–91%; I^2^ = 80%), for direct (*n* = 1,404), compared to 72% (95%-CI: 66–78%; I^2^ = 87%), for indirect (*n* = 5,102) type (Figure 3B). The pooled diagnostic concordance in the group “pigmented lesions” was 78% (95%-CI: 48–93%; I^2^ = 51%), for the direct (*n* = 92), and 76% (95%-CI: 67–83%; I^2^ = 93%), for the indirect (*n* = 5,960) method (Figure 3C). No statistically significant difference was found in any disease category. Forest plots with individual studies listed can be seen in Supplementary Figures S46–S48.

**Figure 4:**
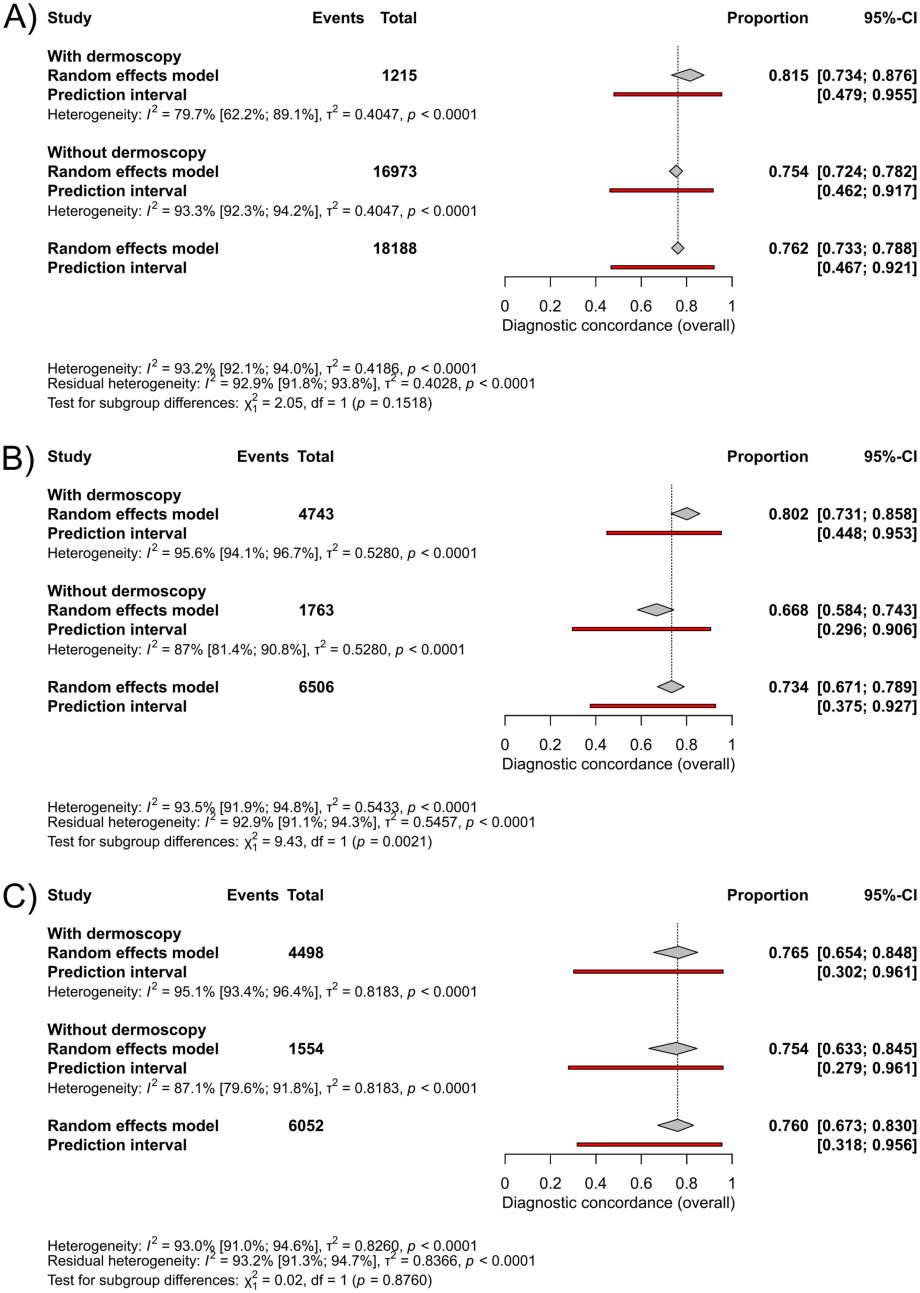
Forest plots for subgrouping based on the use of dermoscopy: **(A)** Forest plot comparing the diagnostic concordance between teledermatological and in-person dermatological diagnosis in the group “all skin conditions”, **(B)** Forest plot comparing diagnostic concordance between teledermatological and in-person dermatological diagnosis in the group “skin cancer”, **(C)** Forest plot comparing diagnostic concordance between teledermatological and in-person dermatological diagnosis in the group “pigmented lesions”.

Subgroup analyses based on the use of dermoscopy in the group “all skin conditions” showed a pooled diagnostic concordance of 82% (95%-CI: 73–88%; I^2^ = 80%) with dermoscopy (*n* = 1,215), compared to 75% (95%-CI: 72–78%; I^2^ = 93%) without dermoscopy (*n* = 16,973), the difference was not statistically significant (Figure 4A). In the group “skin cancer,” the use of dermoscopy (*n* = 4,743) yielded a significantly higher concordance of 80% (95%-CI: 73–86%; I^2^ = 96%), compared to 67% (95%-CI: 58–74%; I^2^ = 87%) without dermoscopy (*n* = 1,763) (Figure 4B). In the group “pigmented lesions” the use of dermoscopy (*n* = 4,498) resulted in a pooled diagnostic concordance of 77% (95%-CI: 65–85%; I^2^ = 95%), compared to 75% (95%-CI: 63–85%; I^2^ = 87%) in cases assessed without dermoscopy (*n* = 1,554), a difference that was not statistically significant (Figure 4C). For forest plots showing individual studies, see Supplementary Figures S49–S52.

No statistically significant subgroup differences were identified based on the photography device (Supplementary Figures S52-S54), or the training received for the image acquisition in any patient population (Supplementary Figures S55–S57). Subgroup differences based on comparator yielded inconclusive results (Supplementary Figures S58–S60).

Results for kappa concordance are presented in Supplementary Figures S61–S79.

##### 3.3.1.2 Sensitivity, specificity

Sensitivity and specificity of teledermatology were found to be 94% (95%-CI: 87–100%; I^2^ = 92%) and 82% (95%-CI: 65–99%; I^2^ = 98%) in the group “skin cancer,” respectively. In the group “pigmented lesions,” sensitivity and specificity were found to be 87% (95%-CI: 80–93%; I^2^ = 0%) and 84% (95%-CI: 74–95%; I^2^ = 87%), respectively (Supplementary Figures S80–S83).

#### 3.3.2 Secondary outcomes

**Figure 5:**
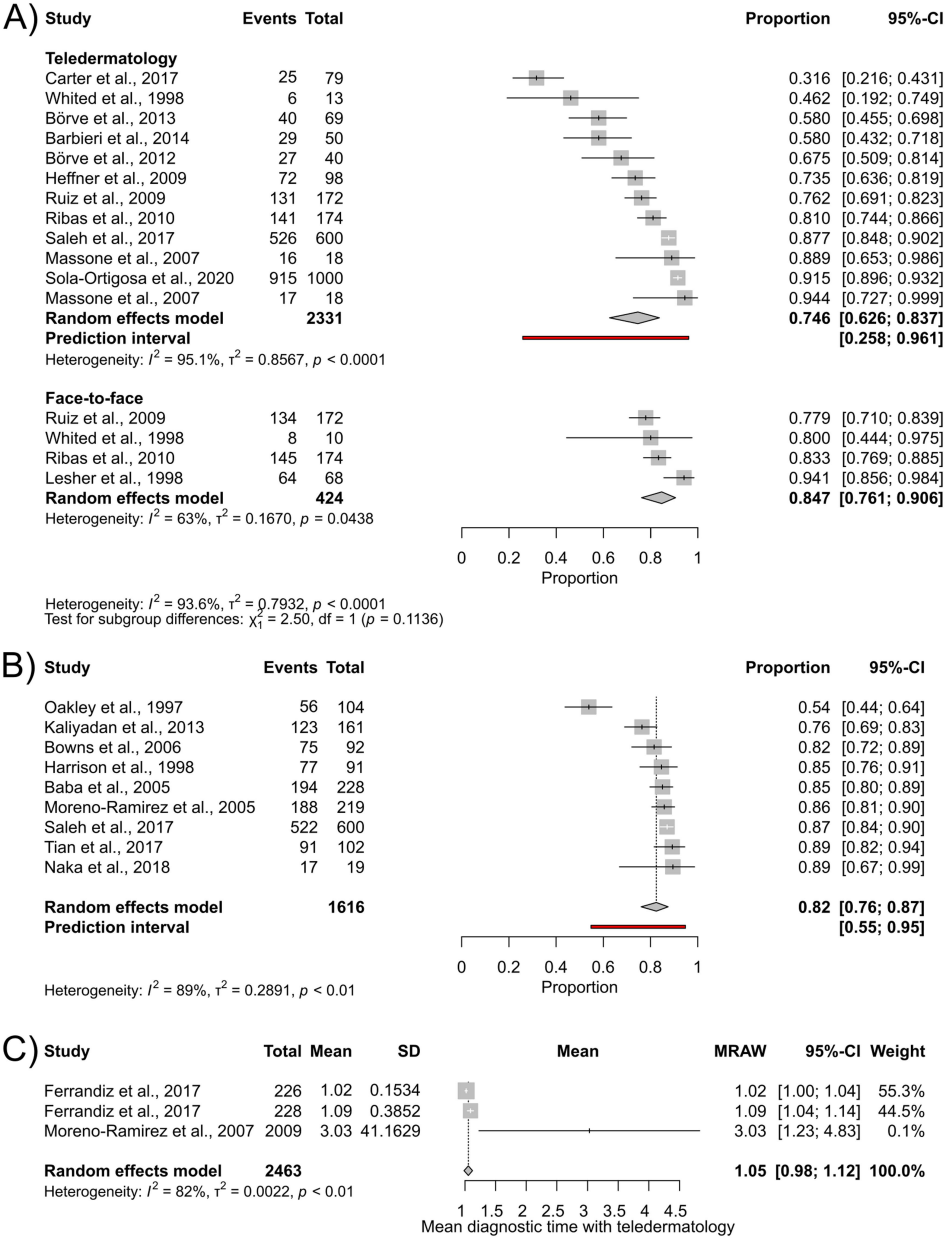
Forest plots for secondary outcomes: **(A)** Forest plot for comparing interrater agreement between teledermatological and in-person dermatological diagnosis; **(B)** forest plot for patient satisfaction; **(C)** forest plot for mean diagnostic time with teledermatology.

Secondary outcomes were assessed by pooling data across all disease categories. Interrater agreement was 75% (95%-CI: 63–84%; I^2^ = 95%) for teledermatology evaluations (*n* = 2,331), and 85% (95%-CI: 76–91%; I^2^ = 63%) for face-to-face assessments (*n* = 424), with no statistically significant difference between the groups (Figure 5A). Results for kappa concordance are presented in Supplementary Figure S83.

The pooled proportion of patients satisfied with teledermatological care was 82% (95%-CI: 76–87%; I^2^ = 89%) (*n* = 1,616)(Figure 5B). There were insufficient data to perform a statistical analysis of teledermatology provider satisfaction (Supplementary Table S3).

The mean diagnostic time during teledermatology consultations was 1.05 min (95%-CI: 0.98–1.12; I^2^ = 82.2%) (*n* = 2,463) (Figure 5C); data were not sufficient on the duration of face-to-face consultations to perform statistical analysis (Supplementary Table S4).

### 3.4 Qualitative analysis

For results on only qualitatively assessed studies and outcomes not possible to pool, see Supplementary Tables S3-S9.

### 3.5 Risk of bias assessment and publication bias

Most studies demonstrated moderate or serious risk of bias in certain domains, possibly due to their observational design (Supplementary Table S10; Supplementary Figure S85). Funnel plots of the main analyses evaluating publication bias are shown in Supplementary Figures S86–S96.

## 4 Discussion

The findings of our systematic review and meta-analysis provide the most comprehensive overview to date, with 155 studies comparing the diagnostic concordance of teledermatology and in-person dermatological examination in the diagnosis of skin diseases.

Our findings support that teledermatology provides diagnostic accuracy comparable to in-person care, with high concordance across all disease categories. This aligns with previous analyses by Kanthraj et al. (176) and Bourkas et al. (177), which also reported strong agreement between teledermatology and face-to-face diagnoses. In contrast, Bastola et al. (178) found teledermatology to be less reliable than in-person assessment, likely due to the small number of included studies and strict inclusion criteria. Our study applied a broader inclusion criteria; however, the analysis was restricted to studies in which diagnoses were established by dermatologists, thereby providing a clinically more relevant assessment (177). Moreover, our study uniquely highlights that the direct store-and-forward method, beside requiring fewer resources, achieves diagnostic performance on par with more complex modalities, indicating scalability across varying resource levels.

Our analyses found no significant differences in diagnostic concordance based on the communication platforms and types, regardless of disease category. This suggests that direct store-and-forward, the most convenient approach, is as effective as more complex and resource-heavy methods. Store-and-forward was the most commonly used platform in the included studies, likely due to its cost-effectiveness and minimal resource requirements (10). Previous studies have already urged its implementation in daily practice in resource-poor areas (176). The indirect method was used by most of the included studies, suggesting the low number and inaccessibility of direct teledermatology platforms. Our results suggest that the direct, patient-initiated method is reliable, and feasible for broad implementation.

Additional dermoscopic images did not significantly enhance diagnostic concordance in the group “pigmented lesions,” which involved only nevus and melanoma, suggesting its added importance in diagnosing non-melanoma skin cancers. A prior meta-analysis by Bourkas et al., which combined different types of skin lesions into a single analysis, suggested the limited additional value of dermoscopic images (177). In contrast, the meta-analysis of Chen et al. reported a substantially larger improvement in diagnostic accuracy for melanocytic lesions when dermoscopy was utilized in addition to clinical examination. This discrepancy, however, is likely attributable to methodological and contextual differences, as their study evaluated in-person, as well as remote clinical and dermoscopic examinations, whereas our present study focused solely on remote diagnostic agreement. They found that the diagnostic benefit of dermoscopy was more pronounced in purely in-person examinations and reduced when remote image assessment was included. These results suggest that the diagnostic gain of dermoscopy is attenuated in remote settings, consistent with our observations (179).

Although our results showed no significant benefit in the groups “all skin conditions” and “pigmented lesions,” we found that dermoscopy improves diagnostic accuracy for skin cancers. Our results, further supported by high sensitivity and specificity values, demonstrate that teledermatology has great potential as a screening tool for malignant skin diseases, including melanoma. As melanoma has the highest mortality among skin cancers, particularly when diagnosis is delayed, early detection is crucial to promote public health, reduce economic costs of health care, and improve patient outcomes (180, 181). However, analyses stratified by individual non-melanoma skin cancer subtypes were precluded by the lack of consistently reported, subtype-specific diagnostic outcomes in the included studies.

Further strengthening the easy applicability of teledermatology, we found no statistically significant difference based on the photography device and the training received for image acquisition. Contrary to previous findings, our results showed that images taken by patients with smartphones, without previous training for image acquisition, are not associated with lower diagnostic concordance, possibly due to the larger number of studies included in our analysis (177, 178, 182). This reinforces the feasibility of implementing the direct method more easily in daily practice.

Time efficiency is crucial to the widespread use of teledermatology. In our analysis, the mean diagnostic time was 1.05 min per case, which can be explained by the fact that all studies included in the analysis used the store-and-forward method, which is recognized in the literature as the fastest method (11). Although a direct comparison with face-to-face visits was not possible, prior studies suggest that traditional face-to-face visits can last up to 15–25 min (183), implying that significantly more patients can be managed through teledermatology in the same time frame. Patient satisfaction was also high, with 82% of patients expressing satisfaction with their teledermatology experience, suggesting that real-time interaction is not essential, since most included studies used the store-and-forward method.

The high concordance rates observed across all disease categories support the broad applicability of teledermatology for a wide range of skin conditions. Notably, our results indicate that teledermatological care remains reliable even without the use of dermoscopy, both for general skin conditions and for melanoma screening. This may be especially relevant in low-resource settings, where access to dermoscopic equipment and training may be limited (184). Given its efficiency, diagnostic accuracy, and high user satisfaction, teledermatology, particularly the direct store-and-forward approach, offers a scalable solution that can be effectively integrated into routine clinical workflows, however further tailoring for low-income settings should be implemented. These findings, in line with the WHO’s call to improve primary care for skin conditions, underscore the potential of teledermatology to alleviate the burden on dermatology services by enabling timely access to specialist care without compromising diagnostic reliability (6).

### 4.1 Strengths and limitations

Our study has multiple strengths. By rigorously following our pre-registered protocol and adhering to the guidelines of the Cochrane Collaboration, we ensured high standards of quality, transparency, and replicability. To our knowledge, this study represents the most comprehensive meta-analysis to date, incorporating diverse methodologies, disease categories, and imaging technologies.

This meta-analysis was based on a large, international dataset comprising studies from 33 countries, the majority of which were conducted in middle- and high-income countries. However, the underrepresentation of low-income countries may restrict the generalizability of our results to lower-resource settings.

A key limitation is pooling different skin cancer types into a single analytical category, as subtype-specyfic analyses were constrained by the existing evidence base, as most studies reported outcomes under broad categories, without providing stratified data by individual skin cancer subtype.

Substantial heterogeneity was also observed across analyses. Despite extensive subgroup analyses, heterogeneity remained high, likely reflecting diverse study settings, and predominantly observational designs.

Additional limitations include the presence of moderate and high risk of bias in several studies, evidence of publication bias, and the inability to pool predictive values due to the inconsistent disease prevalence reporting.

### 4.2 Implications for practice

We emphasize that improving access to dermatological care requires translating scientific findings into practice. We therefore support the integration of teledermatology into daily clinical workflows (185, 186). Our findings underscore the importance for policy engagement and strategic investment in telehealth infrastructure to facilitate its broader implementation.

### 4.3 Implications for research

To facilitate appropriate adoption across heterogenous resource settings, future studies specifically designed to low-income countries are required, focusing on technical feasibility, integration within existing health care infrastructures, diagnostic performance when implemented by non-specialist personnel, long-term sustainability in settings with limited specialist availability, and patient-centered outcomes. To further elucidate the reliability of teledermatology, future studies should focus on reporting outcomes separately for individual disease subtypes, especially for non-melanoma skin cancers, as well as on standardized outcome reporting, in particular true positives, true negatives, false positives, and false negatives, which are needed to determine sensitivity and specificity. Conducting future research on technology-enhancing modalities, such as complementary imaging techniques and artificial intelligence, is necessary to further improve the diagnostic concordance in more complex cases.

## 5 Conclusion

In conclusion, our study suggests that teledermatology is an effective tool for the remote diagnosis of a wide range of skin conditions. The high efficiency of the direct store-and-forward method, as the most easily applicable approach, indicates potential suitability in resource-limited settings, however, further research is needed to ensure its adaptability in low-resource environments.

## Data Availability

The datasets presented in this article are not readily available because the data sets used for the present study can be accessed in the full-text articles included in the systematic review and meta-analysis. Requests to access the datasets should be directed to banvolgyi.andras@semmelweis.hu.
