# Supplementary material for: Diagnostic accuracy of teledermatology for skin diseases: a systematic review and meta-analysis
Source: Front Med (Lausanne). 2026 Mar 2;13:1739592. doi: 10.3389/fmed.2026.1739592 (PMC12989395; doi:10.3389/fmed.2026.1739592)
Supplement: Supplementary file 1 [file Data_Sheet_1.docx]

***Supplementary material***

**Diagnostic accuracy of teledermatology for skin diseases: a systematic review and meta-analysis**

**Authors**

Katalin Martyin^1,2^, Fanni Adél Meznerics^1,2^, Laura Anna Bokor^1,2^, Bence Szabó^2^, Péter Hegyi^2,3,4^, Norbert Kiss^1,2*^, András Bánvölgyi^1,2*^

**Affiliations**

*^1^ Department of Dermatology, Venereology and Dermatooncology, Faculty of Medicine, Semmelweis University, 41 Mária Street, 1084 Budapest, Hungary*

*^2^ Centre for Translational Medicine, Semmelweis University, 22 Baross Street, 1085 Budapest, Hungary*

*^3^ Institute of Pancreatic Diseases, Semmelweis University, 25-29 Tömő Street, 1083 Budapest, Hungary*

*^4^ Institute for Translational Medicine, Medical School, University of Pécs, Szigeti Road 12, 7624 Pécs, Hungary*

*^*^* These authors share last authorship.

**Correspondence to**

András Bánvölgyi MD, PhD

*Department of Dermatology, Venereology and Dermatooncology, Faculty of Medicine, Semmelweis University, 41 Mária Street, 1084 Budapest, Hungary*

Email: [*banvolgyi.andras@semmelweis.hu*](mailto:banvolgyi.andras@semmelweis.hu)

**Contents**

[Search strategy 9](#_Toc199465505)

[Supplementary Table S1: Number of hits in the electronic databases. 9](#_Toc199465506)

[Search key for systematic search used in each electronic database: 9](#_Toc199465507)

[Supplementary Table S2: Additional study and patient characteristics. 11](#_Toc199465508)

[Supplementary Figure S1: Forest plot comparing the diagnostic concordance between teledermatology providers and in-person dermatologists subgrouped by the communication platform in the “all skin conditions” group, excluding undiagnosed cases from the analysis. 25](#_Toc199465509)

[Supplementary Figure S2: Forest plot comparing the diagnostic concordance between teledermatology providers and in-person dermatologists subgrouped by the communication platform in the “skin cancer” group, excluding undiagnosed cases from the analysis. 26](#_Toc199465510)

[Supplementary Figure S3: Forest plot comparing the diagnostic concordance between teledermatology providers and in-person dermatologists subgrouped by the communication platform in the “pigmented lesions” group, excluding undiagnosed cases from the analysis. 27](#_Toc199465511)

[Supplementary Figure S4: Forest plot comparing the diagnostic concordance between teledermatology providers and in-person dermatologists subgrouped by the communication type in the “all skin conditions” group, excluding undiagnosed cases from the analysis. 28](#_Toc199465512)

[Supplementary Figure S5: Forest plot comparing the diagnostic concordance between teledermatology providers and in-person dermatologists subgrouped by the communication type in the “skin cancer” group, excluding undiagnosed cases from the analysis. 29](#_Toc199465513)

[Supplementary Figure S6: Forest plot comparing the diagnostic concordance between teledermatology providers and in-person dermatologists subgrouped by the communication type in the “pigmented lesions group”, excluding undiagnosed cases from the analysis. 30](#_Toc199465514)

[Supplementary Figure S7: Forest plot comparing the diagnostic concordance between teledermatology providers and in-person dermatologists subgrouped by the use of dermoscopy in the “all skin conditions” group, excluding undiagnosed cases from the analysis. 31](#_Toc199465515)

[Supplementary Figure S8: Forest plot comparing the diagnostic concordance between teledermatology providers and in-person dermatologists subgrouped by the use of dermoscopy in the “skin cancer” group, excluding undiagnosed cases from the analysis. 32](#_Toc199465516)

[Supplementary Figure S9: Forest plot comparing the diagnostic concordance between teledermatology providers and in-person dermatologists subgrouped by the use of dermoscopy in the “pigmented lesions” group, excluding undiagnosed cases from the analysis. 33](#_Toc199465517)

[Supplementary Figure S10: Forest plot comparing the diagnostic concordance between teledermatology providers and in-person dermatologists subgrouped by photography device in the “all skin conditions” group, excluding undiagnosed cases from the analysis. 34](#_Toc199465518)

[Supplementary Figure S11: Forest plot comparing the diagnostic concordance between teledermatology providers and in-person dermatologists subgrouped by photography device in the “skin cancer” group, excluding undiagnosed cases from the analysis. 35](#_Toc199465519)

[Supplementary Figure S12: Forest plot comparing the diagnostic concordance between teledermatology providers and in-person dermatologists subgrouped by photography device in the “pigmented lesions” group, excluding undiagnosed cases from the analysis. 36](#_Toc199465520)

[Supplementary Figure S13: Forest plot comparing the diagnostic concordance between teledermatology providers and in-person dermatologists subgrouped by training for image acquisition in the “all skin conditions” group, excluding undiagnosed cases from the analysis. 37](#_Toc199465521)

[Supplementary Figure S14: Forest plot comparing the diagnostic concordance between teledermatology providers and in-person dermatologists subgrouped by training for image acquisition in the “skin cancer” group, excluding undiagnosed cases from the analysis. 38](#_Toc199465522)

[Supplementary Figure S15: Forest plot comparing the diagnostic concordance between teledermatology providers and in-person dermatologists subgrouped by training for image acquisition in the “pigmented lesions” group, excluding undiagnosed cases from the analysis. 39](#_Toc199465523)

[Supplementary Figure S16: Forest plot comparing the diagnostic concordance between teledermatology providers and in-person dermatologists subgrouped by the comparator in the “all skin conditions” group, excluding undiagnosed cases from the analysis. 40](#_Toc199465524)

[Supplementary Figure S17: Forest plot comparing the diagnostic concordance between teledermatology providers and in-person dermatologists subgrouped by the comparator in the “skin cancer” group, excluding undiagnosed cases from the analysis. 41](#_Toc199465525)

[Supplementary Figure S18: Forest plot comparing the diagnostic concordance between teledermatology providers and in-person dermatologists subgrouped by the comparator in the “pigmented lesions” group, excluding undiagnosed cases from the analysis. 42](#_Toc199465526)

[Supplementary Figure S19: Forest plot comparing Cohen’s kappa between teledermatology providers and in-person dermatologists subgrouped by the communication platform in the “all skin conditions” group, excluding undiagnosed cases from the analysis. 43](#_Toc199465527)

[Supplementary Figure S20: Forest plot comparing Cohen’s kappa between teledermatology providers and in-person dermatologists subgrouped by the communication platform in the “skin cancer” group, excluding undiagnosed cases from the analysis. 43](#_Toc199465528)

[Supplementary Figure S21: Forest plot comparing Cohen’s kappa between teledermatology providers and in-person dermatologists subgrouped by the communication platform in the “pigmented lesions” group, excluding undiagnosed cases from the analysis. 44](#_Toc199465529)

[Supplementary Figure S22: Forest plot comparing Cohen’s kappa between teledermatology providers and in-person dermatologists subgrouped by the communication type in the “all skin conditions” group, excluding undiagnosed cases from the analysis. 44](#_Toc199465530)

[Supplementary Figure S23: Forest plot comparing Cohen’s kappa between teledermatology providers and in-person dermatologists subgrouped by the communication type in the “skin cancer” group, excluding undiagnosed cases from the analysis. 45](#_Toc199465531)

[Supplementary Figure S24: Forest plot comparing Cohen’s kappa between teledermatology providers and in-person dermatologists subgrouped by the communication type in the “pigmented lesions” group, excluding undiagnosed cases from the analysis. 45](#_Toc199465532)

[Supplementary Figure S25: Forest plot comparing Cohen’s kappa between teledermatology providers and in-person dermatologists subgrouped by the use of dermoscopy in the “all skin conditions” group, excluding undiagnosed cases from the analysis. 46](#_Toc199465533)

[Supplementary Figure S26: Forest plot comparing Cohen’s kappa between teledermatology providers and in-person dermatologists subgrouped by the use of dermoscopy in the “skin cancer” group, excluding undiagnosed cases from the analysis. 47](#_Toc199465534)

[Supplementary Figure S27: Forest plot comparing Cohen’s kappa between teledermatology providers and in-person dermatologists subgrouped by the use of dermoscopy in the “pigmented lesions” group, excluding undiagnosed cases from the analysis. 47](#_Toc199465535)

[Supplementary Figure S28: Forest plot comparing Cohen’s kappa between teledermatology providers and in-person dermatologists subgrouped by the photography device in the “all skin conditions” group, excluding undiagnosed cases from the analysis. 48](#_Toc199465536)

[Supplementary Figure S29: Forest plot comparing Cohen’s kappa between teledermatology providers and in-person dermatologists subgrouped by the photography device in the “skin cancer” group, excluding undiagnosed cases from the analysis. 48](#_Toc199465537)

[Supplementary Figure S30: Forest plot comparing the Cohen’s kappa between teledermatology providers and in-person dermatologists subgrouped by the photography device in the “pigmented lesions” group, excluding undiagnosed cases from the analysis. 49](#_Toc199465538)

[Supplementary Figure S31: Forest plot comparing Cohen’s kappa between teledermatology providers and in-person dermatologists subgrouped by training for image acquisition in the “all skin conditions” group, excluding undiagnosed cases from the analysis. 49](#_Toc199465539)

[Supplementary Figure S32: Forest plot comparing Cohen’s kappa between teledermatology providers and in-person dermatologists subgrouped by training for image acquisition in the “skin cancer” group, excluding undiagnosed cases from the analysis. 50](#_Toc199465540)

[Supplementary Figure S33: Forest plot comparing Cohen’s kappa between teledermatology providers and in-person dermatologists subgrouped by training for image acquisition in the “pigmented lesions” group, excluding undiagnosed cases from the analysis. 50](#_Toc199465541)

[Supplementary Figure S34: Forest plot comparing Cohen’s kappa between teledermatology providers and in-person dermatologists subgrouped by the comparator in the “all skin conditions” group, excluding undiagnosed cases from the analysis. 51](#_Toc199465542)

[Supplementary Figure 35: Forest plot comparing Cohen’s kappa in the pairwise comparison of “face-to-face ±histopathology” and “face-to-face” comparators in the “all skin conditions” group, excluding undiagnosed cases in the analysis. 52](#_Toc199465543)

[Supplementary Figure S36: Forest plot comparing Cohen’s kappa between teledermatology providers and in-person dermatologists subgrouped by the comparator in the “skin cancer” group, excluding undiagnosed cases from the analysis. 52](#_Toc199465544)

[Supplementary Figure S37: Forest plot comparing Cohen’s kappa between teledermatology providers and in-person dermatologists subgrouped by the comparator in the “pigmented lesions” group, excluding undiagnosed cases from the analysis. 53](#_Toc199465545)

[Supplementary Figure S38: Forest plot comparing Cohen’s kappa in the pairwise comparison of “face-to-face” and “histopathology” comparators in the “pigmented lesions” group, excluding undiagnosed cases in the analysis. 53](#_Toc199465546)

[Supplementary Figure S39: Forest plot for the sensitivity of teledermatology in the “skin cancer” group, excluding undiagnosed cases from the analysis. 54](#_Toc199465547)

[Supplementary Figure S40: Forest plot for the specificity of teledermatology in the “skin cancer” group, excluding undiagnosed cases from the analysis. 54](#_Toc199465548)

[Supplementary Figure S41: Forest plot for the sensitivity of teledermatology in the “pigmented lesions” group, excluding undiagnosed cases from the analysis. 54](#_Toc199465549)

[Supplementary Figure S42: Forest plot for the specificity of teledermatology in the “pigmented lesions” group, excluding undiagnosed cases from the analysis. 54](#_Toc199465550)

[Supplementary Figure S43: Forest plot comparing the diagnostic concordance between teledermatology providers and in-person dermatologists subgrouped by the communication platform in the “all skin conditions” group, including undiagnosed cases in the analysis. 55](#_Toc199465551)

[Supplementary Figure S44: Forest plot comparing the diagnostic concordance between teledermatology providers and in-person dermatologists subgrouped by the communication platform in the “skin cancer” group, including undiagnosed cases in the analysis. 56](#_Toc199465552)

[Supplementary Figure S45: Forest plot comparing the diagnostic concordance between teledermatology providers and in-person dermatologists subgrouped by the communication platform in the “pigmented lesions” group, including undiagnosed cases in the analysis. 57](#_Toc199465553)

[Supplementary Figure S46: Forest plot comparing the diagnostic concordance between teledermatology providers and in-person dermatologists subgrouped by the communication type in the “all skin conditions” group, including undiagnosed cases in the analysis. 58](#_Toc199465554)

[Supplementary Figure S47: Forest plot comparing the diagnostic concordance between teledermatology providers and in-person dermatologists subgrouped by the communication type in the “skin cancer” group, including undiagnosed cases in the analysis. 59](#_Toc199465555)

[Supplementary Figure S48: Forest plot comparing the diagnostic concordance between teledermatology providers and in-person dermatologists subgrouped by the communication type in the “pigmented lesions” group, including undiagnosed cases in the analysis. 60](#_Toc199465556)

[Supplementary Figure S49: Forest plot comparing the diagnostic concordance between teledermatology providers and in-person dermatologists subgrouped by the use of dermoscopy in the “all skin conditions” group, including undiagnosed cases in the analysis. 61](#_Toc199465557)

[Supplementary Figure S50: Forest plot comparing the diagnostic concordance between teledermatology providers and in-person dermatologists subgrouped by the use of dermoscopy in the “skin cancer” group, including undiagnosed cases in the analysis. 62](#_Toc199465558)

[Supplementary Figure S51: Forest plot comparing the diagnostic concordance between teledermatology providers and in-person dermatologists subgrouped by the use of dermoscopy in the “pigmented lesions” group, including undiagnosed cases in the analysis. 63](#_Toc199465559)

[Supplementary Figure S52: Forest plot comparing the diagnostic concordance between teledermatology providers and in-person dermatologists subgrouped by the photography device in the “all skin conditions” group, including undiagnosed cases in the analysis. 64](#_Toc199465560)

[Supplementary Figure S53: Forest plot comparing the diagnostic concordance between teledermatology providers and in-person dermatologists subgrouped by the photography device in the “skin cancer group”, including undiagnosed cases in the analysis. 65](#_Toc199465561)

[Supplementary Figure S54: Forest plot comparing the diagnostic concordance between teledermatology providers and in-person dermatologists subgrouped by the photography device in the “pigmented lesions” group, including undiagnosed cases in the analysis. 66](#_Toc199465562)

[Supplementary Figure S55: Forest plot comparing the diagnostic concordance between teledermatology providers and in-person dermatologists subgrouped by training for image acquisition in the “all skin conditions” group, including undiagnosed cases in the analysis. 67](#_Toc199465563)

[Supplementary Figure S56: Forest plot comparing the diagnostic concordance between teledermatology providers and in-person dermatologists subgrouped by training for image acquisition in the “skin cancer” group, including undiagnosed cases in the analysis. 68](#_Toc199465564)

[Supplementary Figure S57: Forest plot comparing the diagnostic concordance between teledermatology providers and in-person dermatologists subgrouped by training for image acquisition in the “pigmented lesions” group, including undiagnosed cases in the analysis. 68](#_Toc199465565)

[Supplementary Figure S58: Forest plot comparing the diagnostic concordance between teledermatology providers and in-person dermatologists subgrouped by the comparator in the “all skin conditions” group, including undiagnosed cases in the analysis. 69](#_Toc199465566)

[Supplementary Figure S59: Forest plot comparing the diagnostic concordance between teledermatology providers and in-person dermatologists subgrouped by the comparator in the “skin cancer” group, including undiagnosed cases in the analysis. 70](#_Toc199465567)

[Supplementary Figure S60: Forest plot comparing the diagnostic concordance between teledermatology providers and in-person dermatologists subgrouped by the comparator in the “pigmented lesions” group, including undiagnosed cases in the analysis. 71](#_Toc199465568)

[Supplementary Figure S61: Forest plot comparing Cohen’s kappa between teledermatology providers and in-person dermatologists based on the communication platform in the ”all skin conditions” group, including undiagnosed cases in the analysis. 72](#_Toc199465569)

[Supplementary Figure S62: Forest plot comparing Cohen’s kappa between teledermatology providers and in-person dermatologists based on the communication platform in the “skin cancer” group, including undiagnosed cases in the analysis. 72](#_Toc199465570)

[Supplementary Figure S63: Forest plot comparing Cohen’s kappa between teledermatology providers and in-person dermatologists based on the communication platform in the “pigmented lesions” group. including undiagnosed cases in the analysis. 73](#_Toc199465571)

[Supplementary Figure S64: Forest plot comparing Cohen’s kappa between teledermatology providers and in-person dermatologists based on the communication type in the “all skin conditions” group, including undiagnosed cases in the analysis. 73](#_Toc199465572)

[Supplementary Figure S65: Forest plot comparing Cohen’s kappa between teledermatology providers and in-person dermatologists based on the communication type in the “skin cancer” group, including undiagnosed cases in the analysis. 74](#_Toc199465573)

[Supplementary Figure S66: Forest plot comparing Cohen’s kappa between teledermatology providers and in-person dermatologists based on the communication type in the “pigmented lesions” group, including undiagnosed cases in the analysis. 74](#_Toc199465574)

[Supplementary Figure S67: Forest plot comparing Cohen’s kappa between teledermatology providers and in-person dermatologists based on the use of dermoscopy in the “all skin conditions” group, including undiagnosed cases in the analysis. 75](#_Toc199465575)

[Supplementary Figure S68: Forest plot comparing Cohen’s kappa between teledermatology providers and in-person dermatologists based on the use of dermoscopy in the “skin cancer” group, including undiagnosed cases in the analysis. 75](#_Toc199465576)

[Supplementary Figure S69: Forest plot comparing Cohen’s kappa between teledermatology providers and in-person dermatologists based on the use of dermoscopy in the “pigmented lesions” group, including undiagnosed cases in the analysis. 76](#_Toc199465577)

[Supplementary Figure S70: Forest plot comparing Cohen’s kappa between teledermatology providers and in-person dermatologists based on the photography device in the “all skin conditions” group, including undiagnosed cases in the analysis. 77](#_Toc199465578)

[Supplementary Figure S71: Forest plot comparing Cohen’s kappa between teledermatology providers and in-person dermatologists based on the photography device in the “skin cancer” group, including undiagnosed cases in the analysis. 77](#_Toc199465579)

[Supplementary Figure S72: Forest plot comparing Cohen’s kappa between teledermatology providers and in-person dermatologists based on the photography device in the “pigmented lesions” group, including undiagnosed cases in the analysis. 78](#_Toc199465580)

[Supplementary Figure S73: Forest plot comparing Cohen’s kappa between teledermatology providers and in-person dermatologists based on training for image acquisition in the “all skin conditions” group, including undiagnosed cases in the analysis. 78](#_Toc199465581)

[Supplementary Figure S74: Forest plot comparing Cohen’s kappa between teledermatology providers and in-person dermatologists based on training for image acquisition in the “skin cancer” group, including undiagnosed cases in the analysis. 79](#_Toc199465582)

[Supplementary Figure S75: Forest plot comparing Cohen’s kappa between teledermatology providers and in-person dermatologists based on training for image acquisition in the “pigmented lesions” group, including undiagnosed cases in the analysis. 79](#_Toc199465583)

[Supplementary Figure S76: Forest plot comparing Cohen’s kappa between teledermatology providers and in-person dermatologists based on the comparator in the “all skin conditions” group, including undiagnosed cases in the analysis. 80](#_Toc199465584)

[Supplementary Figure S77: Forest plot comparing Cohen’s kappa in the pairwise comparison of “face-to-face ±histopathology” and “face-to-face” comparators in the “all skin conditions” group, including undiagnosed cases in the analysis. 81](#_Toc199465585)

[Supplementary Figure S78: Forest plot comparing Cohen’s kappa between teledermatology providers and in-person dermatologists based on the comparator in the “skin cancer” group, including undiagnosed cases in the analysis. 81](#_Toc199465586)

[Supplementary Figure S79: Forest plot comparing Cohen’s kappa between teledermatology providers and in-person dermatologists based on the comparator in the “pigmented lesions” group, including undiagnosed cases in the analysis. 82](#_Toc199465587)

[Supplementary Figure S80: Forest plot for the sensitivity of teledermatology in the “skin cancer” group, including undiagnosed cases in the analysis. 82](#_Toc199465588)

[Supplementary Figure S81: Forest plot for the specificity of teledermatology in the “skin cancer” group, including undiagnosed cases in the analysis. 82](#_Toc199465589)

[Supplementary Figure S82: Forest plot for the sensitivity of teledermatology in the “pigmented lesions” group, including undiagnosed cases in the analysis. 83](#_Toc199465590)

[Supplementary Figure S83: Forest plot for the specificity of teledermatology in the “pigmented lesions” group, including undiagnosed cases in the analysis. 83](#_Toc199465591)

[Supplementary Figure S84: Forest plot for the interrater agreement between teledermatologists and in-person dermatologists, measured subgrouped by Cohen’s kappa. 83](#_Toc199465592)

[Supplementary Table S3: Summary of results on satisfaction of teledermatology providers 84](#_Toc199465593)

[Supplementary Table S4: Summary of results on diagnostic time during face-to-face dermatology visits 84](#_Toc199465594)

[Supplementary Table S5: Summary of results on diagnostic concordance results excluded from the quantitative analyses 84](#_Toc199465595)

[Supplementary Table S6: Summary of results on Cohen’s kappa results excluded from the quantitative analyses 85](#_Toc199465596)

[Supplementary Table S7: Summary of results on the sensitivity and specificity of teledermatology excluded from the quantitative analyses 85](#_Toc199465597)

[Supplementary Table S8: Summary of results on positive and negative predictive values of teledermatology 86](#_Toc199465598)

[Supplementary Table S9: Summary of results on Area Under the Curve values of teledermatology 87](#_Toc199465599)

[Risk of bias (RoB) assessment 87](#_Toc199465600)

[Supplementary Table S10: Domain-level risk of bias assessments for all included articles 87](#_Toc199465601)

[Supplementary Figure S85: Distribution of risk of bias judgements within bias domains 91](#_Toc199465602)

[Publication bias 92](#_Toc199465603)

[Supplementary Figure S86: Funnel plot for studies in the “all skin conditions” group assessing diagnostic concordance, including undiagnosed cases in the analysis. 92](#_Toc199465604)

[Supplementary Figure S87: Funnel plot for studies in the “all skin conditions” group assessing diagnostic concordance, excluding undiagnosed cases from the analysis. 93](#_Toc199465605)

[Supplementary Figure S88: Funnel plot for studies in the “all skin conditions” group assessing Cohen’s kappa, including undiagnosed cases in the analysis. 94](#_Toc199465606)

[Supplementary Figure S89: Funnel plot for studies in the “all skin conditions” group assessing Cohen’s kappa, excluding undiagnosed cases from the analysis. 95](#_Toc199465607)

[Supplementary Figure S90: Funnel plot for studies in the “skin cancer” group assessing diagnostic concordance, including undiagnosed cases in the analysis. 96](#_Toc199465608)

[Supplementary Figure S91: Funnel plot for studies in the “skin cancer” group assessing diagnostic concordance, excluding undiagnosed cases from the analysis. 97](#_Toc199465609)

[Supplementary Figure S92: Funnel plot for studies in the “pigmented lesions” group assessing diagnostic concordance, including undiagnosed cases in the analysis. 98](#_Toc199465610)

[Supplementary Figure S93: Funnel plot for studies in the “pigmented lesions” group assessing diagnostic concordance, excluding undiagnosed cases from the analysis. 99](#_Toc199465611)

[Supplementary Figure S94: Funnel plot for studies in the “pigmented lesions” group assessing Cohen’s kappa, excluding undiagnosed cases from the analysis. 100](#_Toc199465612)

[Supplementary Figure S95: Funnel plot assessing diagnostic concordance for interrater agreement. 101](#_Toc199465613)

[Supplementary Figure S96: Funnel plot assessing Cohen’s kappa for interrater agreement. 102](#_Toc199465614)

[References 103](#_Toc199465615)

# **Search strategy**

## Supplementary Table S1: Number of hits in the electronic databases.

Supplementary Table S1: Number of hits in the electronic databases

| Database searched | via | Records |
| --- | --- | --- |
| MEDLINE | Pubmed | 15,001 |
| Embase | Embase.com | 12,822 |
| Cochrane Library | Cochranelibrary.com | 2,472 |
| Total |  | 30,412 |

## Search key for systematic search used in each electronic database:

MEDLINE (via PubMed):

telederm* OR (tele* AND derm*) **OR** ((telemedicin* OR ”tele medicin*” OR telehealth* OR ”tele health*” OR telerefer* OR ”tele refer*” OR teleconsult* OR ”tele consult*” OR televisit* OR ”tele visit*” OR telecare* OR ”tele care*” OR telepractic* OR ”tele practic*” OR telemonitor* OR ”tele monitor*” OR telecommunicat* OR ”tele communicat*” OR teleconferenc* OR ”tele conferenc*” OR emedicin* OR ”e medicin*” OR ”electronic medicin*” OR ehealth* OR ”e health*” OR ”electronic health*” OR erefer* OR ”e refer*” OR ”electronic refer*” OR econsult* OR ”e consult*” OR ”electronic consult*” OR evisit* OR ”e visit*” OR ”electronic visit*” OR ecare* OR ”e care*” OR ”electronic care*” OR epractic* OR ”e practic*” OR ”electronic practic*” OR emonitor* OR ”e monitor*” OR ”electronic monitor*” OR ecommunicat* OR ”e communicat*” OR ”electronic communicat*” OR econferenc* OR ”e conferenc*” OR ”electronic conferenc*” OR ”virtual medicin*” OR ”virtual health*” OR ”virtual refer*” OR ”virtual consult*” OR ”virtual visit*” OR ”virtual care*” OR ”virtual practic*” OR ”virtual monitor*” OR ”virtual communicat*” OR ”virtual conferenc*” OR "video medicin*" OR ”video refer*” OR videorefer* OR ”video consult*” OR videoconsult* OR ”video visit*” OR videovisit* OR ”video monitor*” OR videomonitor* OR ”video call*” OR videocall* OR ”video conferenc*” OR videoconferenc* OR ”video communicat*” OR ”video telephon*” OR videotelephon* OR remote* OR ”store and forward*” OR smartphon* OR cellphon* OR phone* OR telephon* OR mhealth*) **AND** (derm* OR skin* OR cutan*))

No special settings or filters were used.

Embase:

(telederm* OR (tele AND derm*) **OR** ((telemedicin* OR ”tele medicin*” OR telehealth* OR ”tele health*” OR telerefer* OR ”tele refer*” OR teleconsult* OR ”tele consult*” OR televisit* OR ”tele visit*” OR telecare* OR ”tele care*” OR telepractic* OR ”tele practic*” OR telemonitor* OR ”tele monitor*” OR telecommunicat* OR ”tele communicat*” OR teleconferenc* OR ”tele conferenc*” OR emedicin* OR ”e medicin*” OR ”electronic medicin*” OR ehealth* OR ”e health*” OR ”electronic health*” OR erefer* OR ”e refer*” OR ”electronic refer*” OR econsult* OR ”e consult*” OR ”electronic consult*” OR evisit* OR ”e visit*” OR ”electronic visit*” OR ecare* OR ”e care*” OR ”electronic care*” OR epractic* OR ”e practic*” OR ”electronic practic*” OR emonitor* OR ”e monitor*” OR ”electronic monitor*” OR ecommunicat* OR ”e communicat*” OR ”electronic communicat*” OR econferenc* OR ”e conferenc*” OR ”electronic conferenc*” OR ”virtual medicin*” OR ”virtual health*” OR ”virtual refer*” OR ”virtual consult*” OR ”virtual visit*” OR ”virtual care*” OR ”virtual practic*” OR ”virtual monitor*” OR ”virtual communicat*”

OR ”virtual conferenc*” OR "video medicin*" OR ”video refer*” OR videorefer* OR ”video consult*” OR videoconsult* OR ”video visit*” OR videovisit* OR ”video monitor*” OR videomonitor* OR ”video call*” OR videocall* OR ”video conferenc*” OR videoconferenc* OR ”video communicat*” OR ”video telephon*” OR videotelephon* OR remote* OR ”store and forward*” OR smartphon* OR cellphon* OR phone* OR telephon* OR mhealth*) AND (derm* OR skin* OR cutan*)))*:ab,kw,ti*

No special settings, filters or restrictions are used. The search is conducted in titles, abstracts and keywords. Advanced search was used, with every setting in mapping unchecked.

Cochrane Library (CENTRAL):

telederm* OR (tele AND derm*) **OR** ((telemedicin* OR tele-medicin* OR telehealth* OR tele-health* OR telerefer* OR tele-refer* OR teleconsult* OR tele-consult* OR televisit* OR tele-visit* OR telecare* OR tele-care* OR telepractic* OR tele-practic* OR telemonitor* OR tele-monitor* OR telecommunicat* OR tele-communicat* OR teleconferenc* OR tele- conferenc* OR emedicin* OR e-medicin* OR ”electronic medicine” OR ehealth* OR e- health* OR ”electronic health” OR erefer* OR e-referral* OR ”electronic referral” OR econsult* OR e-consult* OR ”electronic consultation” OR evisit* OR e-visit* OR ”electronic visit” OR ecare* OR e-care* OR ”electronic care” OR epractic* OR e-practic* OR ”electronic practice” OR emonitor* OR e-monitor* OR ”electronic monitoring” OR ecommunicat* OR e-communicat* OR ”electronic communication” OR econferenc* OR e- conferenc* OR ”electronic conference” OR ”electronic conferencing” OR ”virtual medicine” OR ”virtual health” OR ”virtual-referral” OR ”virtual consultation” OR ”virtual visit” OR ”virtual care” OR ”virtual practice” OR ”virtual monitoring” OR ”virtual communication” OR ”virtual conference” OR ”virtual conferencing” OR video-medicin* OR videorefer* OR video-refer* OR videoconsult* OR video-consult* OR videovisit* OR video-visit* OR videomonitor* OR video-monitor* OR videocall* OR video-call* OR videoconferenc* OR video-conferenc* OR video-communicat* OR video-telephon* OR videotelephon* OR remote* OR ”store and forward” OR smartphon* OR cellphon* OR phone* OR telephon* OR mhealth*) AND (derm* OR skin* OR cutan*))

No special settings, filters or restrictions are used. Advanced search was used (all fields).

# Supplementary Table S2: Additional study and patient characteristics.

| **First author and year of publication** | **Population** | **Age group** | **Mean age in years (SD)** | **Sex (female % of total)** | **Ethnicity / Fitzpatrick scale of patient** | **Total number of patients (total number of cases)** | **Number of patients included in the analysis (number of cases included in the analysis)** | **Photographer** | **Image resolution** | **Was medical history available?** |
| --- | --- | --- | --- | --- | --- | --- | --- | --- | --- | --- |
| **Articles included in the meta-analysis** | | | | | | | | | | |
| Altieri, 2017^1^ | All skin conditions | adult | NA | NA | Latino: 70·7%; White: 14·7%; Asian 9·9%; Black: 3·4%; Middle-Eastern 1·3%; / Type I-III 47%; Type IV-VI 53% | 232 (232) | 232 (232) | dermatologist | NA | NA |
| Baba, 2005^2^ | All skin conditions | pediatric and adult | 35 (2-82)† | 62·7% | NA | 228 (242) | 228 (242) | healthcare provider | 1600x1200 / 352x288 | yes |
| Barbieri, 2014^3^ | All skin conditions (hospitalized patients) | adult | 55·2 (16·2) | 64% | NA | 50 (50) | 50 (50) | healthcare provider | NA | yes |
| Barcaui, 2018^4^ | Pigmented lesions | pediatric and adult | 56·5 (11-78)† | 71% | NA | 31 (41) | 31 (41) | dermatologist | 3264x2448 | no |
| Barnard, 2000^5^ | All skin conditions | NA | NA | NA | NA | 50 (50) | 50 (50) | dermatologist | 1280x1000 | yes |
| Batalla, 2016/1^6^ | All skin conditions | pediatric (<15 years) | 9 (5) | 66% | NA | 183 (183) | 183 (183) | NA | NA | yes |
| Batalla, 2016/2^7^ | All skin conditions | adult | 45·3 (23·7) | NA | NA | 1163 | (525) | NA | NA | yes |
| Borve, 2012^8^ | All skin conditions | adult | 49 (18-95)† | 57·5% | NA | 40 (40) | 40 (40) | healthcare provider | 680x420 | yes |
| Borve, 2013^9^ | Suspected skin cancer | adult | 64 (25-94)† | 38·7% | NA | 62 (69) | 62 (69) | dermatologist | 1024x766 | yes |
| Bowns, 2006^10^ | All skin conditions (subgroup 1) | adult (>16 years) | 43·6 (17·8) | 63% | NA | 92 (92) | 92 (92) | healthcare provider | NA | yes |
|  | Suspected skin cancer (subgroup 2) | adult (>16 years) | NA | 53·1% | NA | 256 (256) | 256 (256) | non-healthcare provider | NA | yes |
| Braun, 2000^11^ | Pigmented lesions suspicious for skin cancer | NA | NA | NA | NA | 51 (55) | 51 (55) | dermatologist | 768x567 | optional |
| Carter, 2017^12^ | All skin conditions | adult* | 47 (12·4) | 74% | White Hispanic: 58%; White non-Hispanic: 14%; Black: 25%; Asian: 3% | NA | 79 (79) | healthcare provider | NA | yes |
| Castillo, 2022^13^ | All skin conditions (subgroup 1 - RT) | adult* | 63 (41-73)† | 16% | NA | 347 (347) | (4) | patient | NA | NA |
|  | All skin conditions (subgroup 2 - SAF) | adult* | 71 (61-75)† | 8% | NA | 2250 (2250) | (71) | patient | NA | yes |
| Cazzaniga, 2016^14^ | Suspected skin cancer | NA | NA | NA | NA | (302) | (135) | patient | NA | yes |
| Chan, 2000^15^ | All skin conditions | adult (>55 years) | NA | NA | NA | 74 (74) | 74 (74) | NA | NA | no |
| Chao, 2003^16^ | All skin conditions | NA | NA | NA | NA | 71 (71) | 71 (71) | dermatologist | NA | yes |
| Chen, 2010^17^ | All skin conditions | pediatric (<13 years) | 5·9 (3·9) | 50·6% | NA | 429 (429) | 429 (429) | dermatologist | NA | yes |
| Cheung, 2018^18^ | Solitary skin lesions | adult | NA | 48·7% | NA | 76 (76) | (8) | healthcare provider | NA | yes |
| Chung, 2007^19^ | All skin conditions (hospitalized patients) | adult | NA | NA | NA | 10 (10) | 10 (10) | healthcare provider | NA | yes |
| Clarke, 2023^20^ | All skin conditions | adult | 56·9 (16·2) | 49·5% | 86·9% white; 1·9% African American; 1·5% Asian; 1·9% other; / Type I - 5·8%·; Type II - 31·1%; Type III - 35·4%; Type IV - 22·3%; Type V - 3·4%; Type VI - 1·9% | 206 (308) | 206 (308) | NA | NA | yes |
| Congalton, 2015^21^ | Suspected melanoma | adult* | 68 (19-92)† | NA | NA | 310 (613) | 99 (129) | healthcare provider | NA | yes |
| Coras, 2003^22^ | Pigmented skin lesions | NA | NA | NA | NA | 100 (100) | 100 (100) | dermatologist | 512x512 | yes |
| D'elia, 2007^23^ | All skin conditions | adult | 45·4 (22·5) | NA | NA | 80 (188) | 80 (188) | healthcare provider | NA | yes |
| de Giorgi, 2016^24^ | Pigmented lesions | adult | 51·3 (26; 69)‡ | 60% | NA | 10 (10) | 10 (10) | NA | NA | yes |
| Dobry, 2021^25^ | All skin conditions | pediatric and adult | 43·4 (17·7) | 60·3% | NA | 3285 | (99) | healthcare provider | NA | yes |
| Du Moulin, 2003^26^ | All skin conditions | adult | 47 | NA | NA | 117 (117) | 117 (117) | healthcare provider | 1200x1800 | yes |
| Edison, 2008^27^ | All skin conditions | pediatric and adult | 42 (7-92)† | 69·1% | White 85·5%; African American: 11·8%; Asian 1·8%; Hispanic: 1% | 110 (110) | 110 (110) | non-healthcare provider | NA | yes |
| Eminovic, 2003^28^ | All skin conditions | pediatric and adult | 36 (14) | 55% | NA | 105 (105) | 105 (105) | patient | NA | no |
| Fabbrocini, 2008^29^ | Melanocytic and non-melanocytic "pink" lesions (poor/absent pigmentation) | adult | NA | NA | NA | (44) | NA | dermatologist | 800x600 | yes |
| Faucon, 2022^30^ | All skin conditions | pediatric and adult* | 44·5 (0-96)† | 57% | NA | 375 (375) | (298) | healthcare provider | NA | yes |
| Ferrandiz, 2017^31^ | Suspected skin cancer | adult* | 54·95 | 62·3% | NA | 454 | (228) | NA | NA | NA |
| Gabel, 2021^32^ | All skin conditions | adult | 54·1 (23·7) | 43·9% | Caucasian: 75·6%; Black: 9·8%; Unknown: 9·8%; Asian: 4·9% | 41 (41) | 41 (41) | dermatologist | NA | yes |
| Gao, 2023^33^ | Suspected skin cancer | pediatric and adult | 62·9 (0-99)§ | 56·5% | European: 93·6%; Maori, Samoan, Tongan: 7·7%; Mixed: 2·9%; Asian: 1·9%; Unknown: 0·3% | 310 (402) | (69) | healthcare provider | NA | NA |
| Gatica, 2015^34^ | All skin conditions | pediatric and adult | 37·7 (22·2) | 58% | NA | 125 (125) | 125 (125) | healthcare provider | NA | yes |
| Gemelas, 2019^35^ | Suspected or confirmed melanoma | adult | NA | NA | NA | (551) | (503) | non-healthcare provider | NA | yes |
| Gerhardt, 2021^36^ | All skin conditions | adult | NA | NA | NA | NA | (1286) | patient | NA | NA |
| Giavina-Bianchi, 2020/1^37^ | Skin neoplasms | pediatric and adult | NA | NA | NA | NA | (803) | healthcare provider | NA | yes |
| Gilmour, 1998^38^ | All skin conditions | pediatric and adult | (0-83)† | 49·2% | NA | 126 (155) | 126 (155) | healthcare provider | 352x288 | yes |
| Gyllencreutz, 2017^39^ | Suspected skin cancer (subgroup 1 - teledermoscopy) | adult* | 59·2 | 51·3% | NA | 88 (88) | 88 (88) | NA | NA | yes |
|  | Suspected skin cancer (subgroup 2 - paper referral) | adult* | 65·2 | 59·7% | NA | 77 (77) | 77 (77) | NA | NA | yes |
| Gyllencreutz, 2018^40^ | All skin conditions | NA | NA | NA | NA | 172 (316) | 172 (316) | healthcare provider | 1024x765 | NA |
| Harrison, 1998^41^ | Pigmented lesions | NA | NA | NA | NA | 657 (657) | 657 (657) | healthcare provider | NA | yes |
| Heffner, 2009^42^ | Rashes | pediatric | 6·167 (0-18)† | 40% | NA | 135 (135) | 135 (135) | healthcare provider | 1200x1600 | yes |
| Herrmann, 2005^43^ | All skin conditions | pediatric and adult | 48·1 (1-89)† | 66·7% | NA | 120 (120) | (28) | NA | 2048x1536 | yes/no |
| High, 2000^44^ | All skin conditions | pediatric and adult | 39·7 (0-81)† | 48% | Type I-IV: 96·7%; Type V-VI: 3·3% | 92 (106) | (99) | healthcare provider | 640x480 | yes |
| Hines, 2021^45^ | All skin conditions (emergency department patients) | pediatric and adult | 41§ (13-64)† | 56% | White Non-Hispanic 84%; Black 4%; Asian: 3%; Other or Unknown: 9% | 450 (527) | (293) | healthcare provider | NA | yes |
| Hue, 2015^46^ | Suspected melanoma | adult | 54§ | 33% | NA | 289 | (11) | healthcare provider | NA | optional |
| Ilie, 2022^47^ | All skin conditions | pediatric and adult | NA | NA | NA | 29 (29) | 29 (29) | patient | NA | yes |
| Ishioka, 2009^48^ | Pigmented skin lesions | pediatric and adult* | (5-8)† | 61% | NA | 300 | 64 (64) | NA | NA | NA |
| Fazil Jaber, 2023^49^ | Atypical pigmented lesions | adult | 55 (18-91)§ | 49·1% | NA | 112 (112) | (40) | healthcare provider | NA | NA |
| Janda, 2020^50^ | Suspected skin cancer | adult* | 41·8 (11·8) | 68% | Very fair: 35%; Fair: 54%; Medium: 10%; Olive or brown 1% | NA | 98 (615) | patient | NA | yes |
| Jang, 2002^51^ | All skin conditions | NA | NA | NA | NA | 77 (88) | 77 (88) | dermatologist | 764x512 | yes |
| Jobbagy, 2022^52^ | All skin conditions | pediatric and adult | 43·5 (21) | 63·3% | Caucasian: 99·3%; Other 0·7% | 749 (779) | 749 (779) | patient | min. 8 mpx | yes |
| Jolliffe, 2001^53^ | Pigmented skin lesions | pediatric and adult | (15-94)† | 66% | NA | 138 (144) | 138 (144) | dermatologist | 768x576 | yes |
| Jones, 2021^54^ | Suspected skin cancer (subgroup 1 – “suspected skin cancer pathway”) | adult* | 61 (19·2) | 56% | New Zealand European 84%; Maori: 6%; Other European 6%; Asian: 2%; Other: 2%; Pasifica: 1% | 1307 (1477) | (114) | healthcare provider | NA | NA |
|  | Suspected skin cancer (subgroup 2 – “virtual lesion clinic 2016”) | adult* | 55 (21) | 64% | New Zealand European 84%; Maori: 6%; Other European 6%; Asian: 2%; Other: 2%; Pasifica: 1% | 400 (682) | (112) | NA | NA | NA |
|  | Suspected skin cancer (subgroup 3 – “virtual lesion clinic 2020”) | adult* | 59 (16·1) | 59% | New Zealand European 84%; Maori: 6%; Other European 6%; Asian: 2%; Other: 2%; Pasifica: 1% | 108 (277) | (32) | dermatologist | NA | yes |
| Josendal, 1991^55^ | All skin conditions | pediatric and adult | NA | NA | NA | 27 (27) | (6) | healthcare provider | NA | yes |
| Kaliyadan, 2013^56^ | All skin conditions | pediatric and adult | (1-73)† | 41·6% | NA | 166 (166) | 166 (166) | healthcare provider | NA | yes |
| Keller, 2020^57^ | All skin conditions | adult | NA | NA | NA | 53 (53) | 53 (53) | healthcare provider | NA | yes |
| Koop, 2023^58^ | Suspected melanoma | pediatric and adult | 39 (17·7) | 65·8% | NA | (4 748) | (4 748) | dermatologist | NA | yes |
| Kravets, 2018^59^ | Skin neoplasms | NA | NA | NA | NA | 314 (314) | 314 (314) | healthcare provider | 832x608 | yes |
| Kroemer, 2011^60^ | Skin neoplasms | pediatric and adult | 69 (3-93)† | 53·4% | NA | 88 (113) | 88 (113) | healthcare provider | NA | yes |
| Krupinski, 1999^61^ | All skin conditions | NA | NA | NA | NA | 308 (308) | 308 (308) | healthcare provider | NA | NA |
| Kvedar, 1997^62^ | All skin conditions | adult | 40 (18-84)† | 49% | NA | 116 (128) | 116 (128) | healthcare provider | NA | NA |
| Lamel, 2012^63^ | Skin cancer screening | adult | 45·2 (13·6) | 58·1% | White: 84·88%; Black: 1·16%; Asian: 11·63%; Hawaiian or Pacific Islander: 2·33% | 86 (137) | 86 (137) | healthcare provider | NA | yes |
| Lasierra, 2012^64^ | All skin conditions (subgroup 1) | pediatric* | 7 (4) | 47% | NA | 82 (82) | 82 (82) | dermatologist | NA | no |
|  | All skin conditions (subgroup 2) | adult* | 56·2 (17·4) | 53% | NA | 38 (38) | 38 (38) | healthcare provider | 1152x768 | yes |
| Lepe, 2004^65^ | All skin conditions | pediatric and adult | 36·8 (0-82)† | 52% | NA | 50 (50) | 50 (50) | healthcare provider | 352x288 | yes |
| Lesher, 1998^66^ | All skin conditions | adult | NA | NA | NA | 60 (68) | 60 (68) | healthcare provider | NA | yes |
| Lim, 2001^67^ | All skin conditions | adult | NA | NA | NA | 72 (80) | 72 (80) | healthcare provider | NA | yes |
| Loane, 1997^68^ | All skin conditions | pediatric and adult* | 41 (0-81)† | 47·7% | NA | 65 (79) | 65 (79) | dermatologist | 756x504 | yes |
| Loane, 1998^69^ | All skin conditions (subgroup 1 – “camera 1”) | pediatric and adult* | 37·1 (0-89)† | 58·5% | NA | 65 (75) | 65 (75) | NA | NA | yes |
|  | All skin conditions (subgroup 2 – “camera 2”) | pediatric and adult | 41 (23) | 89% | NA | 351 (427) | 351 (427) | healthcare provider | 1200x1600 | yes |
| Lowitt, 1998^70^ | All skin conditions | adult | 65 (23-85)§ | 5% | White: 60%; African American: 40% | 131 (318) | (130) | healthcare provider | NA | yes |
| Lyon, 1997^71^ | All skin conditions | NA | NA | NA | NA | 100 (100) | 100 (100) | patient | NA | yes |
| Maclellan, 2021^72^ | Pigmented lesions | adult | NA | NA | Type I-III: 100% | 184 (209) | 184 (209) | NA | 3648x2736 | yes |
| Mahendran, 2005^73^ | Suspected skin cancer | NA | NA | NA | NA | 163 (163) | 163 (163) | healthcare provider | NA | yes |
| Mallett, 2003^74^ | All skin conditions | pediatric and adult | (0-94)† | 52% | NA | 325 (727) | (213) | NA | 1632x1224 | NA |
| Manahan, 2015^75^ | Pigmented lesions | adult | (50-64)† | 51% | "Fair": 90% | 49 (49) | 49 (49) | healthcare provider | NA | optional |
| Marchell, 2017^76^ | All skin conditions | adult | NA | NA | NA | 214 | (134) | NA | NA | NA |
| Markun, 2017^77^ | Suspected skin cancer | adult | 40·4 (17·3) | 60·1% | Type I: 6·4%; Type II: 43·1%; Type III: 34%; Type IV: 6·4%; Type V: 2·1%; Type VI: 0·5% | 188 (195) | (192) | healthcare provider | 1600x1200 | yes |
| Massone, 2007^78^ | Pigmented lesions | pediatric and adult | 43·4 (14-78)† | 66·7% | NA | 18 (18) | 18 (18) | healthcare provider | NA | NA |
| Massone, 2013^79^ | Suspected skin cancer | adult | 47 (18-84)† | 7% | NA | 690 | (32) | healthcare provider | 1600x1200 | yes |
| Montejano, 2022^80^ | All skin conditions | NA | NA | 7·3% | Caucasian 80·4%; Non-Caucasian 3·1%; Unknown: 16·5% | 357 (481) | (19) | healthcare provider | NA | yes |
| Moreno-Ramirez, 2005^81^ | Pigmented lesions | pediatric and adult | 43 (2-84)† | 65% | NA | 219 (219) | (131) | healthcare provider | NA | yes |
| Moreno-Ramirez, 2006^82^ | Pigmented lesions | pediatric and adult | 38·8 (1-73)† | 70·5% | NA | 61 (61) | 61 (61) | healthcare provider | NA | yes |
| Moreno-Ramirez, 2007^83^ | Suspected skin cancer | adult* | 45·5 (44·1; 47)‡ | 54·6% | NA | 2009 | 1285 (1285) | healthcare provider | NA | yes |
| Muir, 2011^84^ | Acute/subacute skin conditions (emergency department patients) | adult | 47 (20) | 65% | NA | 60 (60) | 60 (60) | healthcare provider | 352x288 | yes |
| Naka, 2018^85^ | All skin conditions | adult | 36·6 (20) | 60·1% | Hispanic: 46·7%; Non-Hispanic white: 30·5%; Non-Hispanic black: 10%; Unknown: 8%; Other: 4·8% | 499 | (19) | NA | NA | NA |
| Nami, 2015^86^ | All skin conditions, except pigmented lesions | pediatric and adult | 54 (0-100)† | 52·2% | Caucasian: 99%; North African: 1% | 391 (391) | 391 (391) | patient (parent) | NA | yes |
| Ng, 2011^87^ | Suspected skin cancer | NA | NA | NA | NA | 100 (100) | 100 (100) | healthcare provider | NA | yes |
| Nordal, 2001^88^ | All skin conditions | adult | 40 (17-82)† | 48·8% | NA | (121) | (112) | healthcare provider | 800x600 | yes |
| Norton, 1997^89^ | All skin conditions | NA | NA | NA | NA | 126 (126) | (4) | healthcare provider | 640x480 | yes |
| O'Connor, 2017^90^ | All skin conditions | pediatric | NA | 55% | White: 48%; Black: 33%; Asian: 10%; Pacific Islander 3%; Other: 8% | 40 (40) | 40 (40) | healthcare provider | NA | yes |
| Oakley, 1997^91^ | All skin conditions | pediatric and adult | (2-86)† | 59·6% | NA | 104 (135) | 104 (135) | NA | 800x600 | yes |
| Oakley, 1998^92^ | All skin conditions | pediatric and adult | NA | NA | NA | 100 (100) | 28 (28) | healthcare provider | 1280x1024 / 1280x960 | yes |
| Oakley, 2006^93^ | All skin conditions | adult | (18-92)† | 64·4% | Caucasian: 97% / Type I-III: 97% | 73 | (29) | healthcare provider | 1024x768 | yes |
| Okita, 2016^94^ | All skin conditions (hospitalized patients) | NA | NA | NA | NA | 100 (100) | 100 (100) | healthcare provider | 353x288 | no |
| Oztas, 2004^95^ | All skin conditions | NA | NA | NA | NA | 125 (125) | 125 (125) | dermatologist | NA | NA |
| Pak, 2003^96^ | All skin conditions | adult | 59 (18-92)† | NA | Caucasian 82%; African American: 13%; Asian or Hispanic: 5% | 404 (404) | 404 (404) | NA | NA | yes |
| Paradela-De-La-Morena, 2015^97^ | All skin conditions | pediatric | 7·7 (5·1) | 50·4% | NA | 383 (421) | (135) | NA | NA | NA |
| Phillips, 1997^98^ | All skin conditions | pediatric and adult | 37 (1-68)† | 60% | White: 73·3%; Black: 25%; Other: 1·7% | 60 (79) | 60 (79) | healthcare provider | 640x480 | yes |
| Phillips, 1998^99^ | Skin cancer screening | adult | 46·7 | 84·3% | White: 74·5%; Unknown: 19·6%; Non-white 5·9% | 51 (107) | 51 (107) | dermatologist | NA | yes |
| Preclaro, 2022^100^ | All skin conditions | pediatric and adult | 41 (0-75)† | 50% | Filipino: 100% | 60 (60) | 60 (60) | dermatologist | NA | yes |
| Rajagopal, 2009^101^ | All skin conditions | adult | NA | 37·4% | NA | NA | (115) | healthcare provider | NA | yes |
| Rashid, 2003^102^ | All skin conditions | pediatric and adult | NA | NA | NA | 33 (33) | 33 (33) | healthcare provider | NA | yes |
| Ribas, 2010^103^ | All skin conditions | pediatric and adult | 34·7 (18·1) | 53·4% | Brown: 65·3%; White: 34·1%; Black: 1% | 174 (174) | 174 (174) | healthcare provider | NA | yes |
| Rios, 2012^104^ | All skin conditions | adult | NA | 63·3% | NA | 30 (30) | 30 (30) | healthcare provider | NA | yes |
| Romero, 2006^105^ | All skin conditions (subgroup 1 - SAF) | pediatric and adult* | 42·4 (24·8) | 54% | NA | 74 (74) | 74 (74) | healthcare provider | NA | yes |
|  | All skin conditions (subgroup 2 - HY) | pediatric and adult* | 40·9 (23·5) | 70% | NA | 47 (47) | 47 (47) | dermatologist | 1296x864 | yes |
| Romero, 2010^106^ | All skin conditions (subgroup 1 - SAF) | adult* | NA | NA | NA | NA | (170) | dermatologist | NA | yes |
|  | All skin conditions (subgroup 2 - HY) | adult* | NA | NA | NA | NA | (158) | healthcare provider | NA | yes |
| Romero Aguilera, 2014^107^ | All skin conditions | pediatric and adult* | 36 (0-86)† | 56% | NA | NA | 457 (457) | healthcare provider | 352x288 | yes |
| Rubegni, 2011^108^ | All skin conditions | adult (geriatric) | 80·6 (66-97)† | 53·9% | NA | 130 (130) | 130 (130) | dermatologist | 704x576 | yes |
| Ruiz, 2009^109^ | All skin conditions | pediatric and adult | (0-67)† | 66·3% | NA | 83 (172) | 83 (172) | NA | 4320x3240 | yes |
| Saleh, 2017^110^ | All skin conditions | pediatric and adult | NA | 50·7% | NA | 600 (600) | 600 (600) | patient | NA | yes |
| Santosa, 2023^111^ | All skin conditions (emergency department patients) | adult | (17-81)† | 48·3% | NA | 147 | (120) | healthcare provider | NA | yes |
| Schiener, 2001^112^ | All skin conditions | NA | NA | NA | NA | 60 (60) | 60 (60) | healthcare provider | 3072x2304 | yes |
| Senel, 2014^113^ | Benign and malignant skin lesions | adult | 63 (13) | 43% | NA | 120 (120) | NA | dermatologist | NA | yes |
| Shin, 2014^114^ | All skin conditions (military setting) | adult | 20·6 (18; 26)‡ | 0 | NA | 100 (100) | 100 (100) | healthcare provider | 640x480 | yes |
| Silva, 2009^115^ | All skin conditions | adult (>14 years) | (14-62)† | 44% | NA | 60 (60) | 60 (60) | healthcare provider | 1600x1200 | yes |
| Silveira, 2019^116^ | Suspected skin cancer | adult | 68 (39-91)† | 69% | NA | 49 (49) | 49 (49) | dermatologist | NA | yes |
| Sola-Ortigosa, 2020^117^ | Suspected actinic keratosis | adult | 72·8 (8·6) | 43·2% | Caucasian 99·7%; Asian: 0·15%; Latin: 0·15%; / Type I: 10·7%; Type II: 48·5%; Type III: 25·8%; Type IV: 12·5; Type V: 2·4% | 636 (1000) | NA | healthcare provider | NA | yes |
| Taberner Ferrer, 2009^118^ | All skin conditions | pediatric and adult | 55·3 (21·5) | 59·5% | NA | 158 | (129) | NA | NA | no |
| Tait, 1999^119^ | All skin conditions | NA | NA | NA | NA | 30 (30) | 30 (30) | healthcare provider | 768x288 | yes |
| Tan, 2010^120^ | All skin conditions | pediatric and adult | (11-94)† | 63% | European: 94%; Maori 6%; / Type I: 12·5%; Type II: 69·5%; Type III: 12·5%; Type IV: 5%; Type V: 0·5% | 200 (491) | 200 (491) | healthcare provider | NA | yes |
| Taslidere, 2022^121^ | All skin conditions | pediatric and adult | 32·7 (17·4) | 56·8% | NA | 546 (595) | 546 (595) | healthcare provider | NA | yes |
| Taslidere, 2023 ^122^ | All skin conditions | pediatric | 11 (4·6) | 55·9% | NA | 93 (93) | 93 (93) | patient | NA | yes |
| Taylor, 2001^123^ | All skin conditions | adult | NA | NA | NA | (188) | (188) | dermatologist | 640x480 | yes |
| Teague, 2022^124^ | Suspected melanoma | adult* | 67 (16-95)† | 43% | Type I: 30%; Type II: 46%; Type III: 19%; Type IV: 4%; Unknown: 1% | 591 (591) | 591 (591) | healthcare provider | NA | yes |
| Teoh, 2022^125^ | Suspected skin cancer | pediatric and adult | 53·5 (0-100)† | 63·1% | European: 89·5%; Maori or Pacific Islander: 5·4%; Asian: 2·4%; Other: 2·5%; / Type I - 6·7%; Type II: 62·1%; Type III: 22·8%; Type IV: 7·8%; Type V-VI: 0·4% | 6479 (11005) | (523) | healthcare provider | NA | yes |
| Tian, 2017^126^ | Esthetic conditions | adult | 29·3 (16-67)† | 73·5% | NA | NA | 102 (346) | NA | NA | NA |
| Tucker, 2005^127^ | All skin conditions | pediatric and adult | (3-87)† | 72% | NA | 75 (84) | 75 (84) | healthcare provider | NA | yes |
| Vano-Galvan, 2011^128^ | All skin conditions | adult | NA | NA | NA | 100 (100) | 100 (100) | healthcare provider | 1600x1200 | yes |
| Villa, 2020^129^ | All skin conditions (emergency department patients) | adult | 43·8 (18·4) | 44% | NA | 50 (50) | 50 (50) | patient | 640x480 | yes |
| Wang, 2017^130^ | Suspected melanoma | adult | NA | NA | NA | (61) | (61) | NA | NA | yes |
| Warshaw, 2009/1^131^ | Pigmented neoplasms | adult | 66 (23-94)† | 4·2% | Caucasian 97·1%; African American: 1·3%; Other: 1·5% / Type I: 6·8%; Type II: 26·2%; Type III: 55·5%; Type IV-VI: 11·3% | 542 (542) | 542 (542) | NA | 1280x1000 | yes |
| Warshaw, 2015^132^ | Skin neoplasms | adult | 68 (19-94)† | 3·2% | Caucasian: 97·5%; African American 1·5%; Hispanic or Latino: 0·4%; Asian: 0·1%; Other: 0·1%; / Type I: 9·7%; Type II: 28·3; Type III: 47·6%; Type IV-VI: 14·3% | 2152 (3021) | (3003) | NA | NA | NA |
| Weingast, 2013^133^ | All skin conditions | adult | 39 (28; 49)¶ | 62% | NA | 263 (299) | 263 (299) | NA | 640x480 | yes |
| Whited, 1998^134^ | Suspected skin cancer | adult | NA | NA | NA | 12 (13) | 12 (13) | healthcare provider | NA | yes |
| Whited, 1999^135^ | All skin conditions | adult | 61 (22-82)† | 2·3% | White: 79·8%; Black: 20·2% | 129 (168) | 129 (168) | NA | NA | yes |
| Yamazaki, 2003^136^ | All skin conditions | NA | NA | NA | NA | (112) | (112) | dermatologist | NA | yes |
| Zanini, 2013^137^ | All skin conditions | adult | NA | NA | NA | 100 (100) | 100 (100) | NA | 640x480 | yes |
| Zelickson, 1997^138^ | All skin conditions (nursing home patients) | adult (elderly) | NA | NA | NA | 29 (30) | 29 (30) | healthcare provider | NA | yes |
| Zink, 2017/1^139^ | All skin conditions | pediatric and adult | 50·6 (1-89)† | 40·5% | NA | 195 (195) | 195 (195) | NA | NA | yes |
| Zink, 2017/2^140^ | All skin conditions (in need of dermoscopic evaluation) | adult | NA | NA | NA | 26 (26) | 26 (26) | dermatologist | NA | yes |
| **Articles included in the systematic review** | | | | | | | | | | |
| Alfageme, 2021^141^ | Palpable nodular skin lesions | adult | 47 (23) | 65% | NA | 143 (147) | NA | healthcare provider | NA | yes |
| Creadore, 2023^142^ | Cellulitis / pseudocellulitis patients | NA | NA | NA | Type I: 33·3%; Type II: 20%; Type III: 33·3%; Type V: 10%; Type VI: 10% | 10 (10) | NA | NA | NA | yes |
| Giavina-Bianchi, 2020/2^143^ | Atopic dermatitis | pediatric and adult | NA | NA | NA | 1648 (2058) | (109) | healthcare provider | NA | yes |
| Giavina-Bianchi, 2020/3^144^ | Inflammatory dermatoses | pediatric and adult | NA | NA | NA | NA | 739 (739) | healthcare provider | NA | yes |
| Lozzi, 2007^145^ | Neoplastic and inflammatory dermatoses | pediatric and adult | 51·1 (12-83)† | 45·5% | NA | 33 (33) | NA | healthcare provider | 2048x1536 | yes |
| Ludzik, 2016/1^146^ | Suspected melanoma | NA | NA | NA | NA | (100) | (100) | NA | NA | no |
| Ludzik, 2016/2^147^ | Pink cutaneous lesions | NA | NA | NA | NA | 316 (316) | NA | healthcare provider | 960x1280 | yes |
| Rao, 2013^148^ | Lesions that had been selected for removal for either cosmetic or medical reasons | adult | NA | NA | NA | (334) | NA | NA | NA | NA |
| Senel, 2013^149^ | Non-melanocytic skin tumor | adult | 55 | 49% | NA | 150 (150) | NA | dermatologist | NA | no |
| Shah, 2023^150^ | Dermatitis | pediatric and adult* | NA | 65·8% | White: 76·2%; Black: 10·5%; Asian: 6·5%; Other: 7·2% | 1045(1045) | 120 (120) | healthcare provider | 1600x1200 | yes |
| Tognetti, 2021^151^ | Atypical melanocytic lesions | NA | NA | NA | NA | (979) | NA | NA | NA | yes |
| Trindade, 2008^152^ | Suspicious for leprosy | NA | NA | NA | NA | 106 (106) | NA | NA | NA | no |
| Tugrul, 2022^153^ | Non-melanocytic skin lesions | adult | 47 (18-83)§ | 50% | NA | 26 (26) | NA | NA | NA | NA |
| Van der Heijden, 2013^154^ | Pigmented lesions | pediatric and adult | 47 (6-84)§ | 55% | NA | 105 (108) | NA | dermatologist | NA | yes |
| Warshaw, 2009/2^155^ | Non-pigmented neoplasms | adult | 71 (21-94)† | 2·2% | Caucasian 98·9%; African American 0·7%; Other: 0·4%; / Type I: 12·9%; Type II: 30·1%; Type III: 40·4%; Type IV-VI: 16·6% | 728 (728) | NA | non-healthcare provider | 3072x2304 | yes |
| Witkowski, 2017^156^ | suspected melanoma | adult | NA | NA | NA | 1000 | NA | healthcare provider | NA | NA |

*Data on age refers to the patients included in the analysis

† Mean age in years (range)

‡Mean age in years (95% CI lower; 95% CI upper)

§Median age in years (range)

¶Mean age in years (IQR 25%; IQR 75%)

NA: not applicable; SD: standard deviation; CI: confidence interval; IQR: interquartile range; SAF: store-and-forward; RT: real-time; HY: hybrid

# Supplementary Figure S1: Forest plot comparing the diagnostic concordance between teledermatology providers and in-person dermatologists subgrouped by the communication platform in the “all skin conditions” group, excluding undiagnosed cases from the analysis.


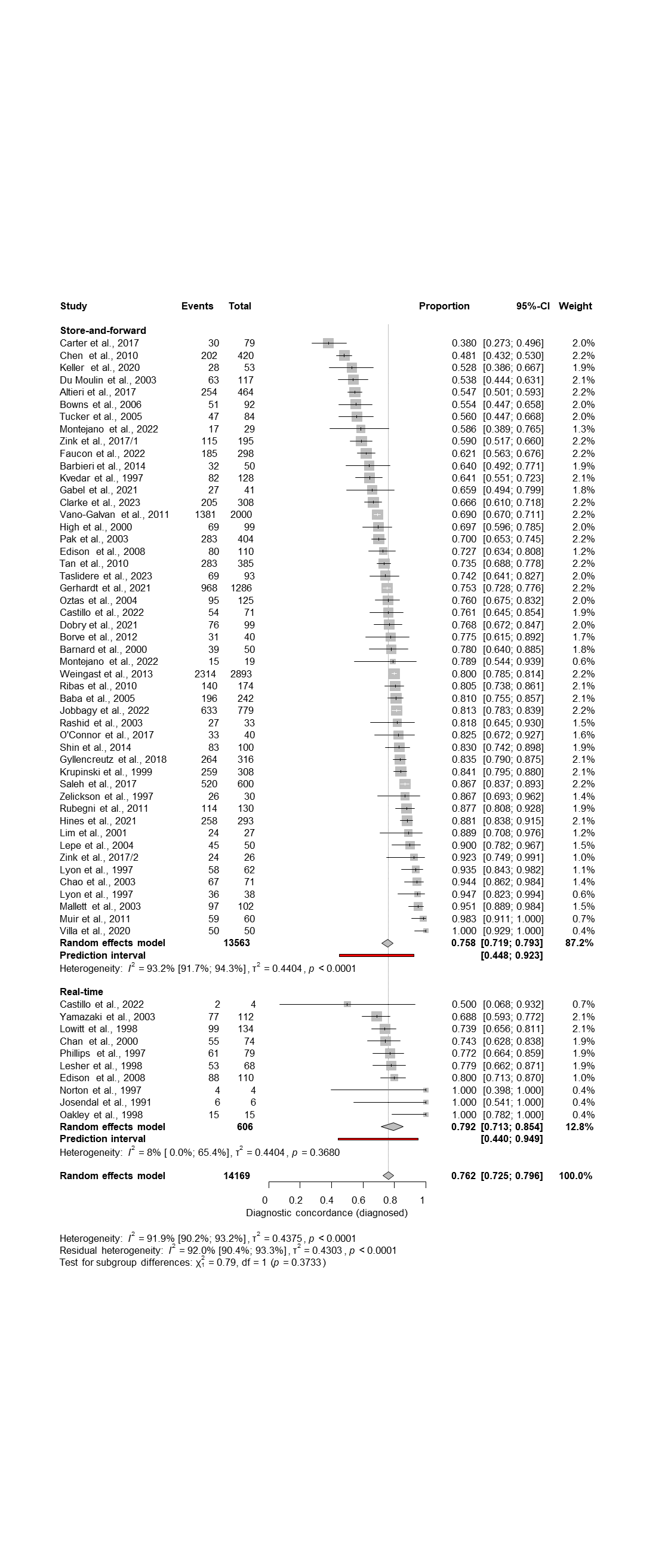


# Supplementary Figure S2: Forest plot comparing the diagnostic concordance between teledermatology providers and in-person dermatologists subgrouped by the communication platform in the “skin cancer” group, excluding undiagnosed cases from the analysis.


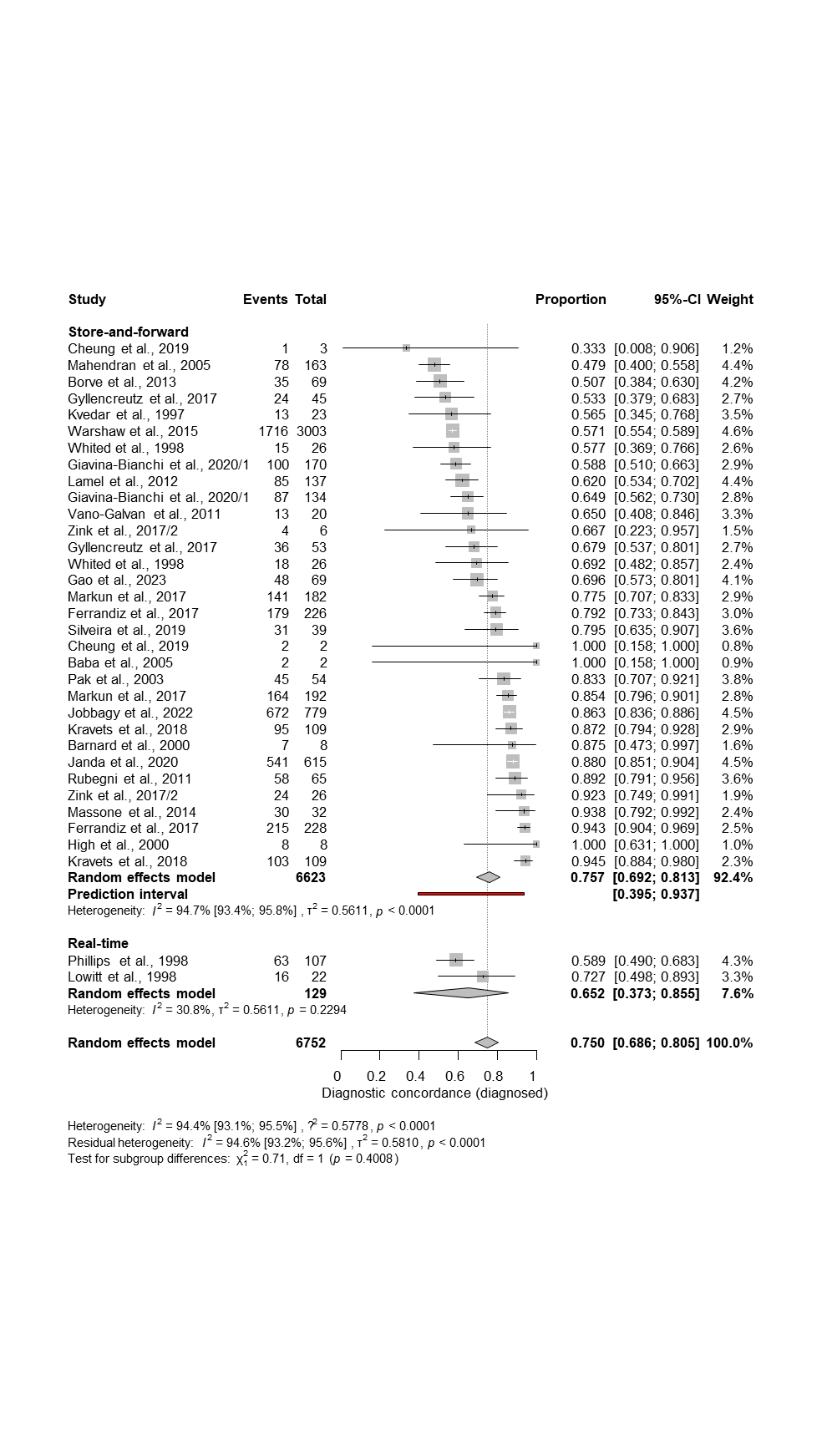


# Supplementary Figure S3: Forest plot comparing the diagnostic concordance between teledermatology providers and in-person dermatologists subgrouped by the communication platform in the “pigmented lesions” group, excluding undiagnosed cases from the analysis.


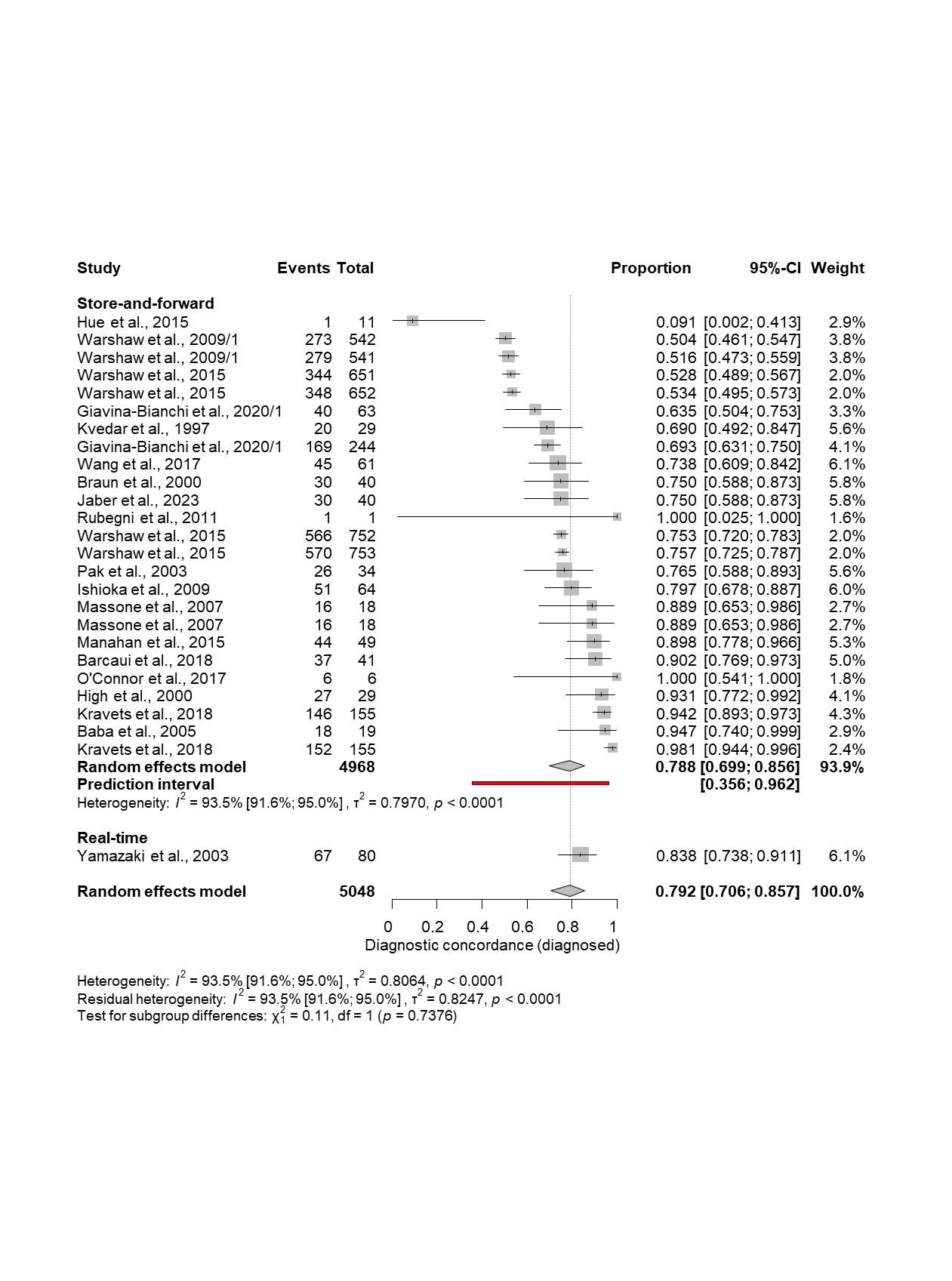


# Supplementary Figure S4: Forest plot comparing the diagnostic concordance between teledermatology providers and in-person dermatologists subgrouped by the communication type in the “all skin conditions” group, excluding undiagnosed cases from the analysis.


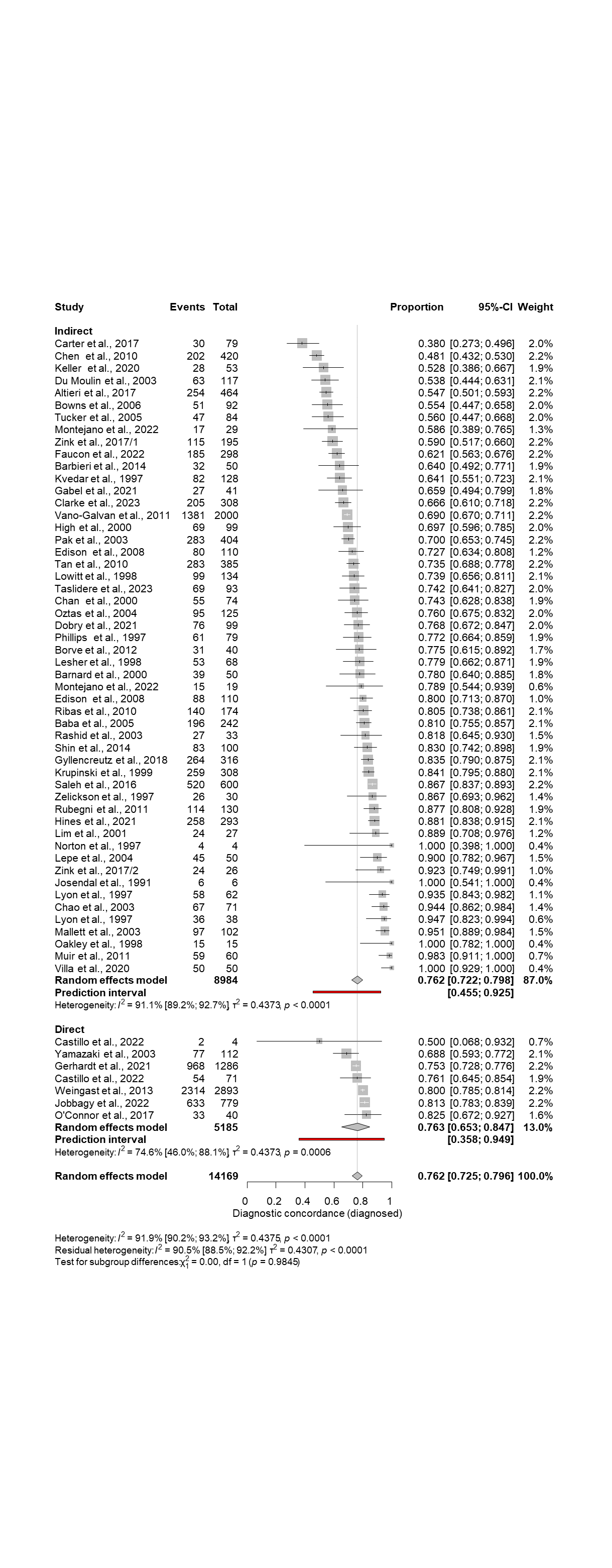


# Supplementary Figure S5: Forest plot comparing the diagnostic concordance between teledermatology providers and in-person dermatologists subgrouped by the communication type in the “skin cancer” group, excluding undiagnosed cases from the analysis.


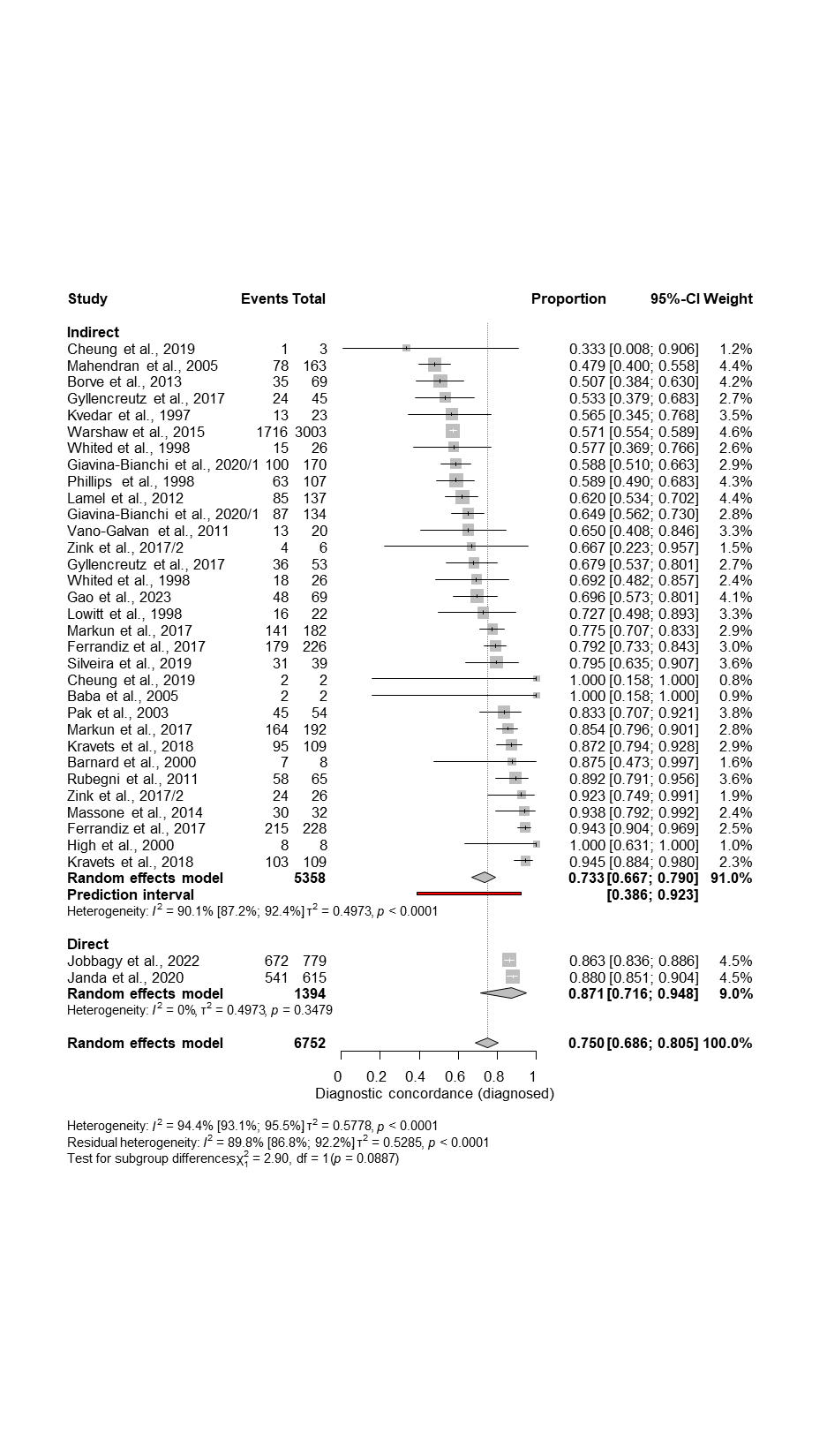


# Supplementary Figure S6: Forest plot comparing the diagnostic concordance between teledermatology providers and in-person dermatologists subgrouped by the communication type in the “pigmented lesions group”, excluding undiagnosed cases from the analysis.


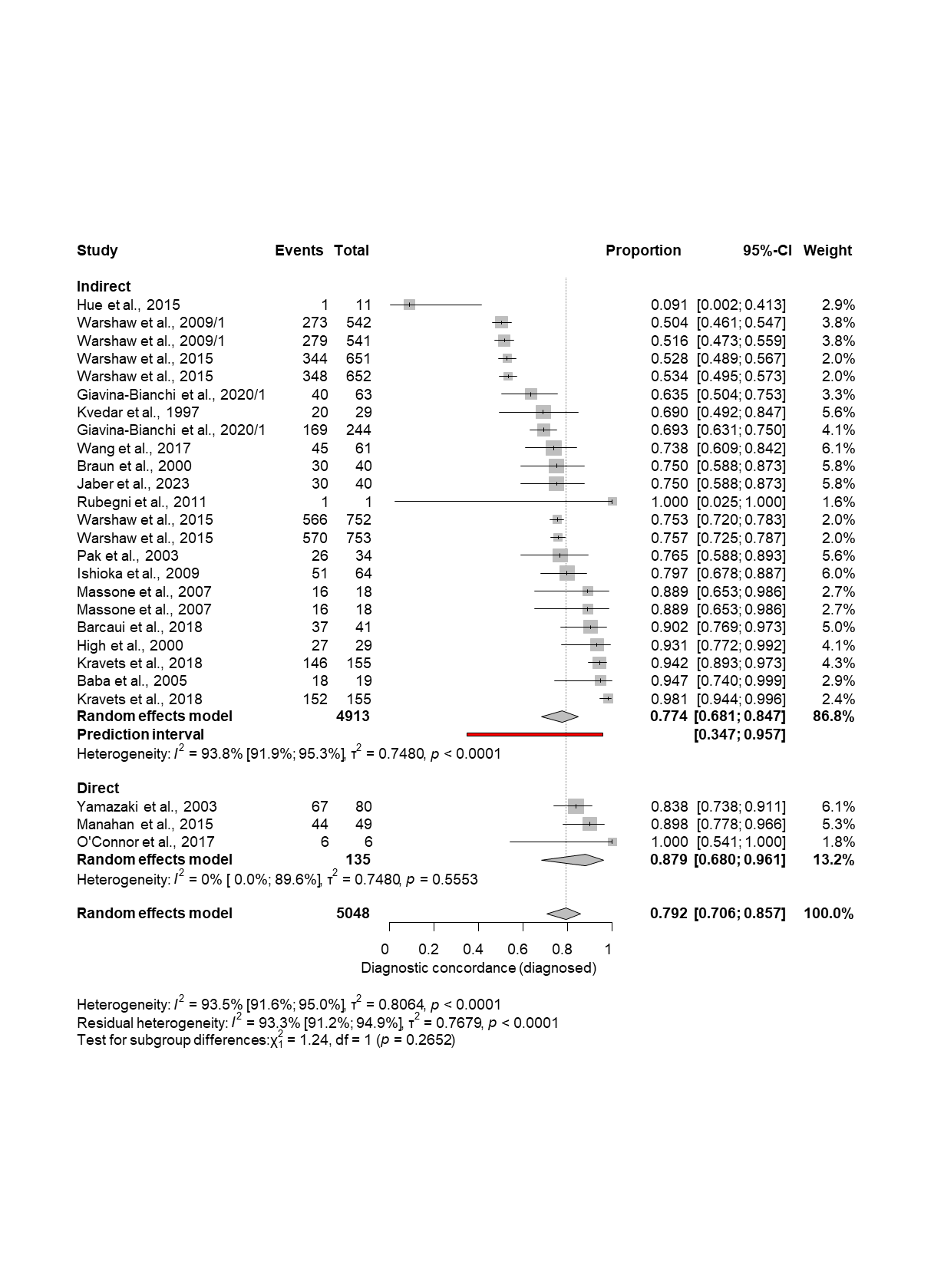


# Supplementary Figure S7: Forest plot comparing the diagnostic concordance between teledermatology providers and in-person dermatologists subgrouped by the use of dermoscopy in the “all skin conditions” group, excluding undiagnosed cases from the analysis.


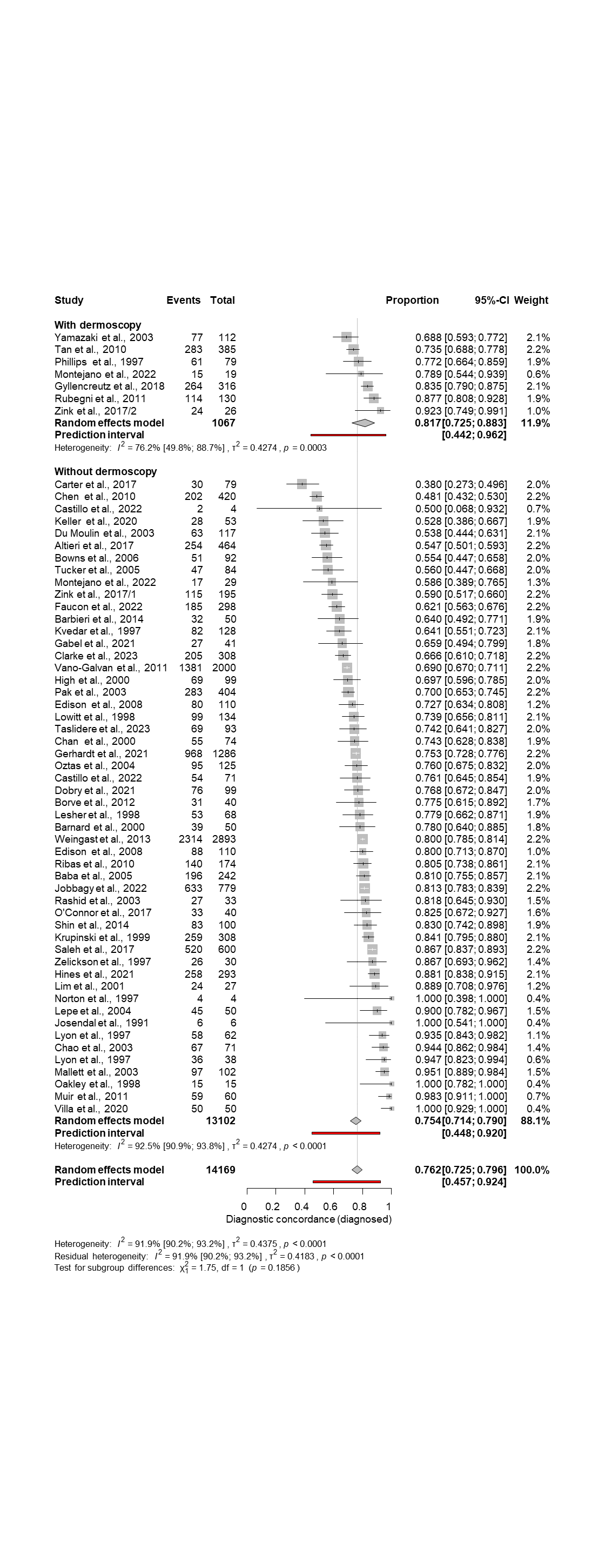


# Supplementary Figure S8: Forest plot comparing the diagnostic concordance between teledermatology providers and in-person dermatologists subgrouped by the use of dermoscopy in the “skin cancer” group, excluding undiagnosed cases from the analysis.


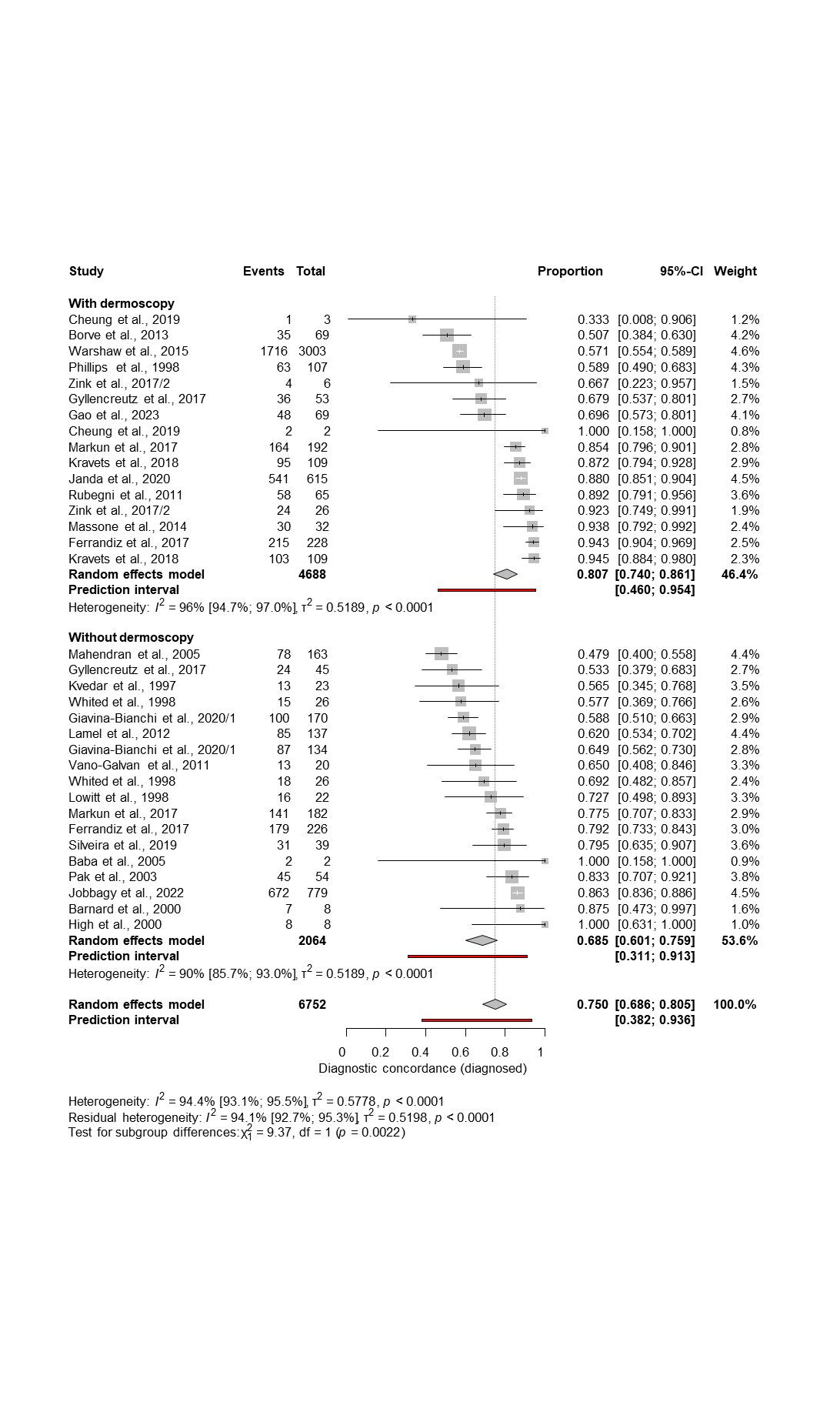


# Supplementary Figure S9: Forest plot comparing the diagnostic concordance between teledermatology providers and in-person dermatologists subgrouped by the use of dermoscopy in the “pigmented lesions” group, excluding undiagnosed cases from the analysis.


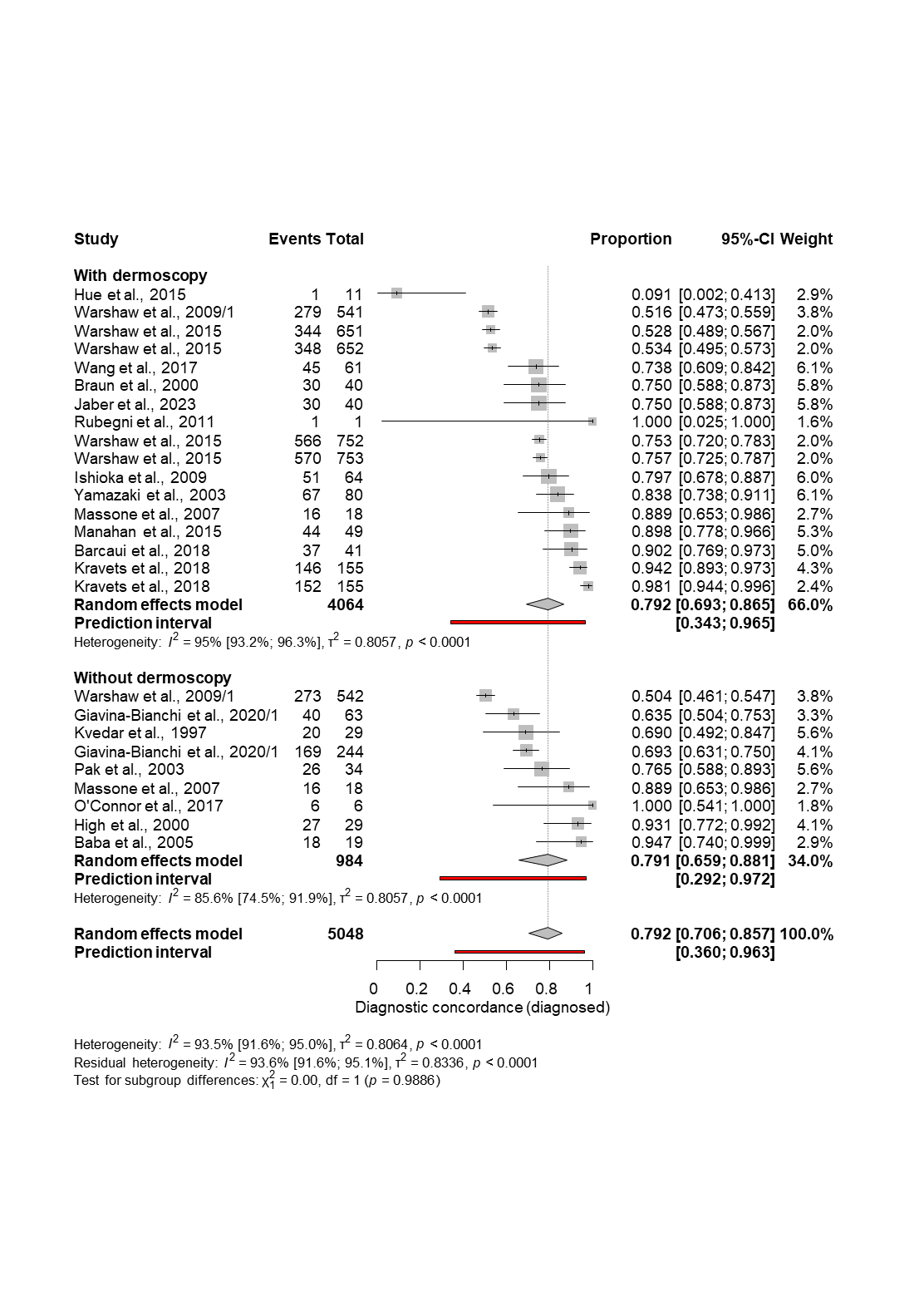


# Supplementary Figure S10: Forest plot comparing the diagnostic concordance between teledermatology providers and in-person dermatologists subgrouped by photography device in the “all skin conditions” group, excluding undiagnosed cases from the analysis.


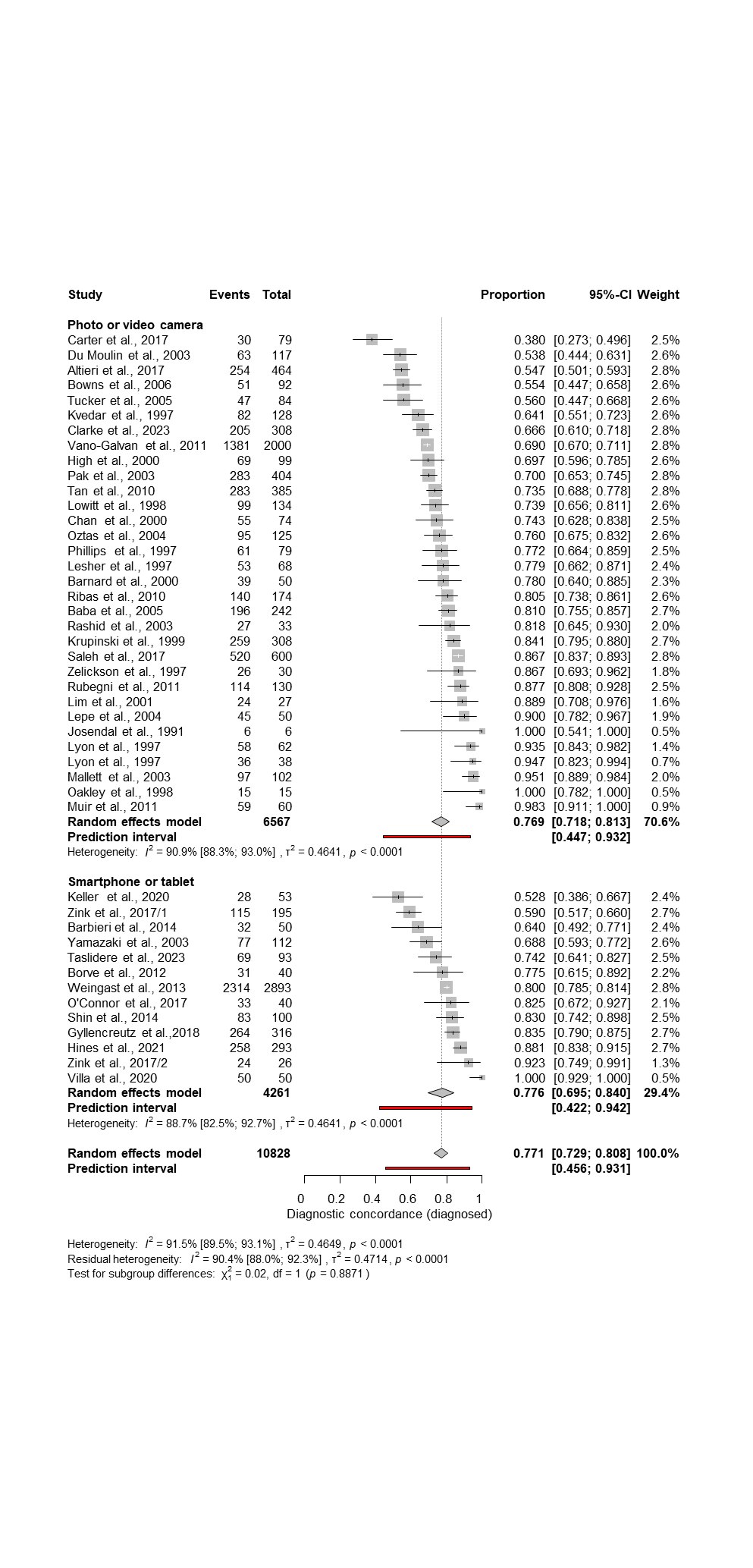


# Supplementary Figure S11: Forest plot comparing the diagnostic concordance between teledermatology providers and in-person dermatologists subgrouped by photography device in the “skin cancer” group, excluding undiagnosed cases from the analysis.


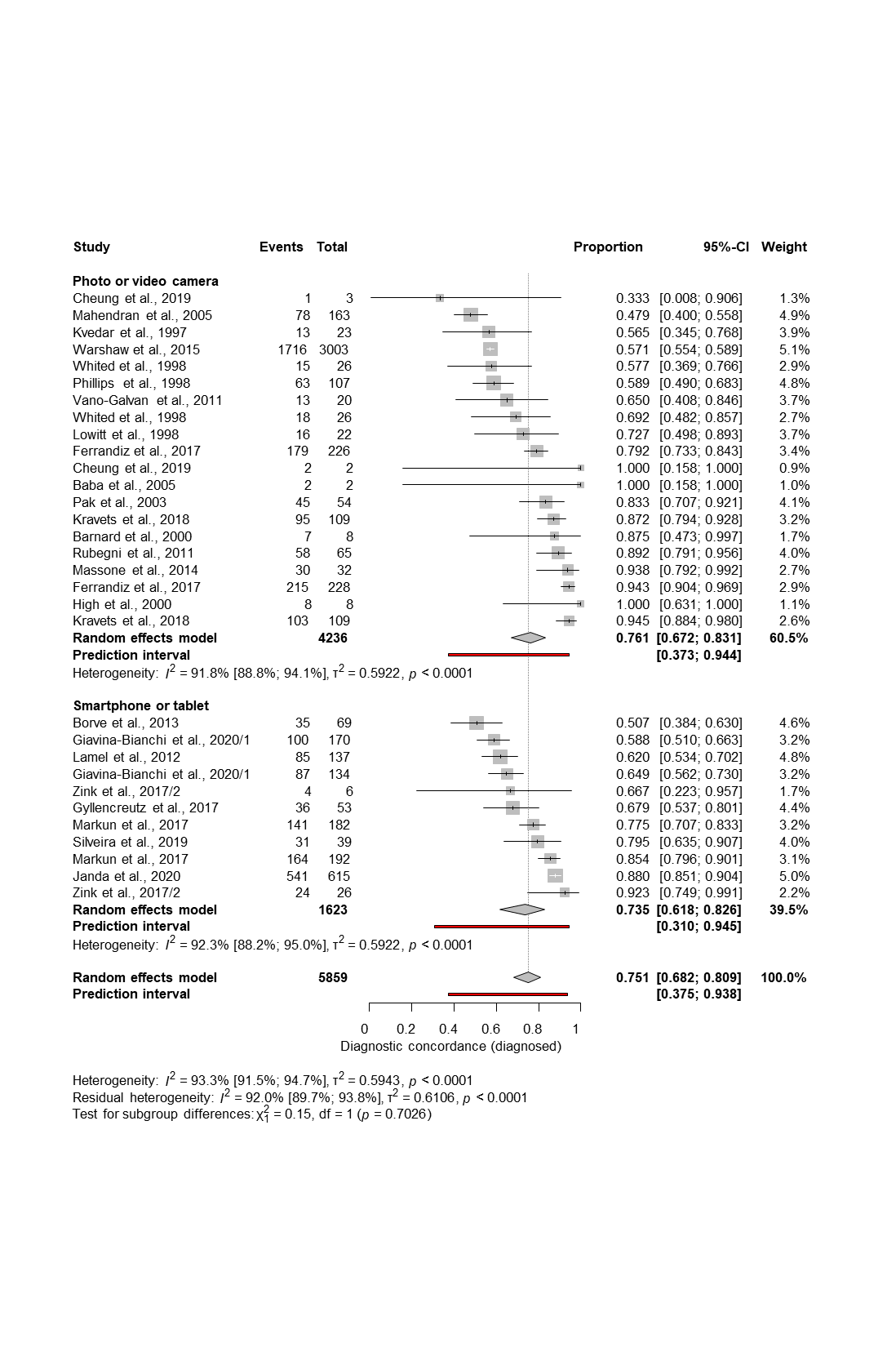


# Supplementary Figure S12: Forest plot comparing the diagnostic concordance between teledermatology providers and in-person dermatologists subgrouped by photography device in the “pigmented lesions” group, excluding undiagnosed cases from the analysis.


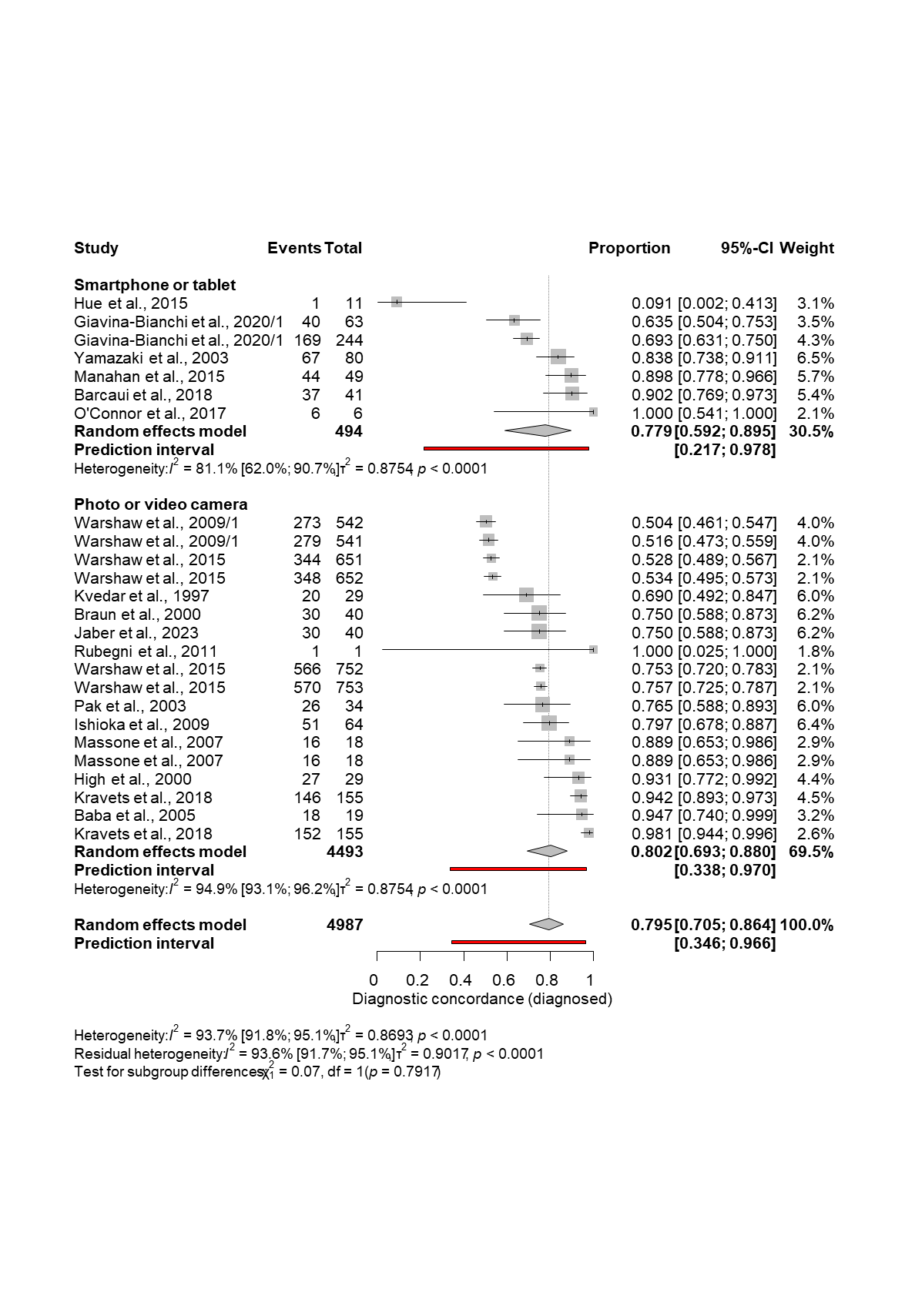


# Supplementary Figure S13: Forest plot comparing the diagnostic concordance between teledermatology providers and in-person dermatologists subgrouped by training for image acquisition in the “all skin conditions” group, excluding undiagnosed cases from the analysis.


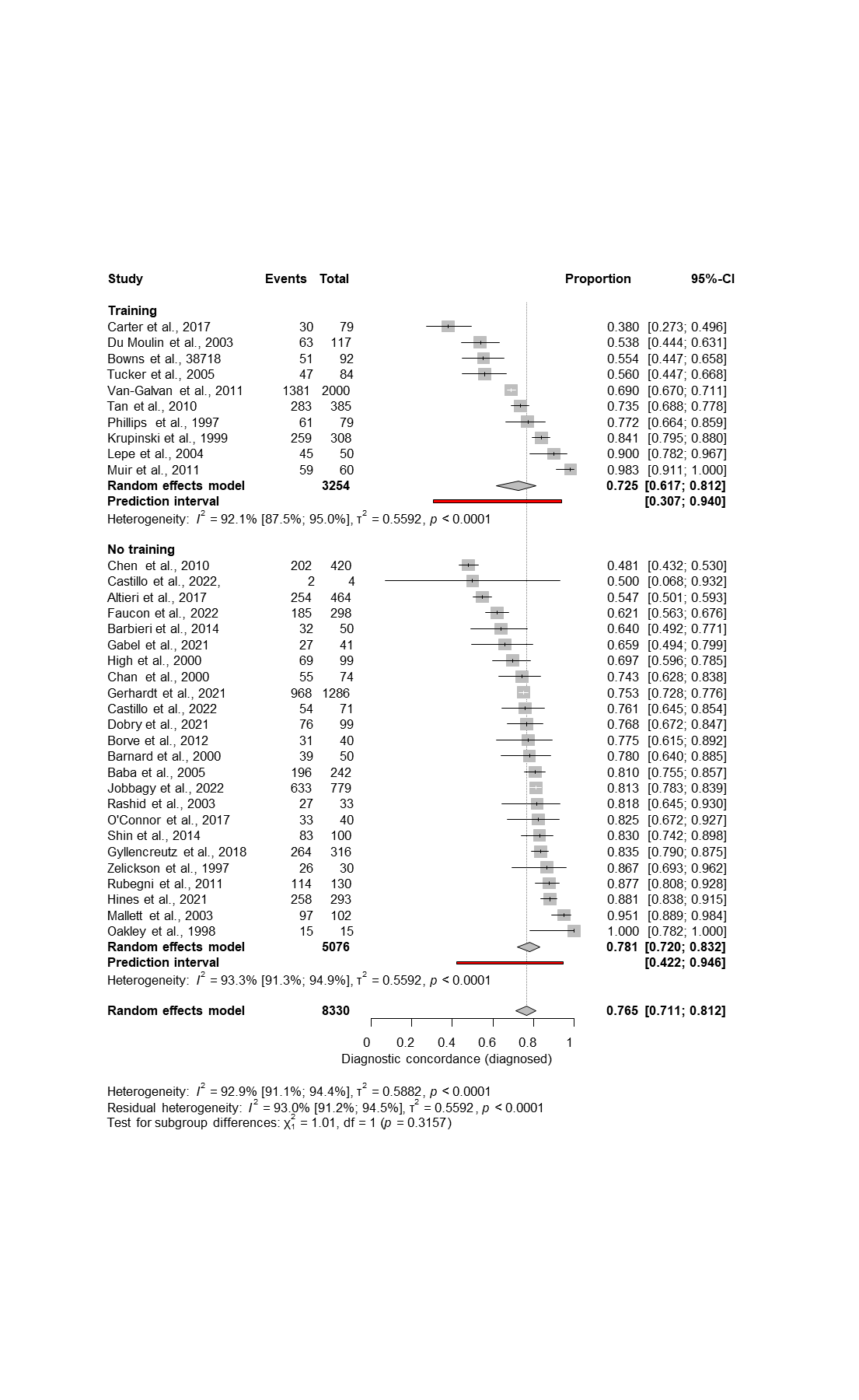


# Supplementary Figure S14: Forest plot comparing the diagnostic concordance between teledermatology providers and in-person dermatologists subgrouped by training for image acquisition in the “skin cancer” group, excluding undiagnosed cases from the analysis.


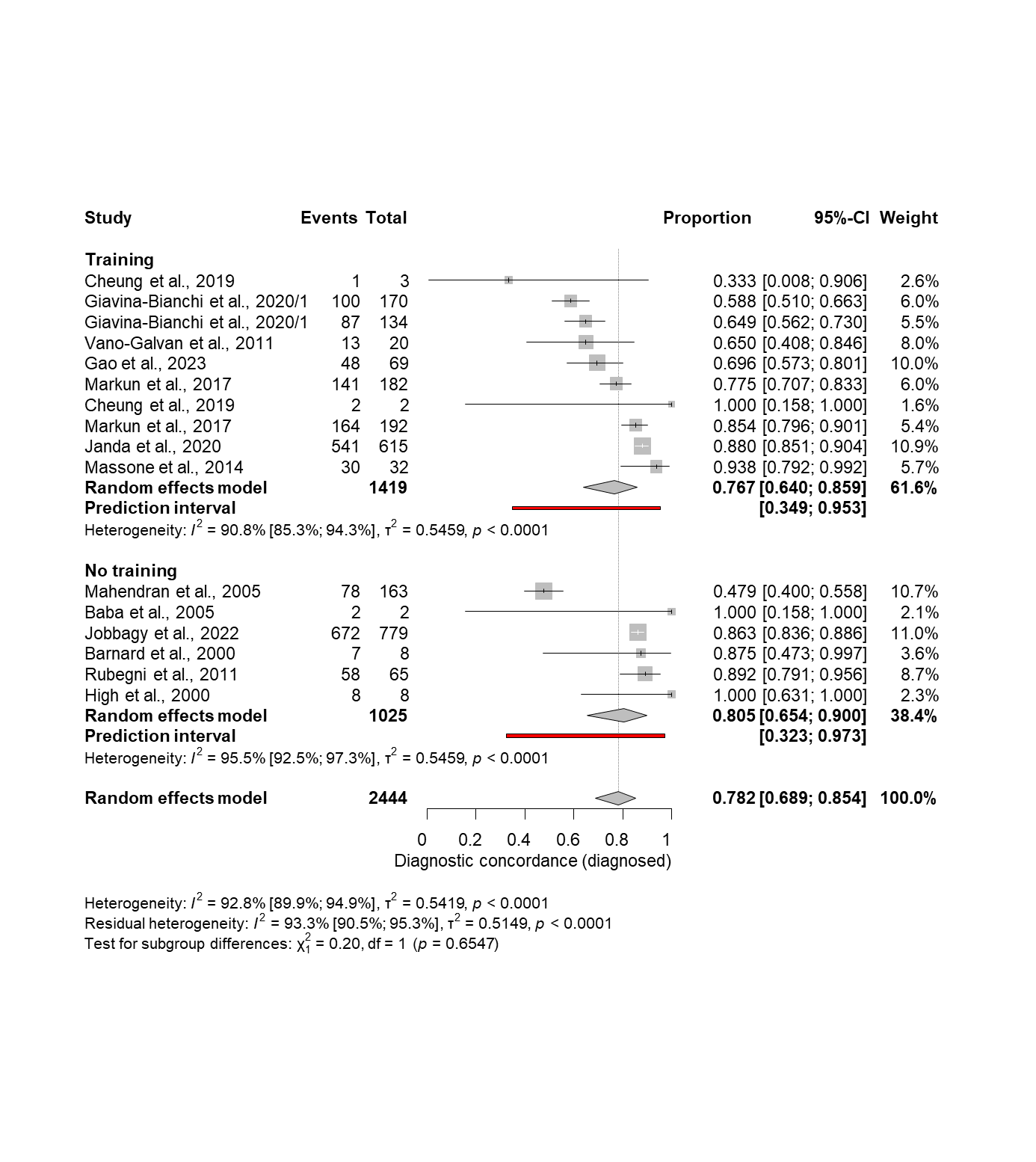


# Supplementary Figure S15: Forest plot comparing the diagnostic concordance between teledermatology providers and in-person dermatologists subgrouped by training for image acquisition in the “pigmented lesions” group, excluding undiagnosed cases from the analysis.


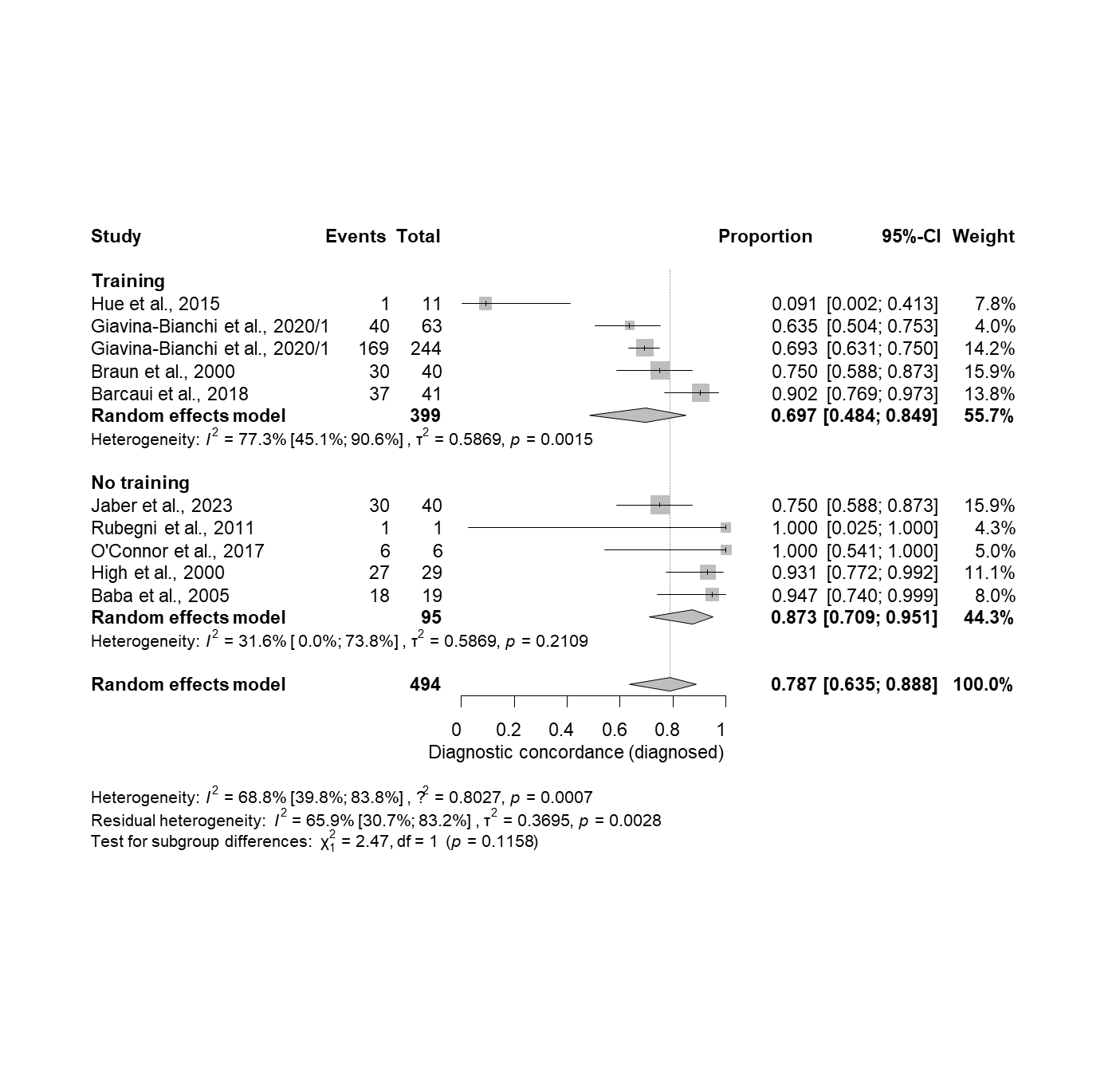


# Supplementary Figure S16: Forest plot comparing the diagnostic concordance between teledermatology providers and in-person dermatologists subgrouped by the comparator in the “all skin conditions” group, excluding undiagnosed cases from the analysis.


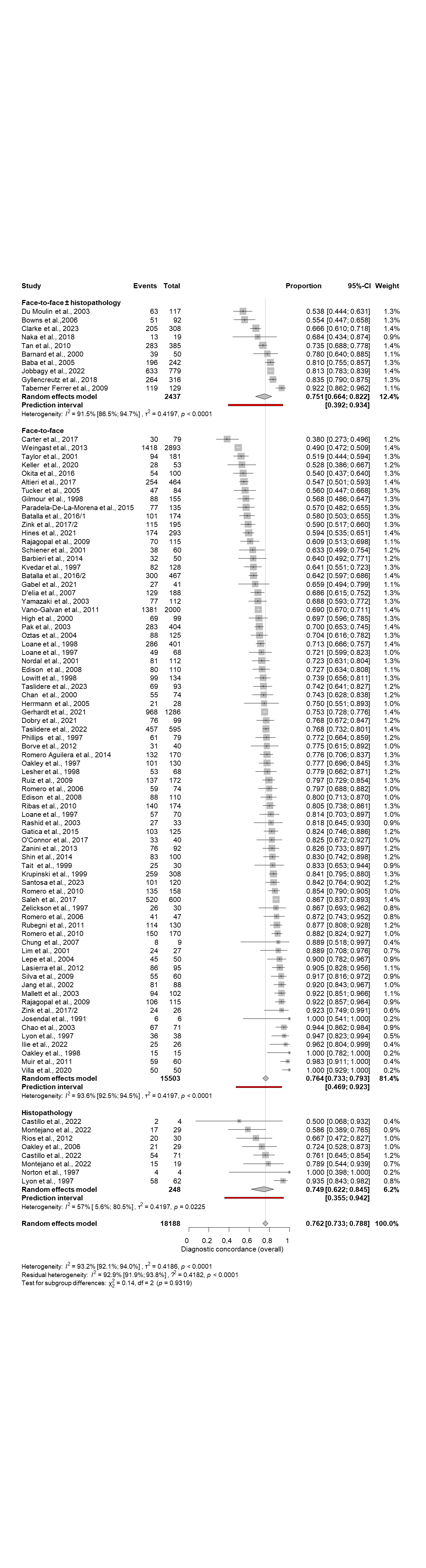


# Supplementary Figure S17: Forest plot comparing the diagnostic concordance between teledermatology providers and in-person dermatologists subgrouped by the comparator in the “skin cancer” group, excluding undiagnosed cases from the analysis.


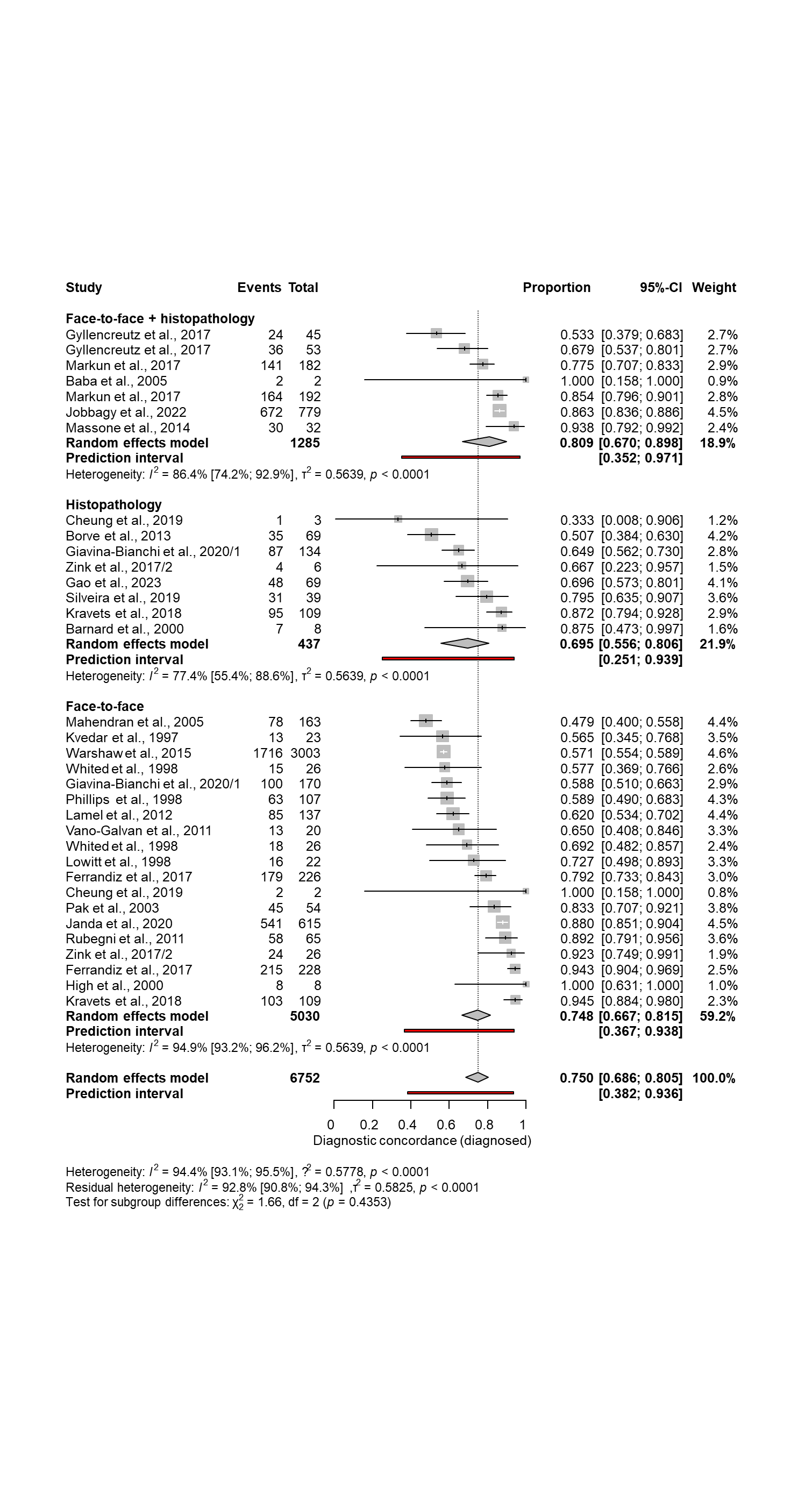


# Supplementary Figure S18: Forest plot comparing the diagnostic concordance between teledermatology providers and in-person dermatologists subgrouped by the comparator in the “pigmented lesions” group, excluding undiagnosed cases from the analysis.


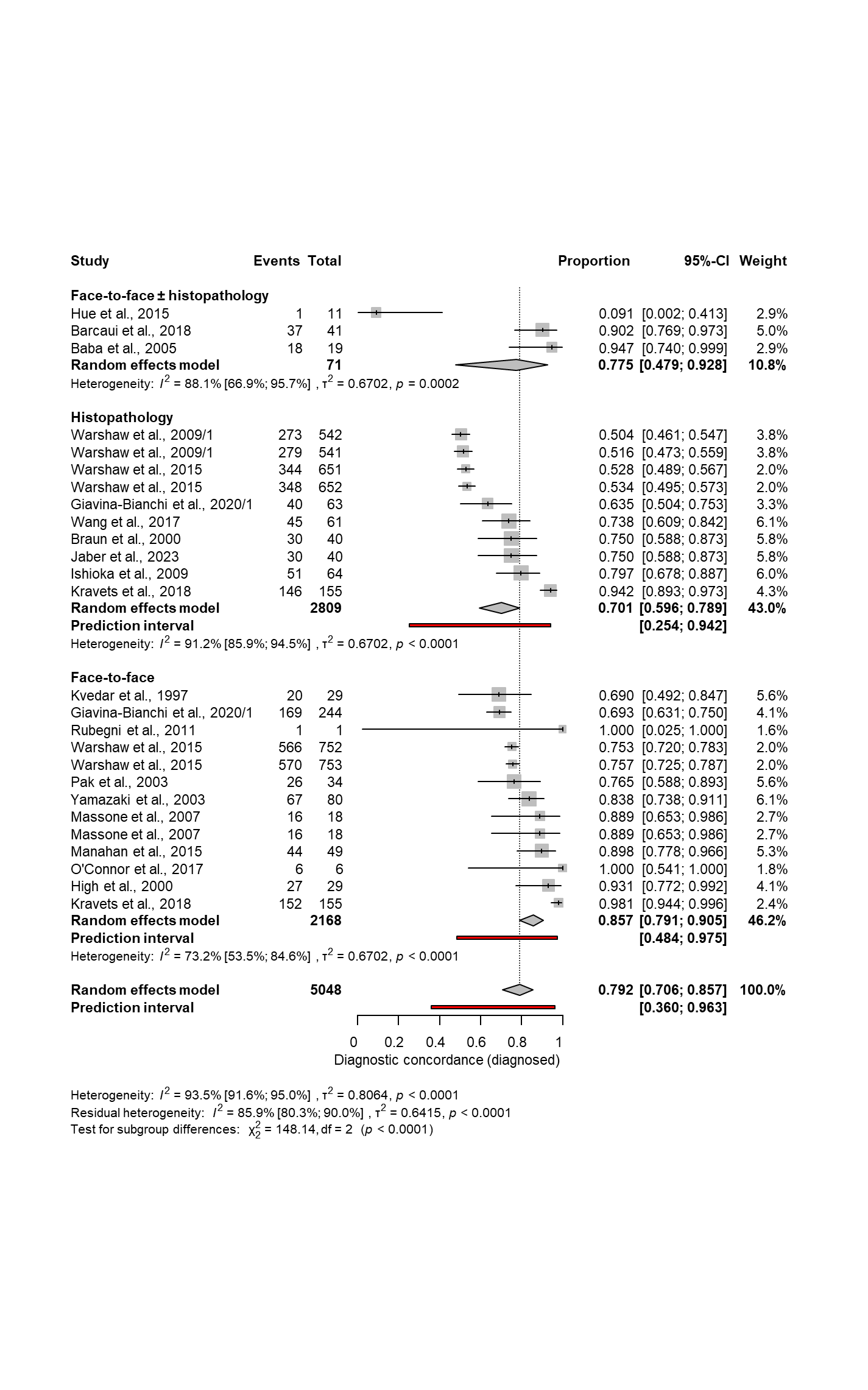


# Supplementary Figure S19: Forest plot comparing Cohen’s kappa between teledermatology providers and in-person dermatologists subgrouped by the communication platform in the “all skin conditions” group, excluding undiagnosed cases from the analysis.


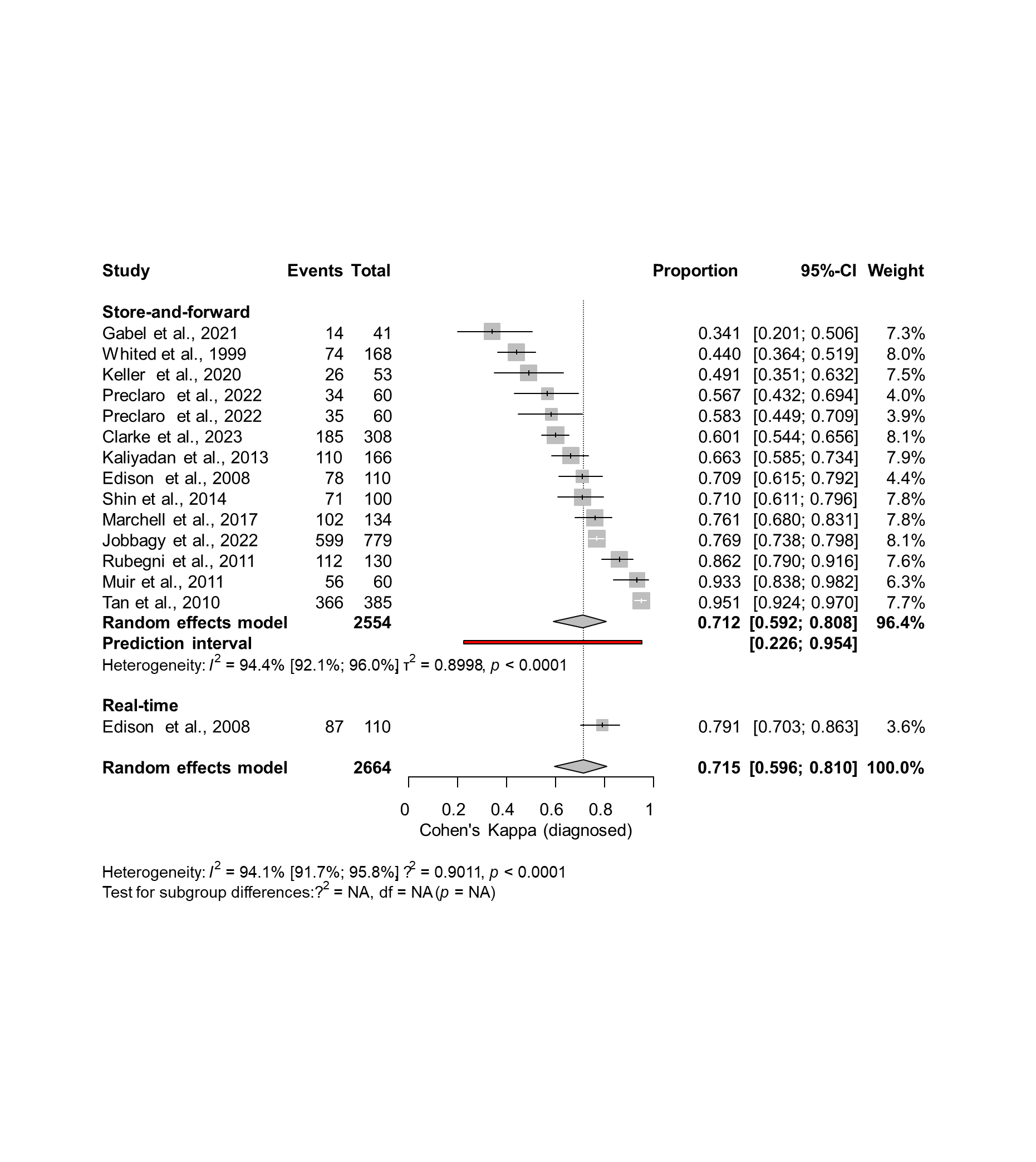


# Supplementary Figure S20: Forest plot comparing Cohen’s kappa between teledermatology providers and in-person dermatologists subgrouped by the communication platform in the “skin cancer” group, excluding undiagnosed cases from the analysis.


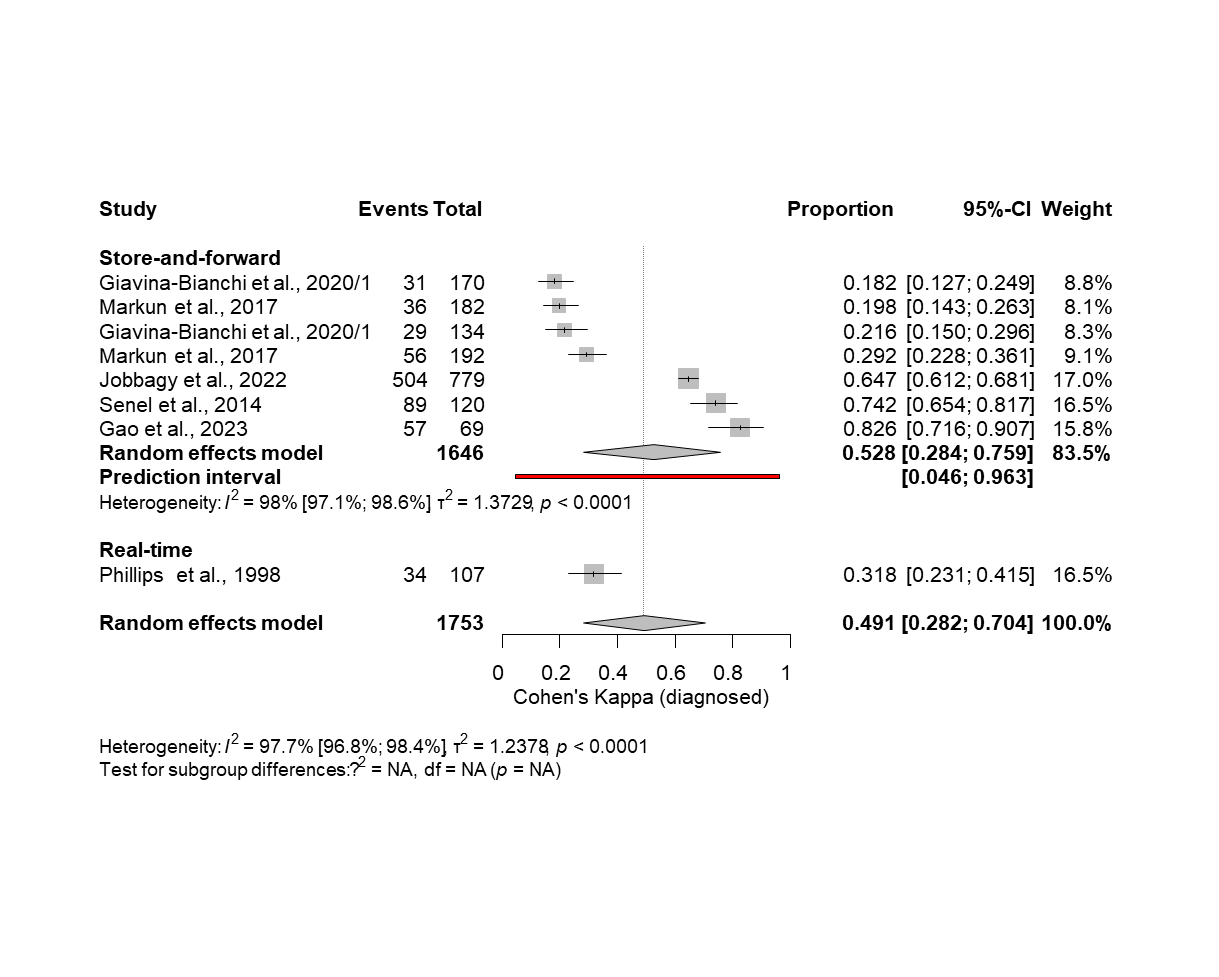


# Supplementary Figure S21: Forest plot comparing Cohen’s kappa between teledermatology providers and in-person dermatologists subgrouped by the communication platform in the “pigmented lesions” group, excluding undiagnosed cases from the analysis.


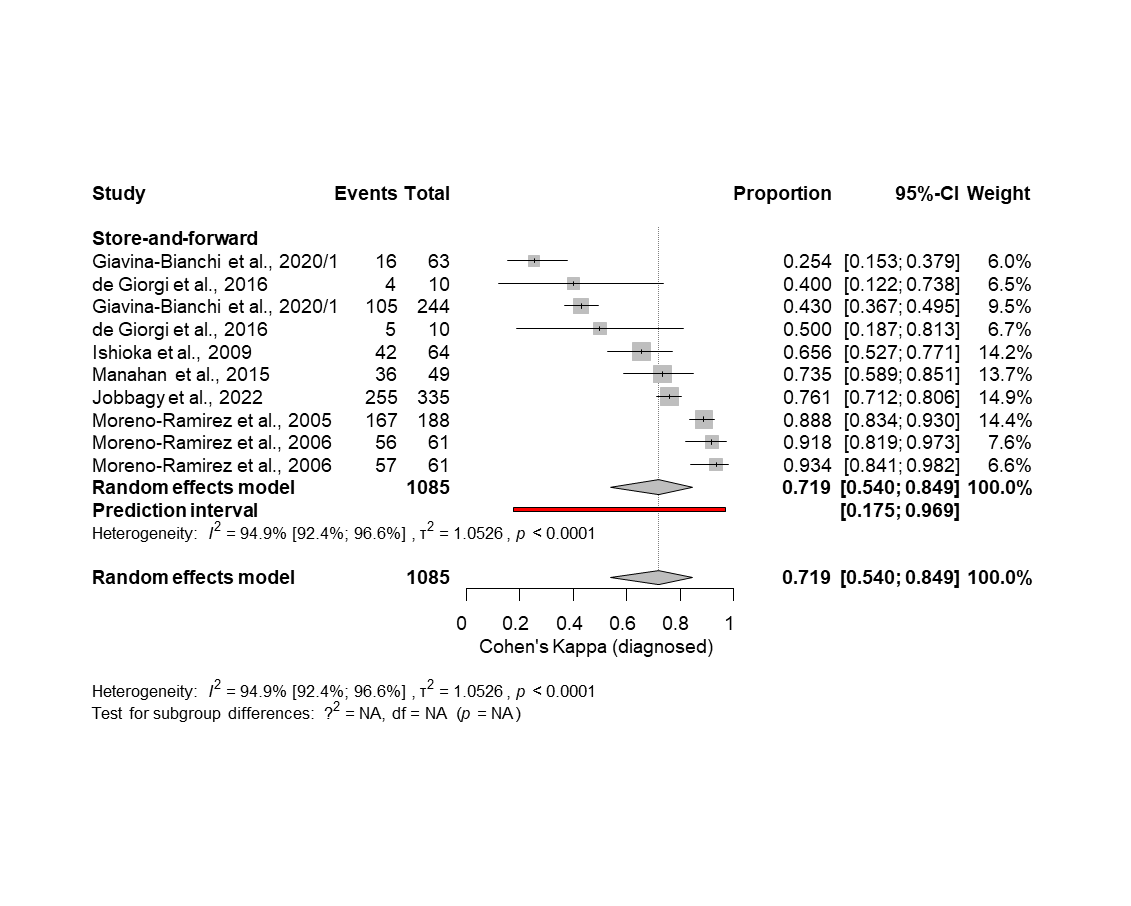


# Supplementary Figure S22: Forest plot comparing Cohen’s kappa between teledermatology providers and in-person dermatologists subgrouped by the communication type in the “all skin conditions” group, excluding undiagnosed cases from the analysis.


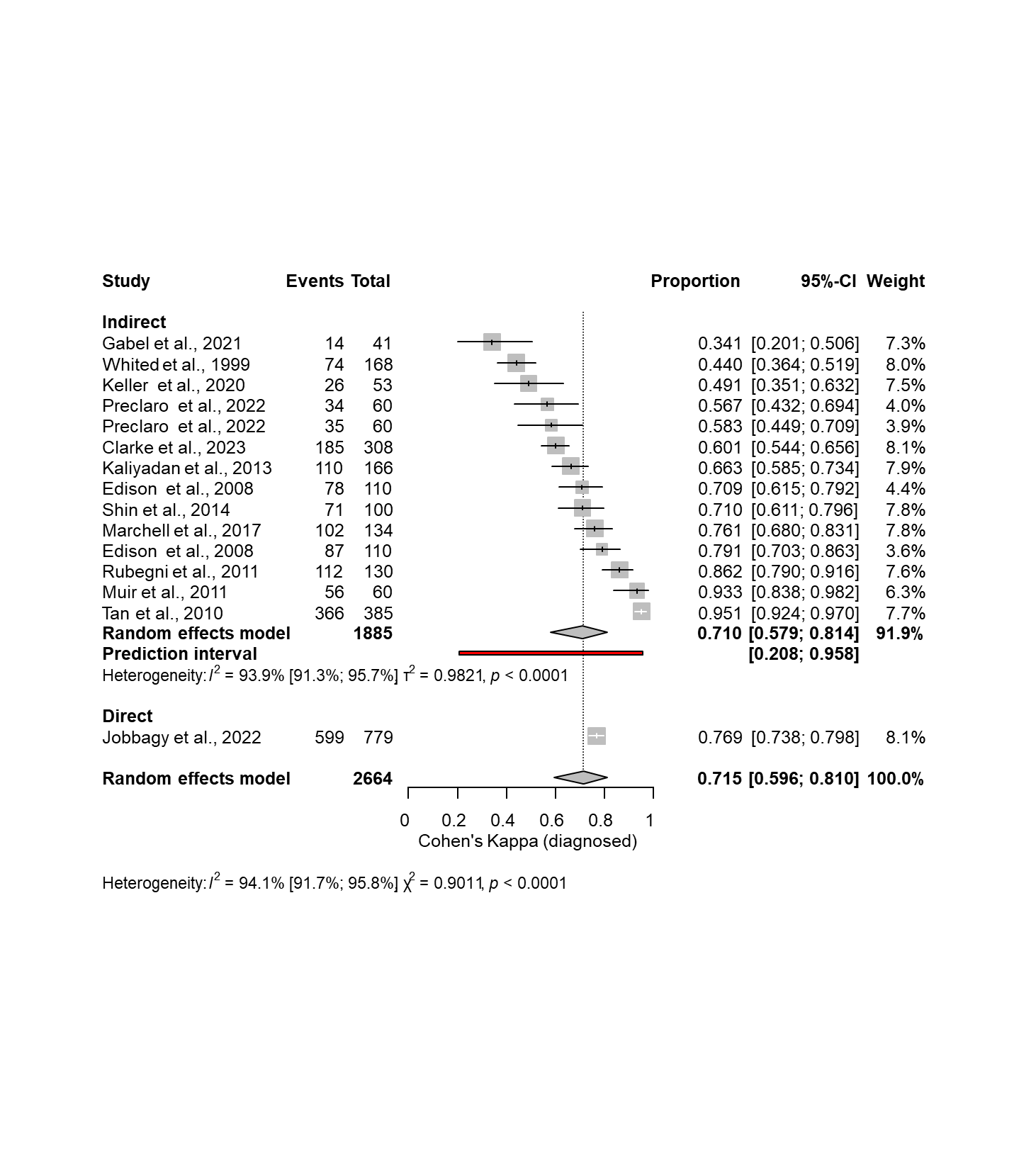


# Supplementary Figure S23: Forest plot comparing Cohen’s kappa between teledermatology providers and in-person dermatologists subgrouped by the communication type in the “skin cancer” group, excluding undiagnosed cases from the analysis.


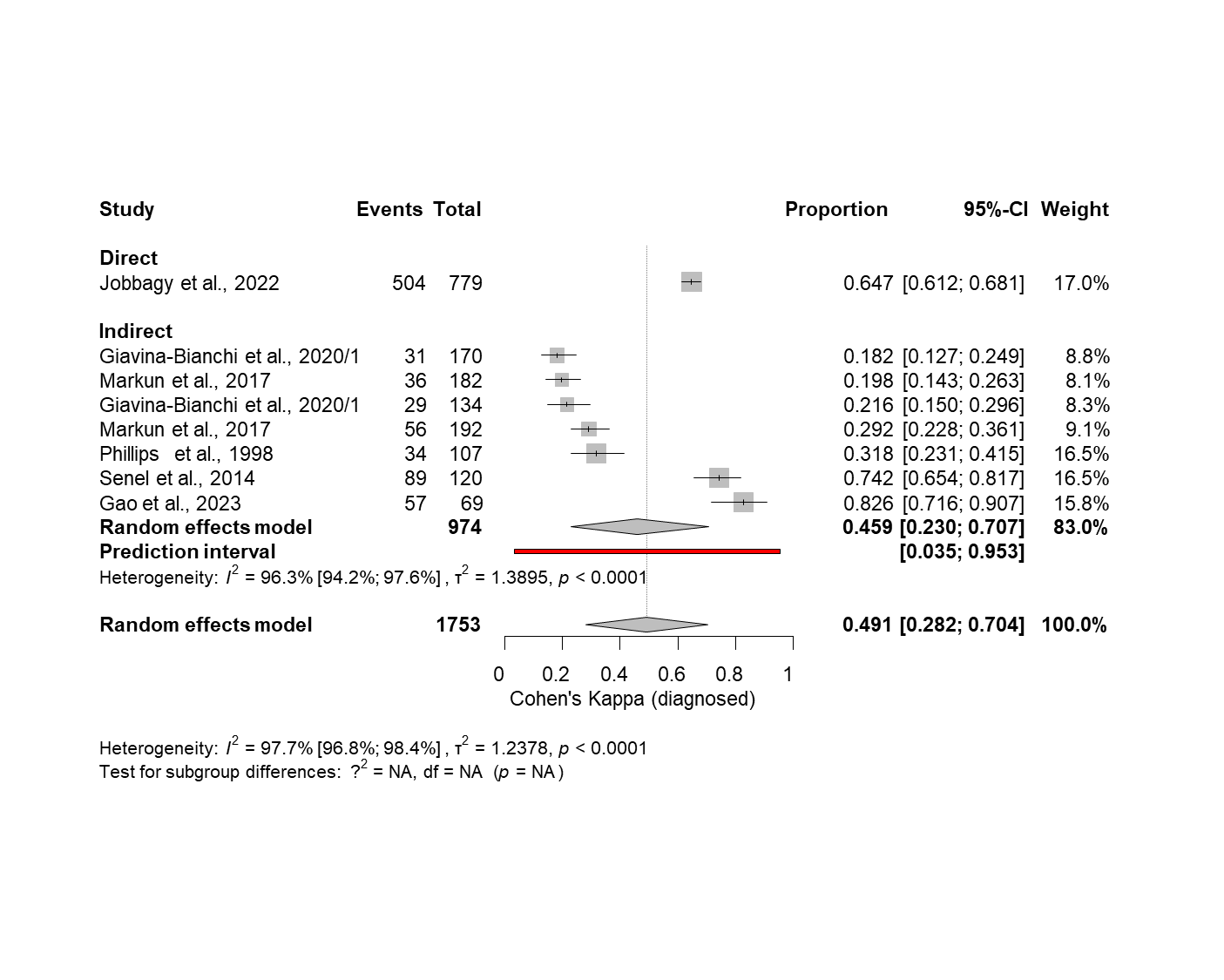


# Supplementary Figure S24: Forest plot comparing Cohen’s kappa between teledermatology providers and in-person dermatologists subgrouped by the communication type in the “pigmented lesions” group, excluding undiagnosed cases from the analysis.


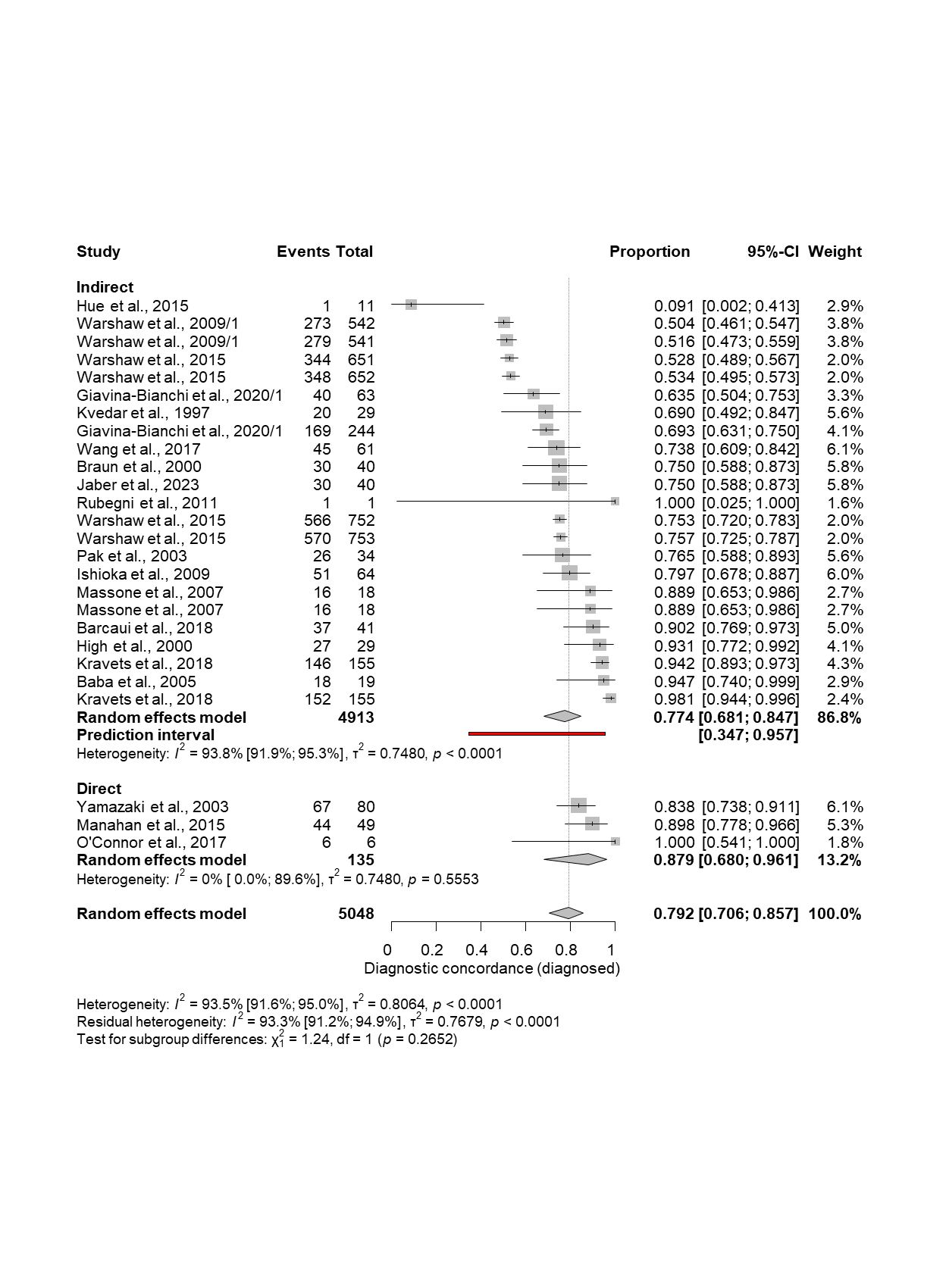


# Supplementary Figure S25: Forest plot comparing Cohen’s kappa between teledermatology providers and in-person dermatologists subgrouped by the use of dermoscopy in the “all skin conditions” group, excluding undiagnosed cases from the analysis.


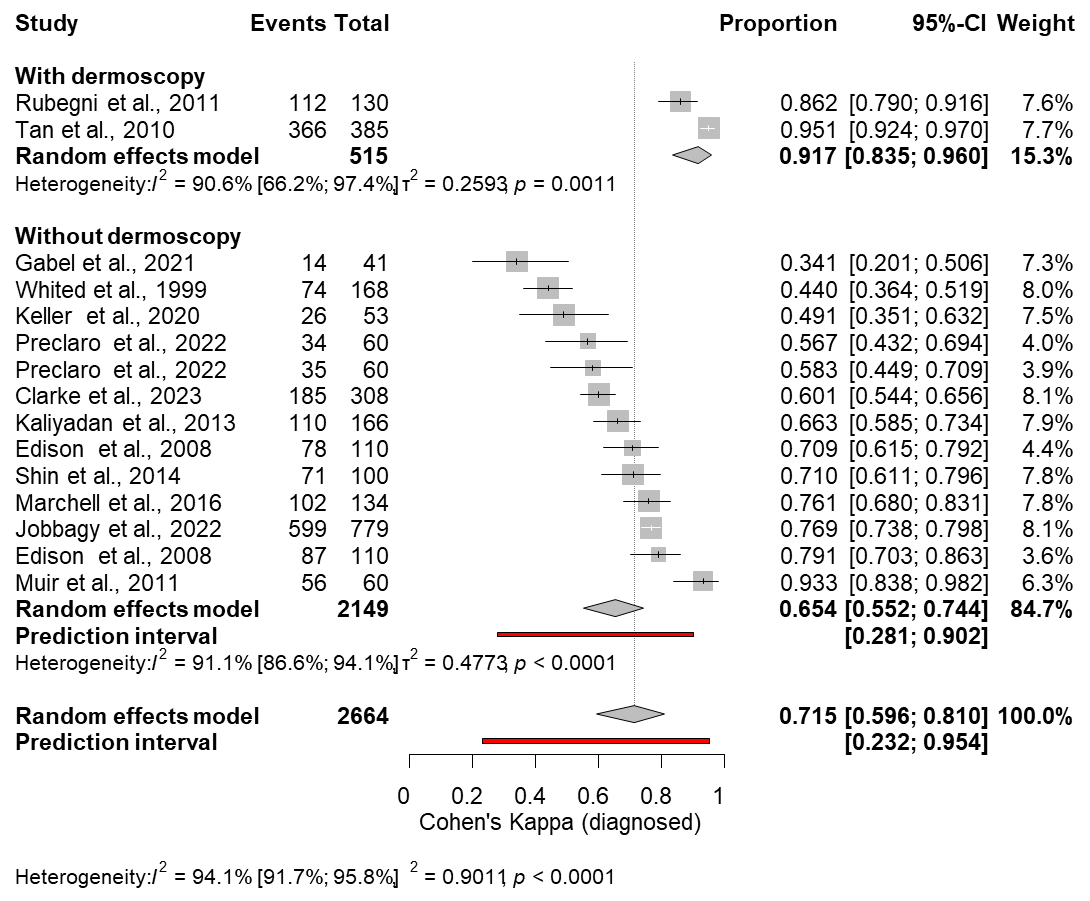


# Supplementary Figure S26: Forest plot comparing Cohen’s kappa between teledermatology providers and in-person dermatologists subgrouped by the use of dermoscopy in the “skin cancer” group, excluding undiagnosed cases from the analysis.


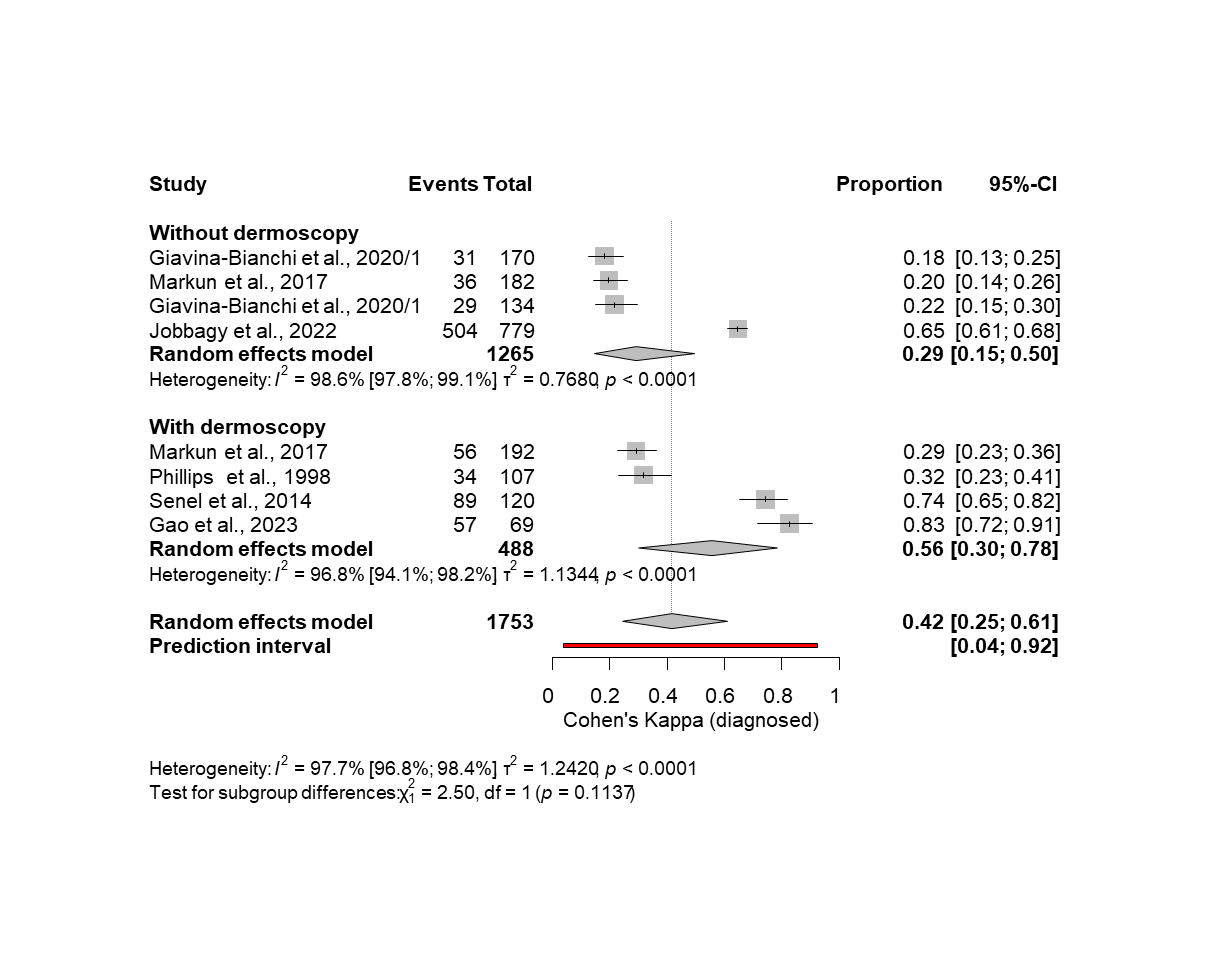


# Supplementary Figure S27: Forest plot comparing Cohen’s kappa between teledermatology providers and in-person dermatologists subgrouped by the use of dermoscopy in the “pigmented lesions” group, excluding undiagnosed cases from the analysis.


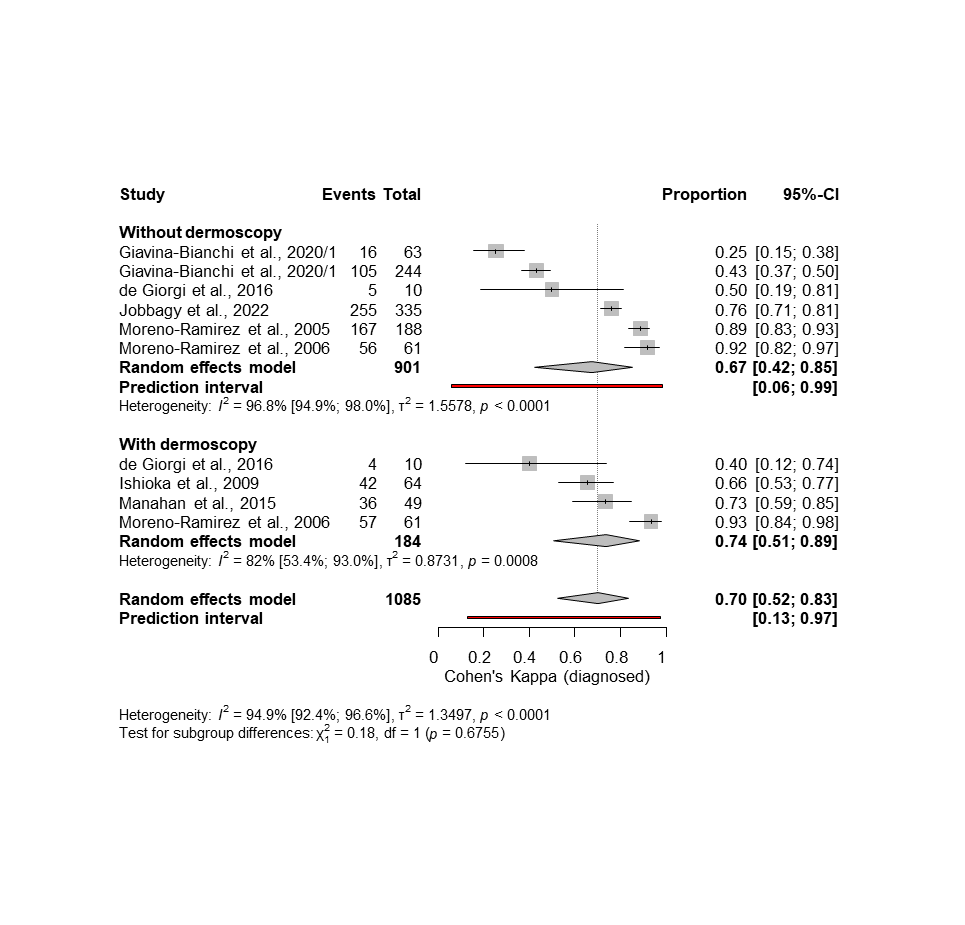


# Supplementary Figure S28: Forest plot comparing Cohen’s kappa between teledermatology providers and in-person dermatologists subgrouped by the photography device in the “all skin conditions” group, excluding undiagnosed cases from the analysis.


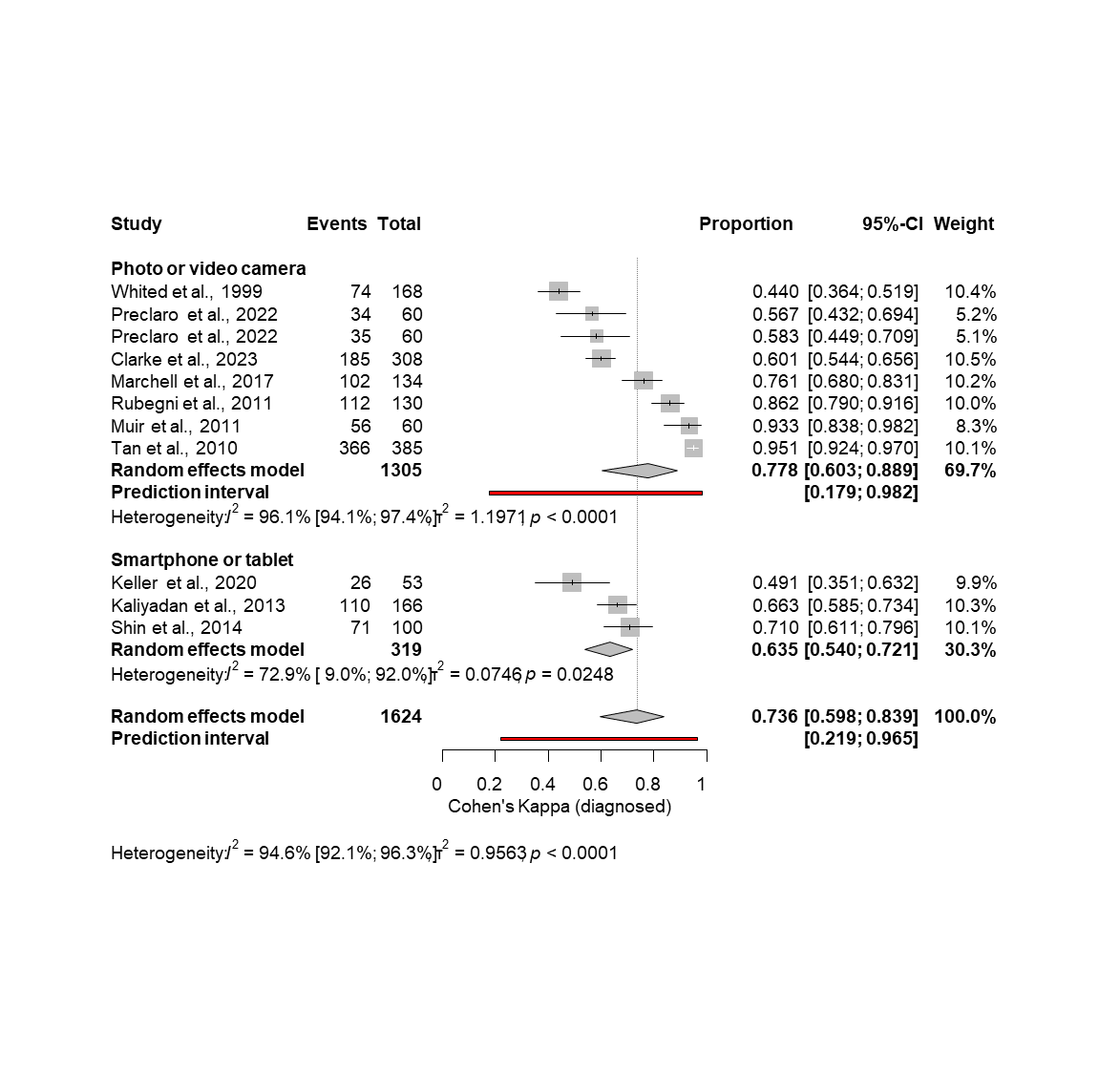


# Supplementary Figure S29: Forest plot comparing Cohen’s kappa between teledermatology providers and in-person dermatologists subgrouped by the photography device in the “skin cancer” group, excluding undiagnosed cases from the analysis.


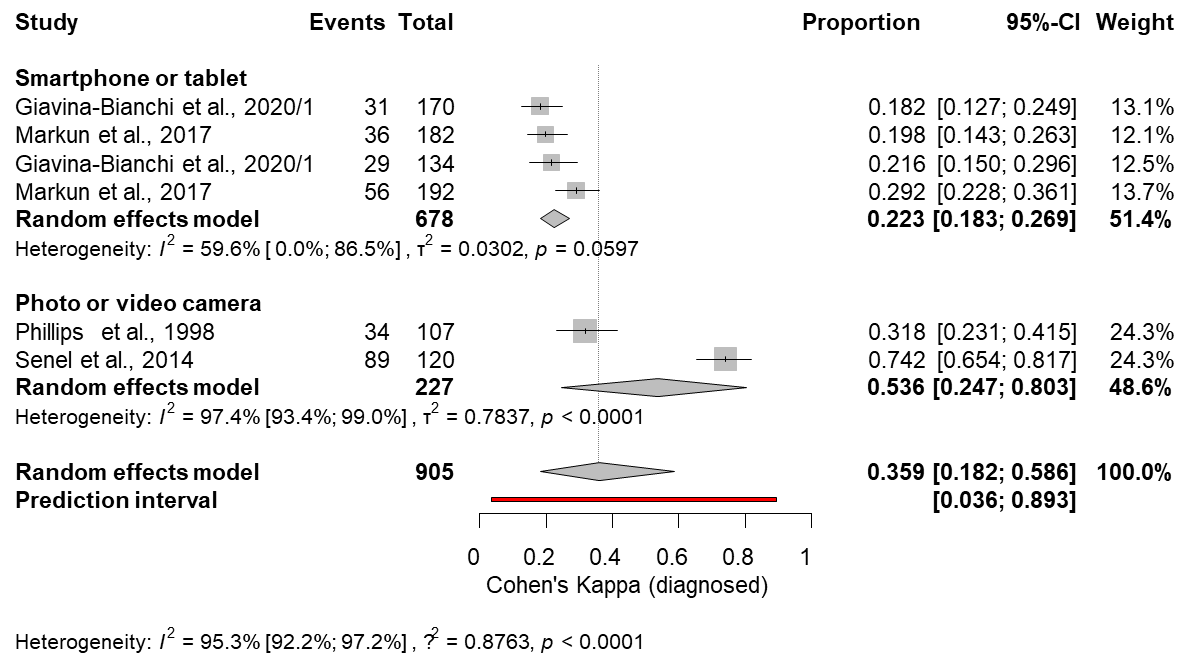


# Supplementary Figure S30: Forest plot comparing the Cohen’s kappa between teledermatology providers and in-person dermatologists subgrouped by the photography device in the “pigmented lesions” group, excluding undiagnosed cases from the analysis.


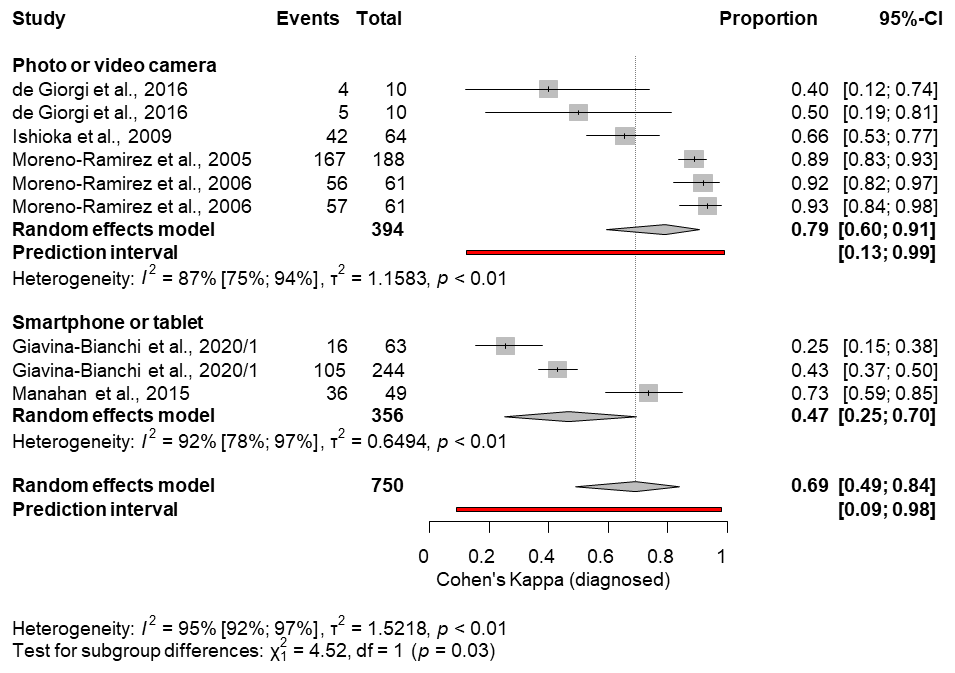


# Supplementary Figure S31: Forest plot comparing Cohen’s kappa between teledermatology providers and in-person dermatologists subgrouped by training for image acquisition in the “all skin conditions” group, excluding undiagnosed cases from the analysis.


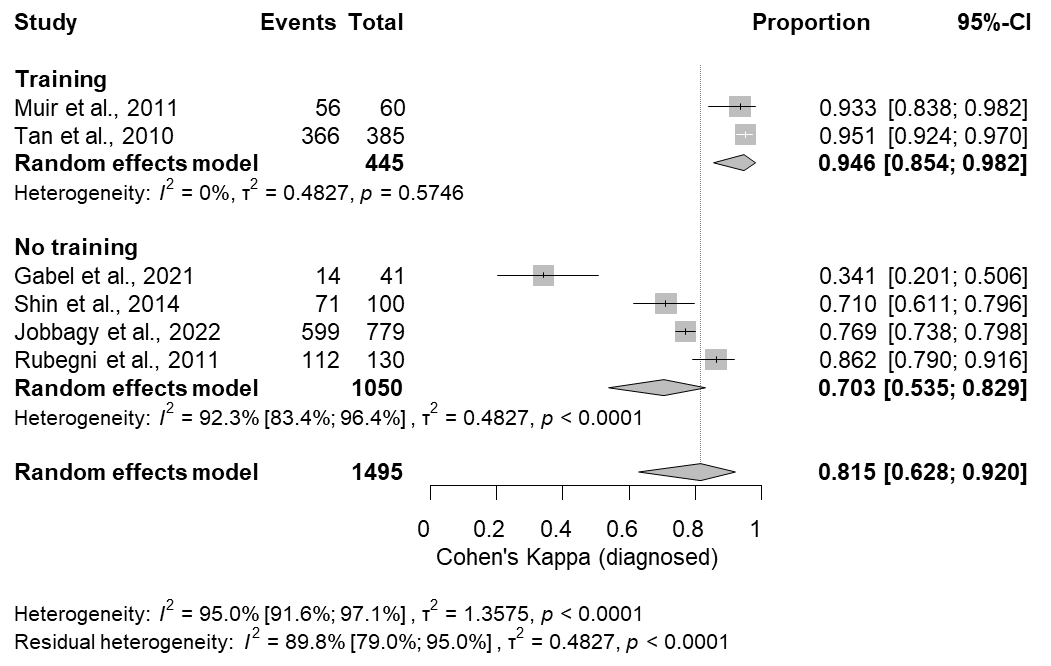


# Supplementary Figure S32: Forest plot comparing Cohen’s kappa between teledermatology providers and in-person dermatologists subgrouped by training for image acquisition in the “skin cancer” group, excluding undiagnosed cases from the analysis.


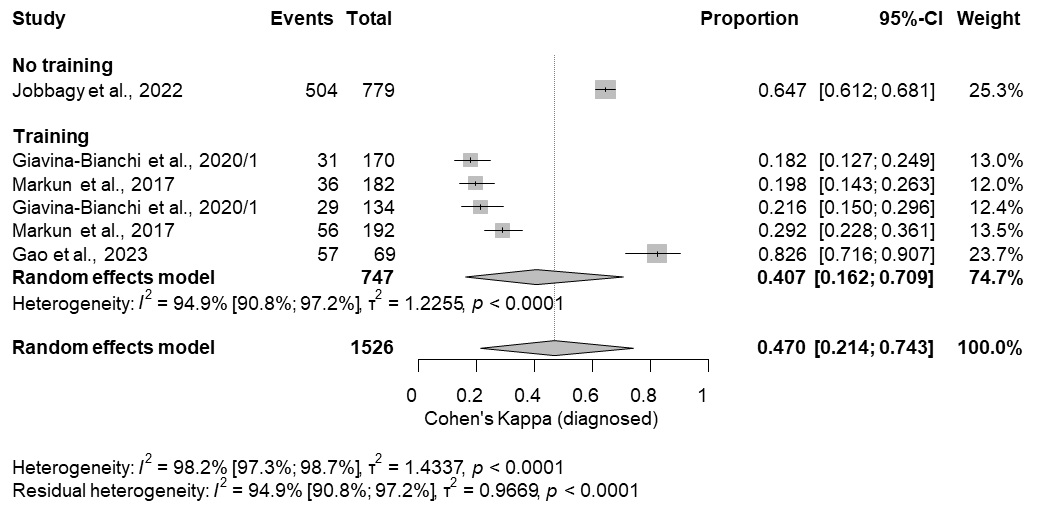


# Supplementary Figure S33: Forest plot comparing Cohen’s kappa between teledermatology providers and in-person dermatologists subgrouped by training for image acquisition in the “pigmented lesions” group, excluding undiagnosed cases from the analysis.


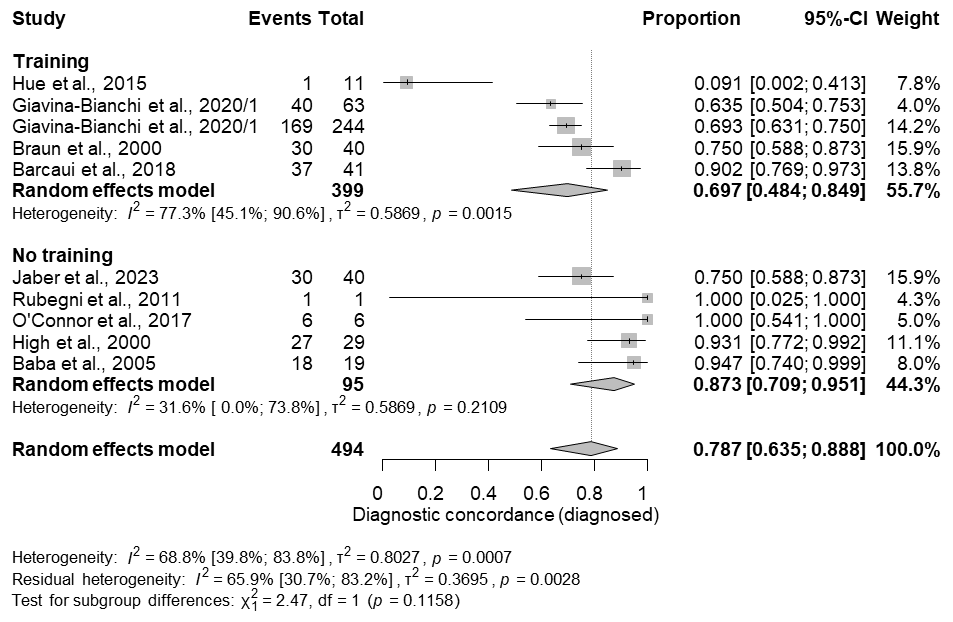


# Supplementary Figure S34: Forest plot comparing Cohen’s kappa between teledermatology providers and in-person dermatologists subgrouped by the comparator in the “all skin conditions” group, excluding undiagnosed cases from the analysis.


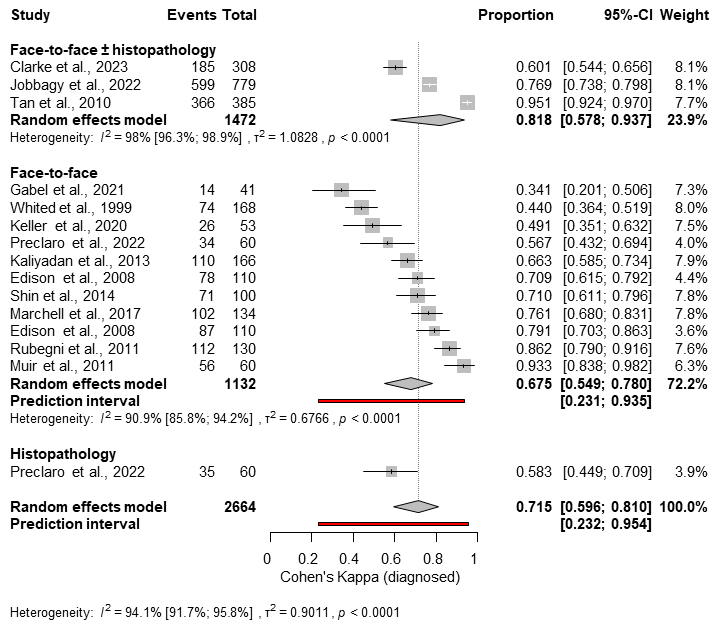


# Supplementary Figure 35: Forest plot comparing Cohen’s kappa in the pairwise comparison of “face-to-face ±histopathology” and “face-to-face” comparators in the “all skin conditions” group, excluding undiagnosed cases in the analysis.


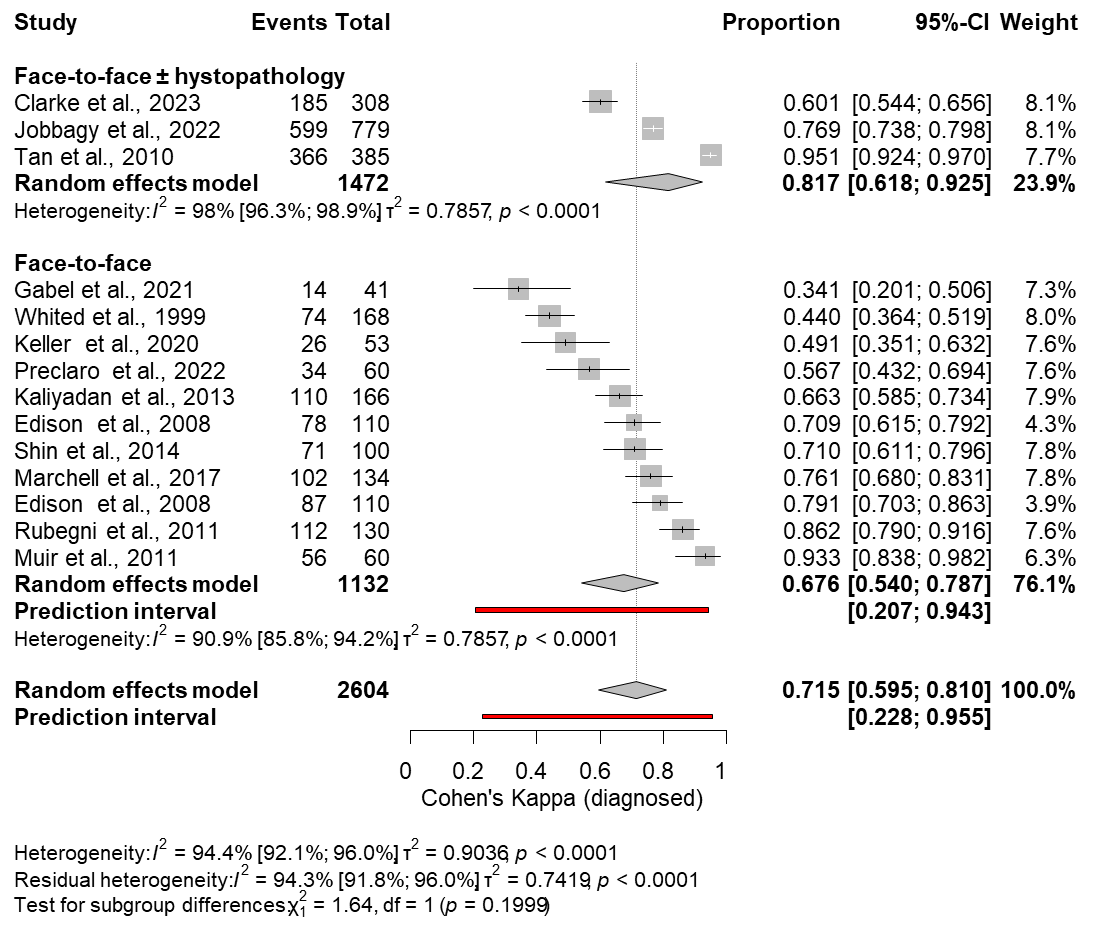


# Supplementary Figure S36: Forest plot comparing Cohen’s kappa between teledermatology providers and in-person dermatologists subgrouped by the comparator in the “skin cancer” group, excluding undiagnosed cases from the analysis.


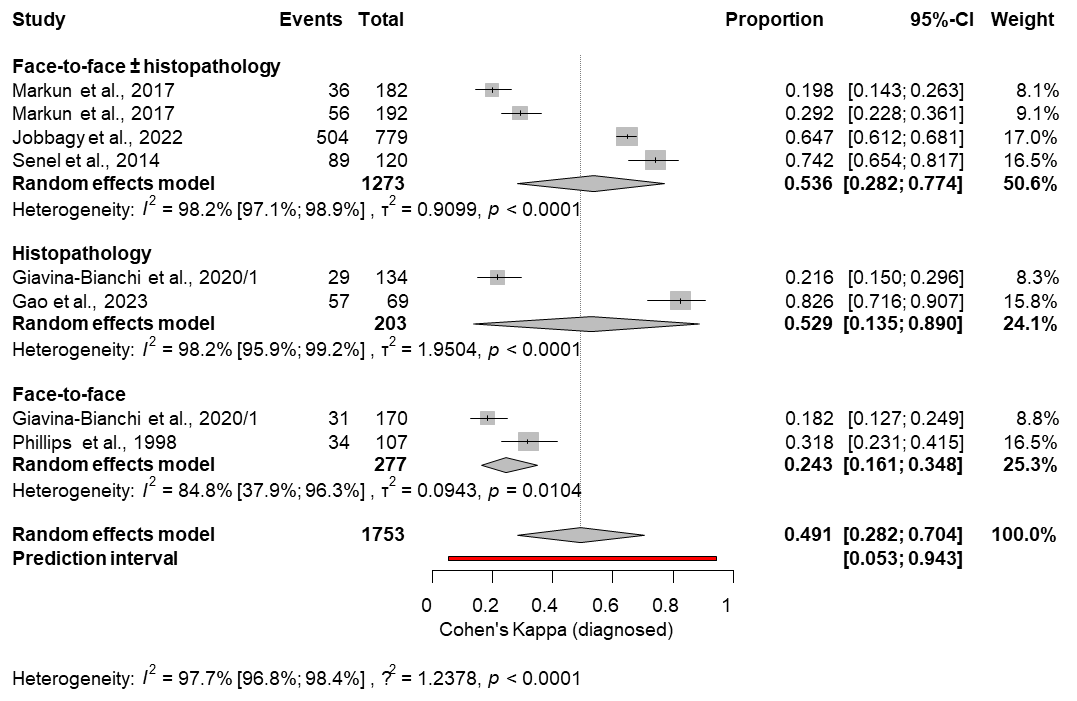


# Supplementary Figure S37: Forest plot comparing Cohen’s kappa between teledermatology providers and in-person dermatologists subgrouped by the comparator in the “pigmented lesions” group, excluding undiagnosed cases from the analysis.


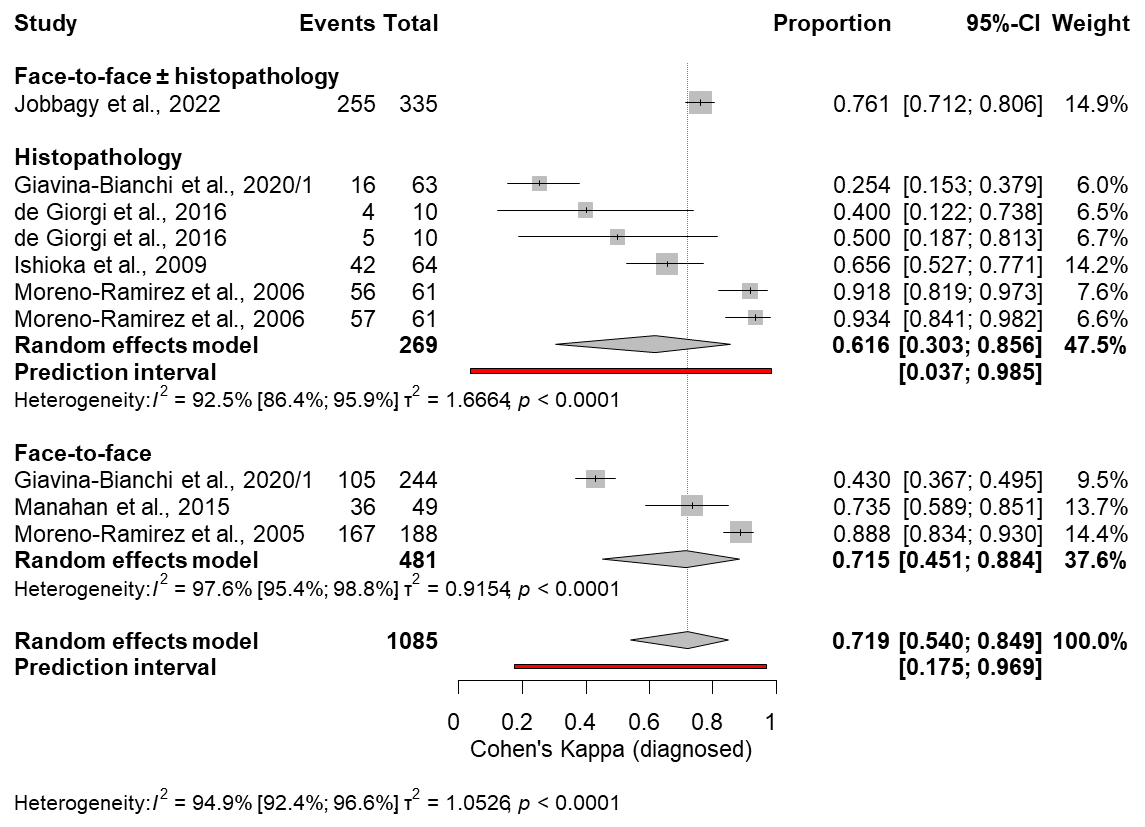


# Supplementary Figure S38: Forest plot comparing Cohen’s kappa in the pairwise comparison of “face-to-face” and “histopathology” comparators in the “pigmented lesions” group, excluding undiagnosed cases in the analysis.


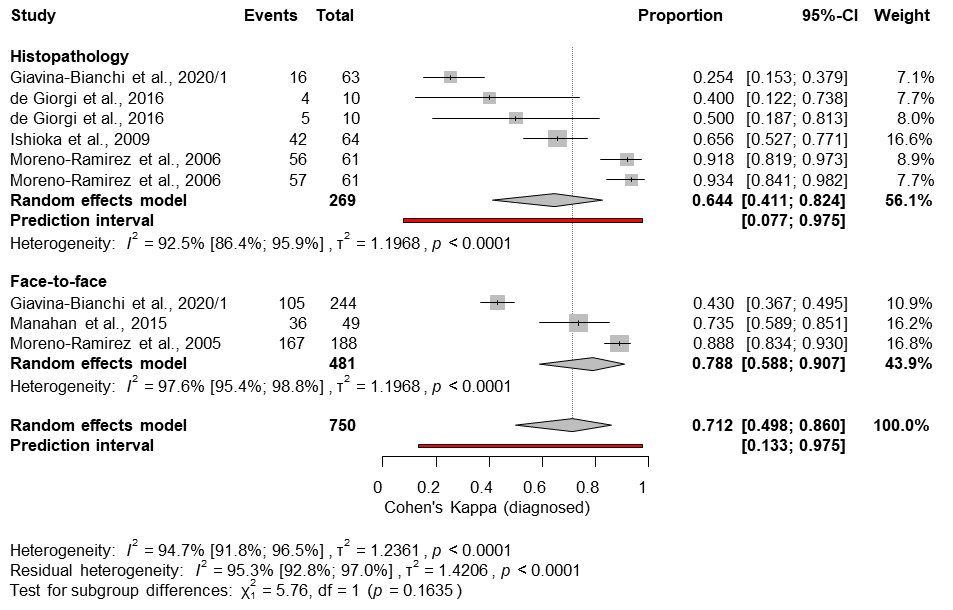


# Supplementary Figure S39: Forest plot for the sensitivity of teledermatology in the “skin cancer” group, excluding undiagnosed cases from the analysis.

# Supplementary Figure S40: Forest plot for the specificity of teledermatology in the “skin cancer” group, excluding undiagnosed cases from the analysis.

# Supplementary Figure S41: Forest plot for the sensitivity of teledermatology in the “pigmented lesions” group, excluding undiagnosed cases from the analysis.

# Supplementary Figure S42: Forest plot for the specificity of teledermatology in the “pigmented lesions” group, excluding undiagnosed cases from the analysis.

# Supplementary Figure S43: Forest plot comparing the diagnostic concordance between teledermatology providers and in-person dermatologists subgrouped by the communication platform in the “all skin conditions” group, including undiagnosed cases in the analysis.


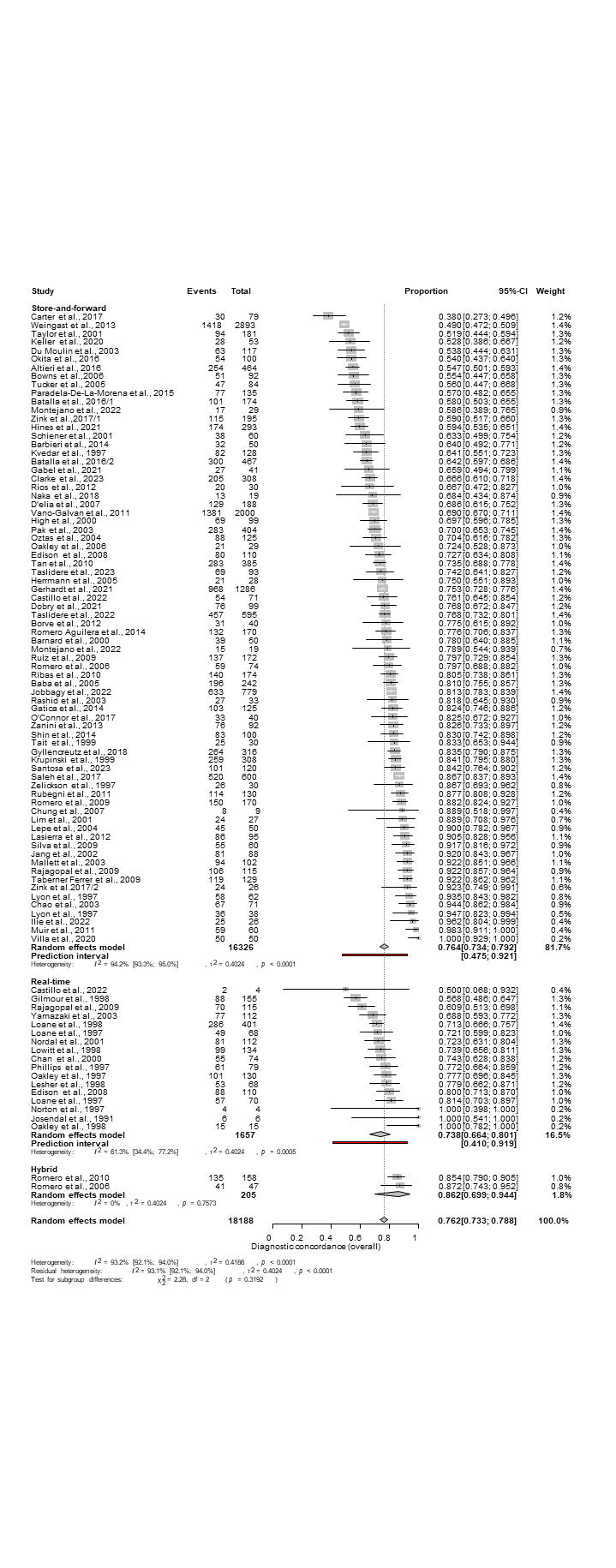


# Supplementary Figure S44: Forest plot comparing the diagnostic concordance between teledermatology providers and in-person dermatologists subgrouped by the communication platform in the “skin cancer” group, including undiagnosed cases in the analysis.


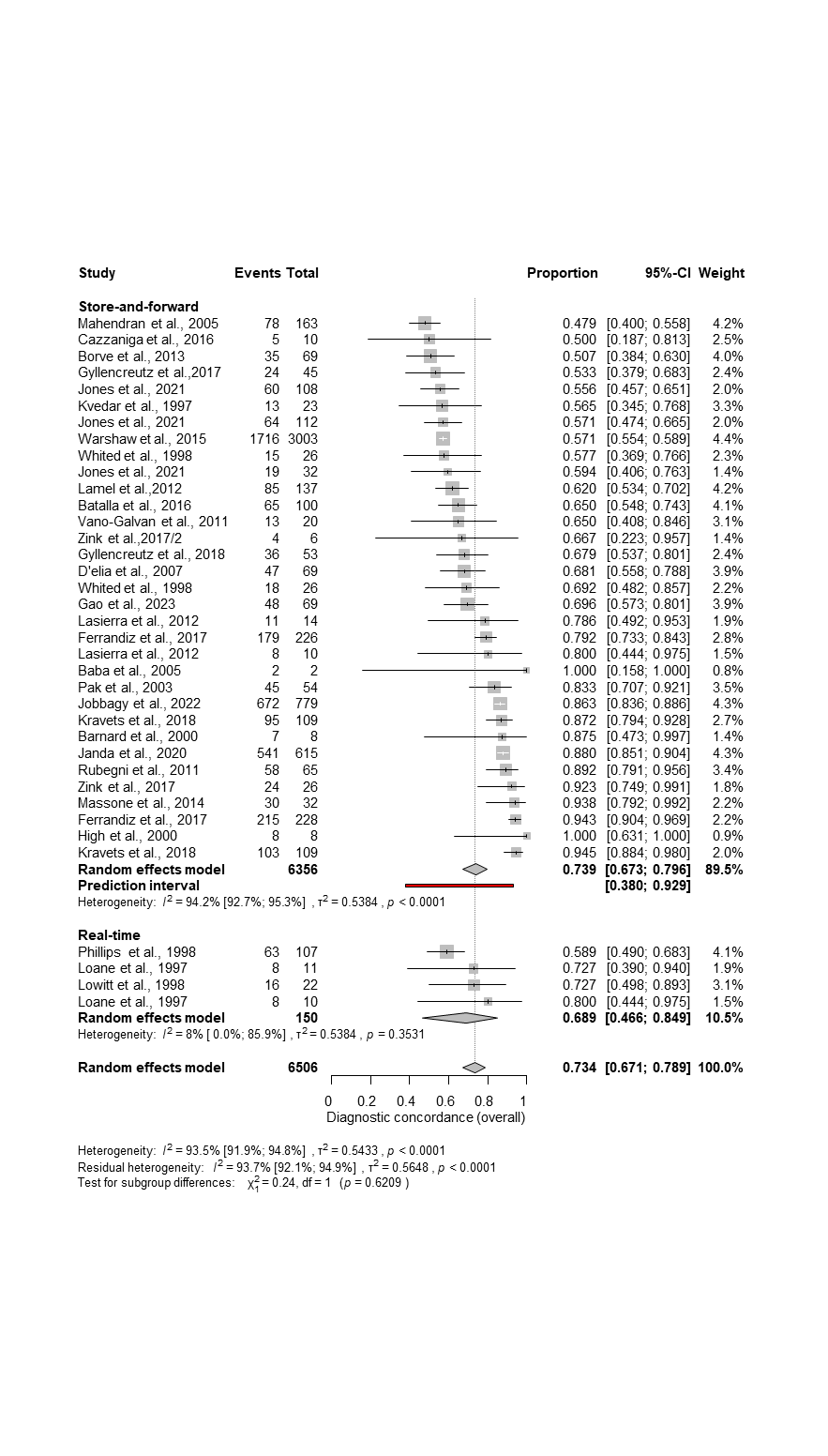


# Supplementary Figure S45: Forest plot comparing the diagnostic concordance between teledermatology providers and in-person dermatologists subgrouped by the communication platform in the “pigmented lesions” group, including undiagnosed cases in the analysis.


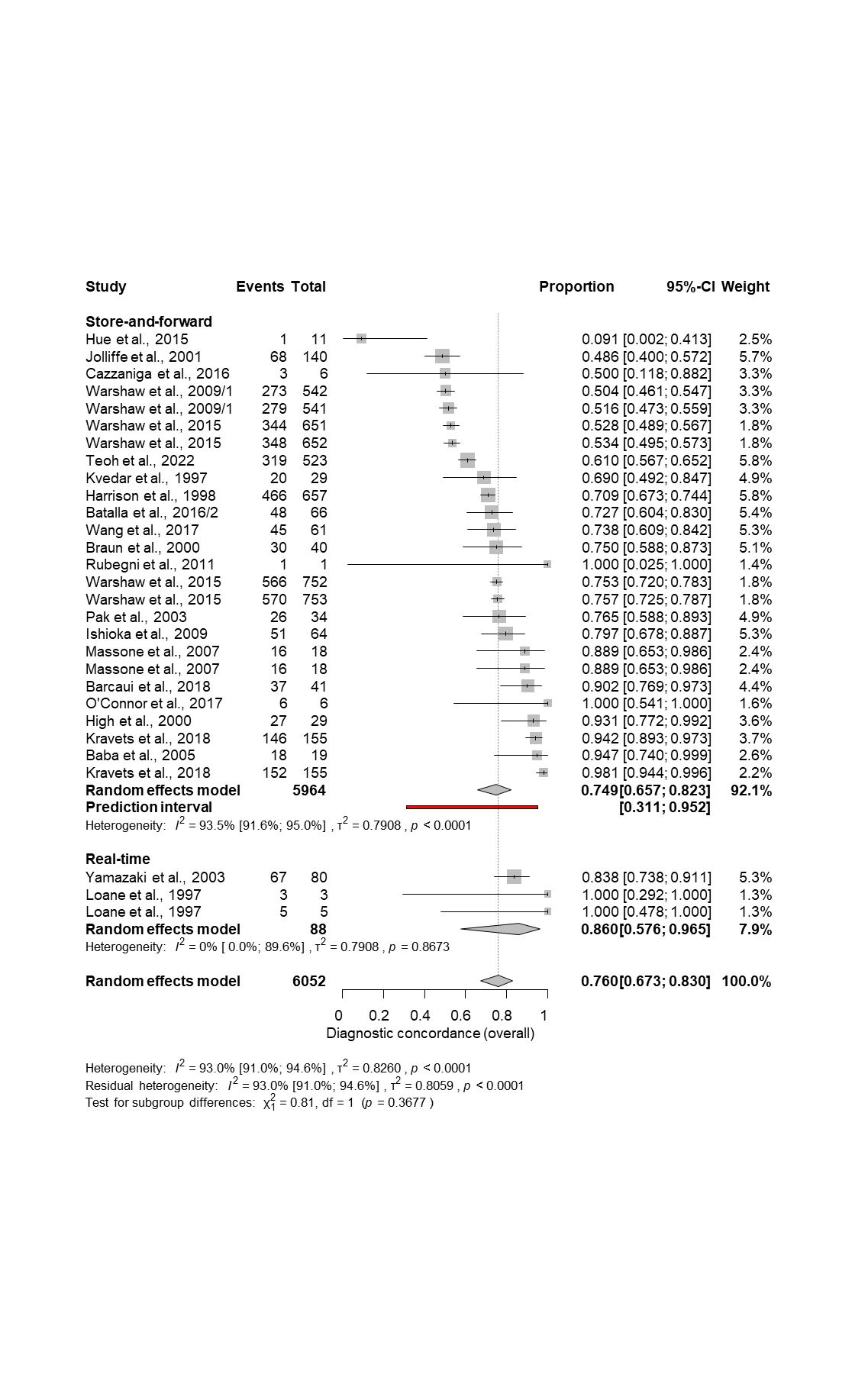


# Supplementary Figure S46: Forest plot comparing the diagnostic concordance between teledermatology providers and in-person dermatologists subgrouped by the communication type in the “all skin conditions” group, including undiagnosed cases in the analysis.


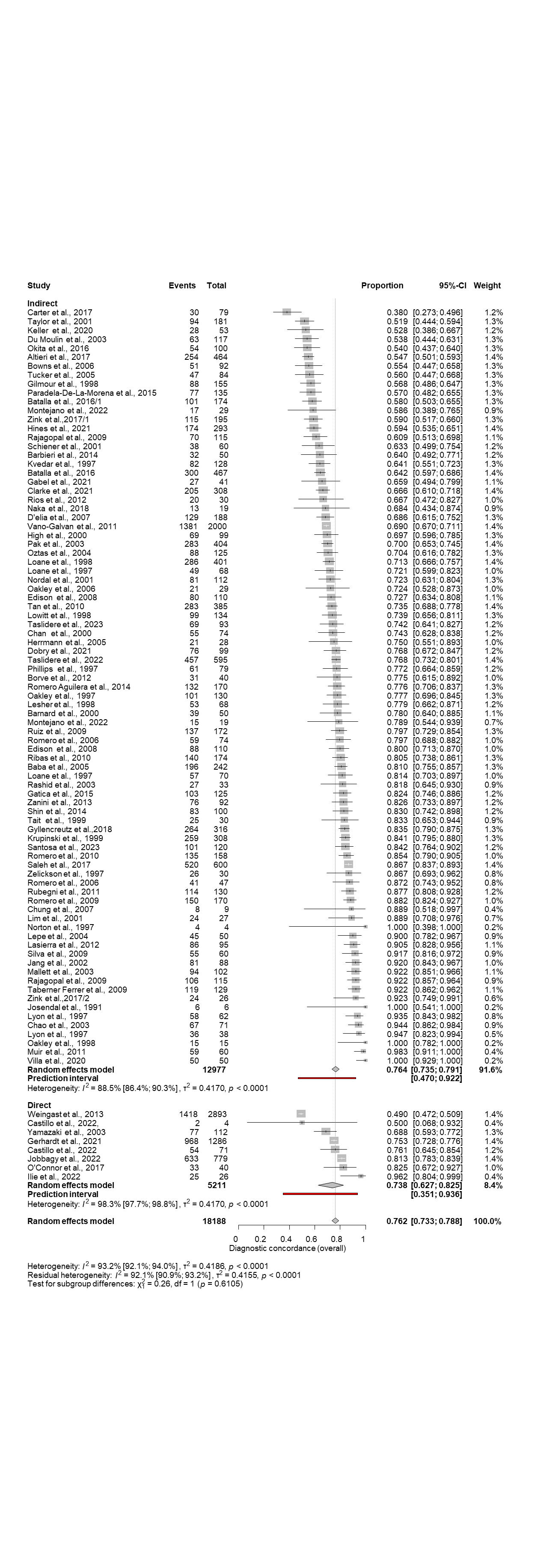


# Supplementary Figure S47: Forest plot comparing the diagnostic concordance between teledermatology providers and in-person dermatologists subgrouped by the communication type in the “skin cancer” group, including undiagnosed cases in the analysis.


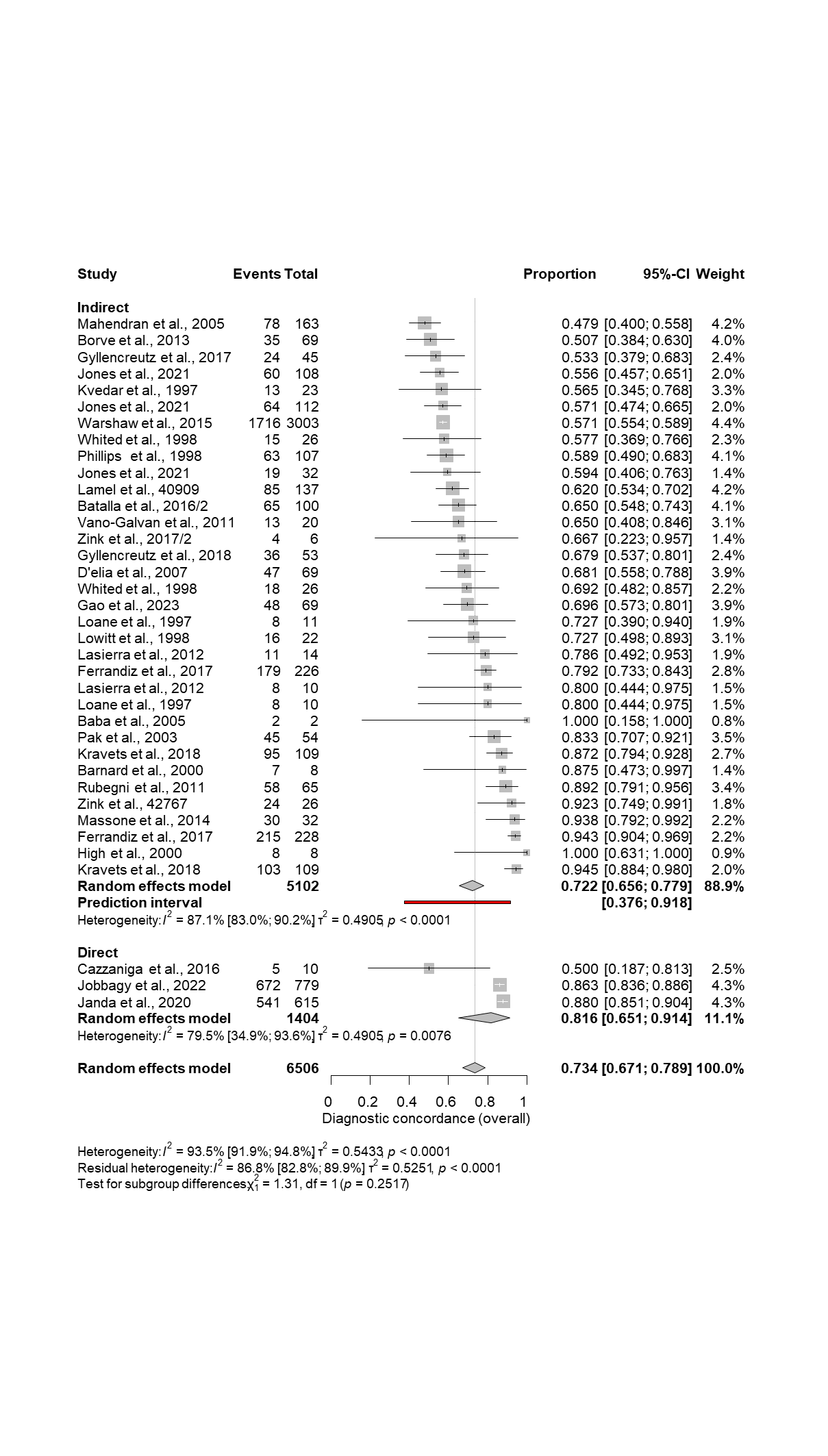


# Supplementary Figure S48: Forest plot comparing the diagnostic concordance between teledermatology providers and in-person dermatologists subgrouped by the communication type in the “pigmented lesions” group, including undiagnosed cases in the analysis.


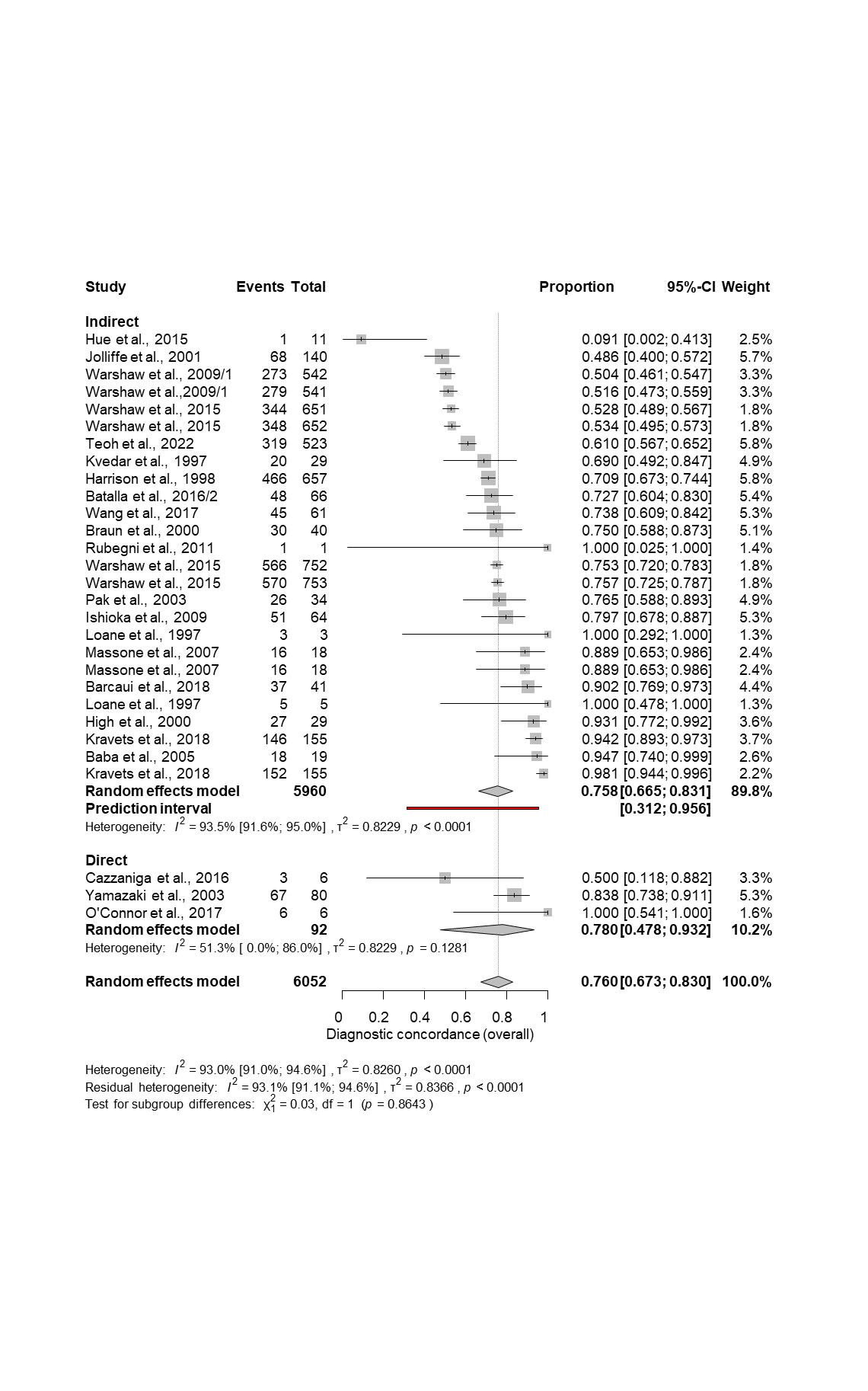


# Supplementary Figure S49: Forest plot comparing the diagnostic concordance between teledermatology providers and in-person dermatologists subgrouped by the use of dermoscopy in the “all skin conditions” group, including undiagnosed cases in the analysis.


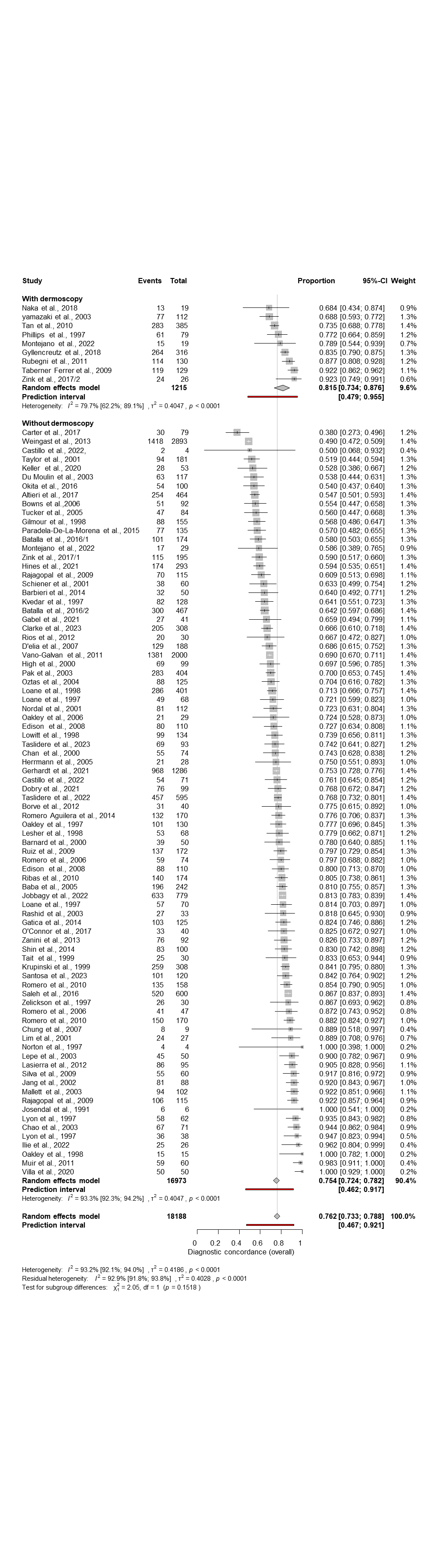


# Supplementary Figure S50: Forest plot comparing the diagnostic concordance between teledermatology providers and in-person dermatologists subgrouped by the use of dermoscopy in the “skin cancer” group, including undiagnosed cases in the analysis.


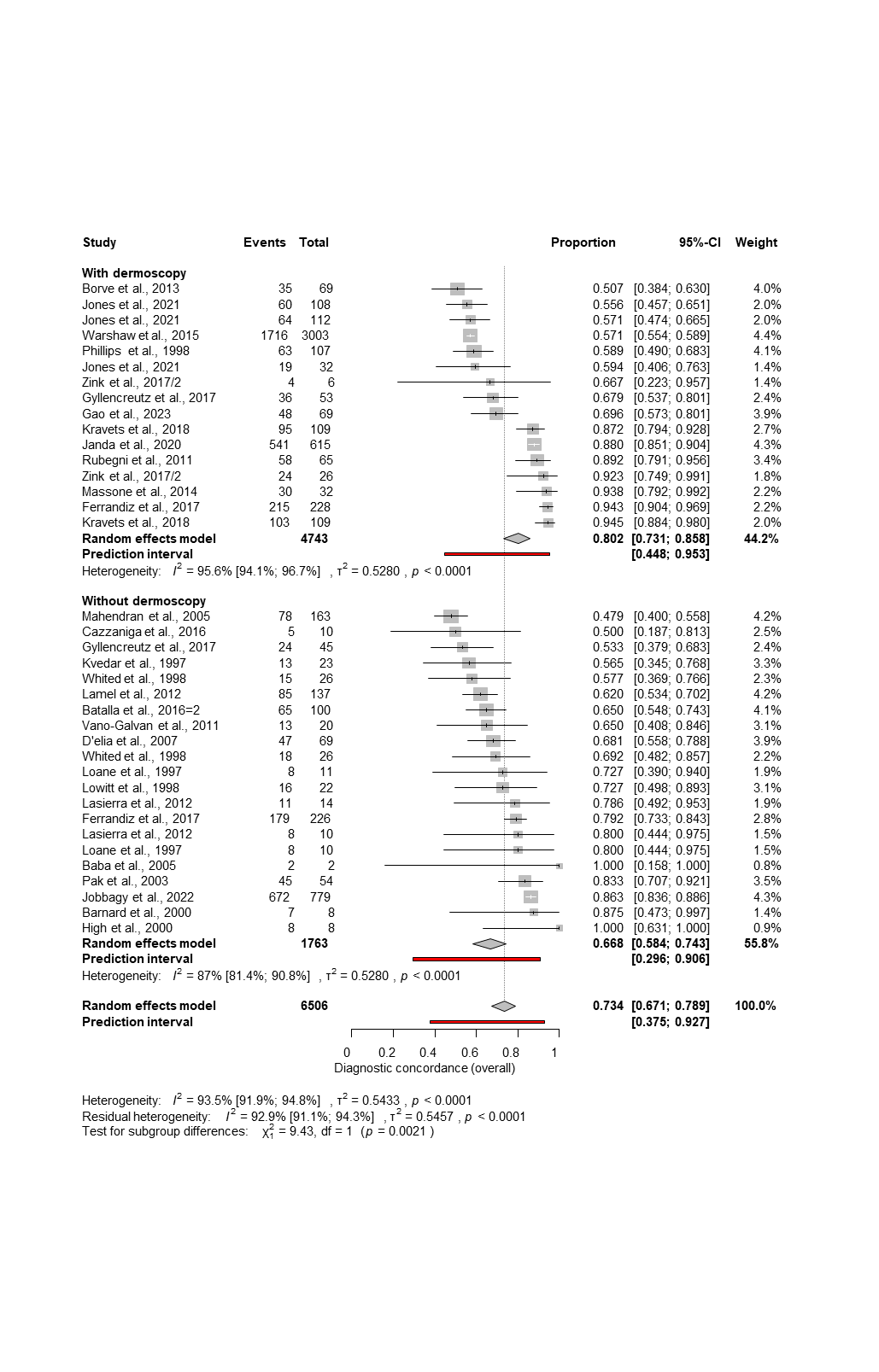


# Supplementary Figure S51: Forest plot comparing the diagnostic concordance between teledermatology providers and in-person dermatologists subgrouped by the use of dermoscopy in the “pigmented lesions” group, including undiagnosed cases in the analysis.


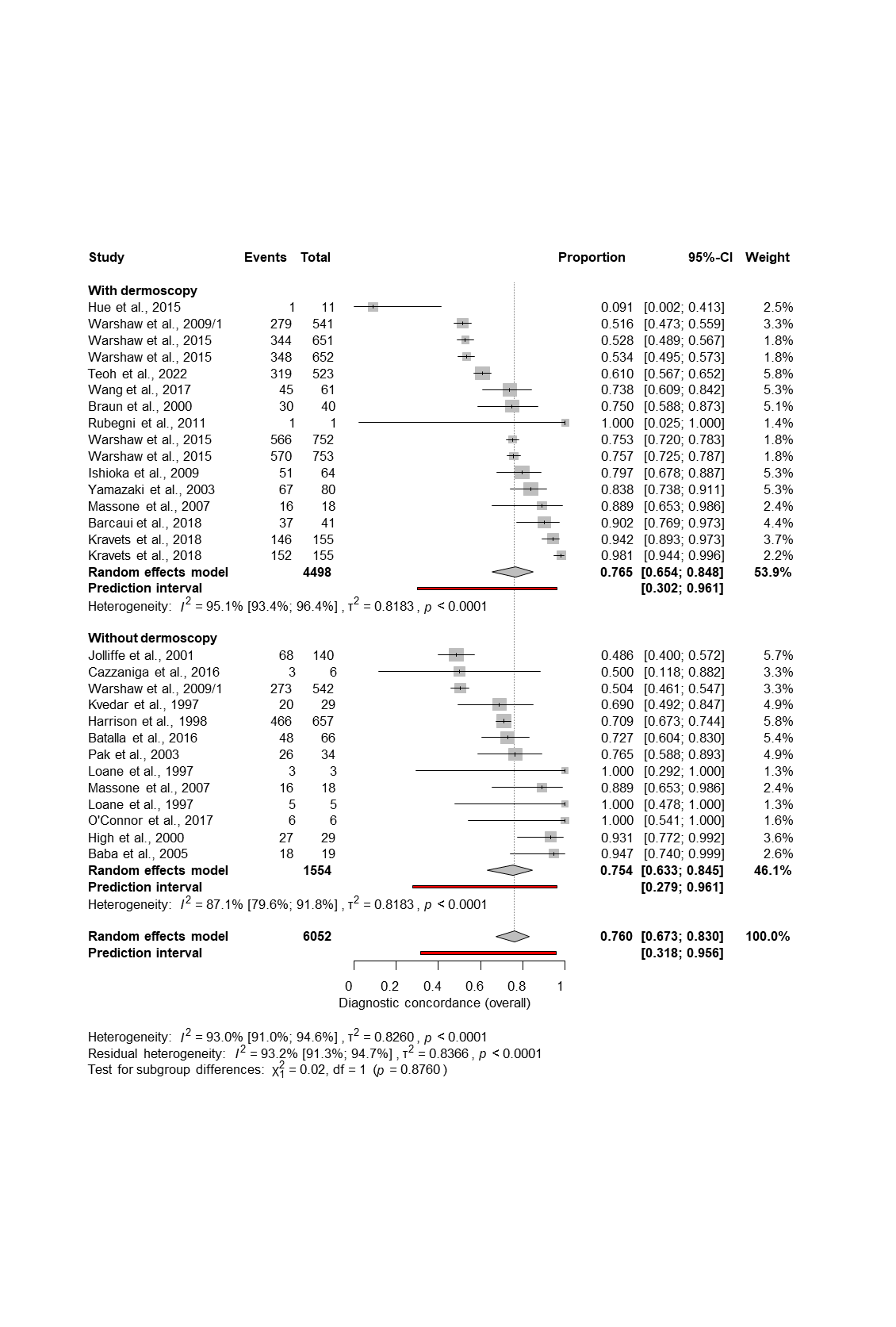


# Supplementary Figure S52: Forest plot comparing the diagnostic concordance between teledermatology providers and in-person dermatologists subgrouped by the photography device in the “all skin conditions” group, including undiagnosed cases in the analysis.


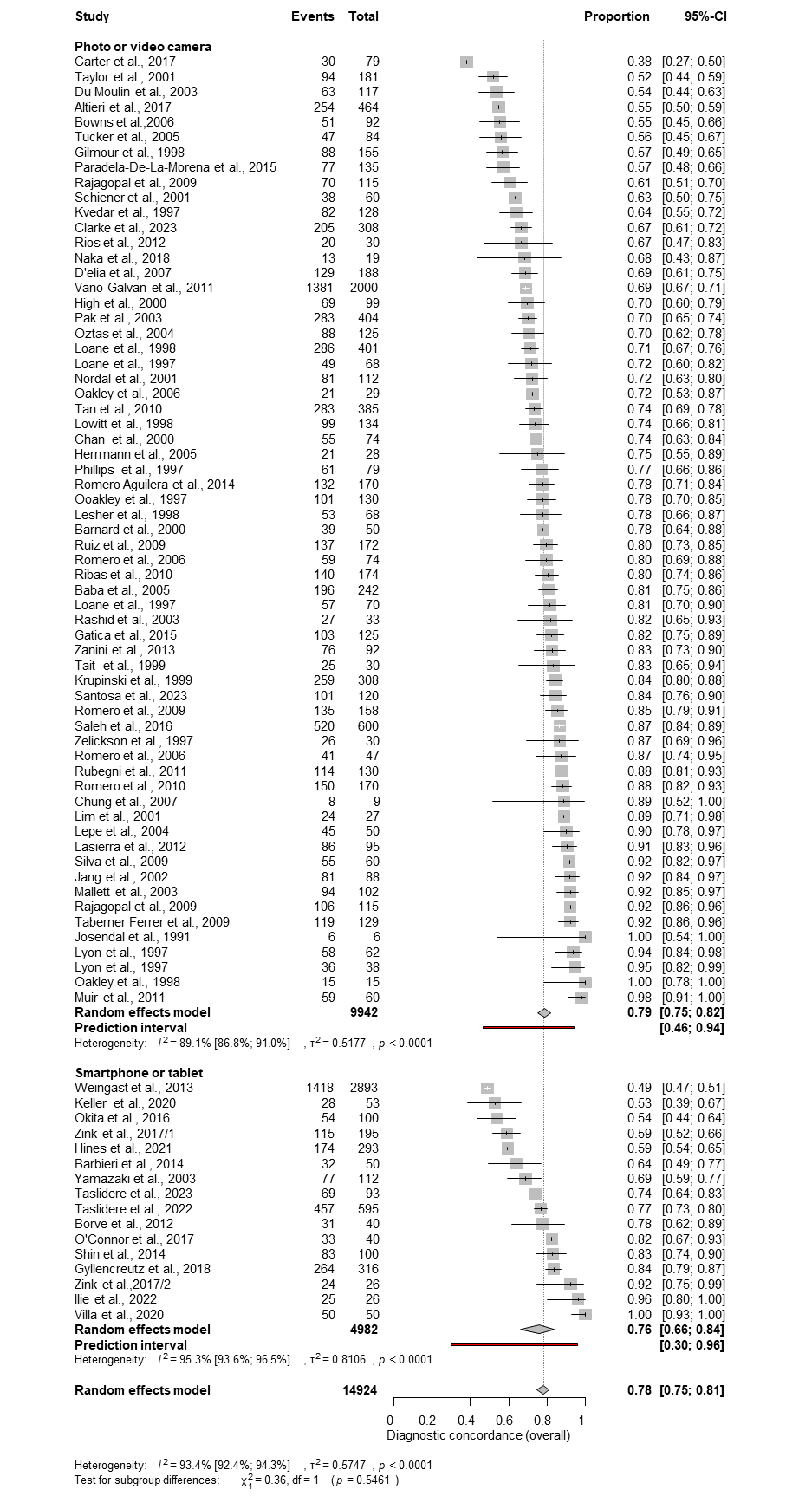


# Supplementary Figure S53: Forest plot comparing the diagnostic concordance between teledermatology providers and in-person dermatologists subgrouped by the photography device in the “skin cancer group”, including undiagnosed cases in the analysis.


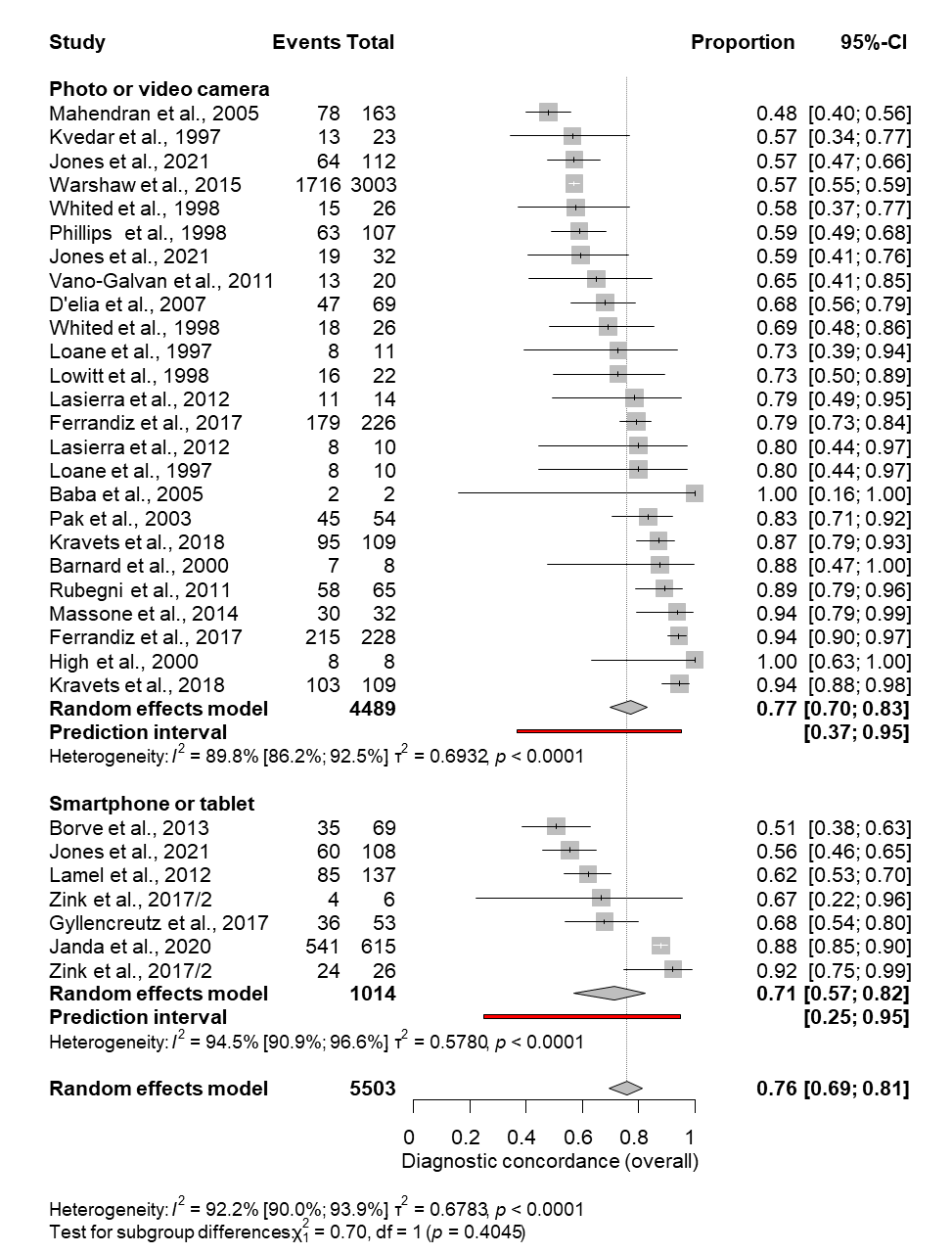


# Supplementary Figure S54: Forest plot comparing the diagnostic concordance between teledermatology providers and in-person dermatologists subgrouped by the photography device in the “pigmented lesions” group, including undiagnosed cases in the analysis.


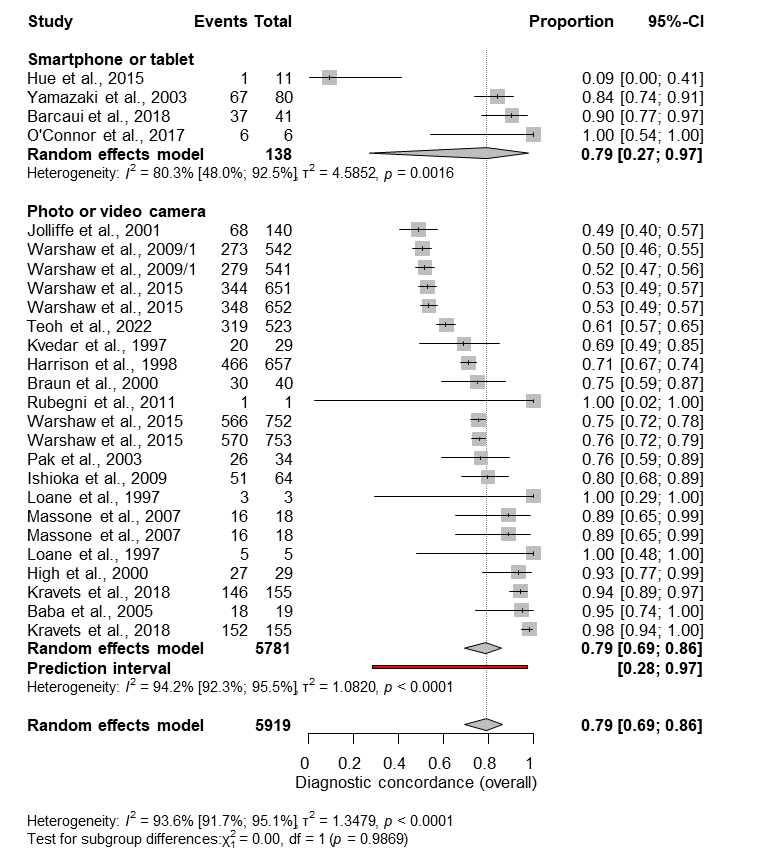


# Supplementary Figure S55: Forest plot comparing the diagnostic concordance between teledermatology providers and in-person dermatologists subgrouped by training for image acquisition in the “all skin conditions” group, including undiagnosed cases in the analysis.


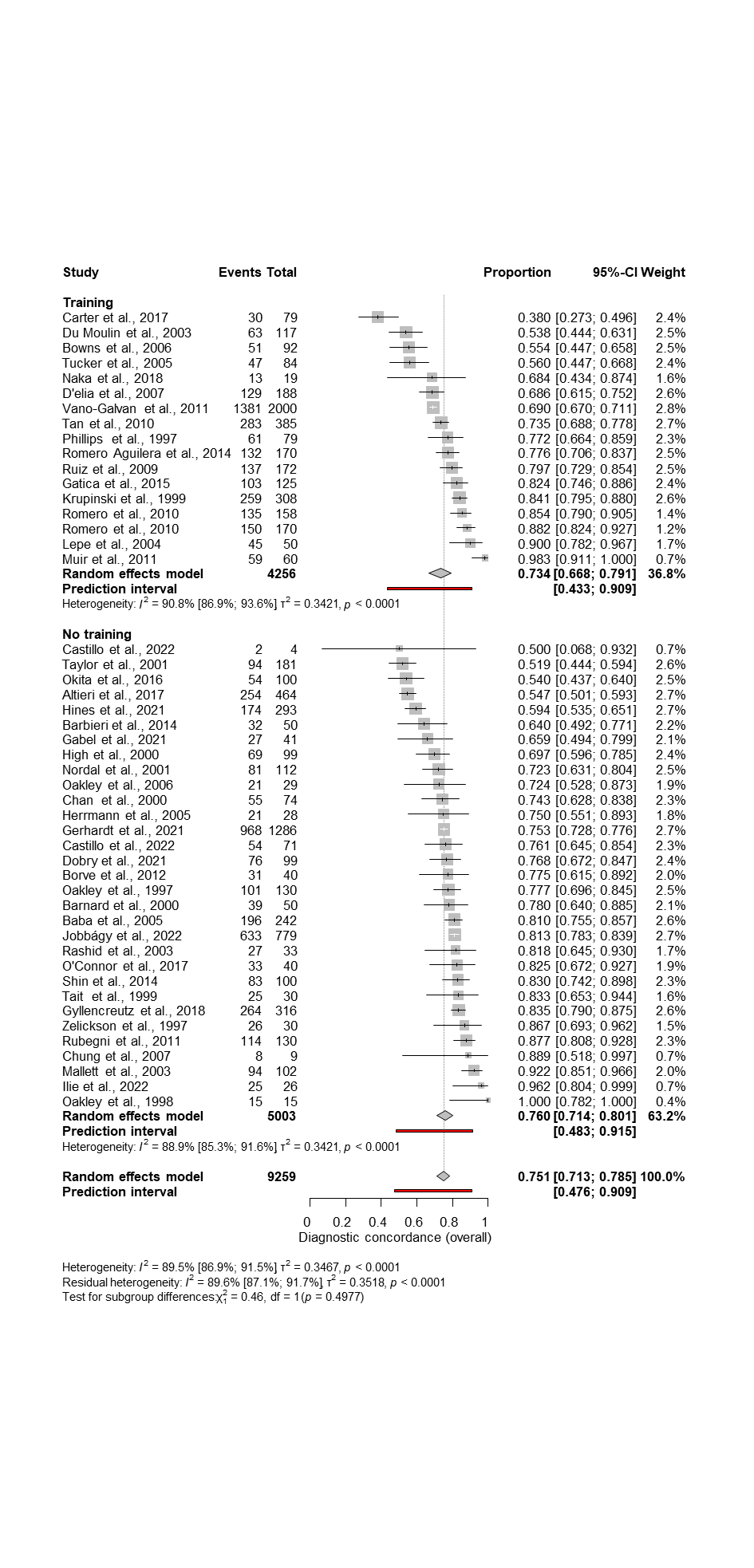


# Supplementary Figure S56: Forest plot comparing the diagnostic concordance between teledermatology providers and in-person dermatologists subgrouped by training for image acquisition in the “skin cancer” group, including undiagnosed cases in the analysis.


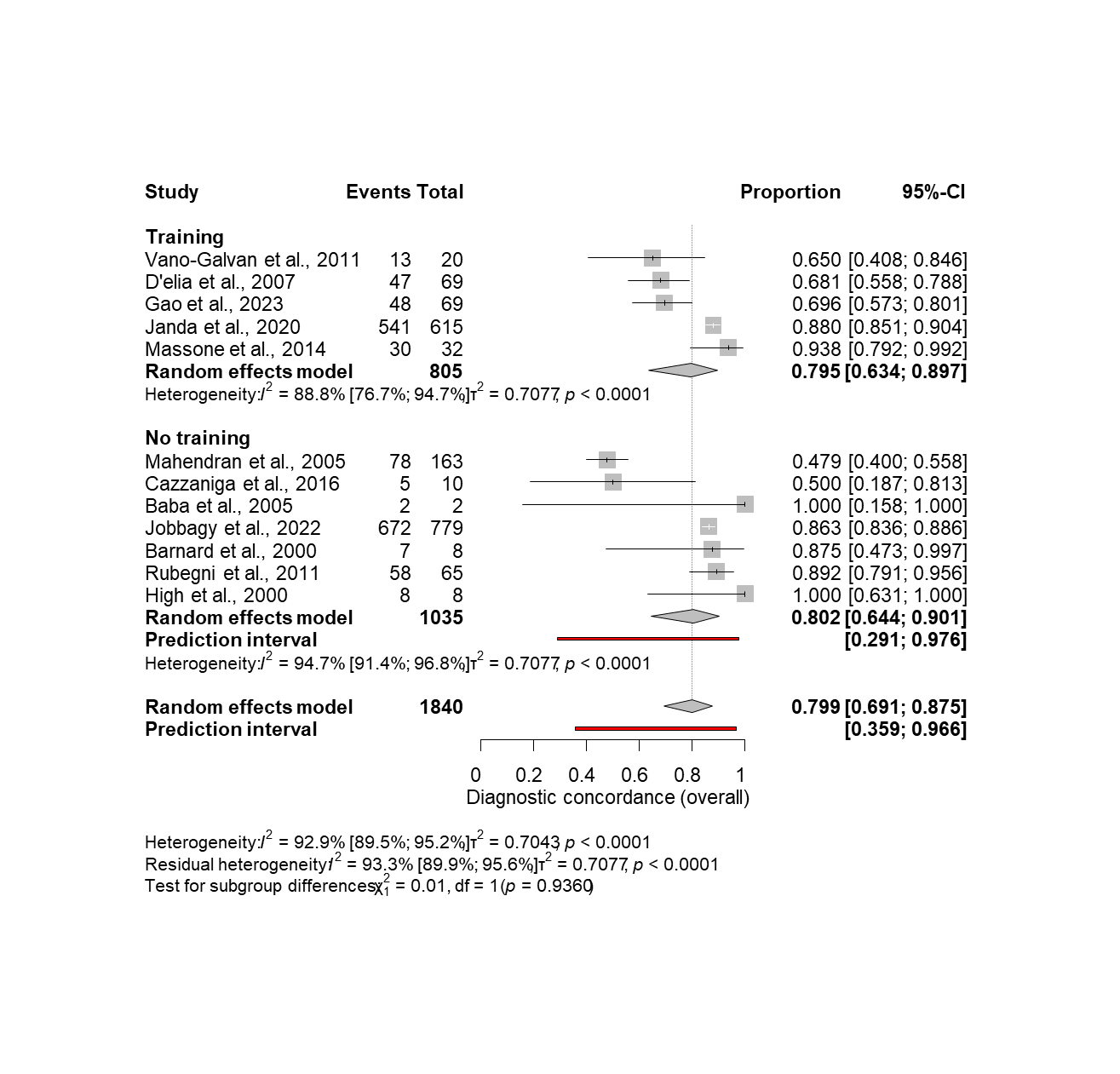


# Supplementary Figure S57: Forest plot comparing the diagnostic concordance between teledermatology providers and in-person dermatologists subgrouped by training for image acquisition in the “pigmented lesions” group, including undiagnosed cases in the analysis.


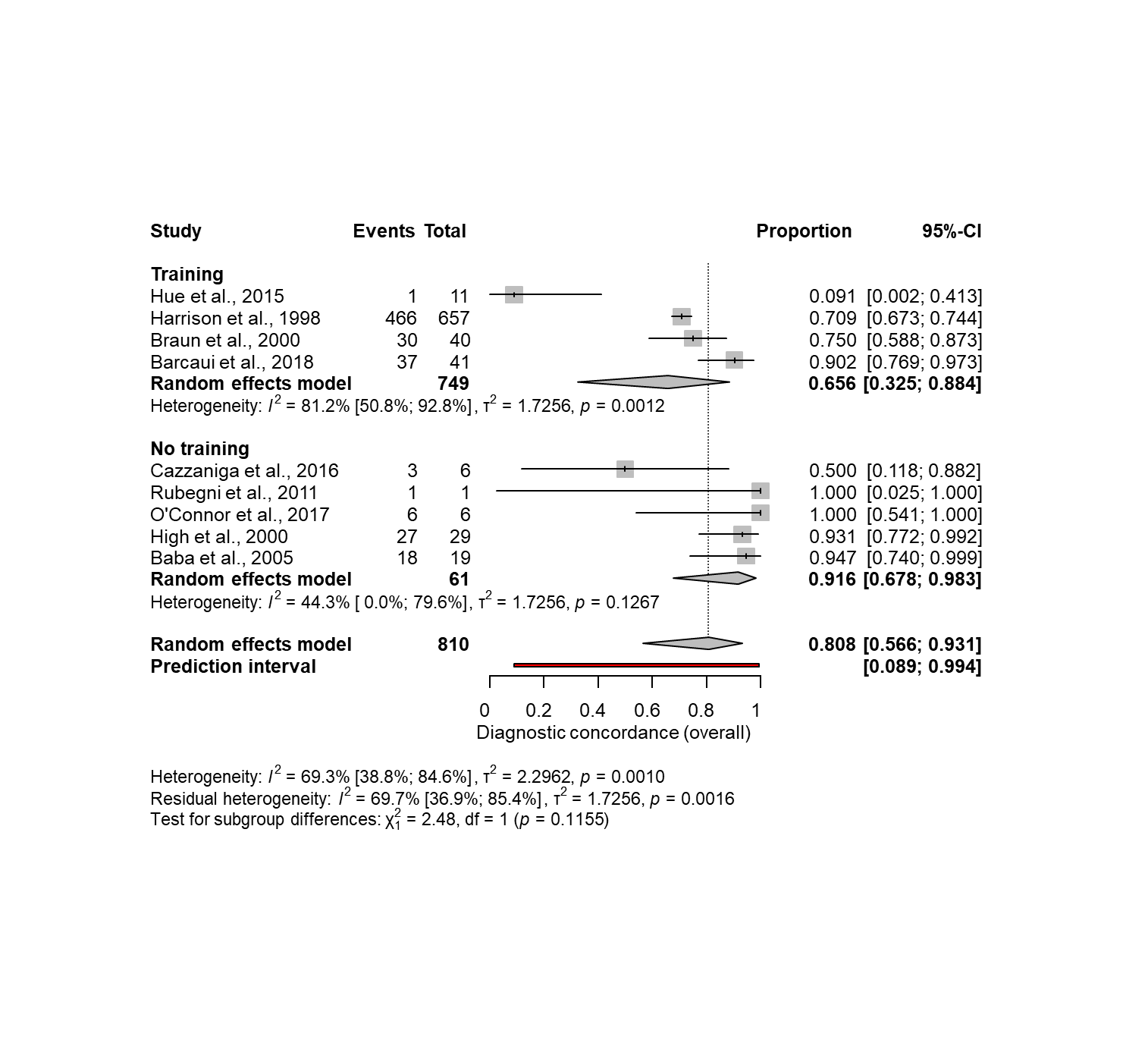


# Supplementary Figure S58: Forest plot comparing the diagnostic concordance between teledermatology providers and in-person dermatologists subgrouped by the comparator in the “all skin conditions” group, including undiagnosed cases in the analysis.


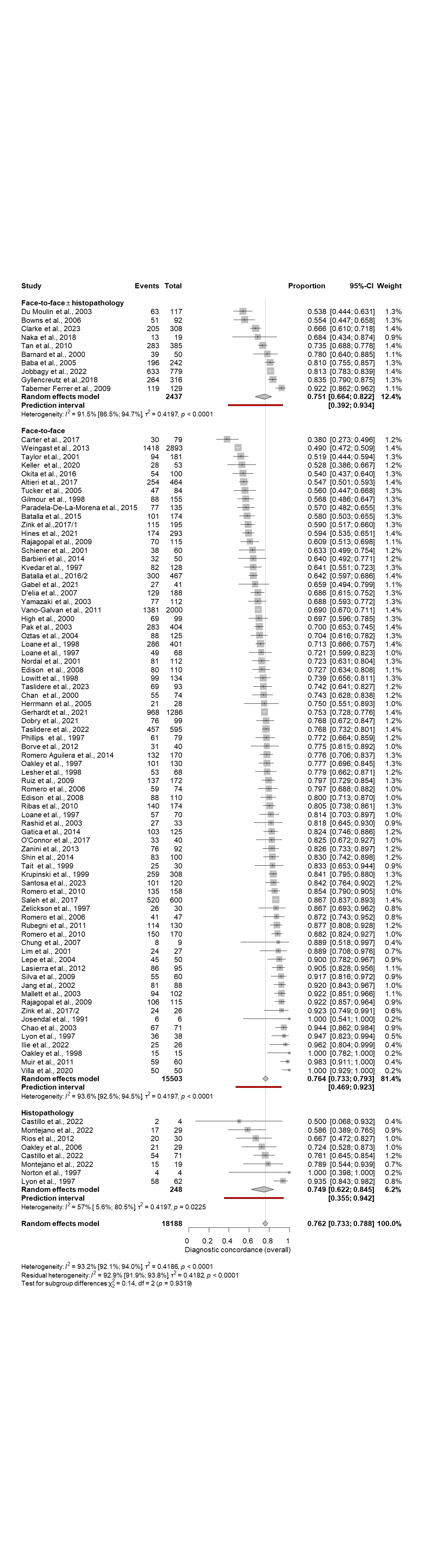


# Supplementary Figure S59: Forest plot comparing the diagnostic concordance between teledermatology providers and in-person dermatologists subgrouped by the comparator in the “skin cancer” group, including undiagnosed cases in the analysis.


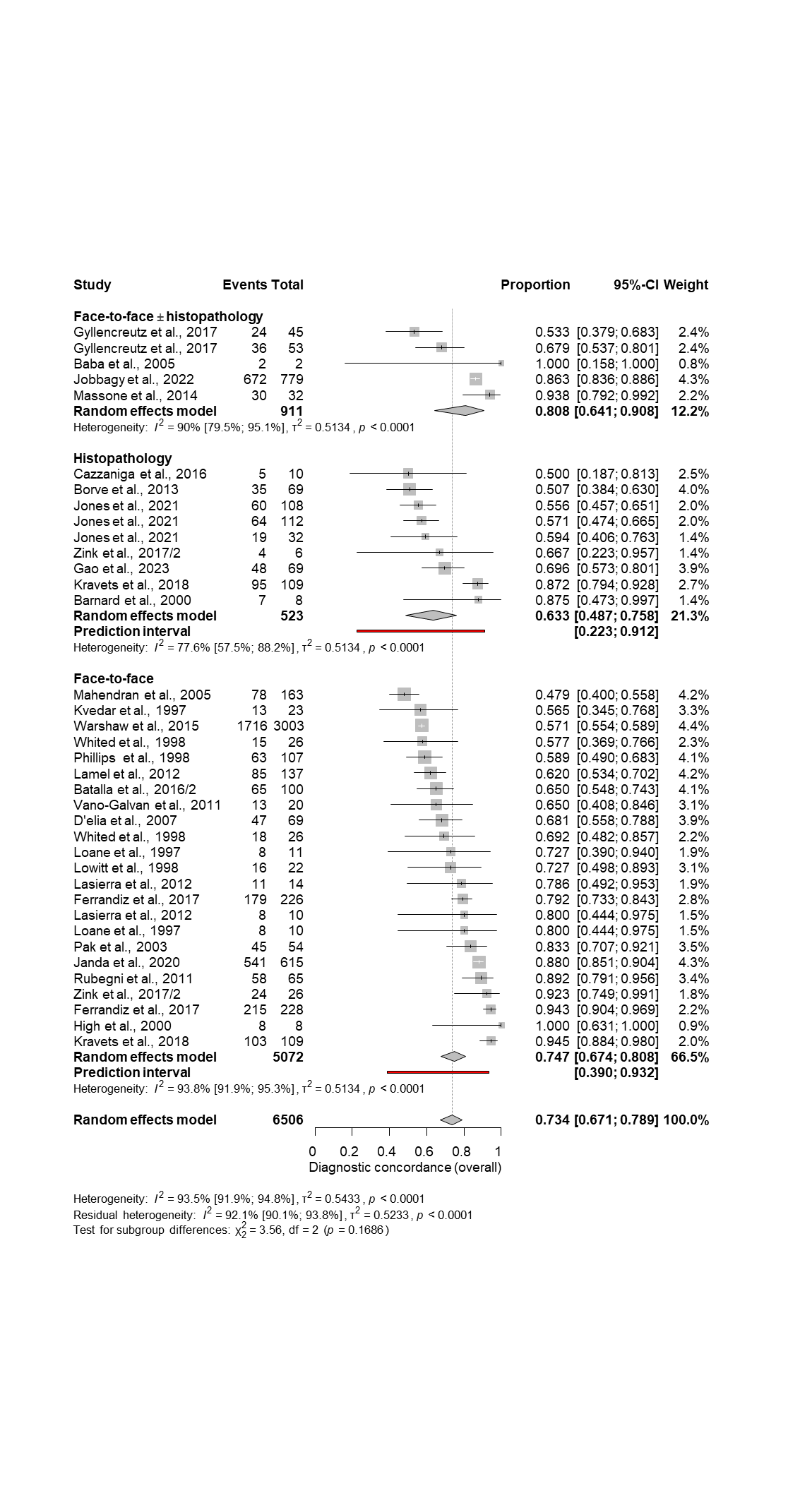


# Supplementary Figure S60: Forest plot comparing the diagnostic concordance between teledermatology providers and in-person dermatologists subgrouped by the comparator in the “pigmented lesions” group, including undiagnosed cases in the analysis.


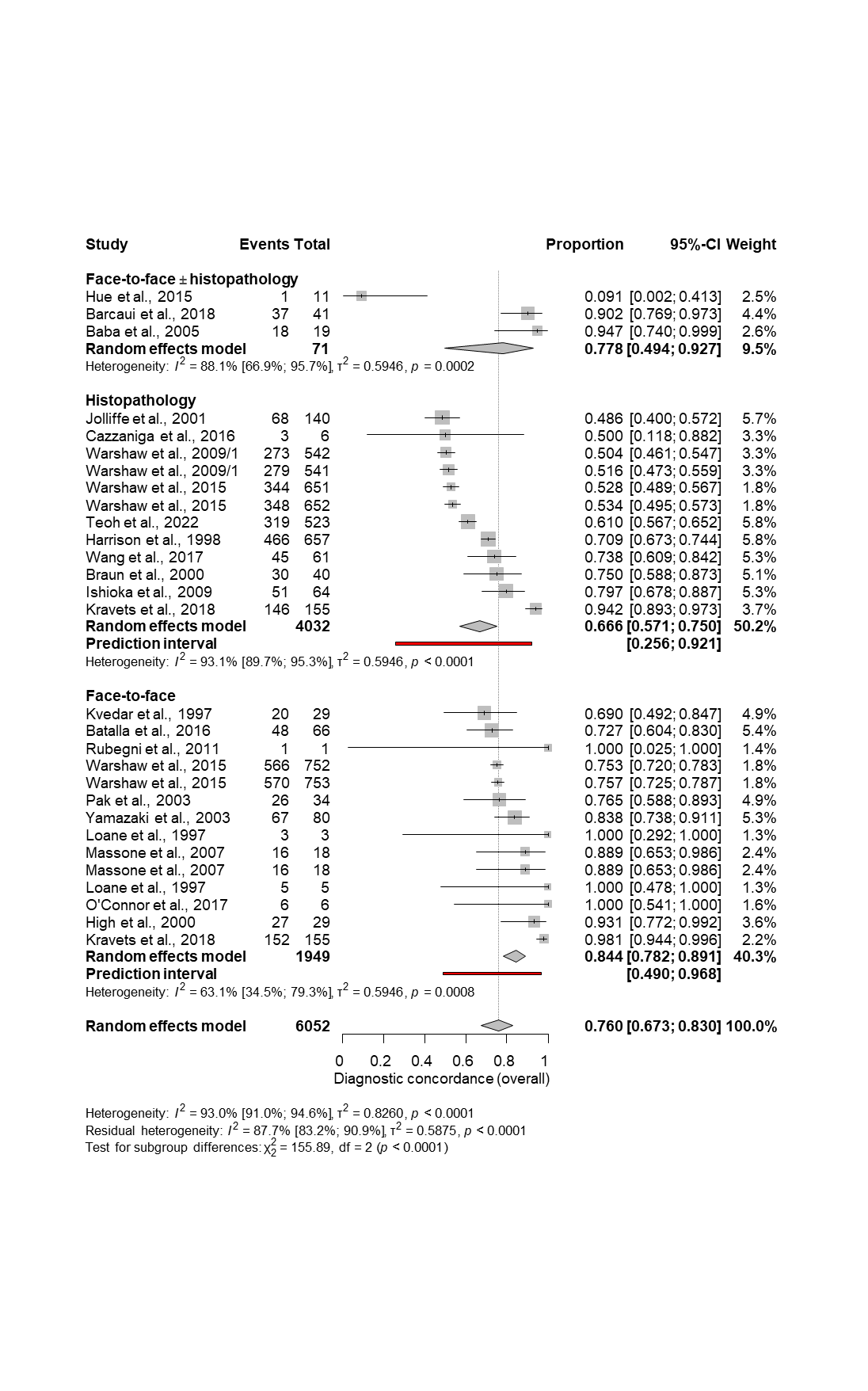


# Supplementary Figure S61: Forest plot comparing Cohen’s kappa between teledermatology providers and in-person dermatologists based on the communication platform in the ”all skin conditions” group, including undiagnosed cases in the analysis.


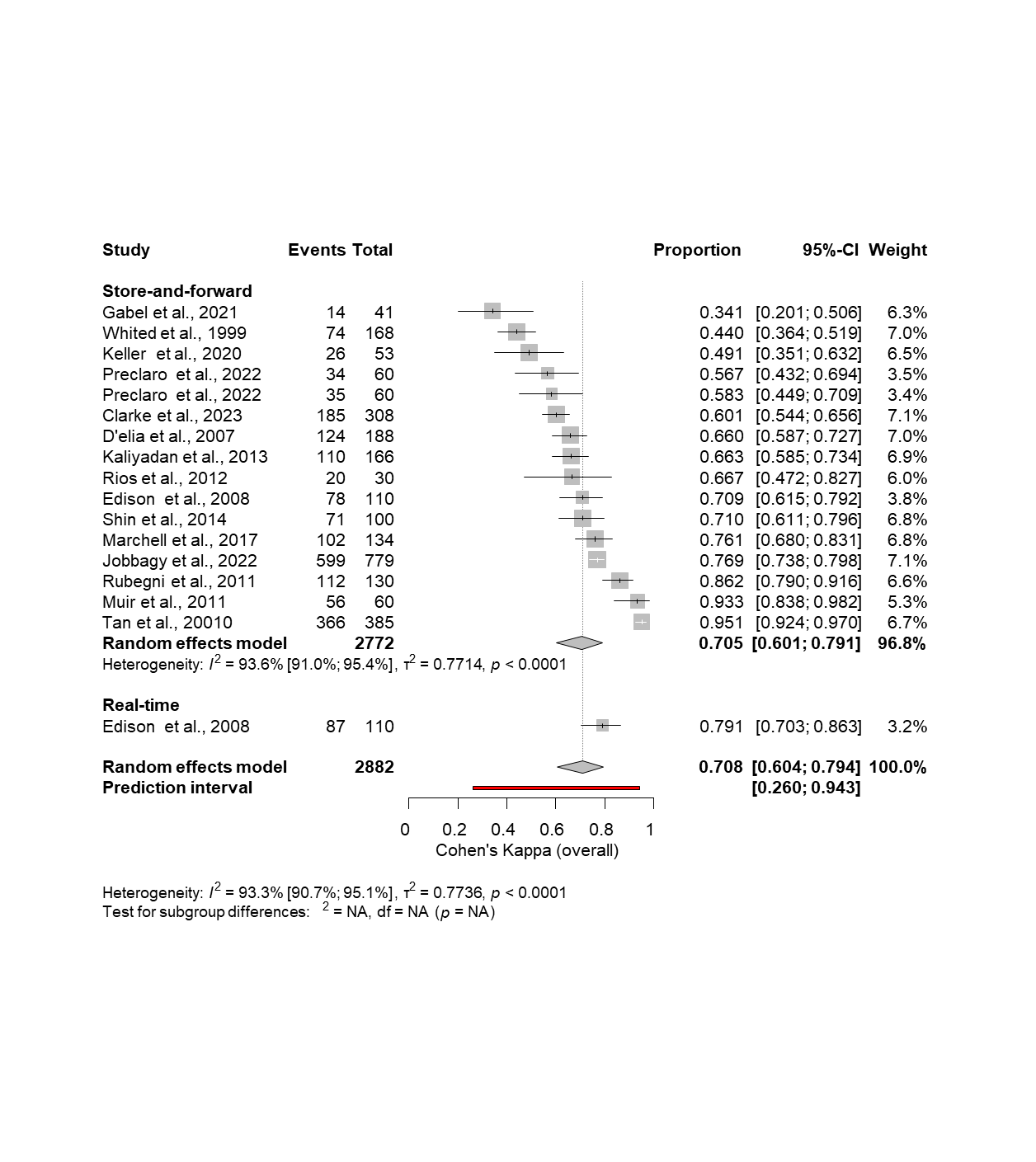


# Supplementary Figure S62: Forest plot comparing Cohen’s kappa between teledermatology providers and in-person dermatologists based on the communication platform in the “skin cancer” group, including undiagnosed cases in the analysis.


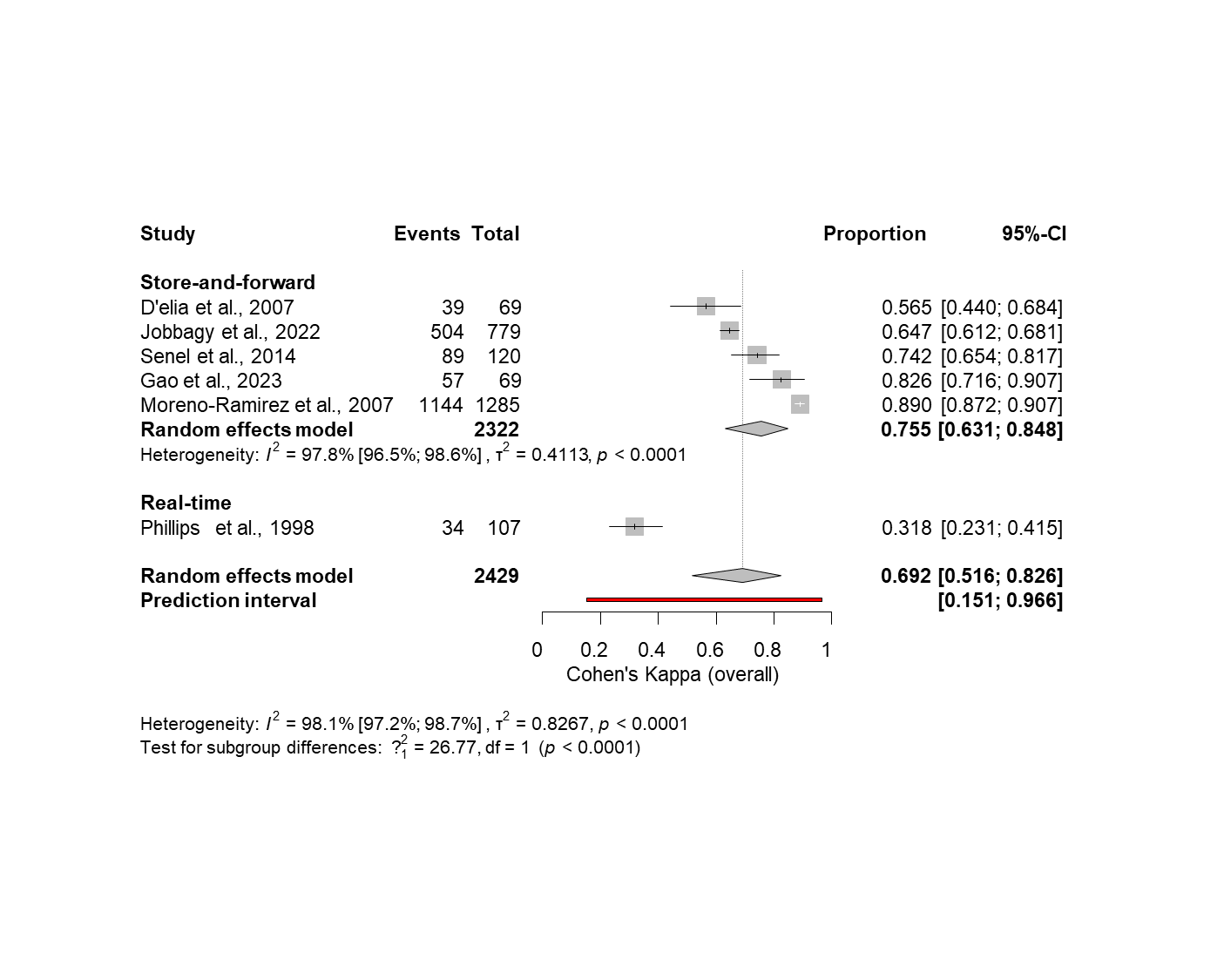


# Supplementary Figure S63: Forest plot comparing Cohen’s kappa between teledermatology providers and in-person dermatologists based on the communication platform in the “pigmented lesions” group. including undiagnosed cases in the analysis.


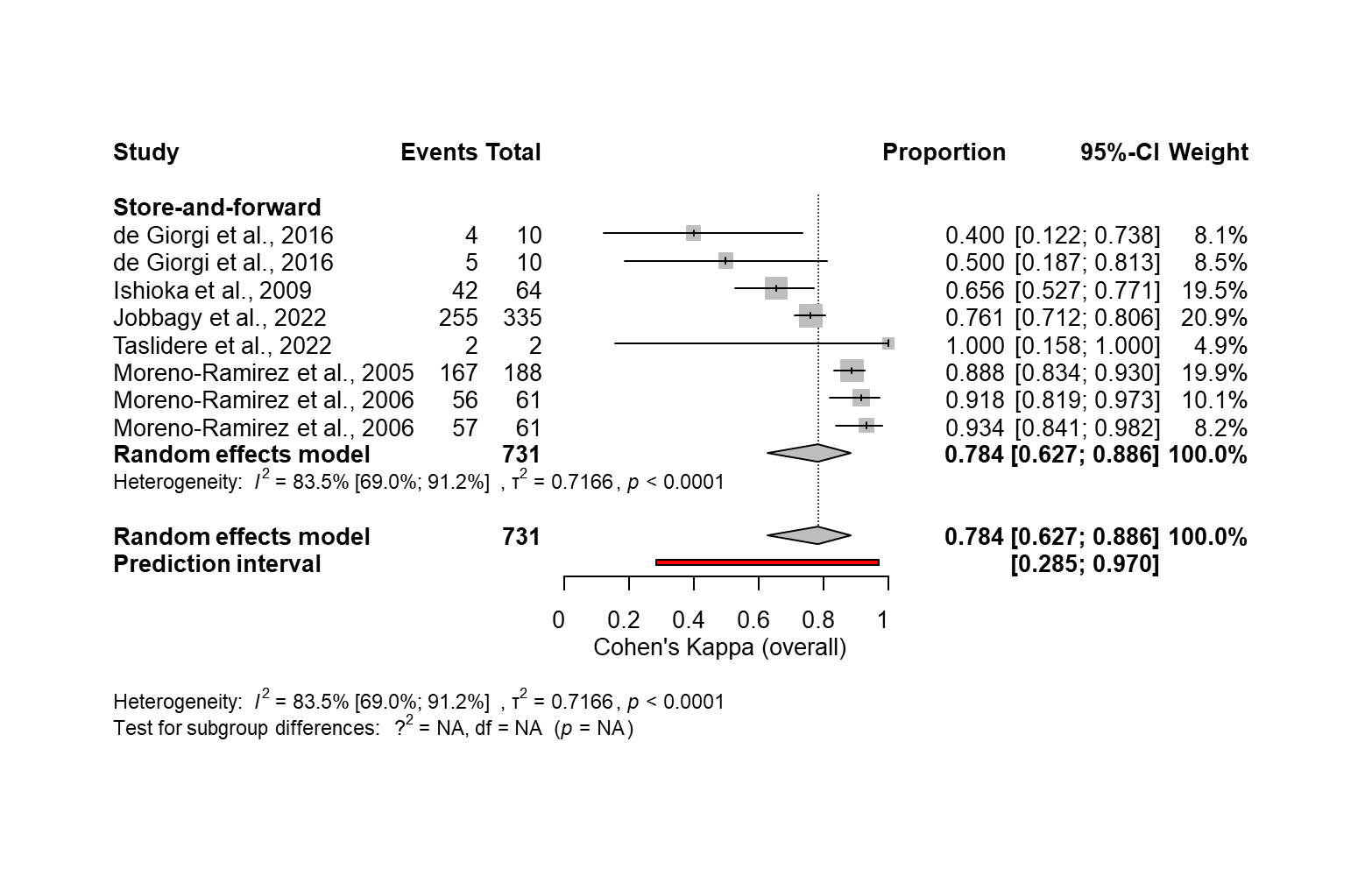


# Supplementary Figure S64: Forest plot comparing Cohen’s kappa between teledermatology providers and in-person dermatologists based on the communication type in the “all skin conditions” group, including undiagnosed cases in the analysis.


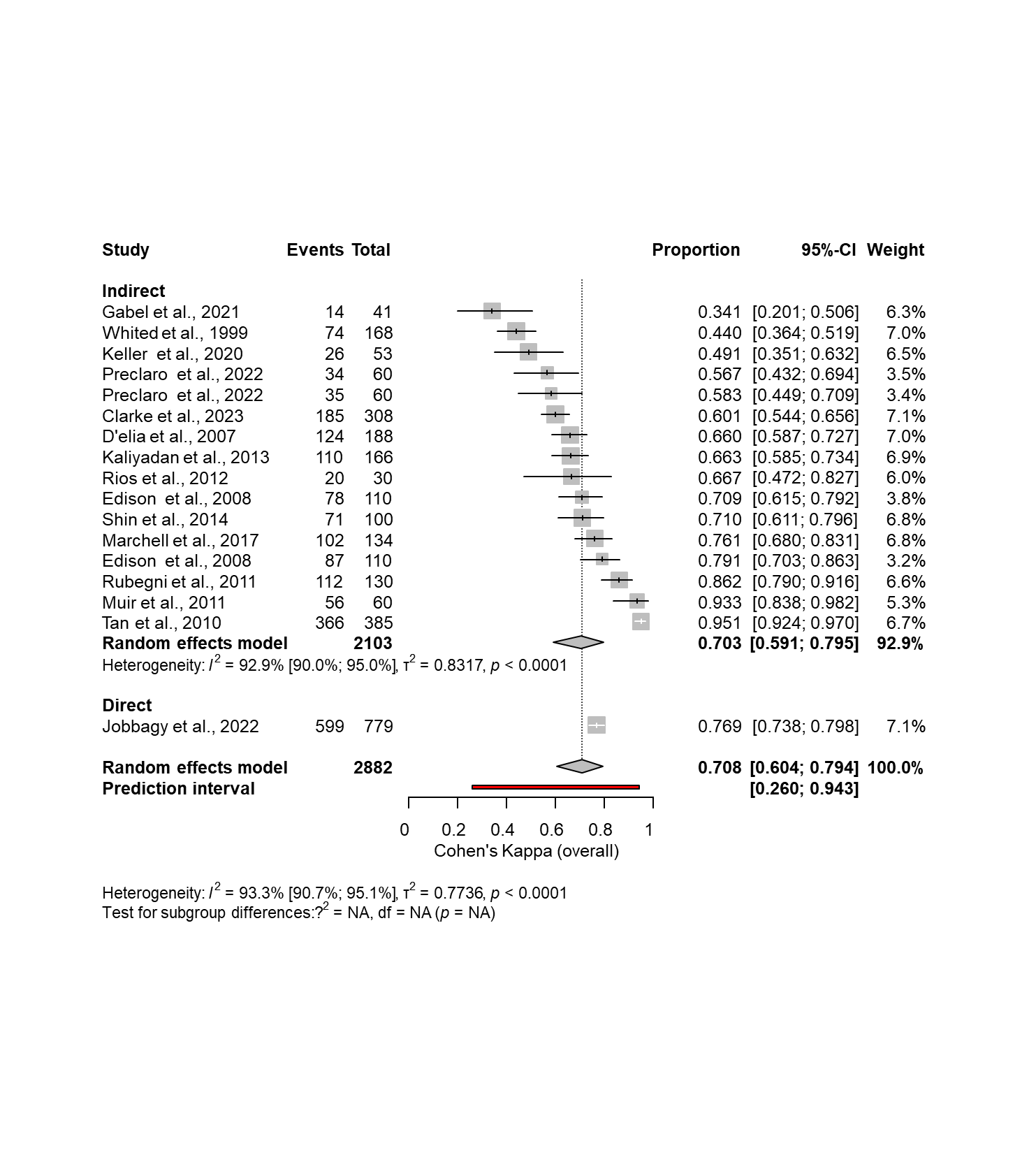


# Supplementary Figure S65: Forest plot comparing Cohen’s kappa between teledermatology providers and in-person dermatologists based on the communication type in the “skin cancer” group, including undiagnosed cases in the analysis.


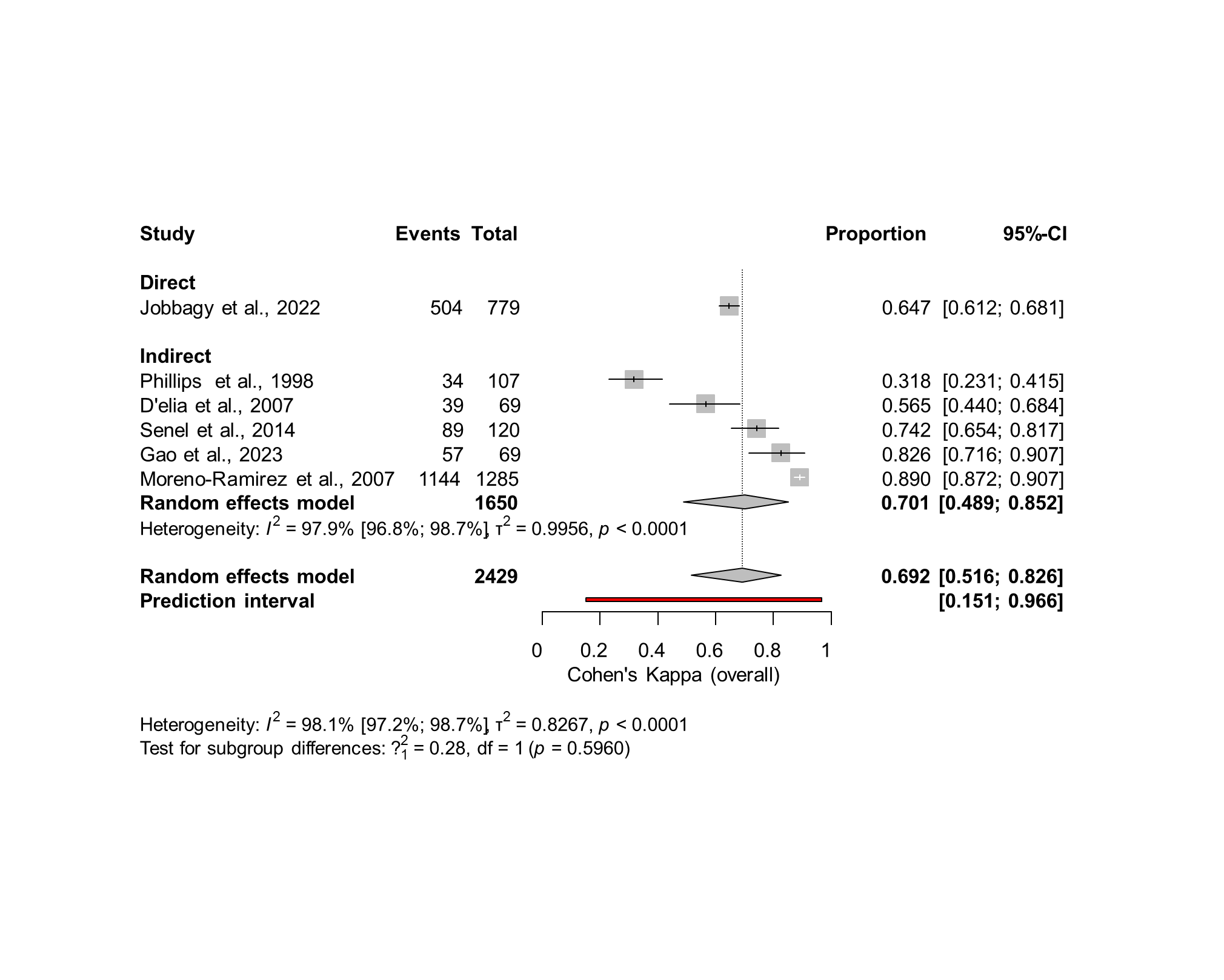


# Supplementary Figure S66: Forest plot comparing Cohen’s kappa between teledermatology providers and in-person dermatologists based on the communication type in the “pigmented lesions” group, including undiagnosed cases in the analysis.


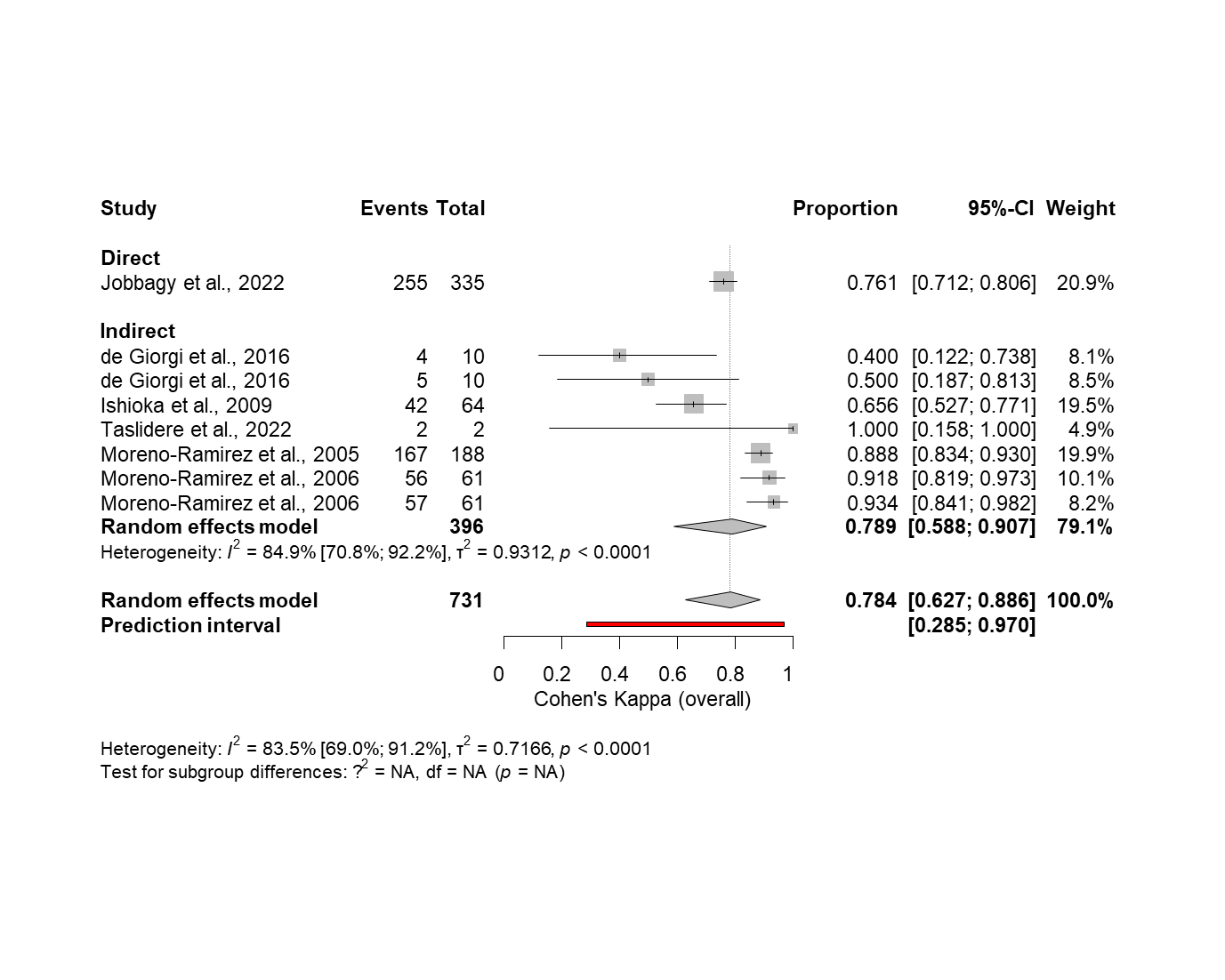


# Supplementary Figure S67: Forest plot comparing Cohen’s kappa between teledermatology providers and in-person dermatologists based on the use of dermoscopy in the “all skin conditions” group, including undiagnosed cases in the analysis.


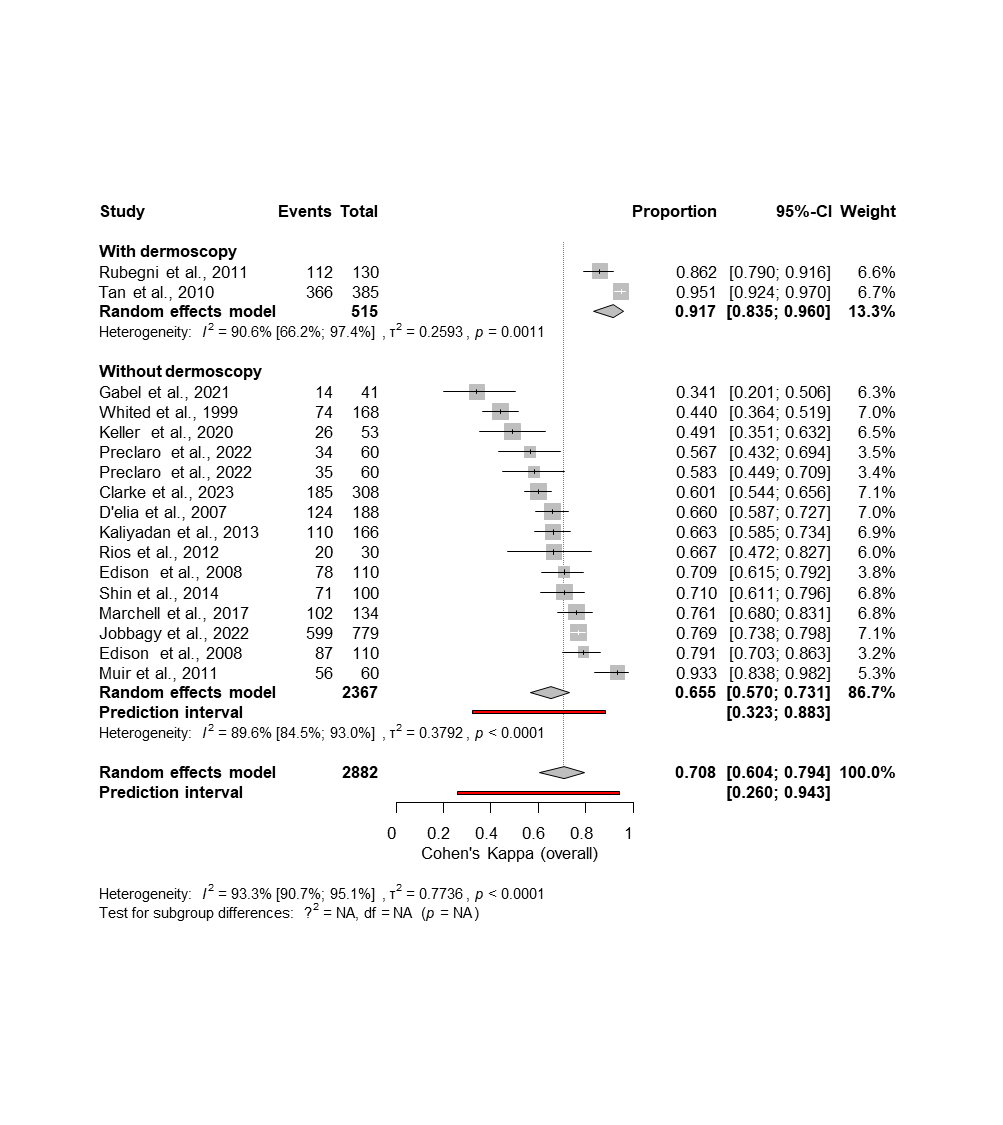


# Supplementary Figure S68: Forest plot comparing Cohen’s kappa between teledermatology providers and in-person dermatologists based on the use of dermoscopy in the “skin cancer” group, including undiagnosed cases in the analysis.


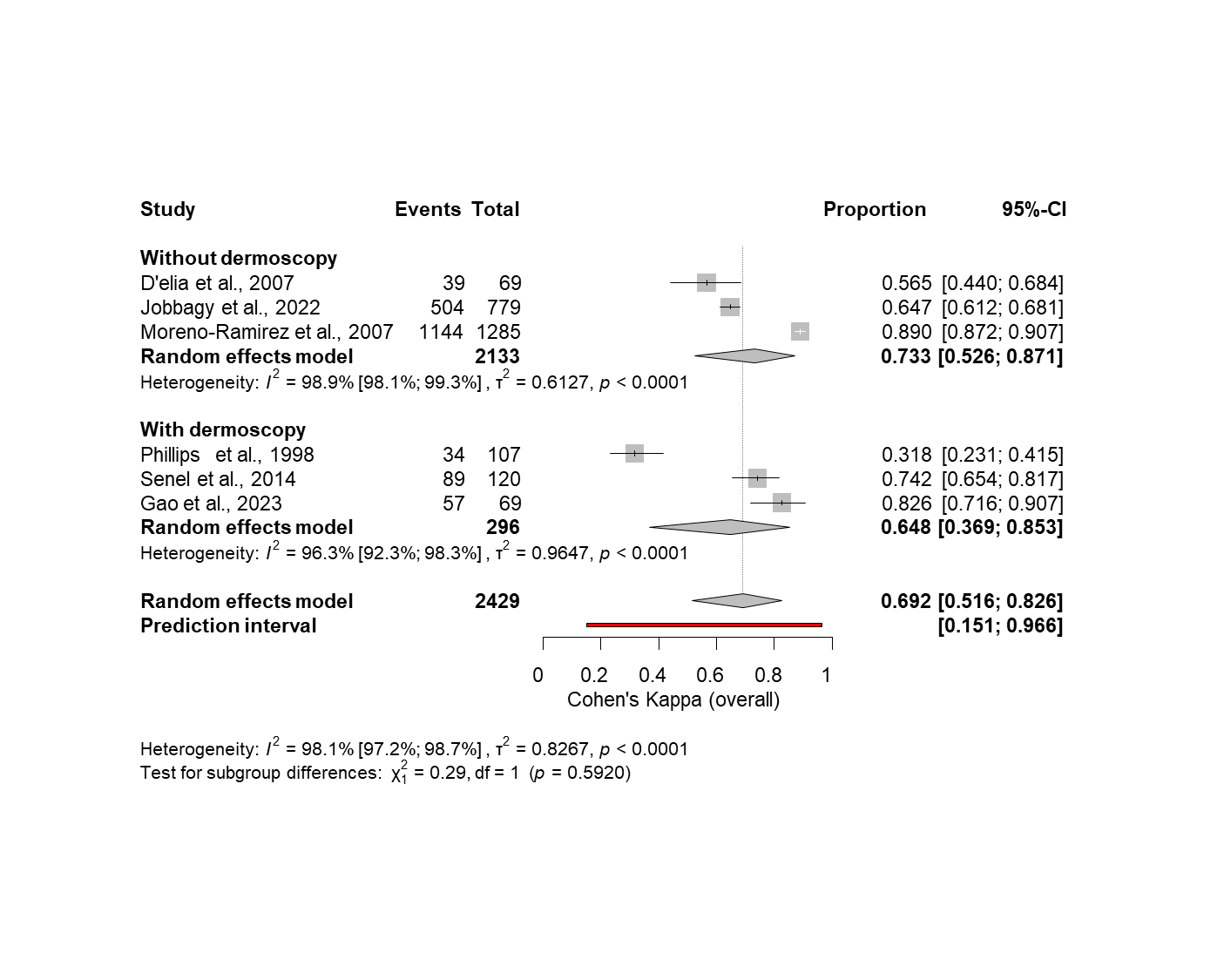


# Supplementary Figure S69: Forest plot comparing Cohen’s kappa between teledermatology providers and in-person dermatologists based on the use of dermoscopy in the “pigmented lesions” group, including undiagnosed cases in the analysis.


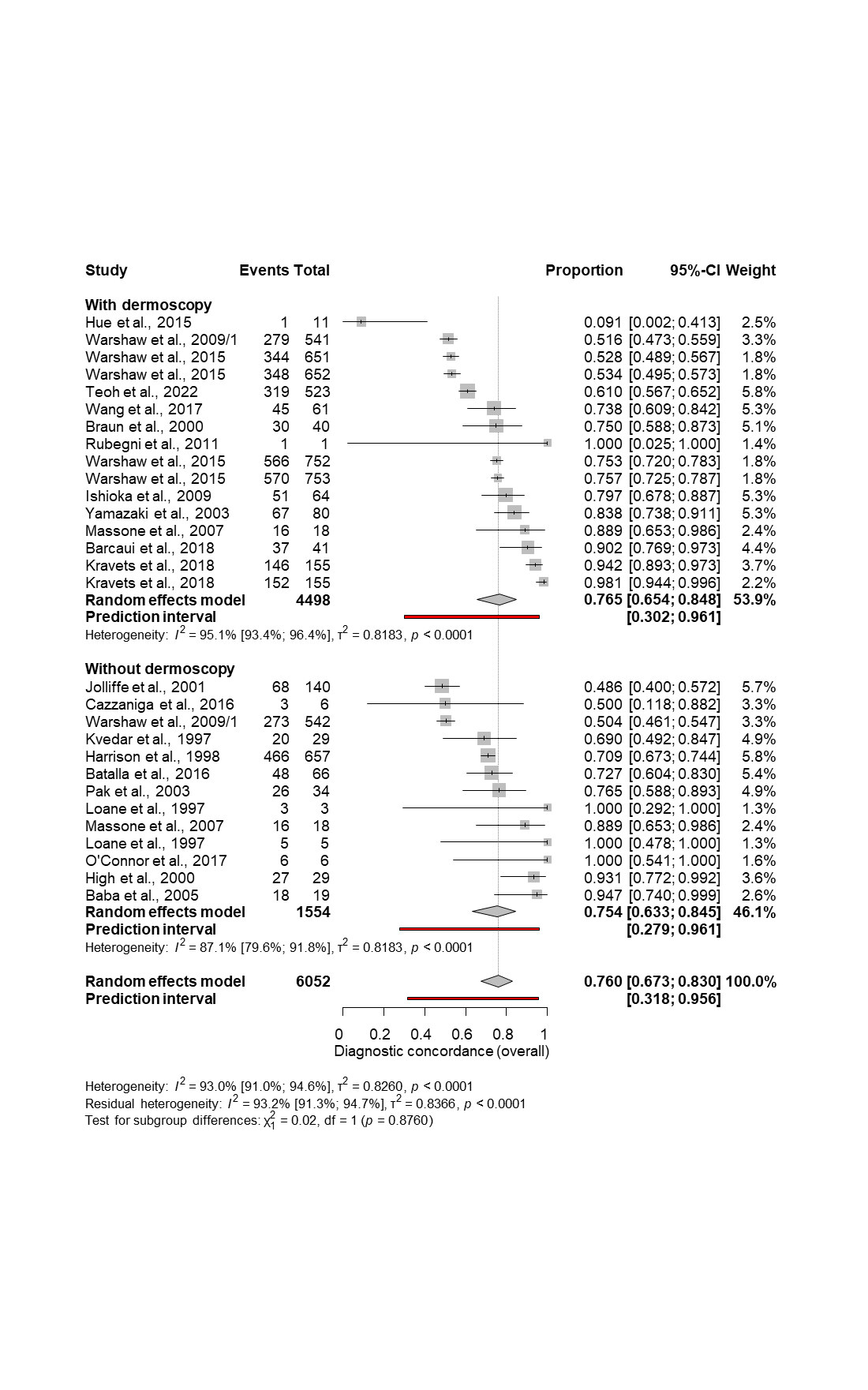


# Supplementary Figure S70: Forest plot comparing Cohen’s kappa between teledermatology providers and in-person dermatologists based on the photography device in the “all skin conditions” group, including undiagnosed cases in the analysis.


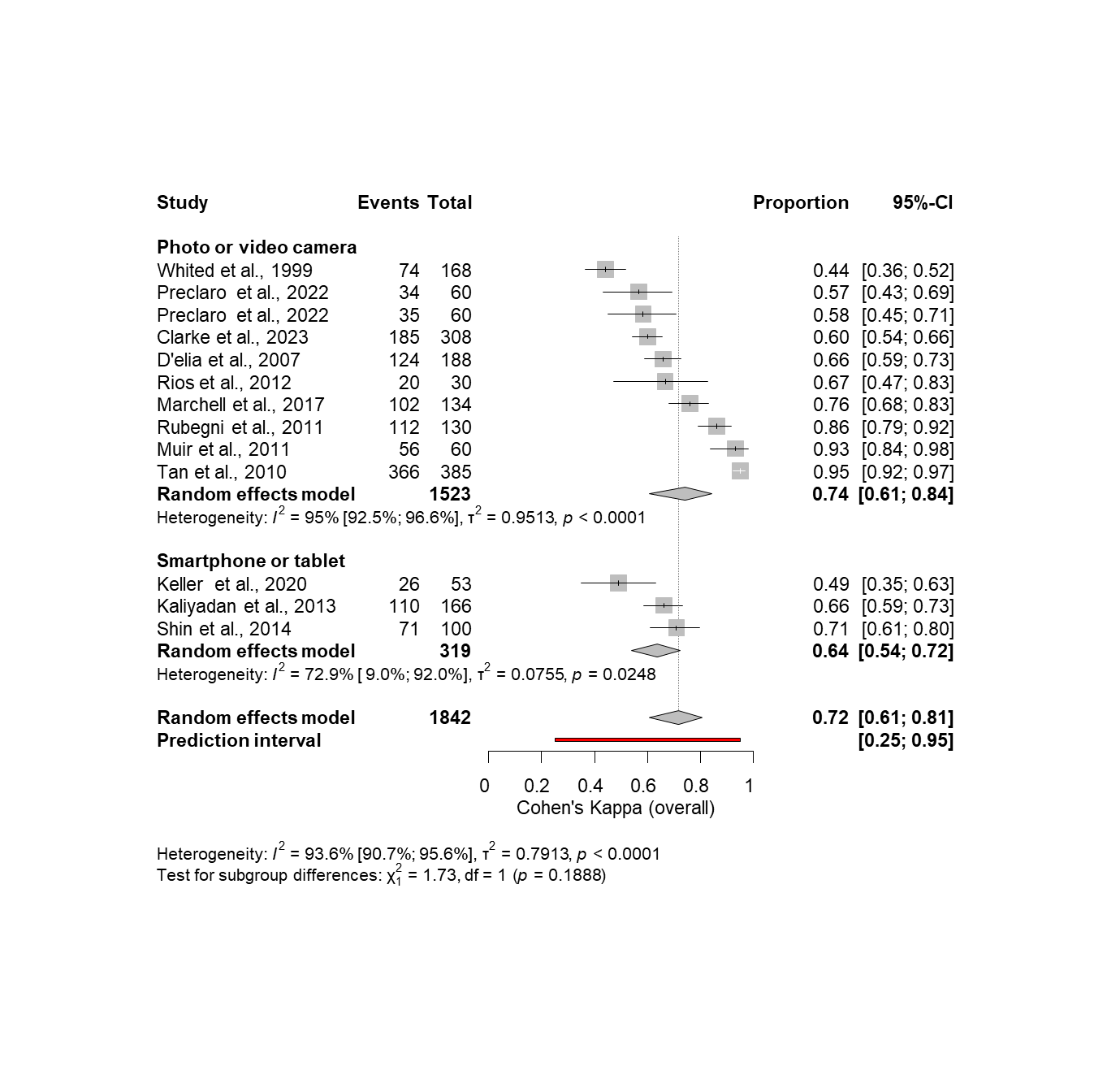


# Supplementary Figure S71: Forest plot comparing Cohen’s kappa between teledermatology providers and in-person dermatologists based on the photography device in the “skin cancer” group, including undiagnosed cases in the analysis.


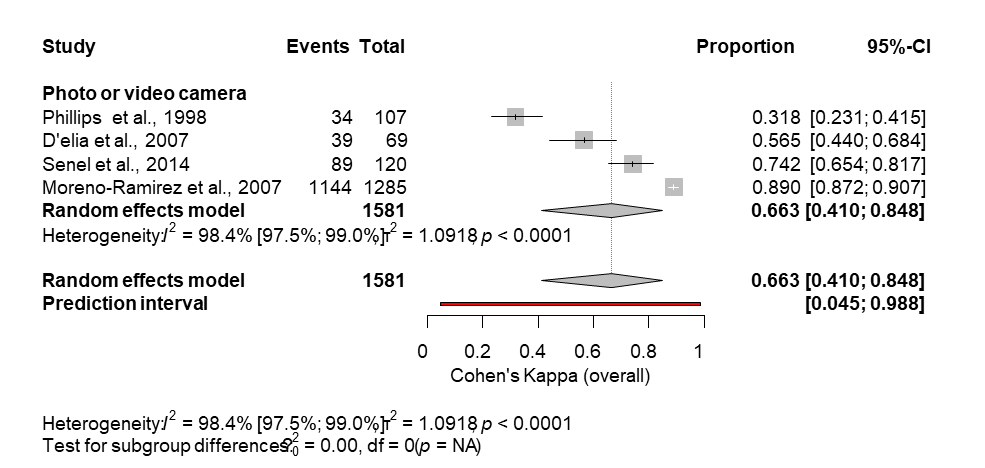


# Supplementary Figure S72: Forest plot comparing Cohen’s kappa between teledermatology providers and in-person dermatologists based on the photography device in the “pigmented lesions” group, including undiagnosed cases in the analysis.


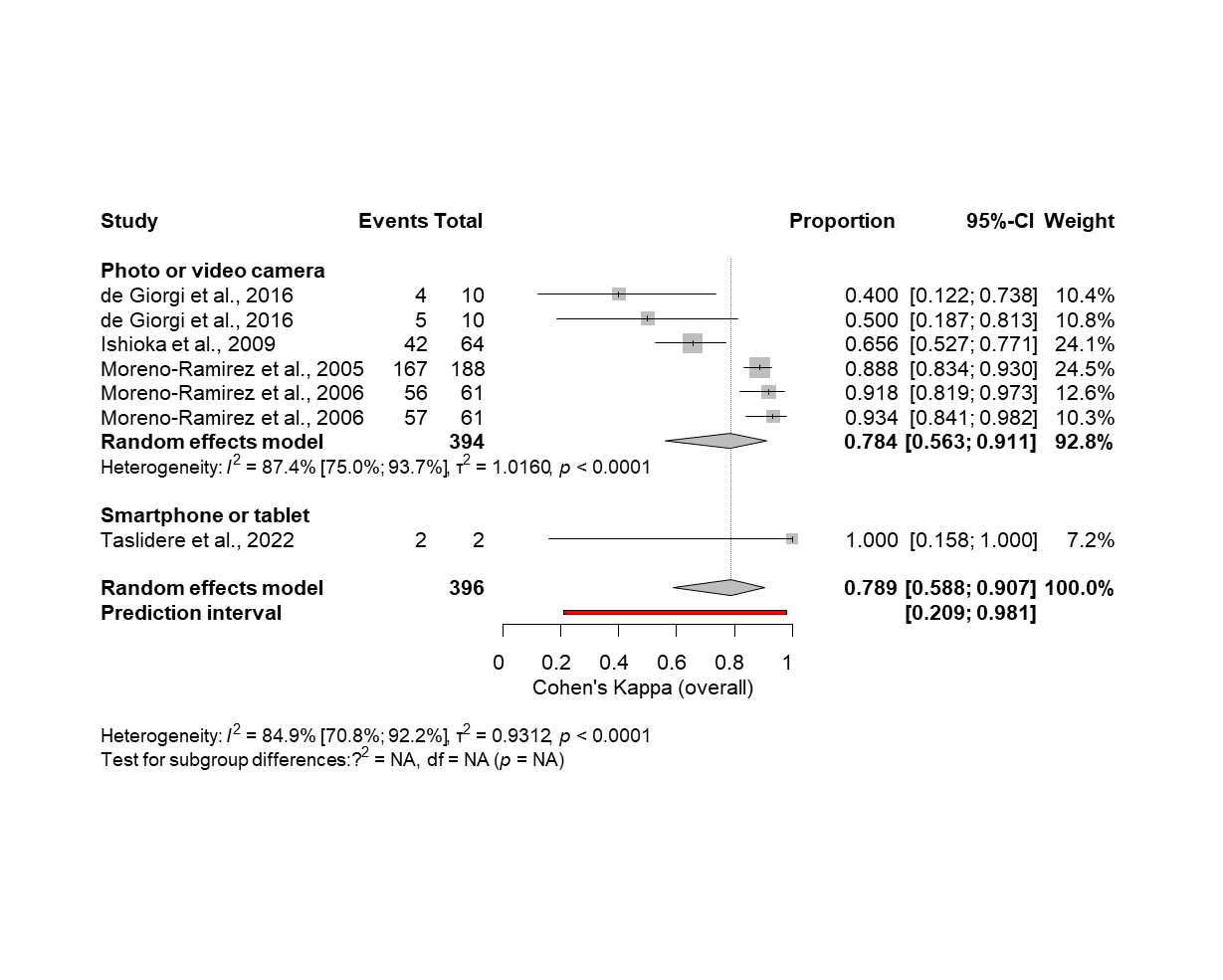


# Supplementary Figure S73: Forest plot comparing Cohen’s kappa between teledermatology providers and in-person dermatologists based on training for image acquisition in the “all skin conditions” group, including undiagnosed cases in the analysis.


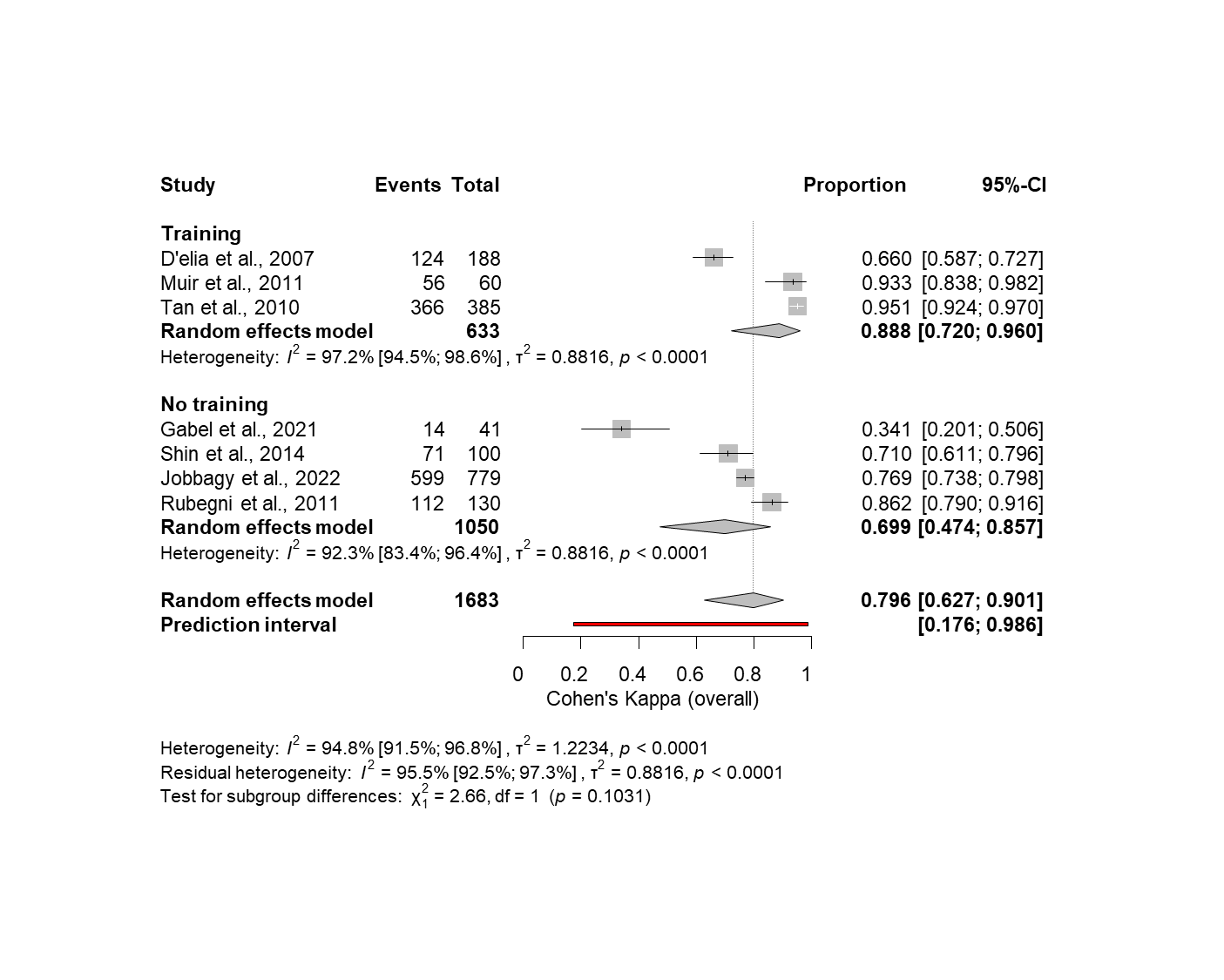


# Supplementary Figure S74: Forest plot comparing Cohen’s kappa between teledermatology providers and in-person dermatologists based on training for image acquisition in the “skin cancer” group, including undiagnosed cases in the analysis.


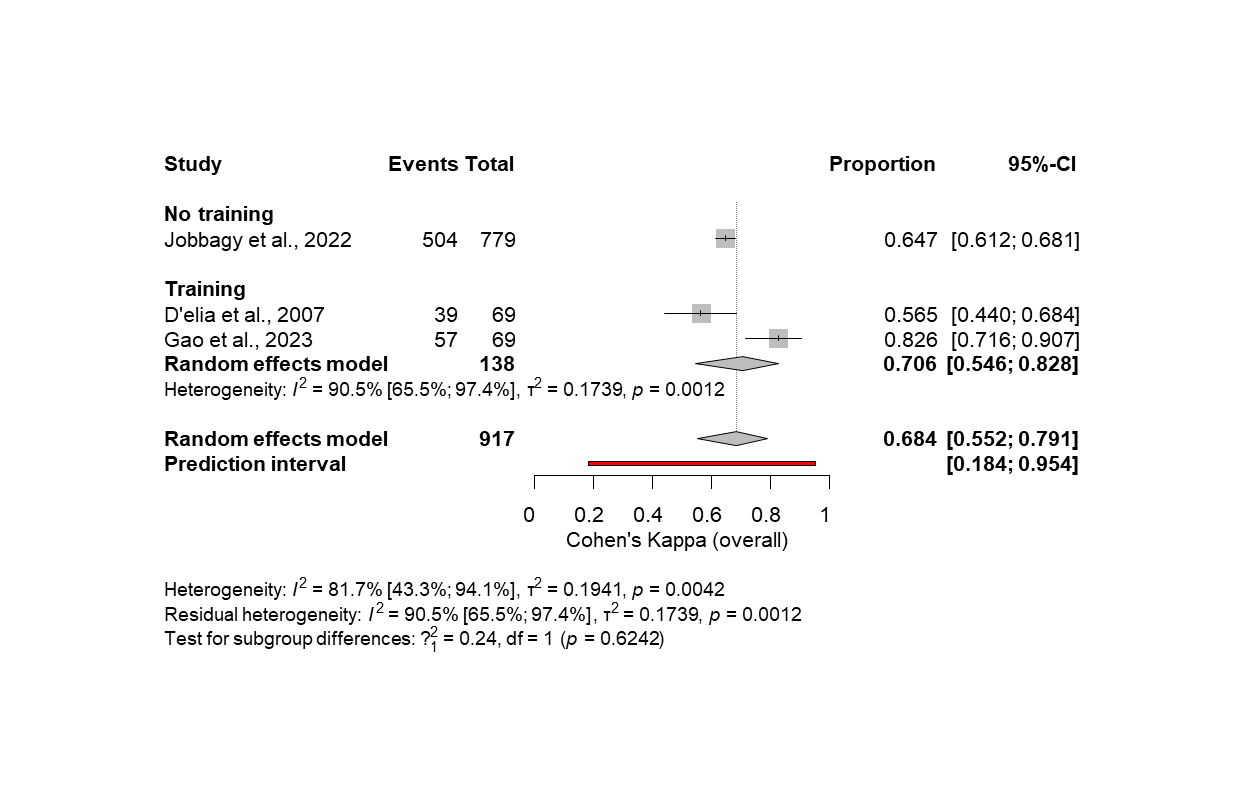


# Supplementary Figure S75: Forest plot comparing Cohen’s kappa between teledermatology providers and in-person dermatologists based on training for image acquisition in the “pigmented lesions” group, including undiagnosed cases in the analysis.


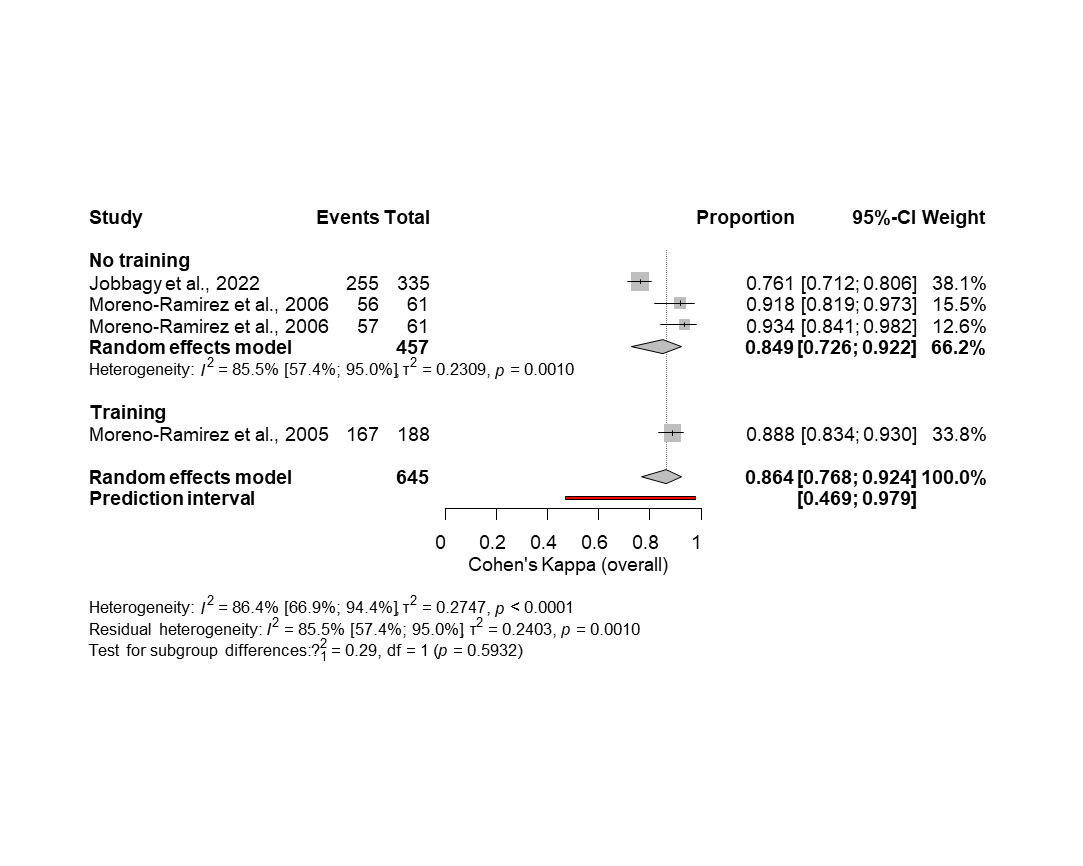


# Supplementary Figure S76: Forest plot comparing Cohen’s kappa between teledermatology providers and in-person dermatologists based on the comparator in the “all skin conditions” group, including undiagnosed cases in the analysis.


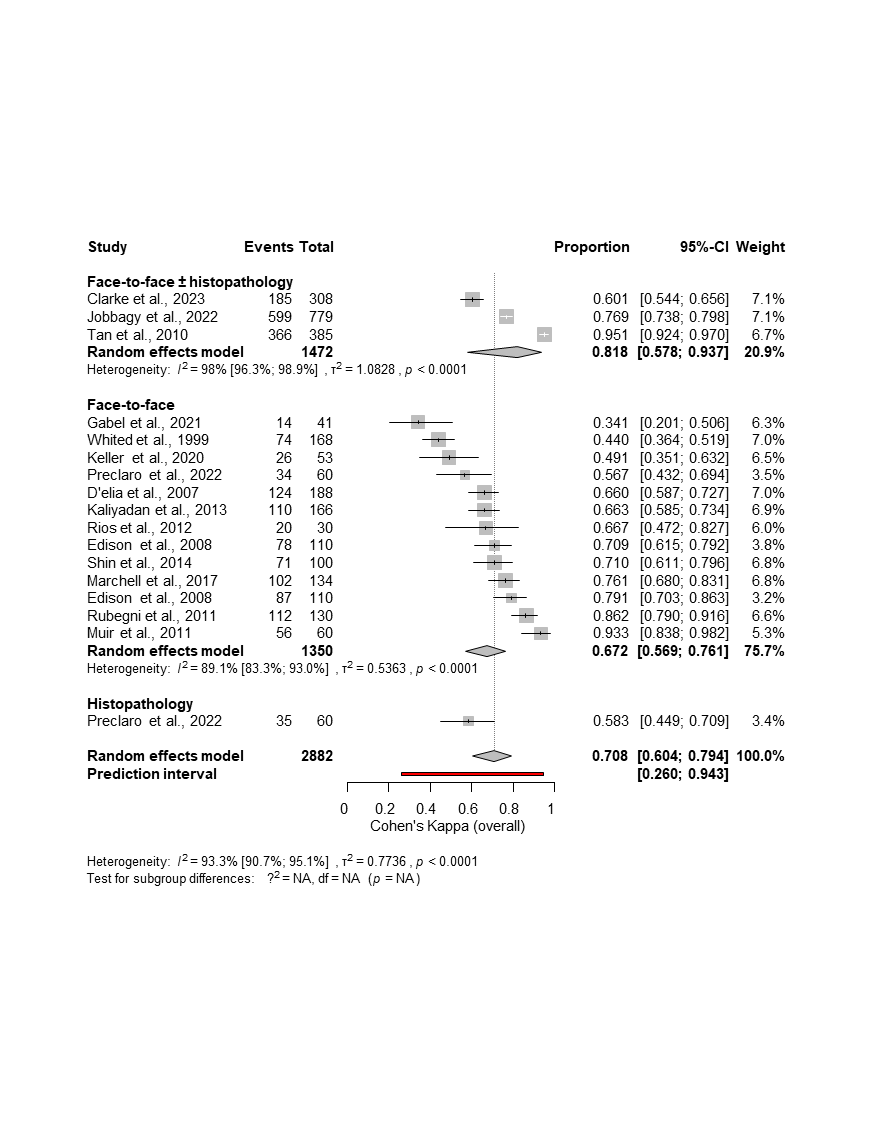


# Supplementary Figure S77: Forest plot comparing Cohen’s kappa in the pairwise comparison of “face-to-face ±histopathology” and “face-to-face” comparators in the “all skin conditions” group, including undiagnosed cases in the analysis.


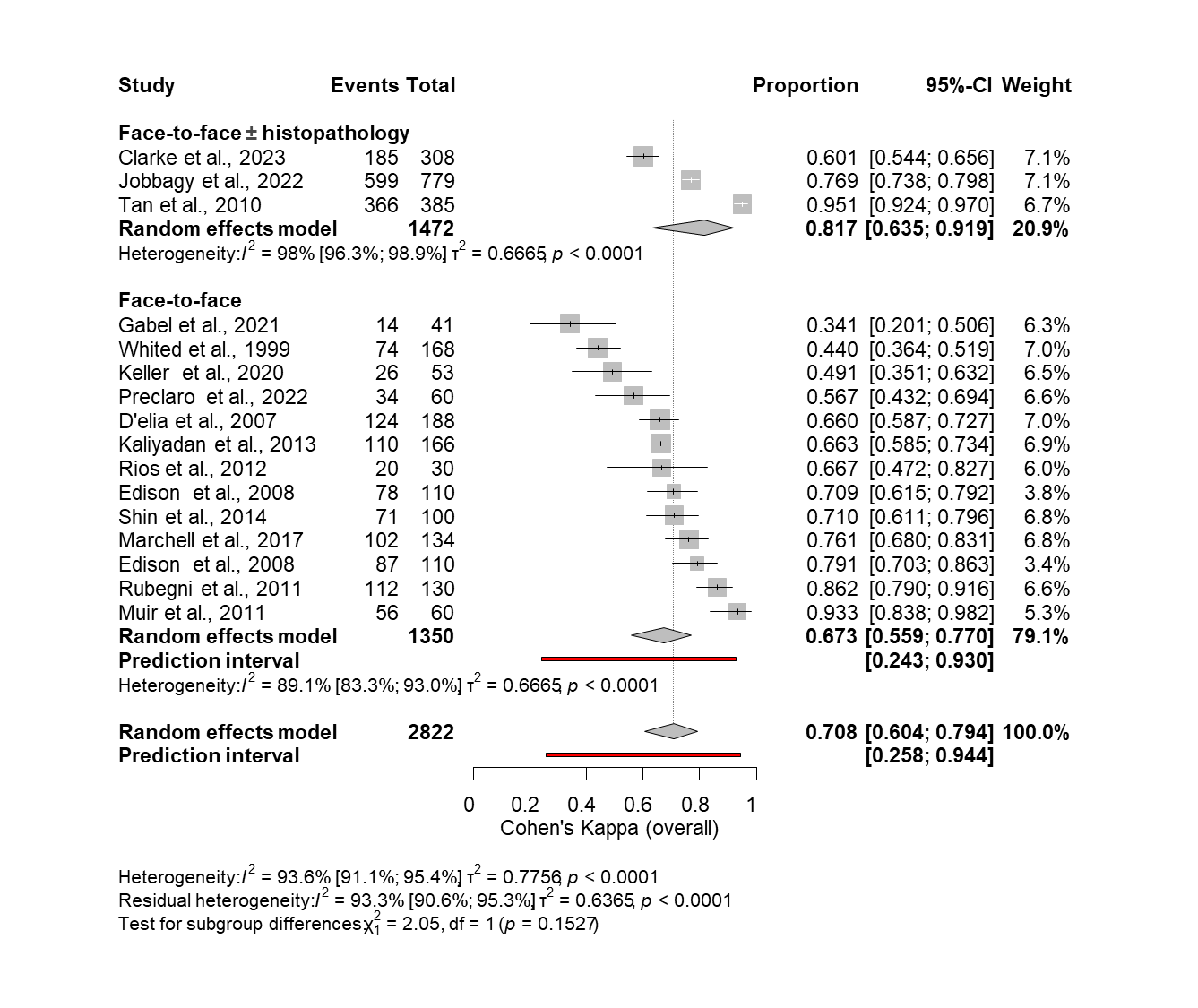


# Supplementary Figure S78: Forest plot comparing Cohen’s kappa between teledermatology providers and in-person dermatologists based on the comparator in the “skin cancer” group, including undiagnosed cases in the analysis.


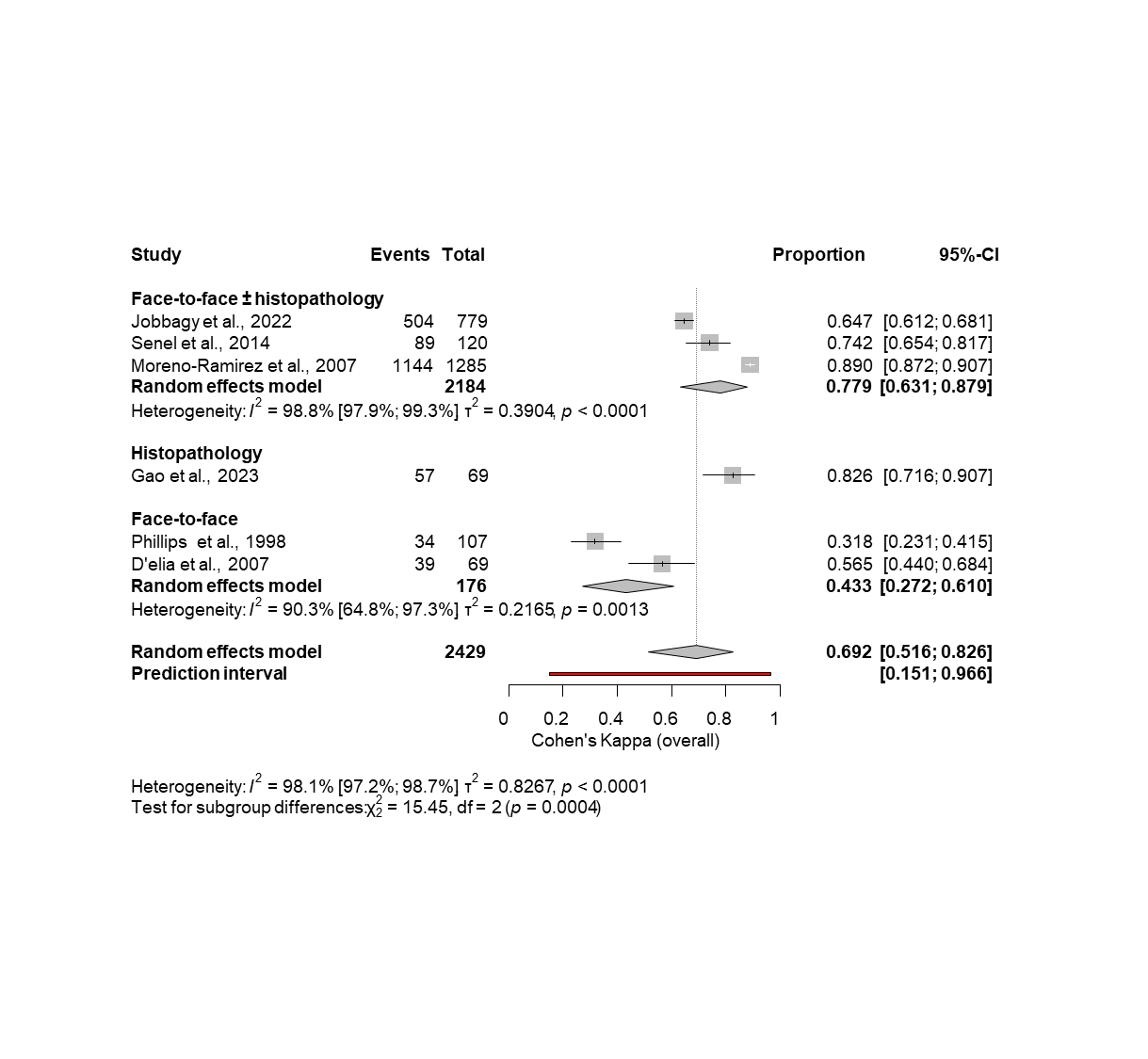


# Supplementary Figure S79: Forest plot comparing Cohen’s kappa between teledermatology providers and in-person dermatologists based on the comparator in the “pigmented lesions” group, including undiagnosed cases in the analysis.


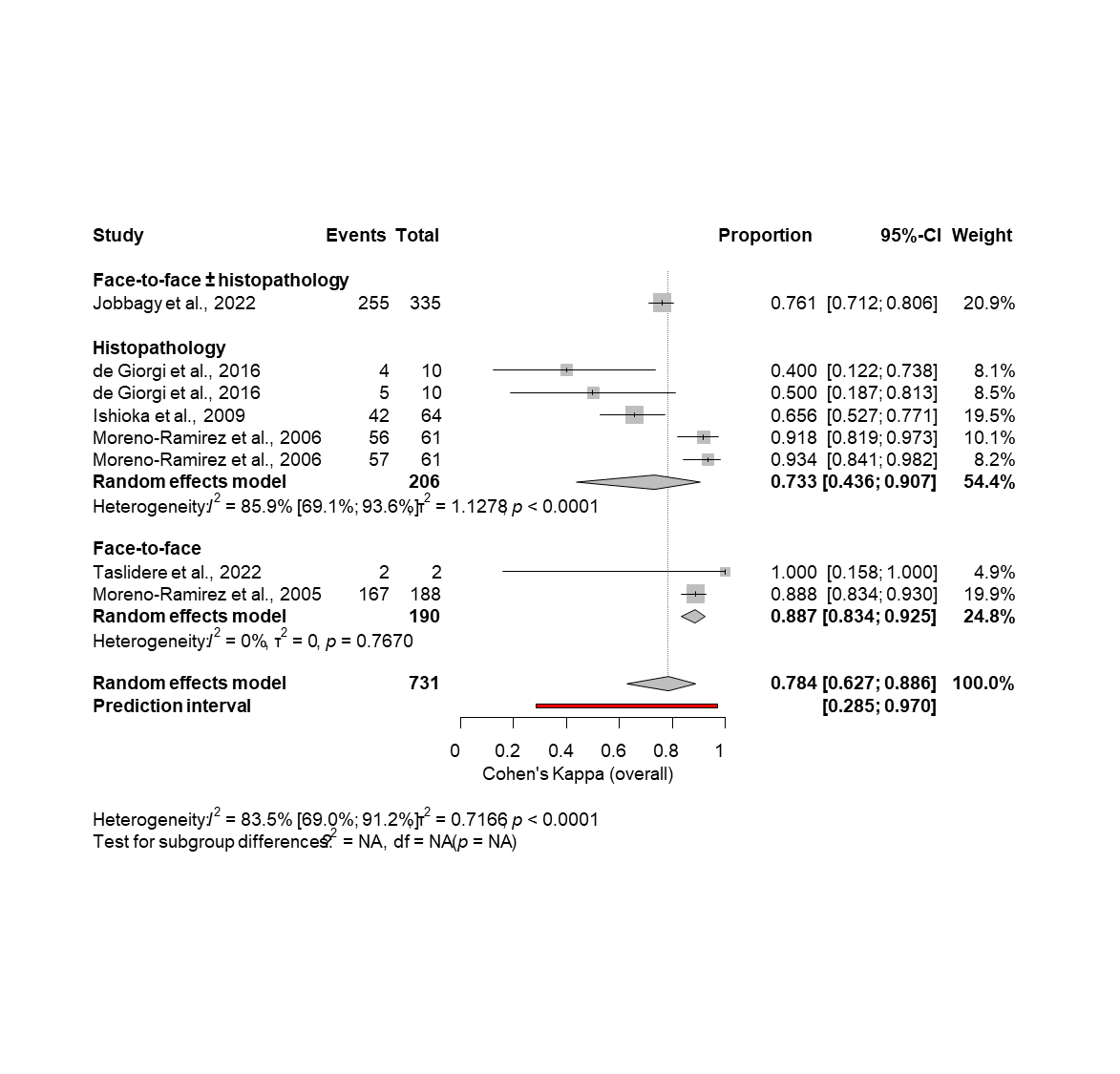


# Supplementary Figure S80: Forest plot for the sensitivity of teledermatology in the “skin cancer” group, including undiagnosed cases in the analysis.


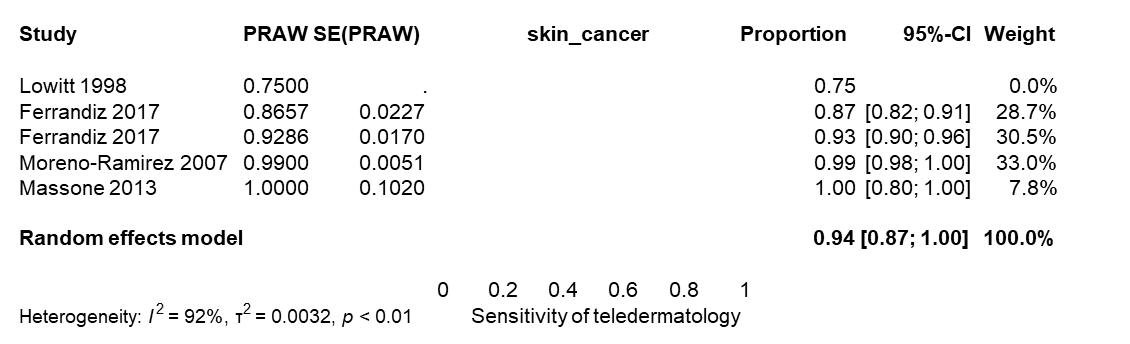


# Supplementary Figure S81: Forest plot for the specificity of teledermatology in the “skin cancer” group, including undiagnosed cases in the analysis.


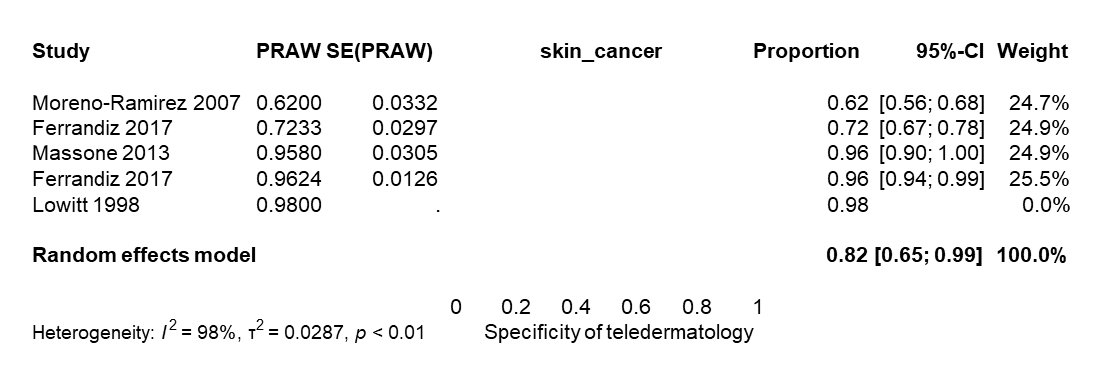


# Supplementary Figure S82: Forest plot for the sensitivity of teledermatology in the “pigmented lesions” group, including undiagnosed cases in the analysis.


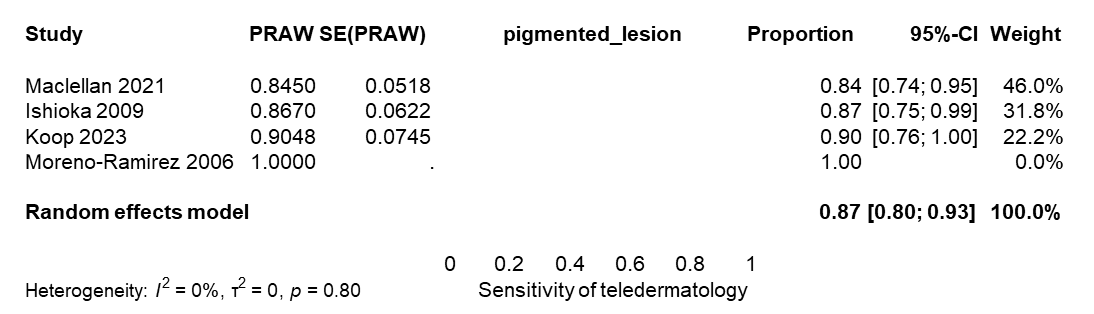


# Supplementary Figure S83: Forest plot for the specificity of teledermatology in the “pigmented lesions” group, including undiagnosed cases in the analysis.


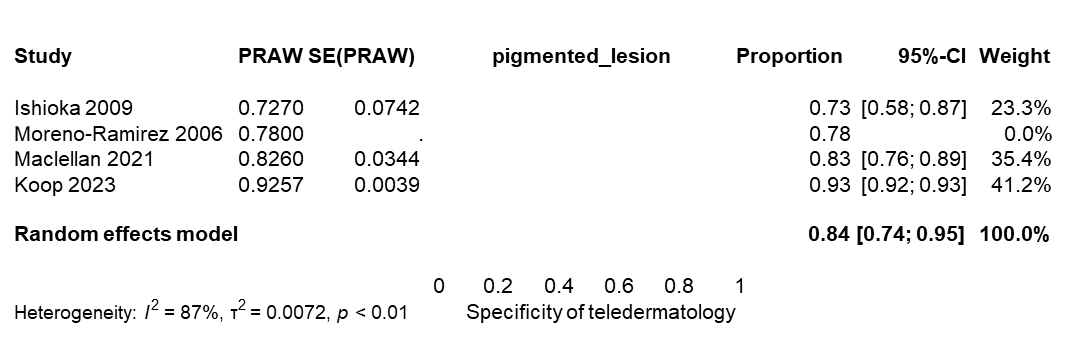


# Supplementary Figure S84: Forest plot for the interrater agreement between teledermatologists and in-person dermatologists, measured subgrouped by Cohen’s kappa.


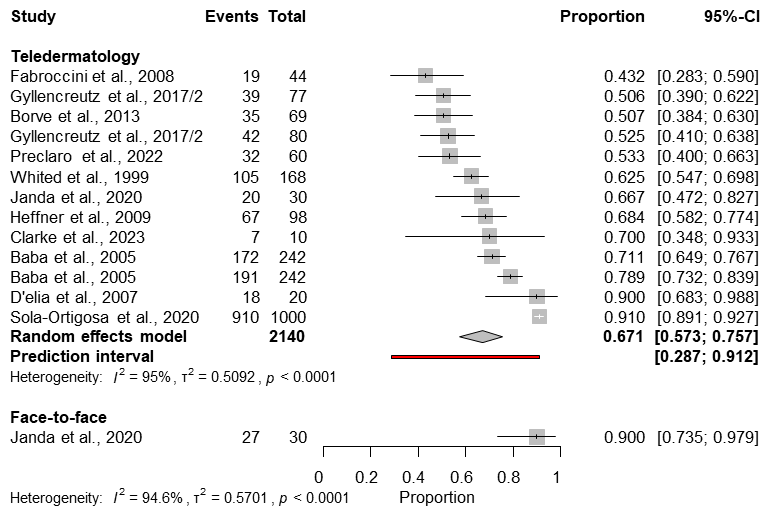


# Supplementary Table S3: Summary of results on satisfaction of teledermatology providers

| **First Author, Year of Publication** | **Number of teledermatology providers** | **Proportion of satisfied teledermatology providers** |
| --- | --- | --- |
| Kaliyadan, 2013^56^ | 2 | 100% |
| Lowitt, 1998^70^ | 4 | 81·0% |

# Supplementary Table S4: Summary of results on diagnostic time during face-to-face dermatology visits

| **First Author, Year of Publication** | **Number of cases** | **Mean time in minutes** |
| --- | --- | --- |
| Ilie, 2022^70^ | 29 | 9·76 |
| Lasierra, 2012^64^ | 82 | 10·0 |
| Nami, 2015^86^ | 391 | 15·0 |
| Vano-Galvan, 2011^128^ | 100 | 5·00 |

# Supplementary Table S5: Summary of results on diagnostic concordance results excluded from the quantitative analyses

| **First Author, Year of publication** | **Subgroup** | **Number of cases** | **Diagnostic concordance (95% CI)** |
| --- | --- | --- | --- |
| Alfageme, 2021^141^ | NA | 147 | 95·7% |
| Creadore, 2023^142^ | History and physical examination* | 10 | 84·0% (4·00%)† |
|  | History, physical examination, cellulitis questionnaire* | 10 | 86·0% (3·00%)† |
|  | History, physical examination, cellulitis questionnaire, thermal images* | 10 | 89·0 (3·00%)† |
| Giavina-Bianchi, 2020/2^143^ | NA | 109 | 84·4%‡ |
| Giavina-Bianchi, 2020/3^144^ | NA | 739 | 78·0% |
| Heffner, 2009^42^ | NA | 137 | 69·0% (60·0%; 77·0%) |
| Lozzi, 2007^145^ | NA | 33 | 78·8% |
| Rao, 2013^148^ | Benign lesions | 255 | 44·0% |
|  | Malignant lesions | 79 | 23·3% |
| Senel, 2013^149^ | Without dermoscopy, teledermatologist A* | 76 | 85·0% |
|  | Without dermoscopy, teledermatologist B* | 76 | 88·0% |
|  | With dermoscopy, teledermatologist A* | 76 | 94·0% |
|  | With dermoscopy, teledermatologist B* | 76 | 96·0% |
| Shah, 2023^150^ | NA | 120 | 78·3% |
| Sola-Ortigosa, 2020^117^ | Without dermoscopy* | 1000 | 92·4% |
|  | With dermoscopy* | 1000 | 97·3% |
| Tian, 2017^126^ | NA | 358 | 95·5% |
| Trindade, 2008^152^ | NA | 106 | 74·0% |
| Tugrul, 2022^153^ | Without dermoscopy* | 26 | 74·3% |
|  | With dermoscopy* | 26 | 82·0% |
| Warshaw, 2009/2^155^ | Without dermoscopy* | 728 | 54·0% |
|  | With dermoscopy* | 728 | 60·8% |

* Subgroups consist of the same/overlapping patient population

†Diagnostic concordance (standard deviation)

‡ Results reported only for analyses excluding undiagnosed cases

CI: confidence interval; NA: not applicable

# Supplementary Table S6: Summary of results on Cohen’s kappa results excluded from the quantitative analyses

| **First Author, Year of publication** | **Subgroup** | **Number of cases** | **Cohen’s kappa (95% CI)** |
| --- | --- | --- | --- |
| Fabbrocini, 2008^29^ | Without dermoscopy* | 44 | 0·36 |
|  | With dermoscopy* | 44 | 0·45 |
| Giavina-Bianchi, 2020/3^144^ | NA | 739 | 0·74 |
| Heffner, 2009^42^ | NA | 137 | 0·65 (0·58; 0·73) |
| Nami, 2015^86^ | NA | 391 | 0·91 (0·88; 0·936) |
| Senel, 2013^149^ | Without dermoscopy, teledermatologist A* | 76 | 0·77 (0·69; 0·85) |
|  | Without dermoscopy, teledermatologist B* | 76 | 0·75 (0·67; 0·83) |
|  | With dermoscopy, teledermatologist A* | 76 | 0·85 (0·79; 0·91) |
|  | With dermoscopy, teledermatologist B* | 76 | 0·86 (0·80; 0·93) |
| van der Heijden, 2013^154^ | NA | 76 | 0·41-0·63 |

* Subgroups consist of the same/overlapping patient population

CI: confidence interval; NA: not applicable

# Supplementary Table S7: Summary of results on the sensitivity and specificity of teledermatology excluded from the quantitative analyses

| **First Author, Year of publication** | **Subgroup** | **Number of cases** | **Sensitivity (95% CI lower; 95% CI upper)** | **Specificity (95% CI)** |
| --- | --- | --- | --- | --- |
| Alfageme, 2021^141^ | Malignant tumors | 147 | 100% | 97·8% |
| Ludzik, 2016/1^147^ | Melanoma | 13 | 100% | NA |
|  | Basal cell carcinoma | 36 | 94·4% | NA |
|  | Naevi | 31 | NA | 78·6% |
| Ludzik, 2016/2^146^ | Dermoscopy* | 173 | 95·9% | 33·6% |
|  | Dermoscopy+RCM* | 173 | 98·3% | 42·7% |
| Rao, 2013^148^ | Reader 1* | 317 | 93·1% | 64·1% |
|  | Reader 2* | 323 | 97·4% | 80·5% |
| Trindade, 2008^152^ | NA | 106 | 78% | 31% |
| Witkowski, 2017^156^ | Melanoma | 83 | 95·2% | 76·3% |

* Subgroups consist of the same/overlapping patient population

CI: confidence interval; NA: not applicable; RCM: reflectance confocal microscopy

# Supplementary Table S8: Summary of results on positive and negative predictive values of teledermatology

| **First Author, Year of Publication** | **Subgroup** | **Number of cases** | **Positive predictive value (95% CI lower; 95% CI upper)** | **Negative predictive value (95% CI)** |
| --- | --- | --- | --- | --- |
| Congalton, 2015^21^ | Suspected melanoma | 129 | 63·0% (51·0%; 74·0% | 96·0% (85·0%; 99·0%) |
| Jobbagy, 2022^52^ | Dysplastic naevi | 38 | 31·6% (19·1%; 47·5%) | 99·6% (98·8%; 99·9%) |
|  | Naevi | 265 | 93·6% (90·0%; 96·0%) | 92·8% (90·2%; 94·7%) |
|  | Seborrheic keratosis | 123 | 91·1% (84·7%; 94·9%) | 95·3% (93·4%; 96·7%) |
|  | Hemangiomas | 67 | 97% (89·8%; 99·5%) | 99·3% (98·4%; 99·7%) |
|  | Warts | 15 | 100% (79·6%; 100%) | 99·5% (98·7%; 99·8%) |
|  | Melanoma | 32 | 31·3% (18·0%; 48·6%) | 99·3% (98·4%; 99·7%) |
|  | Basal cell carcinoma | 98 | 71·4% (61·8%; 79·4%) | 98·8% (97·7%; 99·4%) |
|  | Squamous cell carcinoma | 36 | 36·1% (22·5%; 52·4%) | 98·9% (97·9%; 99·5%) |
|  | Actinic keratosis | 25 | 80·0% (60·9%; 91·1%) | 98·5% (97·4%; 99·2%) |
| Ferrandiz, 2017^31^ | Suspected skin cancer | 228 | 84·4% (79·7%; 89·1%) | 98·2% (96·4%; 99·9%) |
| Gemelas, 2019^35^ | Suspected melanoma | 455 | 15·2% | NA |
| Ishioka, 2009^48^ | Pigmented skin lesions | 64 | 74·3% (57·9%; 85·8%) | 85·7% (68·5%; 94·3%) |
| Koop, 2023^58^ | Suspected melanoma | 4748 | 5·14% (4·36%; 6·04%) | 100% (99·8%; 100%) |
| Lowitt, 1998^70^ | Acneiform lesions | 17 | 67·0% | NA |
|  | Dermatitis | 33 | 92·0% | NA |
|  | Fungal infections | 9 | 100% | NA |
|  | Papulosquamous diseases | 17 | 86·0% | NA |
|  | Benign tumors | 36 | 86·0% | NA |
|  | Premalignant lesions | 16 | 87·0% | NA |
|  | Malignant tumors | 6 | 60·0% | NA |
| Moreno-Ramirez, 2007^83^ | Suspected skin cancer | 403 | 66·0% (60·0%; 71·0%) | 99·0% (98·0%; 100%) |
| Ng, 2011^87^ | Suspected skin cancer (subgroup 1 – “good photographs”) | 50 | 62·6% | 88·2% |
|  | Suspected skin cancer (subgroup 2 – “poor photographs”) | 50 | 43·0% | 94·0% |
| Sola-Ortigosa, 2020^117^ | Actinic keratosis | 64 | 91·0% | 92·1% |
| Teague, 2022^124^ | Suspected melanoma | 591 | 62·0% (56·0%; 68·0%) | NA |
| Teoh, 2022^125^ | Suspected melanoma | 523 | 61·1% | NA |

CI: confidence interval; NA: not applicable

# Supplementary Table S9: Summary of results on Area Under the Curve values of teledermatology

| **First Author, Year of publication** | **Number of cases** | **Average AUC (95% CI)** |
| --- | --- | --- |
| Tognetti, 2021^151^ | 30 | 0·680 (0·641; 0.719)* |

*Results based on intuitive diagnosis, mixed screen dimensions.

# Risk of bias (RoB) assessment

## Supplementary Table S10: Domain-level risk of bias assessments for all included articles

|  | **BIAS** | | | | **APPLICABILITY** | | |  |
| --- | --- | --- | --- | --- | --- | --- | --- | --- |
| **First author and year of publication** | **PATIENT SELECTION** | **INDEX TEST** | **REFERENCE STANDARD** | **FLOW AND TIMING** | **PATIENT SELECTION** | **INDEX TEST** | **REFERENCE STANDARD** |  |
| **Studies included in the meta-analysis** | | | | | | | | |
| Altieri, 2017^1^ | low | low | low | low | low | low | low |  |
| Baba, 2005^2^ | low | low | high | low | low | low | low |  |
| Barbieri, 2014^3^ | unclear | low | low | low | low | low | low |  |
| Barcaui, 2018^4^ | low | low | high | low | low | low | low |  |
| Barnard, 2000^5^ | high | low | low | low | high | low | low |  |
| Batalla, 2016/1^6^ | low | low | low | low | low | low | low |  |
| Batalla, 2016/2^7^ | low | low | unclear | unclear | high | low | high |  |
| Borve, 2012^8^ | low | low | high | high | low | low | low |  |
| Borve, 2013^9^ | low | low | low | low | low | low | low |  |
| Bowns, 2006^10^ | low | low | low | high | low | low | low |  |
| Braun, 2000^11^ | low | low | low | low | low | low | low |  |
| Carter, 2017^12^ | low | low | unclear | high | high | low | low |  |
| Castillo, 2022^13^ | low | low | low | low | high | low | low |  |
| Cazzaniga, 2016^14^ | low | low | unclear | low | high | low | high |  |
| Chan, 2000^15^ | low | low | unclear | low | high | low | low |  |
| Chao, 2003^16^ | unclear | low | high | low | unclear | high | low |  |
| Chen, 2010^17^ | low | high | low | low | low | low | low |  |
| Cheung, 2018^18^ | low | low | unclear | high | high | low | low |  |
| Chung, 2007^19^ | high | low | low | low | high | high | high |  |
| Clarke, 2023^20^ | high | low | low | low | high | low | low |  |
| Congalton, 2015^21^ | low | low | low | low | high | low | low |  |
| Coras, 2003^22^ | unclear | low | low | low | low | low | low |  |
| D'elia, 2007^23^ | low | low | low | low | low | low | low |  |
| de Giorgi, 2016^24^ | high | low | low | low | high | low | low |  |
| Dobry, 202^25^ | low | low | low | unclear | high | low | low |  |
| Du Moulin, 2003^26^ | low | low | low | low | low | low | low |  |
| Edison, 2008^27^ | low | low | low | low | low | low | low |  |
| Eminovic, 2003^28^ | low | low | low | low | low | low | low |  |
| Fabbrocini, 2008^29^ | low | low | low | low | high | low | low |  |
| Faucon, 2022^30^ | low | low | low | high | low | low | low |  |
| Ferrandiz, 2017^31^ | low | low | low | low | low | low | low |  |
| Gabel, 2021^32^ | low | low | low | high | low | low | low |  |
| Gao, 2023^33^ | low | low | unclear | high | high | low | high |  |
| Gatica, 2015^34^ | low | low | low | low | low | low | low |  |
| Gemelas, 2019^35^ | low | low | unclear | high | high | high | high |  |
| Gerhardt, 2021^36^ | low | low | unclear | high | high | low | high |  |
| Giavina-Bianchi, 2020/1^37^ | low | low | unclear | unclear | high | low | high |  |
| Gilmour, 1998^38^ | low | low | low | low | low | low | low |  |
| Gyllencreutz, 2017^39^ | low | low | low | high | low | low | low |  |
| Gyllencreutz, 2018^40^ | high | low | low | low | high | high | low |  |
| Harrison, 1998^41^ | low | low | unclear | low | high | low | high |  |
| Heffner, 2009^42^ | low | low | low | low | high | low | low |  |
| Herrmann, 2005^43^ | low | low | high | low | low | low | low |  |
| High, 2000^44^ | low | low | low | high | high | low | low |  |
| Hines, 2021^45^ | low | low | low | high | high | low | low |  |
| Hue, 2015^46^ | low | low | low | high | high | low | low |  |
| Ilie, 2022^47^ | low | low | unclear | high | low | high | high |  |
| Ishioka, 2009^48^ | high | low | low | low | high | low | low |  |
| Fazil Jaber, 2023^49^ | low | low | low | low | high | low | low |  |
| Janda, 2020^50^ | low | low | low | low | high | low | low |  |
| Jang, 2002^51^ | unclear | low | low | low | low | low | low |  |
| Jobbagy, 2022^52^ | low | low | unclear | high | low | low | low |  |
| Jolliffe, 2001^53^ | low | low | high | high | low | low | low |  |
| Jones, 2021^54^ | low | low | unclear | low | high | low | high |  |
| Josendal, 1991^55^ | unclear | low | unclear | low | high | low | high |  |
| Kaliyadan, 2013^56^ | low | low | low | low | low | low | low |  |
| Keller, 2020^57^ | low | low | low | low | low | low | low |  |
| Koop, 2023^58^ | low | low | high | high | high | low | low |  |
| Kravets, 2018^59^ | low | low | low | low | low | low | low |  |
| Kroemer, 2011^60^ | low | low | low | low | low | low | low |  |
| Krupinski, 1999^61^ | low | high | low | low | low | low | low |  |
| Kvedar, 1997^62^ | high | low | low | low | high | low | low |  |
| Lamel, 2012^63^ | low | low | low | low | low | low | low |  |
| Lasierra, 2012^64^ | low | low | high | high | low | low | low |  |
| Lepe, 2004^65^ | low | low | low | low | low | low | low |  |
| Lesher, 1998^66^ | low | low | low | low | low | low | low |  |
| Lim, 2001^67^ | low | low | low | low | low | low | low |  |
| Loane, 1997^68^ | low | low | high | high | low | low | low |  |
| Loane, 1998^69^ | low | low | low | high | low | low | low |  |
| Lowitt, 1998^70^ | low | low | high | low | high | high | low |  |
| Lyon, 1997^71^ | low | high | low | low | low | low | low |  |
| Maclellan, 2021^72^ | high | low | low | low | high | low | low |  |
| Mahendran, 2005^73^ | low | low | low | low | low | low | low |  |
| Mallett, 2003^74^ | high | unclear | unclear | unclear | high | low | low |  |
| Manahan, 2015^75^ | low | low | high | high | low | low | low |  |
| Marchell, 2017^76^ | low | low | low | low | low | low | low |  |
| Markun, 2017^77^ | low | low | low | high | low | low | low |  |
| Massone, 2007^78^ | unclear | low | low | low | high | low | low |  |
| Massone, 2013^79^ | high | low | low | high | high | low | low |  |
| Montejano, 2022^80^ | low | low | low | low | high | low | low |  |
| Moreno-Ramirez, 2005^81^ | low | low | low | low | high | low | low |  |
| Moreno-Ramirez, 2006^82^ | low | low | low | low | low | low | low |  |
| Moreno-Ramirez, 2007^83^ | low | high | high | high | high | low | low |  |
| Muir, 2011^84^ | high | low | high | high | high | low | low |  |
| Naka, 2018^85^ | high | low | unclear | high | high | low | high |  |
| Nami, 2015^86^ | high | low | low | high | low | low | low |  |
| Ng, 2011^87^ | low | low | low | low | low | low | low |  |
| Nordal, 2001^88^ | high | low | unclear | high | high | high | high |  |
| Norton, 1997^89^ | low | low | low | low | high | high | low |  |
| O'Connor, 2017^90^ | low | low | low | low | low | low | low |  |
| Oakley, 1997^91^ | low | low | high | low | low | low | low |  |
| Oakley, 1998^92^ | low | low | unclear | unclear | high | low | high |  |
| Oakley, 2006^93^ | high | low | low | low | high | low | low |  |
| Okita, 2016^94^ | low | low | low | low | high | low | low |  |
| Oztas, 2004^95^ | low | low | low | low | low | low | low |  |
| Pak, 2003^96^ | low | low | low | low | low | low | low |  |
| Paradela-De-La-Morena, 2015^97^ | low | low | low | high | high | high | low |  |
| Phillips, 1997^98^ | low | low | low | high | low | low | low |  |
| Phillips, 1998^99^ | low | low | low | low | low | high | high |  |
| Preclaro, 2022^100^ | low | low | low | low | low | low | low |  |
| Rajagopal, 2009^101^ | low | low | low | low | high | low | low |  |
| Rashid, 2003^102^ | high | low | low | low | high | low | low |  |
| Ribas, 2010^103^ | low | low | low | low | low | low | low |  |
| Rios, 2012^104^ | low | low | low | high | high | high | low |  |
| Romero, 2006^105^ | low | low | low | high | low | low | low |  |
| Romero, 2010^106^ | low | low | low | low | high | low | low |  |
| Romero Aguilera, 2014^107^ | low | low | high | low | low | low | low |  |
| Rubegni, 2011^108^ | low | low | low | low | high | low | low |  |
| Ruiz, 2009^109^ | low | low | high | high | low | high | high |  |
| Saleh, 2017^110^ | low | low | low | low | low | high | low |  |
| Santosa, 2023^111^ | low | low | high | low | high | low | low |  |
| Schiener, 2001^112^ | low | low | low | low | low | low | low |  |
| Senel, 2014^113^ | low | low | low | low | low | low | low |  |
| Shin, 2014^114^ | low | low | low | low | high | low | low |  |
| Silva, 2009^115^ | low | low | low | low | low | low | low |  |
| Silveira, 2019^116^ | unclear | low | low | high | low | low | low |  |
| Sola-Ortigosa, 2020^117^ | low | high | low | low | low | high | high |  |
| Taberner Ferrer, 2009^118^ | low | low | low | high | high | low | low |  |
| Tait, 1999^119^ | low | low | high | low | low | low | low |  |
| Tan, 2010^120^ | low | high | high | high | low | low | low |  |
| Taslidere, 2022^121^ | low | low | low | low | low | low | low |  |
| Taslidere, 2023^122^ | low | low | low | low | low | low | low |  |
| Taylor, 2001^123^ | low | low | low | low | low | low | low |  |
| Teague, 2022^124^ | high | low | low | low | high | low | low |  |
| Teoh, 2022^125^ | low | unclear | low | low | high | high | low |  |
| Tian, 2017^126^ | high | low | low | low | high | low | low |  |
| Tucker, 2005^127^ | low | low | low | low | low | low | low |  |
| Vano-Galvan, 2011^128^ | low | low | low | low | low | high | low |  |
| Villa, 2020^129^ | high | low | high | low | high | low | low |  |
| Wang, 2017^130^ | low | unclear | unclear | unclear | high | high | high |  |
| Warshaw, 2009/1^131^ | low | low | low | low | high | low | low |  |
| Warshaw, 2015^132^ | low | low | low | low | high | low | low |  |
| Weingast, 2013^133^ | low | low | low | low | low | low | low |  |
| Whited, 1998^134^ | low | low | low | low | low | high | low |  |
| Whited, 1999^135^ | low | low | low | low | low | low | low |  |
| Yamazaki, 2003^136^ | unclear | low | unclear | unclear | high | low | high |  |
| Zanini, 2013^137^ | low | low | low | low | low | low | low |  |
| Zelickson, 1997^138^ | low | low | low | low | high | low | low |  |
| Zink, 2017/1^139^ | low | low | low | low | low | low | low |  |
| Zink, 2017/2^140^ | low | low | low | low | low | high | low |  |
| **Studies included in the systematic review** | | | | | | | |  |
| Alfageme, 2021^141^ | high | low | low | high | high | high | low |  |
| Creadore, 2023^142^ | low | low | low | low | high | high | low |  |
| Giavina-Bianchi, 2020/2^143^ | low | low | unclear | unclear | high | low | high |  |
| Giavina-Bianchi, 2020/3^144^ | low | low | unclear | unclear | high | low | high |  |
| Lozzi, 2007^145^ | high | low | high | high | high | low | low |  |
| Ludzik, 2016/1^146^ | low | low | low | low | high | low | low |  |
| Ludzik, 2016/2^147^ | low | low | low | low | high | low | low |  |
| Rao, 2013^148^ | low | low | low | low | high | low | low |  |
| Senel, 2013^149^ | low | low | low | low | low | high | low |  |
| Shah, 2023^150^ | low | low | unclear | high | high | low | high |  |
| Tognetti, 2021^151^ | unclear | low | low | unclear | high | high | low |  |
| Trindade, 2008^152^ | low | low | low | low | high | low | low |  |
| Tugrul, 2022^153^ | low | low | low | low | low | high | low |  |
| Van der Heijden, 2013^154^ | low | low | high | high | high | low | low |  |
| Warshaw, 2009/2^155^ | low | low | low | low | low | low | low |  |
| Witkowski, 2017^156^ | high | low | low | low | high | high | low |  |

## Supplementary Figure S85: Distribution of risk of bias judgements within bias domains


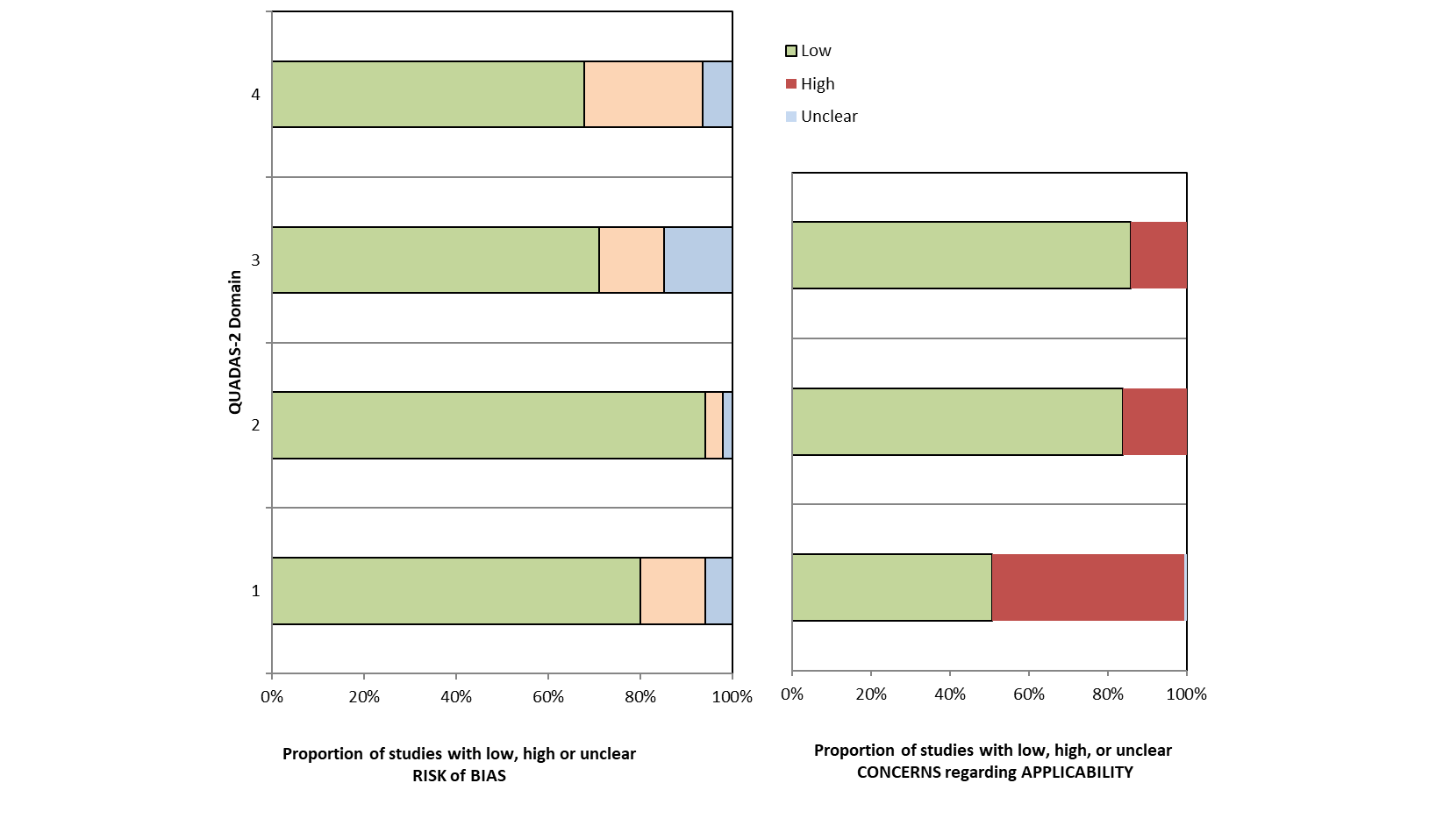


# **Publication bias**

## Supplementary Figure S86: Funnel plot for studies in the “all skin conditions” group assessing diagnostic concordance, including undiagnosed cases in the analysis.

## Supplementary Figure S87: Funnel plot for studies in the “all skin conditions” group assessing diagnostic concordance, excluding undiagnosed cases from the analysis.

## Supplementary Figure S88: Funnel plot for studies in the “all skin conditions” group assessing Cohen’s kappa, including undiagnosed cases in the analysis.

## Supplementary Figure S89: Funnel plot for studies in the “all skin conditions” group assessing Cohen’s kappa, excluding undiagnosed cases from the analysis.

## Supplementary Figure S90: Funnel plot for studies in the “skin cancer” group assessing diagnostic concordance, including undiagnosed cases in the analysis.

## Supplementary Figure S91: Funnel plot for studies in the “skin cancer” group assessing diagnostic concordance, excluding undiagnosed cases from the analysis.

## Supplementary Figure S92: Funnel plot for studies in the “pigmented lesions” group assessing diagnostic concordance, including undiagnosed cases in the analysis.

## Supplementary Figure S93: Funnel plot for studies in the “pigmented lesions” group assessing diagnostic concordance, excluding undiagnosed cases from the analysis.

## Supplementary Figure S94: Funnel plot for studies in the “pigmented lesions” group assessing Cohen’s kappa, excluding undiagnosed cases from the analysis.

## Supplementary Figure S95: Funnel plot assessing diagnostic concordance for interrater agreement.


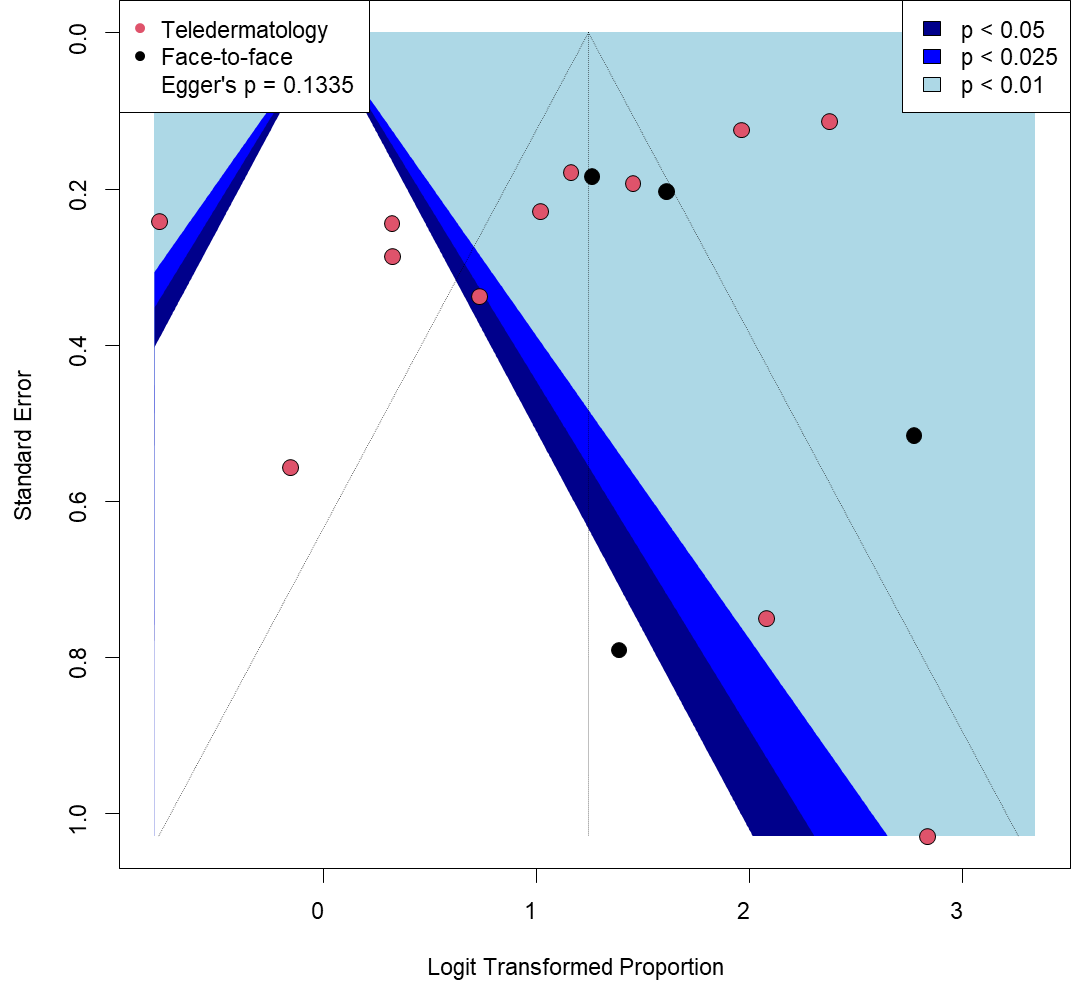


## Supplementary Figure S96: Funnel plot assessing Cohen’s kappa for interrater agreement.


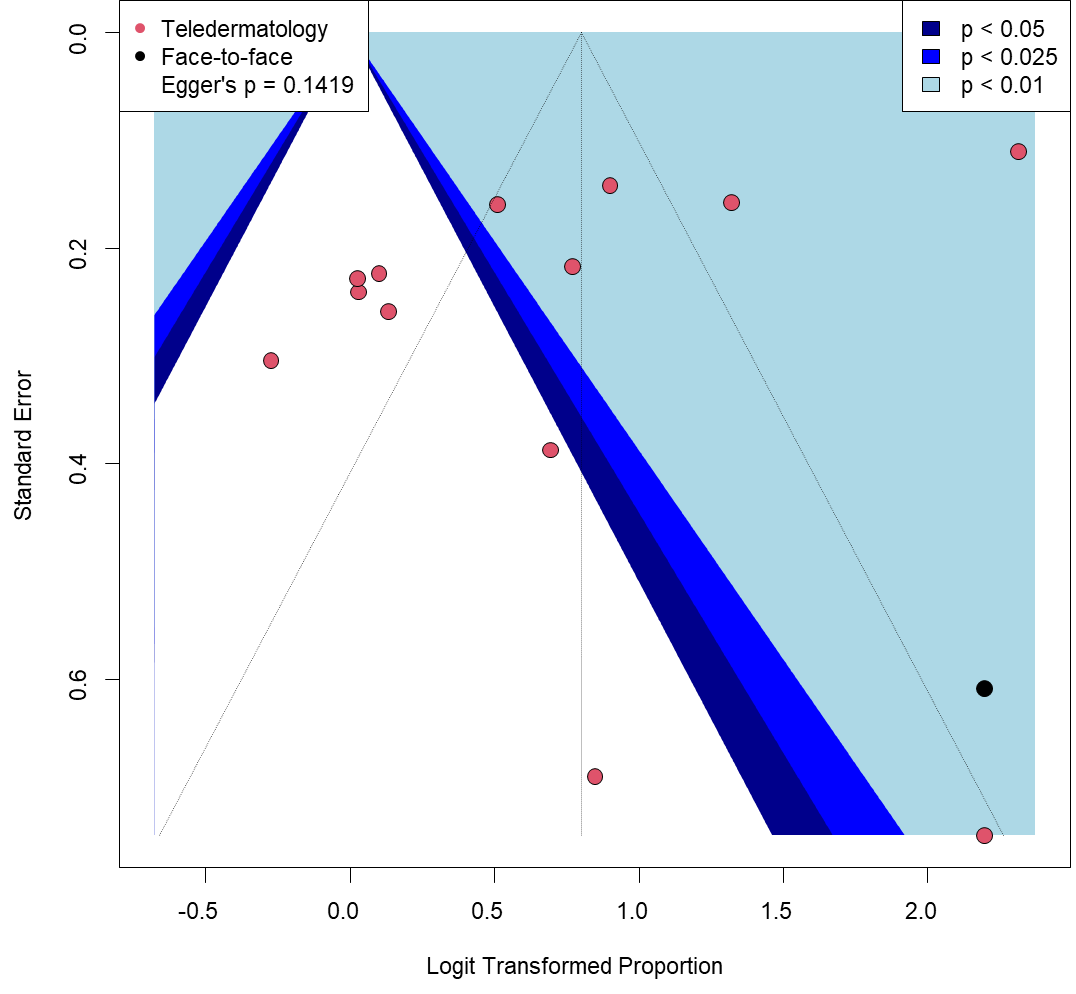


# References

1. Altieri L, Hu J, Nguyen A, Cockburn M, Chiu M, Cotliar J, et al. Interobserver reliability of teledermatology across all Fitzpatrick skin types. J Telemed Telecare. 2017;23(1):68-73.

2. Baba M, Seçkin D, Kapdağli S. A comparison of teledermatology using store-and-forward methodology alone, and in combination with Web camera videoconferencing. J Telemed Telecare. 2005;11(7):354-60.

3. Barbieri JS, Nelson CA, James WD, Margolis DJ, Littman-Quinn R, Kovarik CL, et al. The reliability of teledermatology to triage inpatient dermatology consultations. JAMA Dermatol. 2014;150(4):419-24.

4. Barcaui CB, Lima PMO. Application of Teledermoscopy in the Diagnosis of Pigmented Lesions. Int J Telemed Appl. 2018;2018:1624073.

5. Barnard CM, Goldyne ME. Evaluation of an asynchronous teleconsultation system for diagnosis of skin cancer and other skin diseases. Telemed J E Health. 2000;6(4):379-84.

6. Batalla A, Suh-Oh HJ, Abalde T, Salgado-Boquete L, de la Torre C. [Teledermatology in Paediatrics. Observations in daily clinical practice]. An Pediatr (Barc). 2016;84(6):324-30.

7. Batalla A, Suh-Oh HJ, Salgado-Boquete L, Abalde T, De La Torre C. Teledermatology: Effectiveness in reducing face-to-face visits according to the disease group. Piel. 2016;31(3):156-63.

8. Börve A, Holst A, Gente-Lidholm A, Molina-Martinez R, Paoli J. Use of the mobile phone multimedia messaging service for teledermatology. J Telemed Telecare. 2012;18(5):292-6.

9. Börve A, Terstappen K, Sandberg C, Paoli J. Mobile teledermoscopy-there's an app for that! Dermatol Pract Concept. 2013;3(2):41-8.

10. Bowns IR, Collins K, Walters SJ, McDonagh AJ. Telemedicine in dermatology: a randomised controlled trial. Health Technol Assess. 2006;10(43):iii-iv, ix-xi, 1-39.

11. Braun RP, Meier ML, Pelloni F, Ramelet AA, Schilling M, Tapernoux B, et al. Teledermatoscopy in Switzerland: A preliminary evaluation. Journal of the American Academy of Dermatology. 2000;42(5):770-5.

12. Carter ZA, Goldman S, Anderson K, Li X, Hynan LS, Chong BF, et al. Creation of an Internal Teledermatology Store-and-Forward System in an Existing Electronic Health Record: A Pilot Study in a Safety-Net Public Health and Hospital System. JAMA Dermatol. 2017;153(7):644-50.

13. Castillo F, Peracca S, Oh DH, Twigg AR. The Utilization and Impact of Live Interactive and Store-and-Forward Teledermatology in a Veterans Affairs Medical Center During the COVID-19 Pandemic. Telemed J E Health. 2022;28(8):1186-92.

14. Cazzaniga S, Castelli E, Di Landro A, Zucchi A, Naldi L. [Development of a teledermatology system for the melanoma diagnosis. The pilot experience of the project Clicca il neo]. Recenti Prog Med. 2016;107(8):440-3.

15. Chan HHL, Woo J, Chan WM, Hjelm M. Teledermatology in Hong Kong: A cost-effective method to provide service to the elderly patients living in institutions. International Journal of Dermatology. 2000;39(10):774-8.

16. Chao LW, Cestari TF, Bakos L, Oliveira MR, Miot HA, Zampese M, et al. Evaluation of an Internet-based teledermatology system. J Telemed Telecare. 2003;9:S9-12.

17. Chen TS, Goldyne ME, Mathes EFD, Frieden IJ, Gilliam AE. Pediatric teledermatology: observations based on 429 consults. J Am Acad Dermatol. 2010;62(1):61-6.

18. Cheung CMM, Muttardi K, Chinthapalli S, Ismail F. Pilot Teledermatology Service for Assessing Solitary Skin Lesions in a Tertiary London Dermatology Center. Journal for Healthcare Quality. 2019;41(1):E1-E6.

19. Chung P, Yu T, Scheinfeld N. Using cellphones for teledermatology, a preliminary study. Dermatol Online J. 2007;13(3):2.

20. Clarke EL, Reichenberg JS, Ahmed AM, Keeling B, Custer J, Rathouz PJ, et al. The utility of teledermatology in the evaluation of skin lesions. J Telemed Telecare. 2023;29(5):382-9.

21. Congalton AT, Oakley AM, Rademaker M, Bramley D, Martin RCW. Successful melanoma triage by a virtual lesion clinic (teledermatoscopy). Journal of the European Academy of Dermatology and Venereology. 2015;29(12):2423-8.

22. Coras B, Glaessl A, Kinateder J, Klövekorn W, Braun R, Lepski U, et al. Teledermatoscopy in daily routine--results of the first 100 cases. Curr Probl Dermatol. 2003;32:207-12.

23. D'Elia PB, Harzheim E, Fisher PD, Ramos MC, Bordin R. Agreement between dermatological diagnoses made by direct observation and digital images. Anais Brasileiros de Dermatologia. 2007;82(6):521-7.

24. de Giorgi V, Gori A, Savarese I, D'Errico A, Grazzini M, Papi F, et al. Teledermoscopy in doubtful melanocytic lesions: is it really useful? Int J Dermatol. 2016;55(10):1119-23.

25. Dobry A, Begaj T, Mengistu K, Sinha S, Droms R, Dunlap R, et al. Implementation and Impact of a Store-and-Forward Teledermatology Platform in an Urban Academic Safety-Net Health Care System. Telemed J E Health. 2021;27(3):308-15.

26. Du Moulin MF, Bullens-Goessens YI, Henquet CJ, Brunenberg DE, de Bruyn-Geraerds DP, Winkens RA, et al. The reliability of diagnosis using store-and-forward teledermatology. J Telemed Telecare. 2003;9(5):249-52.

27. Edison KE, Ward DS, Dyer JA, Lane W, Chance L, Hicks LL. Diagnosis, diagnostic confidence, and management concordance in live-interactive and store-and-forward teledermatology compared to in-person examination. Telemed J E Health. 2008;14(9):889-95.

28. Eminović N, Witkamp L, Ravelli AC, Bos JD, van den Akker TW, Bousema MT, et al. Potential effect of patient-assisted teledermatology on outpatient referral rates. J Telemed Telecare. 2003;9(6):321-7.

29. Fabbrocini G, Balato A, Rescigno O, Mariano M, Scalvenzi M, Brunetti B. Telediagnosis and face-to-face diagnosis reliability for melanocytic and non-melanocytic 'pink' lesions. J Eur Acad Dermatol Venereol. 2008;22(2):229-34.

30. Faucon C, Gribi D, Courvoisier DS, Senet P, Itani O, Barbaud A, et al. Performance accuracy, advantages and limitations of a store-and-forward teledermatology platform developed for general practitioners: A retrospective study of 298 cases. Ann Dermatol Venereol. 2022;149(4):245-50.

31. Ferrándiz L, Ojeda-Vila T, Corrales A, Martín-Gutiérrez FJ, Ruíz-de-Casas A, Galdeano R, et al. Internet-based skin cancer screening using clinical images alone or in conjunction with dermoscopic images: A randomized teledermoscopy trial. J Am Acad Dermatol. 2017;76(4):676-82.

32. Gabel CK, Nguyen E, Karmouta R, Liu KJ, Zhou G, Alloo A, et al. Use of teledermatology by dermatology hospitalists is effective in the diagnosis and management of inpatient disease. J Am Acad Dermatol. 2021;84(6):1547-53.

33. Gao JL, Oakley A. Teledermatology for Enhancing Skin Cancer Diagnosis and Management: Retrospective Chart Review. JMIR Dermatol. 2023;6:e45430.

34. Gatica JL, Bertoló S, Morales E, Espinoza M, Contreras C. Store-and-forward teledermatogy in Chile: A contribution to primary health care. Piel. 2015;30(3):148-54.

35. Gemelas J, Capulong D, Lau C, Mata-Diaz S, Raugi GJ. Positive Predictive Value of Melanoma Diagnosis in Store-and-Forward Teledermatology. Telemed J E Health. 2019;25(8):701-7.

36. Gerhardt CA, Foels R, Grewe S, Baldwin BT. Assessing the Diagnostic Accuracy of Teledermatology Consultations at a Local Veterans Affairs Dermatology Clinic. Cureus. 2021;13(6):e15406.

37. Giavina-Bianchi M, Azevedo MFD, Sousa RM, Cordioli E. Part II: Accuracy of Teledermatology in Skin Neoplasms. Front Med (Lausanne). 2020;7:598903.

38. Gilmour E, Campbell SM, Loane MA, Esmail A, Griffiths CEM, Roland MO, et al. Comparison of teleconsultations and face-to-face consultations: Preliminary results of a United Kingdom multicentre teledermatology study. British Journal of Dermatology. 1998;139(1):81-7.

39. Dahlén Gyllencreutz J, Paoli J, Bjellerup M, Bucharbajeva Z, Gonzalez H, Nielsen K, et al. Diagnostic agreement and interobserver concordance with teledermoscopy referrals. J Eur Acad Dermatol Venereol. 2017;31(5):898-903.

40. Dahlén Gyllencreutz J, Johansson Backman E, Terstappen K, Paoli J. Teledermoscopy images acquired in primary health care and hospital settings – a comparative study of image quality. Journal of the European Academy of Dermatology and Venereology. 2018;32(6):1038-43.

41. Harrison PV, Kirby B, Dickinson Y, Schofield R. Teledermatology--high technology or not? J Telemed Telecare. 1998;4:31-2.

42. Heffner VA, Lyon VB, Brousseau DC, Holland KE, Yen K. Store-and-forward teledermatology versus in-person visits: a comparison in pediatric teledermatology clinic. J Am Acad Dermatol. 2009;60(6):956-61.

43. Herrmann FE, Sönnichsen K, Blum A. [Teledermatology versus consultations--a comparative study of 120 consultations]. Hautarzt. 2005;56(10):942-8.

44. High WA, Houston MS, Calobrisi SD, Drage LA, McEvoy MT. Assessment of the accuracy of low-cost store-and-forward teledermatology consultation. J Am Acad Dermatol. 2000;42(5):776-83.

45. Hines AS, Zayas J, Wetter DA, Bridges AG, Camilleri MJ, McEvoy MT, et al. Retrospective analysis of 450 emergency department dermatology consultations: An analysis of in-person and teledermatology consultations from 2015 to 2019. J Telemed Telecare. 2021:1357633x211024844.

46. Hue L, Makhloufi S, Sall N'Diaye P, Blanchet-Bardon C, Sulimovic L, Pomykala F, et al. Real-time mobile teledermoscopy for skin cancer screening targeting an agricultural population: an experiment on 289 patients in France. Journal of the European Academy of Dermatology and Venereology. 2016;30(1):20-4.

47. Ilie PC, Stefanescu S, Aldridge J, Gaffney P, Belcher J, Smith L. Value of DermaCheckup as a novel dedicated digital health solution for teledermatology. Med Int (Lond). 2022;2(1):5.

48. Ishioka P, Tenório JM, Lopes PRL, Yamada S, Michalany NS, Amaral MB, et al. A comparative study of teledermatoscopy and face-to-face examination of pigmented skin lesions. Journal of Telemedicine and Telecare. 2009;15(5):221-5.

49. Fazil Jaber N, Jerkovic Gulin S, Seifert O. Analysis of Teledermoscopy and Face-to-Face Examination of Atypical Pigmented Lesions: a Cross-Sectional, Retrospective Study. Dermatol Pract Concept. 2023;13(3).

50. Janda M, Horsham C, Vagenas D, Loescher LJ, Gillespie N, Koh U, et al. Accuracy of mobile digital teledermoscopy for skin self-examinations in adults at high risk of skin cancer: an open-label, randomised controlled trial. Lancet Digit Health. 2020;2(3):e129-e37.

51. Jang SJ, Lee YS, Chun DK. Diagnostic agreement between teleconsultation and clinic-based consultation in dermatology. Korean Journal of Dermatology. 2002;40(11):1339-45.

52. Jobbágy A, Kiss N, Meznerics FA, Farkas K, Plázár D, Bozsányi S, et al. Emergency Use and Efficacy of an Asynchronous Teledermatology System as a Novel Tool for Early Diagnosis of Skin Cancer during the First Wave of COVID-19 Pandemic. International Journal of Environmental Research and Public Health. 2022;19(5).

53. Jolliffe VM, Harris DW, Whittaker SJ. Can we safely diagnose pigmented lesions from stored video images? A diagnostic comparison between clinical examination and stored video images of pigmented lesions removed for histology. Clin Exp Dermatol. 2001;26(1):84-7.

54. Jones L, Jameson M, Oakley A. Remote Skin Cancer Diagnosis: Adding Images to Electronic Referrals Is More Efficient Than Wait-Listing for a Nurse-Led Imaging Clinic. Cancers (Basel). 2021;13(22).

55. Jøsendal O, Fosse G, Andersen KA, Stenvold SE, Falk ES. [Distance diagnosis of skin diseases]. Tidsskr Nor Laegeforen. 1991;111(1):20-2.

56. Kaliyadan F, Amin TT, Kuruvilla J, Ali WH. Mobile teledermatology--patient satisfaction, diagnostic and management concordance, and factors affecting patient refusal to participate in Saudi Arabia. J Telemed Telecare. 2013;19(6):315-9.

57. Keller JJ, Johnson JP, Latour E. Inpatient teledermatology: Diagnostic and therapeutic concordance among a hospitalist, dermatologist, and teledermatologist using store-and-forward teledermatology. J Am Acad Dermatol. 2020;82(5):1262-7.

58. Koop C, Kruus P, Hallik R, Lehemets H, Vettus E, Niin M, et al. A country-wide teledermatoscopy service in Estonia shows results comparable to those in experimental settings in management plan development and diagnostic accuracy: A retrospective database study. JAAD Int. 2023;12:81-9.

59. Kravets K, Vasylenko O, Dranyk Z, Bogomolets O. Store-and-forward teledermatology for the most common skin neoplasms in Ukraine. Acta Dermatovenerol Alp Pannonica Adriat. 2018;27(2):79-83.

60. Kroemer S, Frühauf J, Campbell TM, Massone C, Schwantzer G, Soyer HP, et al. Mobile teledermatology for skin tumour screening: diagnostic accuracy of clinical and dermoscopic image tele-evaluation using cellular phones. Br J Dermatol. 2011;164(5):973-9.

61. Krupinski EA, LeSueur B, Ellsworth L, Levine N, Hansen R, Silvis N, et al. Diagnostic accuracy and image quality using a digital camera for teledermatology. Telemed J. 1999;5(3):257-63.

62. Kvedar JC, Edwards RA, Menn ER, Mofid M, Gonzalez E, Dover J, et al. The substitution of digital images for dermatologic physical examination. Arch Dermatol. 1997;133(2):161-7.

63. Lamel SA, Haldeman KM, Ely H, Kovarik CL, Pak H, Armstrong AW. Application of mobile teledermatology for skin cancer screening. J Am Acad Dermatol. 2012;67(4):576-81.

64. Lasierra N, Alesanco A, Gilaberte Y, Magallón R, García J. Lessons learned after a three-year store and forward teledermatology experience using internet: Strengths and limitations. Int J Med Inform. 2012;81(5):332-43.

65. Lepe V, Moncada B, Castanedo-Cázares JP, Martínez-Rodríguez A, Mercado-Ceja SM, Gordillo-Moscoso A. [First study of teledermatology in Mexico. A new public health tool]. Gac Med Mex. 2004;140(1):23-6.

66. Lesher JL, Jr., Davis LS, Gourdin FW, English D, Thompson WO. Telemedicine evaluation of cutaneous diseases: a blinded comparative study. J Am Acad Dermatol. 1998;38(1):27-31.

67. Lim AC, Egerton IB, See A, Shumack SP. Accuracy and reliability of store-and-forward teledermatology: preliminary results from the St George Teledermatology Project. Australas J Dermatol. 2001;42(4):247-51.

68. Loane MA, Gore HE, Corbett R, Steele K, Mathews C, Bloomer SE, et al. Preliminary results from the Northern Ireland arms of the UK Multicentre Teledermatology Trial: effect of camera performance on diagnostic accuracy. J Telemed Telecare. 1997;3:73-5.

69. Loane MA, Corbett R, Bloomer SE, Eedy DJ, Gore HE, Mathews C, et al. Diagnostic accuracy and clinical management by realtime teledermatology. Results from the Northern Ireland arms of the UK Multicentre Teledermatology Trial. J Telemed Telecare. 1998;4(2):95-100.

70. Lowitt MH, Kessler IZ, Kauffman CL, Hooper FJ, Siegel E, Burnett JW. Teledermatology and in-person examinations: A comparison of patient and physician perceptions and diagnostic agreement. Archives of Dermatology. 1998;134(4):471-6.

71. Lyon CC, Harrison PV. Digital imaging and teledermatology: educational and diagnostic applications of a portable digital imaging system for the trainee dermatologist. Clin Exp Dermatol. 1997;22(4):163-5.

72. MacLellan AN, Price EL, Publicover-Brouwer P, Matheson K, Ly TY, Pasternak S, et al. The use of noninvasive imaging techniques in the diagnosis of melanoma: a prospective diagnostic accuracy study. J Am Acad Dermatol. 2021;85(2):353-9.

73. Mahendran R, Goodfield MJD, Sheehan-Dare RA. An evaluation of the role of a store-and-forward teledermatology system in skin cancer diagnosis and management. Clinical and Experimental Dermatology. 2005;30(3):209-14.

74. Mallett RB. Teledermatology in practice. Clin Exp Dermatol. 2003;28(4):356-9.

75. Manahan MN, Soyer HP, Loescher LJ, Horsham C, Vagenas D, Whiteman DC, et al. A pilot trial of mobile, patient-performed teledermoscopy. Br J Dermatol. 2015;172(4):1072-80.

76. Marchell R, Locatis C, Burges G, Maisiak R, Liu WL, Ackerman M. Comparing High Definition Live Interactive and Store-and-Forward Consultations to In-Person Examinations. Telemed J E Health. 2017;23(3):213-8.

77. Markun S, Scherz N, Rosemann T, Tandjung R, Braun RP. Mobile teledermatology for skin cancer screening: A diagnostic accuracy study. Medicine (Baltimore). 2017;96(10):e6278.

78. Massone C, Hofmann-Wellenhof R, Ahlgrimm-Siess V, Gabler G, Ebner C, Soyer HP. Melanoma screening with cellular phones. PLoS One. 2007;2(5):e483.

79. Massone C, Maak D, Hofmann-Wellenhof R, Soyer HP, Frühauf J. Teledermatology for skin cancer prevention: an experience on 690 Austrian patients. J Eur Acad Dermatol Venereol. 2014;28(8):1103-8.

80. Montejano RD, Oh DH, Twigg AR. Limited impact of teledermoscopy on referrals to face-to-face dermatology. Dermatol Online J. 2022;28(5).

81. Moreno-Ramirez D, Ferrandiz L, Perez Bernal A, Carrasco Duran R, Rios Martín JJ, Camacho F. Teledermatology as a filtering system in pigmented lesion clinics. Journal of Telemedicine and Telecare. 2005;11(6):298-303.

82. Moreno-Ramirez D, Ferrandiz L, Galdeano R, Camacho FM. Teledermatoscopy as a triage system for pigmented lesions: a pilot study. Clin Exp Dermatol. 2006;31(1):13-8.

83. Moreno-Ramirez D, Ferrandiz L, Nieto-Garcia A, Carrasco R, Moreno-Alvarez P, Galdeano R, et al. Store-and-forward teledermatology in skin cancer triage: experience and evaluation of 2009 teleconsultations. Arch Dermatol. 2007;143(4):479-84.

84. Muir J, Xu C, Paul S, Staib A, McNeill I, Singh P, et al. Incorporating teledermatology into emergency medicine. Emerg Med Australas. 2011;23(5):562-8.

85. Naka F, Lu J, Porto A, Villagra J, Wu ZH, Anderson D. Impact of dermatology eConsults on access to care and skin cancer screening in underserved populations: A model for teledermatology services in community health centers. J Am Acad Dermatol. 2018;78(2):293-302.

86. Nami N, Massone C, Rubegni P, Cevenini G, Fimiani M, Hofmann-Wellenhof R. Concordance and time estimation of store-and-forward mobile teledermatology compared to classical face-to-face consultation. Acta Derm Venereol. 2015;95(1):35-9.

87. Ng MF, Stevenson JH. Diagnostic value and cost-effectiveness of good quality digital images accompanying electronic referrals for suspected skin malignancies. Ann Plast Surg. 2011;66(4):377-80.

88. Nordal EJ, Moseng D, Kvammen B, Løchen ML. A comparative study of teleconsultations versus face-to-face consultations. J Telemed Telecare. 2001;7(5):257-65.

89. Norton SA, Burdick AE, Phillips CM, Berman B. Teledermatology and underserved populations. Arch Dermatol. 1997;133(2):197-200.

90. O'Connor DM, Jew OS, Perman MJ, Castelo-Soccio LA, Winston FK, McMahon PJ. Diagnostic Accuracy of Pediatric Teledermatology Using Parent-Submitted Photographs: A Randomized Clinical Trial. JAMA Dermatol. 2017;153(12):1243-8.

91. Oakley AM, Astwood DR, Loane M, Duffill MB, Rademaker M, Wootton R. Diagnostic accuracy of teledermatology: results of a preliminary study in New Zealand. N Z Med J. 1997;110(1038):51-3.

92. Oakley AM, Duffill MB, Reeve P. Practising dermatology via telemedicine. N Z Med J. 1998;111(1071):296-9.

93. Oakley AM, Reeves F, Bennett J, Holmes SH, Wickham H. Diagnostic value of written referral and/or images for skin lesions. J Telemed Telecare. 2006;12(3):151-8.

94. Okita AL, Molina Tinoco LJ, Patatas OH, Guerreiro A, Criado PR, Gabbi TV, et al. Use of Smartphones in Telemedicine: Comparative Study Between Standard and Teledermatological Evaluation of High-Complex Care Hospital Inpatients. Telemed J E Health. 2016;22(9):755-60.

95. Oztas MO, Calikoglu E, Baz K, Birol A, Onder M, Calikoglu T, et al. Reliability of Web-based teledermatology consultations. J Telemed Telecare. 2004;10(1):25-8.

96. Pak HS, Harden D, Cruess D, Welch ML, Poropatich R. Teledermatology: an intraobserver diagnostic correlation study, part I. Cutis. 2003;71(5):399-403.

97. Paradela-De-La-Morena S, Fernandez-Torres R, Martínez-Gómez W, Fonseca-Capdevila E. Teledermatology: diagnostic reliability in 383 children. Eur J Dermatol. 2015;25(6):563-9.

98. Phillips CM, Burke WA, Shechter A, Stone D, Balch D, Gustke S. Reliability of dermatology teleconsultations with the use of teleconferencing technology. J Am Acad Dermatol. 1997;37(3):398-402.

99. Phillips CM, Burke WA, Allen MH, Stone D, Wilson JL. Reliability of telemedicine in evaluating skin tumors. Telemed J. 1998;4(1):5-9.

100. Preclaro IAC, Gulmatico-Flores Z, Tianco EAV. Concordance and Accuracy of Teledermatology Using Mobile Phones in the Outpatient Clinic of Jose R Reyes Memorial Medical Center: Cross-sectional Study. JMIR Dermatol. 2022;5(4):e32546.

101. Rajagopal R, Sood A, Arora S. Teledermatology in Air Force: Our Experience. Med J Armed Forces India. 2009;65(4):342-6.

102. Rashid E, Ishtiaq O, Gilani S, Zafar A. Comparison of store and forward method of teledermatology with face-to-face consultation. J Ayub Med Coll Abbottabad. 2003;15(2):34-6.

103. Ribas J, Schettini APM, Cunha MDGS, Ribas CBDR. Agreement between dermatological diagnoses made by live examination compared to analysis of digital images. Anais Brasileiros de Dermatologia. 2010;85(4):441-7.

104. Ríos-Yuil JM. [Correlation between face-to-face assessment and telemedicine for the diagnosis of skin disease in case conferences]. Actas Dermosifiliogr. 2012;103(2):138-43.

105. Romero G, García M, Vera E, Martínez C, Cortina. Preliminary results of DERMATEL: prospective randomized study comparing synchronous and asynchronous modalities of teledermatology. Actas dermo-sifiliográficas. 2006;97(10):630‐6.

106. Romero G, Sánchez P, García M, Cortina P, Vera E, Garrido JA. Randomized controlled trial comparing store-and-forward teledermatology alone and in combination with web-camera videoconferencing. Clin Exp Dermatol. 2010;35(3):311-7.

107. Romero Aguilera G, Cortina de la Calle P, Vera Iglesias E, Sánchez Caminero P, García Arpa M, Garrido Martín JA. Interobserver reliability of store-and-forward teledermatology in a clinical practice setting. Actas Dermosifiliogr. 2014;105(6):605-13.

108. Rubegni P, Nami N, Cevenini G, Poggiali S, Hofmann-Wellenhof R, Massone C, et al. Geriatric teledermatology: store-and-forward vs. face-to-face examination. J Eur Acad Dermatol Venereol. 2011;25(11):1334-9.

109. Ruiz C, Gaviria C, Gaitán M, Manrique R, Zuluaga Á, Trujillo A. Concordance studies of a web based system in teledermatology. Colombia Medica. 2009;40(3):259-70.

110. Saleh N, Abdel Hay R, Hegazy R, Hussein M, Gomaa D. Can teledermatology be a useful diagnostic tool in dermatology practice in remote areas? An Egyptian experience with 600 patients. J Telemed Telecare. 2017;23(2):233-8.

111. Santosa A, Li Z, Chandran NS. Teledermatology in an emergency department: benefits and gaps. BMC Emerg Med. 2023;23(1):115.

112. Schiener R, Bredlich RO, Pillekamp H, Peter RU. [Evaluation of a telemedicine pilot project]. Hautarzt. 2001;52(1):26-30.

113. Senel E, Sabancılar E, Mansuroğlu C, Demir E. A preliminary study of the contribution of telemicroscopy to the diagnosis and management of skin tumours in teledermatology. J Telemed Telecare. 2014;20(4):178-83.

114. Shin H, Kim DH, Ryu HH, Yoon SY, Jo SJ. Teledermatology consultation using a smartphone multimedia messaging service for common skin diseases in the Korean army: a clinical evaluation of its diagnostic accuracy. J Telemed Telecare. 2014;20(2):70-4.

115. Silva CS, Souza MB, Duque IA, de Medeiros LM, Melo NR, Araújo Cde A, et al. [Teledermatology: diagnostic correlation in a primary care service]. An Bras Dermatol. 2009;84(5):489-93.

116. Silveira CEG, Silva TB, Fregnani JHGT, Vieira RAC, Haikel RL, Syrjänen K, et al. Digital photography in skin cancer screening by mobile units in remote areas of Brazil. BMC Dermatology. 2014;14(1).

117. Sola-Ortigosa J, Muñoz-Santos C, Masat-Ticó T, Isidro-Ortega J, Guilabert A. The Role of Teledermatology and Teledermoscopy in the Diagnosis of Actinic Keratosis and Field Cancerization. Journal of Investigative Dermatology. 2020;140(10):1976-84.e4.

118. Taberner Ferrer R, Pareja Bezares A, Llambrich Mañes A, Vila Mas A, Torné Gutiérrez I, Nadal Lladó C, et al. Diagnostic reliability of an asynchronous teledermatology consultation. Atencion Primaria. 2009;41(10):552-7.

119. Tait CP, Clay CD. Pilot study of store and forward teledermatology services in Perth, Western Australia. Australas J Dermatol. 1999;40(4):190-3.

120. Tan E, Yung A, Jameson M, Oakley A, Rademaker M. Successful triage of patients referred to a skin lesion clinic using teledermoscopy (IMAGE IT trial). Br J Dermatol. 2010;162(4):803-11.

121. Taslidere N, Su Kucuk O. Can a Correct Diagnosis Be Established Using the Teledermatology Method? Acta Dermatovenerol Croat. 2022;30(1):32-9.

122. Taslidere N, Kucuk OS. Investigation of the effectiveness of teledermatology in the diagnosis of skin lesions in pediatric patients. Rev Assoc Med Bras (1992). 2023;69(10):e20230253.

123. Taylor P, Goldsmith P, Murray K, Harris D, Barkley A. Evaluating a telemedicine system to assist in the management of dermatology referrals. Br J Dermatol. 2001;144(2):328-33.

124. Teague R, Wang M, Wen D, Sunderland M, Rolfe G, Oakley AMM, et al. Virtual lesion clinic – Evaluation of a teledermatology triage system for referrals for suspected melanoma. Australasian Journal of Dermatology. 2022;63(1):e33-e40.

125. Teoh NSC, Oakley A. A 9-Year Teledermoscopy Service in New Zealand: Retrospective Service Review. JMIR Dermatol. 2022;5(4):e36351.

126. Tian B. Tele-aesthetics in South Asia. J Cosmet Dermatol. 2017;16(1):21-5.

127. Tucker WFG, Lewis FM. Digital imaging: A diagnostic screening tool? International Journal of Dermatology. 2005;44(6):479-81.

128. Vañó-Galván S, Hidalgo A, Aguayo-Leiva I, Gil-Mosquera M, Ríos-Buceta L, Plana MN, et al. [Store-and-forward teledermatology: assessment of validity in a series of 2000 observations]. Actas Dermosifiliogr. 2011;102(4):277-83.

129. Villa L, Matz O, Olaciregui Dague K, Kluwig D, Rossaint R, Brokmann JC. The assessment of dermatological emergencies in the emergency department via telemedicine is safe: a prospective pilot study. Intern Emerg Med. 2020;15(7):1275-9.

130. Wang M, Gendreau JL, Gemelas J, Capulong D, Lau C, Mata-Diaz S, et al. Diagnosis and Management of Malignant Melanoma in Store-and-Forward Teledermatology. Telemed J E Health. 2017;23(11):877-80.

131. Warshaw EM, Lederle FA, Grill JP, Gravely AA, Bangerter AK, Fortier LA, et al. Accuracy of teledermatology for pigmented neoplasms. J Am Acad Dermatol. 2009;61(5):753-65.

132. Warshaw EM, Gravely AA, Nelson DB. Reliability of store and forward teledermatology for skin neoplasms. J Am Acad Dermatol. 2015;72(3):426-35.

133. Weingast J, Scheibböck C, Wurm EMT, Ranharter E, Porkert S, Dreiseitl S, et al. A prospective study of mobile phones for dermatology in a clinical setting. Journal of Telemedicine and Telecare. 2013;19(4):213-8.

134. Whited JD, Mills BJ, Hall RP, Drugge RJ, Grichnik JM, Simel DL. A pilot trial of digital imaging in skin cancer. J Telemed Telecare. 1998;4(2):108-12.

135. Whited JD, Hall RP, Simel DL, Foy ME, Stechuchak KM, Drugge RJ, et al. Reliability and accuracy of dermatologists' clinic-based and digital image consultations. J Am Acad Dermatol. 1999;41(5):693-702.

136. Yamazaki Y, Saida T, Takizawa M, Murase S. [Inter-hospital teledermatology conference using a videophone network]. Igaku Butsuri. 2003;23(1):40-3.

137. Zanini M. Analyze of diagnostic concordance between face-to-face and teledermatology diagnosis. Medicina Cutanea Ibero-Latino-Americana. 2013;41(2):60-2.

138. Zelickson BD, Homan L. Teledermatology in the nursing home. Arch Dermatol. 1997;133(2):171-4.

139. Zink A, Kolbinger A, Leibl M, Leon-Suarez I, Gloning J, Merkel C, et al. The value of teledermatology using a mobile app compared to conventional dermatology. Eur J Dermatol. 2017;27(4):429-31.

140. Zink A, Kolbinger A, Leibl M, Léon Suarez I, Gloning J, Merkel C, et al. [Teledermoscopy by mobile phones : Reliable help in the diagnosis of skin lesions?]. Hautarzt. 2017;68(11):890-5.

141. Alfageme F, Minguela E, Martínez C, Salgüero I, Calvo A, León F, et al. Dermatologic Ultrasound in Primary Care: A New Modality of Teledermatology: A Prospective Multicenter Validation Study. Journal of ultrasound in medicine : official journal of the American Institute of Ultrasound in Medicine. 2021;40(2):351-6.

142. Creadore A, Manjaly P, Tkachenko E, Li DG, Kaffenberger B, Shinkai K, et al. The utility of augmented teledermatology to improve dermatologist diagnosis of cellulitis: a cross-sectional study. Arch Dermatol Res. 2023;315(5):1347-53.

143. Giavina-Bianchi M, Sousa R, Cordioli E. Part I: Accuracy of Teledermatology in Inflammatory Dermatoses. Front Med (Lausanne). 2020;7:585792.

144. Giavina-Bianchi M, Giavina-Bianchi P, Santos AP, Rizzo LV, Cordioli E. Accuracy and efficiency of telemedicine in atopic dermatitis. JAAD Int. 2020;1(2):175-81.

145. Lozzi GP, Soyer P, Massone C, Micantonio T, Kraenke B, Fargnoli MC, et al. The additive value of second opinion teleconsulting in the management of patients with challenging inflammatory, neoplastic skin diseases: A best practice model in dermatology? Journal of the European Academy of Dermatology and Venereology. 2007;21(1):30-4.

146. Łudzik J, Witkowski AM, Roterman-Konieczna I. New telemedicine techniques in dermatology - evaluation with reflectance confocal microscopy via cloud-based platform. Folia Med Cracov. 2016;56(3):21-9.

147. Łudzik J, Witkowski AM, Roterman-Konieczna I, Bassoli S, Farnetani F, Pellacani G. Improving Diagnostic Accuracy of Dermoscopically Equivocal Pink Cutaneous Lesions with Reflectance Confocal Microscopy in Telemedicine Settings: Double Reader Concordance Evaluation of 316 Cases. PLoS One. 2016;11(9):e0162495.

148. Rao BK, Mateus R, Wassef C, Pellacani G. In vivo confocal microscopy in clinical practice: comparison of bedside diagnostic accuracy of a trained physician and distant diagnosis of an expert reader. J Am Acad Dermatol. 2013;69(6):e295-300.

149. Şenel E, Baba M, Durdu M. The contribution of teledermatoscopy to the diagnosis and management of non-melanocytic skin tumours. J Telemed Telecare. 2013;19(1):60-3.

150. Shah VK, Jaklitsch E, Agarwal A, Chen J, James AJ, Ferris LK, et al. Descriptive and Concordance Data for Asynchronous Teledermatology Consultations for Dermatitis: A Retrospective Study. Telemed J E Health. 2023.

151. Tognetti L, Cartocci A, Balistreri A, Cataldo G, Cinotti E, Moscarella E, et al. The Comparative Use of Multiple Electronic Devices in the Teledermoscopic Diagnosis of Early Melanoma. Telemed J E Health. 2021;27(5):495-502.

152. Trindade MA, Wen CL, Neto CF, Escuder MM, Andrade VL, Yamashitafuji TM, et al. Accuracy of store-and-forward diagnosis in leprosy. J Telemed Telecare. 2008;14(4):208-10.

153. Tugrul B, Yalici-Armagan B, Demirdag HG, Gunduz O. Evaluation of Diagnostic Accuracy and Therapeutic Approach of Dermatologists and Plastic Surgeons To Non-Melanocytic Skin Lesions By Using Telemedicine. Dermatol Pract Concept. 2022;12(3):e2022124.

154. van der Heijden JP, Thijssing L, Witkamp L, Spuls PI, de Keizer NF. Accuracy and reliability of teledermatoscopy with images taken by general practitioners during everyday practice. J Telemed Telecare. 2013;19(6):320-5.

155. Warshaw EM, Lederle FA, Grill JP, Gravely AA, Bangerter AK, Fortier LA, et al. Accuracy of teledermatology for nonpigmented neoplasms. J Am Acad Dermatol. 2009;60(4):579-88.

156. Witkowski AM, Łudzik J, Arginelli F, Bassoli S, Benati E, Casari A, et al. Improving diagnostic sensitivity of combined dermoscopy and reflectance confocal microscopy imaging through double reader concordance evaluation in telemedicine settings: A retrospective study of 1000 equivocal cases. PLoS One. 2017;12(11):e0187748.
